# Endemics determine bioregionalization in the alpine zone of the Irano-Anatolian biodiversity hotspot (South-West Asia)

**DOI:** 10.1007/s00035-021-00266-7

**Published:** 2021-08-04

**Authors:** Jalil Noroozi, Sina Khalvati, Haniyeh Nafisi, Akram Kaveh, Behnaz Nazari, Golshan Zare, Masoud Minaei, Ernst Vitek, Gerald M. Schneeweiss

**Affiliations:** 1 Department of Botany and Biodiversity Research, University of Vienna, Vienna, Austria; 2 Department of Biology, Bu-Ali Sina University, Hamedan, Iran; 3 Department of Plant Biology, Faculty of Biological Sciences, Tarbiat Modares University, Tehran, Iran; 4 Department of Pharmaceutical Botany, Faculty of Pharmacy, Hacettepe University, Ankara, Turkey; 5 Department of Geography, Ferdowsi University of Mashhad, Mashhad, Iran; 6 Natural History Museum of Vienna, Vienna, Austria; 7 Geographic Information Science/System and Remote Sensing Laboratory (GISSRS: lab), Ferdowsi University of Mashhad, Mashhad, Iran

**Keywords:** Alpine habitats, Areas of endemism, Bioregions, Endemicity analysis, Global biodiversity hotspots, Network-clustering

## Abstract

Alpine habitats are characterized by a high rate of range restricted species compared to those of lower elevations. This is also the case for the Irano-Anatolian global biodiversity hotspot in South-West Asia, which is a mountainous area harbouring a high amount of endemic species. Using two quantitative approaches, Endemicity Analysis and Network-Clustering, we want to identify areas of concordant species distribution patterns in the alpine zone of this region as well as to test the hypothesis that, given the high proportion of endemics among alpine species, delimitation of these areas is determined mainly by endemic alpine species, i.e., areas of concordant species distribution patterns are congruent with areas of endemism. Endemicity Analysis identified six areas of concordant species distribution patterns irrespective of dataset (total alpine species versus endemic alpine species), whereas the Network-Clustering approach identified five and four Bioregions from total alpine species and endemic alpine species, respectively. Most of these areas have been previously identified using the endemic flora of different elevational zones. The identified units using both methods and both datasets are strongly congruent, proposing that they reveal meaningful distribution patterns. Bioregionalization in the Irano-Anatolian biodiversity hotspot appears to be strongly influenced by the endemic alpine species, a pattern likely to hold in alpine regions outside the Irano-Anatolian hotspot.

**Supplementary Information:**

The online version contains supplementary material available at 10.1007/s00035-021-00266-7.

## Introduction

High mountains are biodiversity hotspots with a high endemic richness (Antonelli et al. 2018; Muellner-Riehl et al. 2019; Rahbek et al. 2019). This is largely due to alpine ecosystems (i.e., those above the treeline), which are distributed across all continents and latitudes and cover almost three percent of the terrestrial land outside the Antarctic (Körner et al. 2011; Testolin et al. 2020). Alpine habitats are characterized by a high rate of endemism compared to the lower zones in most mountain ranges (Körner 2003; Tribsch and Schönswetter 2003; Hobohm 2014; Noroozi et al. 2018), which is due to the complex origins of high elevations, climate changes, geographic isolation, and considerable microhabitat variation (Agakhanjanz and Breckle 1995; Körner 1995; Dirnböck et al. 2011; Sandel et al. 2011).

The Irano-Anatolian region is a mountainous area in South-West (SW) Asia and one of the 35 global biodiversity hotspots (Mittermeier et al. 2011). More than 40% of the plant species of this area are endemic (Mittermeier et al. 2005), and the rate of endemism increases sharply with increasing elevation (Noroozi et al. 2018, 2019a, c). The higher extinction risk of range-restricted species compared to widely distributed species emphasizes their relevance for conservation biology (Myers et al. 2000; Brooks et al. 2006). Climate change is one of the most important threats for global biodiversity (Thomas et al. 2004; Willis and Bhagwat 2009; Urban 2015), and alpine species are among the most sensitive organisms to this phenomenon (Dullinger et al. 2012; Gottfried et al. 2012; Ernakovich et al. 2014). Therefore, identifying areas of endemism of these habitats is very important for conservation biology.

Areas of endemism (AEs), i.e., areas with at least two co-occurring species being essentially restricted to this area (Szumik et al. 2002; Szumik and Goloboff 2004), are principal units of analyses in historical biogeography (Nelson and Platnick 1981; Morrone 1994; Morrone 2008), which can be used for designing biogeographical regionalization schemes (Morrone 2014). Recognition of AEs has been popular in the recent decades (Harold and Mooi 1994; Morrone 2008) to explain their evolutionary and ecological causes (Nelson and Platnick 1981; Major 1988; Anderson 1994) and because of their significance in defining conservation priorities (Linder 2001; Jetz et al. 2004). A wide array of methods is available to identify AEs (Morrone 1994; Szumik et al. 2002; Hausdorf and Hennig 2003; Dos Santos et al. 2008; Dos Santos et al. 2012; Torres-Miranda et al. 2013; Vilhena and Antonelli 2015; Salinas and Wheeler 2019). The same toolbox can be used, however, to identify areas of concordant species distribution patterns (hereinafter referred to as ACDs) from a dataset including also non-endemic biota (for an example see Aagesen et al. 2009), which is expected to improve the identification of biogeographic units (Vilhena and Antonelli 2015). Although AEs are also ACDs, but based on endemic species only, we will hereinafter use ACDs for those cases, where, also or exclusively, non-endemics are considered.

In this study, we want to identify ACDs and AEs in the alpine habitats of the Irano-Anatolian region using two quantitative approaches, Endemicity Analysis (Szumik et al. 2002) and Network-Clustering (Vilhena and Antonelli 2015; Edler et al. 2017), to answer the following questions: (1) Where are the main ACDs and AEs of the alpine flora in the Irano-Anatolian region? (2) Do inferences based on the total alpine flora differ from those of the endemic alpine flora, i.e., are ACDs congruent with AEs? As the study area is a global biodiversity hotspot with a high proportion of endemic species and as the rate of endemism is particularly high at higher elevations (i.e., in the alpine zone), it can be expected that ACDs and AEs will be largely congruent, i.e., that ACDs will be determined essentially by alpine endemics.

## Methods

### Study area

**Fig. 1.**
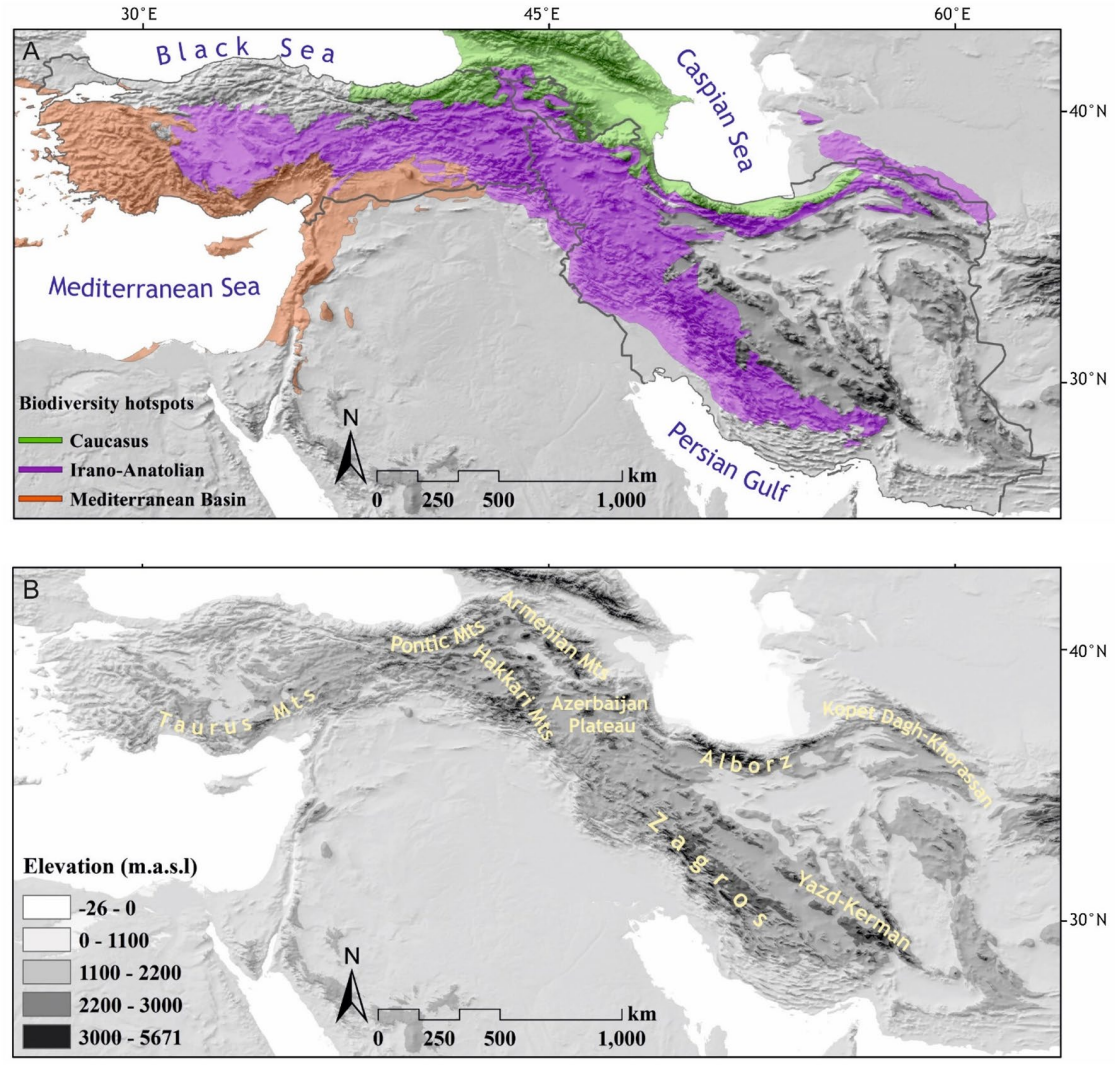
Study area with **A** its global biodiverstiy hotspots and **B** its main high mountain ranges

The Irano-Anatolian Plateau is a mountainous region in SW Asia and a meeting point of three biogeographical regions (Irano-Anatolian, Euro-Siberian and Saharo-Sindian; Zohary 1973) and of three global biodiversity hotspots (Irano-Anatolian, Caucasian and Mediterranean; Mittermeier et al. 2005). The major part of the region is covered by the Irano-Anatolian global biodiversity hotspot, comprising high elevations of central and eastern Turkey, Armenia, north-eastern Iraq, and of Iran (Fig. 1). Being part of the Alpine-Himalayan orogenic system, the uplift of high mountain ranges of the region occurred from the Late Oligocene to the Late Miocene (Stöcklin 1968; Berberian and King 1981). Major mountain ranges of the area are Taurus Mountains, Pontic Mountains, Hakkari Mountains, Armenian Mountains, Azerbaijan Plateau, Alborz, Kopet Dagh-Khorassan, Zagros and the Yazd-Kerman Massif (Fig. 1).

### Plant distribution data

The (sub)alpine species, i.e., species with mean elevational distribution above 2300 m a.s.l. for Turkey and the Armenian Mountains, above 2500 m a.s.l. for Alborz, the Azerbaijan Plateau and Kopet Dagh-Khorassan, above 2700 m a.s.l. for Zagros, and above 3000 m a.s.l. for the Yazd-Kerman Massif, were compiled. These thresholds were chosen based on the cutoff for the subalpine–alpine habitats established by previous publications (Noroozi et al. 2016; Fayvush and Aleksanyan 2020; Parolly 2020). The distribution data of these species were extracted from different floras (ca. 80% of data points; Rechinger 1963-2015; Davis 1965-1985; Assadi et al. 1989-2018), relevant publications pertaining to species and records published after these floras (ca. 5% of data points), and our own data (ca. 15% of data points). Thus, the total (sub)alpine species dataset included 1672 species (Table S1) from 19,680 localities, georeferenced with a precision of at least 0.25° × 0.25° (records that could not be georeferenced to at least this level of resolution were not included). Of those species, 1267 species (76%) were categorized as endemic or subendemic (i.e., having no more than 20% of its distribution range outside the study region) corresponding to 14,285 localities (the endemic alpine species); the complimentary set of 405 species and 5395 localities constituted the non-endemic alpine species. Most of the studied species are rare and known only from a few localities: 53% of the species have maximally 5 records, 70% of species have maximally 10 records, and 83% of species have maximally 20 records. The number of records per species is given in Table S1.

### Data analyses

Endemicity Analysis (Szumik et al. 2002), as implemented in NDM/VNDM3 (Szumik and Goloboff 2004), is one of the most commonly applied approaches to identify AEs (Martínez-Hernández et al. 2015; Szumik and Goloboff 2015; Elías and Aagesen 2016; Hoffmeister and Ferrari 2016; Zhang et al. 2016; Weirauch et al. 2017; Noroozi et al. 2018, 2019c; Lago-Barcia et al. 2020). In this method, an endemicity score for each assumed AE (i.e., set of grid cells) is measured as the sum of the endemicity scores of each contributing species (Szumik and Goloboff 2004). In this study, the data were analysed with a cell size of 1.23° × 1.23°, the optimal size as determined by the program. The following parameters were used: a set must have at least 10 contributing species with an endemicity score of at least 2 (the threshold of 10 species was determined experimentally as the number at which delimitation of AEs became stable); temporarily saving sets within 0.99 of the current score; keeping overlapping subsets when 30% of the species were unique; using 100 replicates. To reduce the level of redundancy in the inferred AEs or ACDs, consensus areas were assembled using the loose consensus rule (considered sufficiently detailed for large-scale studies: Aagesen et al. 2013), i.e., areas were added when each area shares at least 10% of its defining species with at least one, but not necessarily all, of the other areas in the consensus.

As a second method we used the Network-Clustering approach (Vilhena and Antonelli 2015) implemented in the web application Infomap Bioregions (Edler et al. 2017; https://www.mapequation.org/bioregions/). This method generates a bipartite network (i.e., a network describing the associations between species and locations), where primary locations (point data or polygons describing distribution ranges) are discretized into bins, i.e., quadratic grid cells; this discretizing may be done using adaptive binning, where each grid cell can be, but does not have to be, recursively divided into four cells, thus accounting for different spatial resolutions of the input data (Vilhena and Antonelli 2015). Then, groups of nodes of this bipartite network are partitioned into clusters using the map equation (Rosvall and Bergstrom 2008, 2011). The map equation specifies the (theoretical) limit of how concisely the trajectory of a random walker on a network can be described (Rosvall et al. 2009); minimizing the map equation over all possible partitions allows an optimal partitioning scheme to be identified. Finally, these clusters are mapped to geographic space, where they then constitute bioregions. For the present study, the maximum and the minimum cell size were set to 1° (only cell sizes in increments of doubling the size from 1/8° onward are possible, i.e., 1/8, 1/4, 1/2, 1, 2, 4, etc.); whereas this effectively prevents adaptive binning, it is a good compromise between resolution of the input data (minimum resolution of the georeferenced records is 0.25°; see above) and the correspondence between grid cells and the distribution of subalpine–alpine regions. Using a fixed cell size permitted us to retain only a single occurrence per species and grid cell (filtered manually), as densely sampled grid cells may be identified as separate clusters, even if they hardly differ in their species composition from neighbouring cells as was observed for one grid cell in central Alborz, for which a large dataset from vegetation relevés was available (data not shown). Minimum and maximum cell capacity, i.e., the minimum and maximum number of observations per cell, were set to 40 (ca. 0.2% of all records) and 400, respectively; different combinations of cell capacities were tested, and this combination was identified as one around which the number and the delimitation of clusters remained stable. Clustering was achieved using 10 trials and applying a cluster cost of 1.0; this cost allows tuning the clustering algorithm to search for fewer (by increasing the cost) or more (by decreasing the cost) clusters. As this approach cannot infer overlapping units and instead may identify transitional zones as separate bioregions (Vilhena and Antonelli 2015; Bloomfield et al. 2018; Droissart et al. 2018), clusters with a proportion of unique species below 30% were considered as transitional zones.

## Results

**Fig 2.**
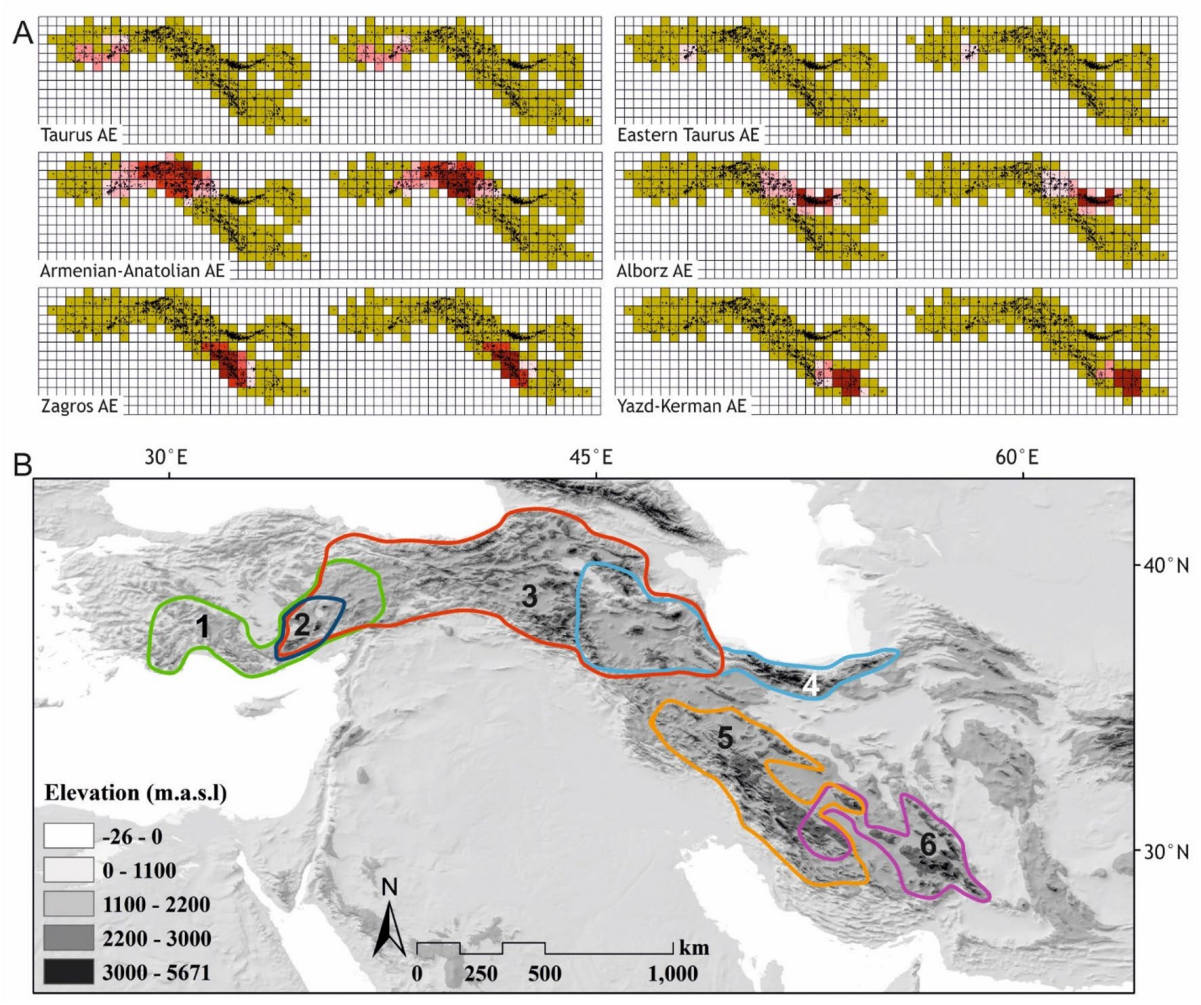
Areas of concordant species distribution patterns (ACDs) and areas of endemism (AEs) of the Irano-Anatolian region identified using Endemicity Analysis. **A** Single ACDs (inferred from total alpine species) are shown in the left map, the single AEs (inferred from the endemic alpine species) are shown on the right map; light pink to dark red coloured grid cells indicate increasing endemicity scores. **B** Territories of the identified AEs

Endemicity Analysis identified six ACDs and six AEs for the total alpine and the endemic alpine flora, respectively. The identified areas were highly congruent among the two species datasets covering almost the same territories (Fig. 2; Table 1) and will, therefore, be referred to as AEs. These six areas were: (1) Taurus AE, covering the Taurus Mountains in southern Turkey, situated close to or even within the Mediterranean biodiversity hotspot; (2) Eastern Taurus AE, nested within the former; (3) Anatolian–Armenian AE, covering the high mountains of eastern Taurus, the Pontic Mountains, the Hakkari Mountains, as well as mountain ranges of Armenia, north-eastern Iraq and north-western Iran reaching the Talysh Mountains; this was the largest AE with a high amount of alpine endemics; (4) Alborz AE, covering the entire Alborz range plus the Azerbaijan Plateau in north-western Iran; (5) Zagros AE, encompassing high elevations of the Zagros mountains in western Iran; and (6) Yazd-Kerman AE including the Yazd-Kerman massifs in southern Iran. The list of species contributing to the scores of each ACD and AE is provided in Tables S2 and S3.

**Table 1:** Areas of concordant species distribution patterns (ACDs) and areas of endemism (AE; numbers as in Fig. 2) identified by Endemicity Analysis from the total alpine and the endemic alpine species, respectively

No.	Name	Total alpine species				Endemic alpine species			
		Supported areas	No. of cells	(Sub)alpine area size (km^2^)	Supporting species	Supported areas	No. of cells	(Sub)alpine area size (km^2^)	Supporting species
1	Taurus AE	4	13	2886	16	3	12	2886	16
2	Eastern Taurus AE	1	4	1980	15	1	4	1980	15
3	Anatolian–Armenian AE	194	43	59,406	374	131	35	59,202	239
4	Alborz AE	33	23	19,734	122	23	21	19,695	107
5	Zagros AE	18	18	11,627	64	17	16	11,460	63
6	Yazd-Kerman AE	6	15	2956	39	5	11	2745	27

**Fig. 3.**
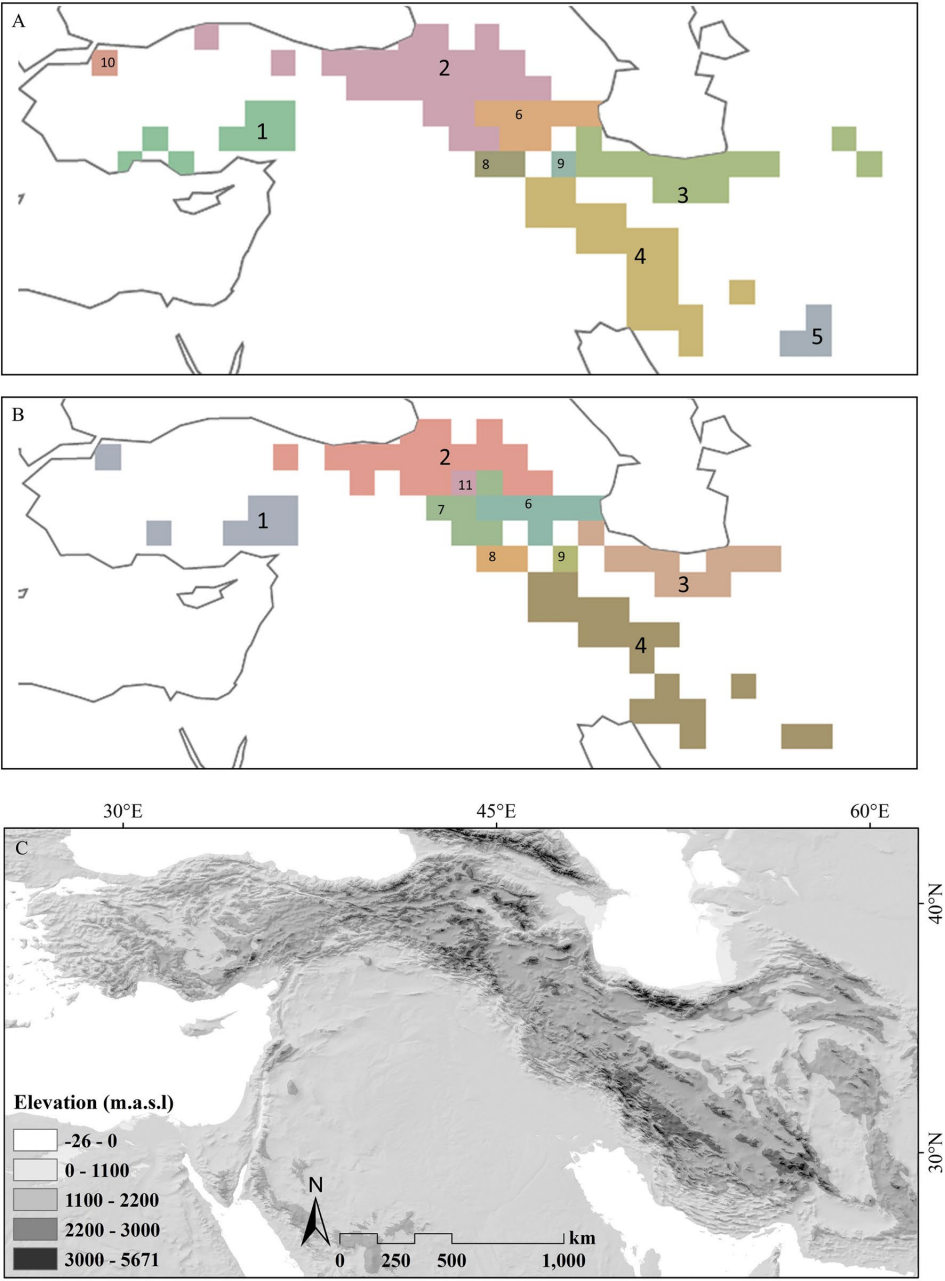
Bioregions (BR; numbers 1–5) and transitional zones (numbers 6–11) of the Irano-Anatolian region identified using Network-Clustering from **A** total alpine species and **B** endemic alpine species. These BRs (indicated in big numbers) are: 1, Taurus BR; 2, Anatolian–Armenian BR; 3, Alborz BR; 4, Zagros BR; 5, Yazd-Kerman BR. **C** Topographic map of the region

Using the Network-Clustering approach implemented in InfoMap, a total of nine units were identified for each dataset. For the total alpine flora, five units were considered as Bioregions (BR; with unique species proportion ranging from 31 to 51%; Table 2) and four units considered as transitional zones (with unique species proportion ranging from 4 to 14%; Table 2), whereas for the endemic alpine flora, four units were considered as BRs (with unique species proportion ranging from 34 to 58%; Table 2) and five as transitional zones (with unique species proportion ranging from 5 to 25%; Table 2). The identified areas were: (1) Taurus BR in southern Turkey; (2) the Anatolian–Armenian BR in eastern Turkey and in Armenia; (3) Alborz BR in northern Iran; (4) Zagros BR in western Iran; and (5) Yazd-Kerman BR in southern Iran (identified only by the total alpine species; Fig. 3, Table 2). Except one transitional zone in north-western Turkey (Uludağ), all transitional zones were located in an area from south-eastern Turkey to north-western Iran (Fig. 3). The list of species present in each Bioregion is provided in Tables S4 and S5.

**Table 2:** Bioregions (BR; numbers 1–5) and transitional zones (numbers 6–11; numbers as in Fig. 3) identified using Network-Clustering

No.	Name	Total alpine species					Endemic alpine species				
		No. of cells	(Sub)alpine area size	Total species	Unique species		No. of cells	(Sub)alpine area size	Total species	Unique species	
			(km^2^)	No.	No.	%		(km^2^)	No.	No.	%
1	Taurus BR	8	2542	300	94	31	7	2479	231	90	39
2	Anatolian–Armenian BR	25	42,019	910	467	51	17	24,583	429	145	34
3	Alborz BR	14	12,091	518	166	32	10	10,307	354	138	39
4	Zagros BR	19	10,131	446	161	36	19	9651	388	226	58
5	Yazd-Kerman BR	3	2220	161	50	31	–	–	–	–	–
6	Azerbaijan	7	–	326	26	8	6	–	216	21	10
7	Hakkari	–	–	–	–	–	5	–	392	97	25
8	–	2	–	162	6	4	2	–	116	6	5
9	–	1	–	36	5	14	1	–	34	5	15
10	–	1	–	68	7	10	–	–	–	–	–
11	–	–	–	–	–	–	1	–	48	7	15

ACDs inferred from the non-endemic alpine species differed substantially from ACDS and AEs identified with the other two datasets (the total alpine and the endemic alpine flora, respectively). With Endemicity Analysis, only the Alborz and the Anatolian–Armenian area were identified as ACDs, but both just covering part of the areas identified by endemics and total alpine species datasets (Figure S1). Using the Network-Clustering approach, the Iranian mountains and the Armenian mountains as well as several small areas in Anatolia were identified as bioregions (Figure S2).

## Discussion

The aim of this study is to identify major ACDs and AEs in the alpine zone of the Irano-Anatolian region, a global biodiversity hotspot (Mittermeier et al. 2005), using two methodological approaches, Endemicity Analysis and Network-Clustering. Reassuringly, the identified ACDs and AEs are largely congruent (Figs. 2, 3), discrepancies being mostly due to the inability of Network-Clustering to infer overlapping or even nested ACDs or AEs. Apart from the Taurus mountains in southern Turkey, where Endemicity Analysis infers two essentially nested ACDs or AEs (Fig. 2), the major differences concern south-eastern Turkey and north-western Iran. This region is located between the high mountains of Anatolia and Armenia on one side and Alborz and Zagros on the other side and constitutes a transitional zone combining elements of adjacent regions. Whereas Endemicity Analysis can accommodate this by inferring partially overlapping ACDs and AEs, Network-Clustering recognizes one to several additional bioregions (Figs. 2, 3). Additional information, such as the proportion of unique species, can help to address the nature of such transitional zones as overlapping ACDs or AEs. Still, bioregionalization using the Network-Clustering approach should be supplemented by methods that can directly address these transitions, such as Endemicity Analysis (Szumik et al. 2002) or Fuzzy Logics (Olivero et al. 2012).

Given the high degree of endemism in the alpine zone it can be expected that the delimitation of ACDs will be determined nearly exclusively by the endemic species, i.e., that AEs will be largely congruent with ACDs. This is actually the case for those areas identified by Endemicity Analysis, where the level of resolution (number and position of ACDs and AEs, respectively) is the same between the total alpine species datasets (used to infer ACDs) and the endemic alpine species (used to infer AEs), differences being mainly restricted to the scores of some cells (Table 1, Fig. 2). In line with this, the non-endemic alpine species just confirmed AEs already identified by the endemic alpine species and could do this only in the northern part of the study region, where the proportion of alpine species also occurring in mountain ranges outside the study area, most notably the Greater Caucasus, is higher (Noroozi et al. 2008). In contrast to Endemicity Analysis, the differences between datasets were bigger for Network-Clustering. Specifically, using the alpine endemics dataset resulted in decreased resolution (fewer bioregions) in western Anatolia (Uludağ merged with Taurus) and in south-eastern Iran (Yazd-Kerman merged with Zagros), but in increased resolution (more bioregions) in the Anatolian–Armenian area (separation of the Hakkari region in southeastern Turkey). A reason for this may be the higher sensitivity of Network-Clustering to quantitative features of the data, as was observed when comparing inferences from an unfiltered (duplicates retained) and a filtered dataset (duplicates removed; data not shown). Still, the non-endemic alpine species contribute little to delimiting bioregions. Jointly, these results demonstrate that in alpine habitats using fewer data, i.e., those from the endemic species only, can suffice to reliably identify congruent distribution patterns of the alpine flora.

Most of the AEs identified in this study, e.g., Taurus, Zagros or Alborz, have been identified before (Noroozi et al. 2018, 2019c). An exception is the Yazd-Kerman Massif, identified here as a separate unit, especially using Endemicity Analysis, in contrast to previous inferences based on the Iranian endemic species of Asteraceae (Noroozi et al. 2018). Despite the considerable surface area size of the Yazd-Kerman Massif, it harbours low species and endemic richness compared to Zagros and Alborz (Noroozi et al. 2019a; Doostmohammadi et al. 2020), which likely is due to the dry climate of the region (Djamali et al. 2011). However, the endemicity rate is high because of its topographic and climatic isolation, especially in the higher elevations (Djamali et al. 2011; Doostmohammadi et al. 2020).

Two AEs inferred in previous studies are not confirmed, because they are not found by Endemicity Analysis but are only identified as transitional zones by Network Clustering. The first area is the Hakkari area in south-eastern Turkey identified by Noroozi et al. (2019c) using data from three vascular plant families of Turkey. Compared to the other transitional zones, this region has a relatively high number of species and of unique species (Fig. 3, Table 2), but the proportion of unique species is below our threshold for recognizing an independent AE. Whereas this threshold is ad hoc, it allows separating areas (bioregions) supported also by Endemicity Analysis from those (transitional zones) not supported by Endemicity Analysis. Yet it is possible that additional analyses with more data may support recognition of the Hakkari area as an independent AE. The second area is the Azerbaijan Plateau in north-western Iran identified by Noroozi et al. (2018) based on Iranian endemic Asteraceae. Reasons for these discrepancies may include the different taxonomic coverage (wider in the current study) and the broader geographic coverage (both regions are close the edge of their respective study regions, Turkey and Iran).

## Conclusion

In general, the units identified using both approaches and both datasets (total alpine and endemic alpine species) are strongly congruent, suggesting that they reflect meaningful distribution patterns. As most of these areas have been identified before using the endemic flora of different elevational zones (Noroozi et al. 2018, 2019a, c), bioregionalization in the Irano-Anatolian hotspot appears to be strongly influenced by the alpine endemic species.

Identifying the major AEs (and ACDs) of the alpine flora inside the Irano-Anatolian biodiversity hotspot is a first step towards determining hotspots-within-hotspots (Cañadas et al. 2014). These AEs are not only areas with congruent distribution of alpine species, but are also regions with a high proportion of rare species at high elevations. Previous studies demonstrated that centres of endemism in the Irano-Anatolian biodiversity hotspot are poorly protected resulting in a high proportion of conservation gaps (Noroozi et al. 2019b) and that conservation efficiency in the already established nature reserves is lower than on the global average (Kolahi et al. 2013). As range restricted species of alpine habitats are highly threatened by ongoing climate change (Dullinger et al. 2012; Pauli et al. 2012) and by strong overgrazing (at least in the SW Asia; Kürschner and Parolly 2012; Kenar and Ketenoğlu 2016; Karimi and Jones 2020), putting more attention to the conservation status of these AEs is highly recommended.

## Supplementary Information

Below is the link to the electronic supplementary material.

Supplementary file1 (DOCX 1242 KB)
