# Supplementary material for: Endemics determine bioregionalization in the alpine zone of the Irano-Anatolian biodiversity hotspot (South-West Asia)
Source: Alp Bot. 2021 Aug 4;131(2):177–86. doi: 10.1007/s00035-021-00266-7 (PMC8549998; doi:10.1007/s00035-021-00266-7)

SUPPORTING INFORMATION

**Endemic determine bioregionalization in the alpine zone of the Irano-Anatolian biodiversity hotspot (South-West Asia)**

Jalil Noroozi, Sina Khalvati, Haniyeh Nafisi, Akram Kaveh, Behnaz Nazari, Masoud Minaei, Ernst Vitek, Gerald M. Schneeweiss

**Appendix S Supplementary Tables and Figures**

**Table S1** List of alpine species of the study area and their distribution as endemic and non-endemic, and number of recorded localities.

| **Species** | **Distribution** | Nr. of records |
| --- | --- | --- |
| Acantholimon albocalycinum | Endemic | 1 |
| Acantholimon artosense | Endemic | 1 |
| Acantholimon brachystachyum | Endemic | 8 |
| Acantholimon calvertii | Endemic | 10 |
| Acantholimon cupreo-olivascens | Endemic | 1 |
| Acantholimon demavendicum | Endemic | 6 |
| Acantholimon dianthifolium | Endemic | 4 |
| Acantholimon ekimii | Endemic | 1 |
| Acantholimon erinaceum | Non-Endemic | 36 |
| Acantholimon eschkerense | Endemic | 10 |
| Acantholimon haesarensis | Endemic | 1 |
| Acantholimon hohenackeri | Endemic | 30 |
| Acantholimon kermanense | Endemic | 5 |
| Acantholimon mirtadzadinii | Endemic | 1 |
| Acantholimon modestum | Endemic | 16 |
| Acantholimon nigricans | Endemic | 6 |
| Acantholimon oliganthum | Endemic | 4 |
| Acantholimon sahendicum | Endemic | 9 |
| Acantholimon scabrellum | Endemic | 9 |
| Acantholimon sirchense | Endemic | 3 |
| Acantholimon tomentellum | Endemic | 8 |
| Acantholimon ulicinum | Endemic | 7 |
| Acantholimon zaeifii | Endemic | 5 |
| Achillea armenorum | Endemic | 1 |
| Achillea aucheri | Endemic | 6 |
| Achillea kellalensis | Endemic | 1 |
| Achillea latiloba | Endemic | 4 |
| Achillea millefolium | Endemic | 35 |
| Achillea vermicularis | Endemic | 75 |
| Aconitum anthora | Endemic | 3 |
| Aconitum cochleare | Endemic | 6 |
| Aethionema caespitosum | Endemic | 8 |
| Aethionema fimbriatum | Endemic | 15 |
| Aethionema munzurense | Endemic | 2 |
| Aethionema oppositifolium | Endemic | 8 |
| Aethionema papillosum | Endemic | 2 |
| Aethionema rotundifolium | Non-Endemic | 1 |
| Aethionema semnanensis | Endemic | 4 |
| Aethionema speciosum | Endemic | 8 |
| Aethionema stenopterum | Endemic | 7 |
| Aethionema subulatum | Endemic | 4 |
| Aethionema trinervium | Non-Endemic | 46 |
| Aethionema umbellatum | Endemic | 8 |
| Aethionema virgatum | Endemic | 8 |
| Agropyron canaliculatum | Non-Endemic | 1 |
| Agropyron cognatum | Non-Endemic | 2 |
| Agropyron imbricatum | Non-Endemic | 29 |
| Agrostis canina | Non-Endemic | 1 |
| Agrostis lazica | Non-Endemic | 6 |
| Agrostis olympica | Non-Endemic | 15 |
| Agrostis vinealis | Non-Endemic | 13 |
| Alchemilla amardica | Endemic | 2 |
| Alchemilla basakii | Endemic | 1 |
| Alchemilla caucasica | Non-Endemic | 16 |
| Alchemilla ciminensis | Endemic | 2 |
| Alchemilla citrina | Endemic | 8 |
| Alchemilla compactilis | Endemic | 17 |
| Alchemilla dura | Non-Endemic | 6 |
| Alchemilla ellenbergiana | Non-Endemic | 10 |
| Alchemilla erythropoda | Non-Endemic | 23 |
| Alchemilla erzincanensis | Endemic | 2 |
| Alchemilla farinosa | Endemic | 4 |
| Alchemilla fluminea | Endemic | 4 |
| Alchemilla gigantodus | Endemic | 3 |
| Alchemilla grossheimii | Non-Endemic | 12 |
| Alchemilla hemsinica | Endemic | 2 |
| Alchemilla hessii | Endemic | 6 |
| Alchemilla heterophylla | Non-Endemic | 5 |
| Alchemilla kackarensis | Endemic | 1 |
| Alchemilla kurdica | Endemic | 4 |
| Alchemilla mazandarana | Endemic | 1 |
| Alchemilla melancholica | Endemic | 5 |
| Alchemilla microscopica | Endemic | 3 |
| Alchemilla minusculiflora | Non-Endemic | 6 |
| Alchemilla oligotricha | Non-Endemic | 4 |
| Alchemilla oriturcica | Endemic | 4 |
| Alchemilla ovitensis | Endemic | 1 |
| Alchemilla paracompactilis | Endemic | 1 |
| Alchemilla pectiniloba | Endemic | 3 |
| Alchemilla persica | Endemic | 42 |
| Alchemilla plicatissima | Endemic | 3 |
| Alchemilla plicatula | Non-Endemic | 2 |
| Alchemilla procerrima | Endemic | 2 |
| Alchemilla pseudocartalinica | Non-Endemic | 22 |
| Alchemilla raddeana | Non-Endemic | 1 |
| Alchemilla retinervis | Non-Endemic | 18 |
| Alchemilla rivularis | Endemic | 1 |
| Alchemilla rizensis | Endemic | 5 |
| Alchemilla sedelmeyeriana | Non-Endemic | 8 |
| Alchemilla sericata | Non-Endemic | 13 |
| Alchemilla sericea | Non-Endemic | 21 |
| Alchemilla straminea | Non-Endemic | 2 |
| Alchemilla surculosa | Endemic | 6 |
| Alchemilla tiryalensis | Endemic | 3 |
| Alchemilla venosa | Non-Endemic | 4 |
| Alkanna bracteosa | Endemic | 26 |
| Alkanna frigida | Endemic | 26 |
| Allium akaka | Endemic | 31 |
| Allium alamutense | Endemic | 7 |
| Allium alpinarii | Endemic | 1 |
| Allium anacoleum | Endemic | 5 |
| Allium arlgirdense | Endemic | 2 |
| Allium aucheri | Endemic | 9 |
| Allium austroiranicum | Endemic | 80 |
| Allium balansae | Endemic | 4 |
| Allium brachyodon | Endemic | 3 |
| Allium breviscapum | Endemic | 16 |
| Allium capitellatum | Endemic | 8 |
| Allium cathodicarpum | Endemic | 31 |
| Allium derderianum | Endemic | 108 |
| Allium djimilense | Endemic | 6 |
| Allium donmezii | Endemic | 1 |
| Allium dumanii | Endemic | 1 |
| Allium egorovae | Endemic | 2 |
| Allium elburzense | Endemic | 53 |
| Allium flavum | Endemic | 2 |
| Allium hoshabicum | Endemic | 1 |
| Allium hymenorhizum | Endemic | 2 |
| Allium iranshahrii | Endemic | 6 |
| Allium kuhrangense | Endemic | 1 |
| Allium kunthianum | Non-Endemic | 14 |
| Allium lalesaricum | Endemic | 3 |
| Allium longivaginatum | Endemic | 8 |
| Allium mahneshanense | Endemic | 4 |
| Allium microspathum | Endemic | 1 |
| Allium montelburzense | Endemic | 1 |
| Allium oreophilum | Non-Endemic | 1 |
| Allium pseudoampeloprasum | Endemic | 2 |
| Allium pseudostrictum | Non-Endemic | 7 |
| Allium rhetoreanum | Endemic | 1 |
| Allium sabalense | Endemic | 5 |
| Allium sahandicum | Endemic | 12 |
| Allium schoenoprasum | Non-Endemic | 19 |
| Allium scotostemon | Endemic | 41 |
| Allium shatakiense | Endemic | 6 |
| Allium stearnianum | Endemic | 4 |
| Allium straussii | Endemic | 4 |
| Allium szovitsii | Endemic | 10 |
| Allium talyschense | Endemic | 1 |
| Allium tauricola | Endemic | 11 |
| Allium tuchalense | Endemic | 67 |
| Allium yamadagensis | Endemic | 1 |
| Alopecurus apiatus | Non-Endemic | 3 |
| Alopecurus aucheri | Endemic | 18 |
| Alopecurus dasyanthus | Non-Endemic | 7 |
| Alopecurus glacialis | Non-Endemic | 4 |
| Alopecurus himalaicus | Non-Endemic | 1 |
| Alopecurus laguroides | Endemic | 6 |
| Alopecurus lanatus | Endemic | 6 |
| Alopecurus mucronatus | Non-Endemic | 1 |
| Alopecurus seravschanicus | Non-Endemic | 2 |
| Alopecurus textilis | Endemic | 36 |
| Alopecurus vaginatus | Non-Endemic | 23 |
| Alyssopsis mollis | Endemic | 13 |
| Alyssum aizoides | Endemic | 5 |
| Alyssum armenum | Endemic | 7 |
| Alyssum aurantiacum | Endemic | 2 |
| Alyssum gehamense | Endemic | 10 |
| Alyssum haussknechtii | Endemic | 3 |
| Alyssum lanceolatum | Non-Endemic | 12 |
| Alyssum muelleri | Endemic | 11 |
| Alyssum peltarioides | Endemic | 7 |
| Alyssum persicum | Endemic | 3 |
| Alyssum polycladum | Endemic | 9 |
| Alyssum propinquum | Endemic | 3 |
| Amygdalus carduchorum | Endemic | 4 |
| Amygdalus elaeagnifolia | Endemic | 28 |
| Anchonium elichrysifolium | Endemic | 55 |
| Androsace albana | Non-Endemic | 9 |
| Androsace armeniaca | Endemic | 23 |
| Androsace caduca | Non-Endemic | 4 |
| Androsace chamaejasme | Non-Endemic | 4 |
| Androsace intermedia | Non-Endemic | 8 |
| Androsace multiscapa | Endemic | 4 |
| Androsace villosa | Non-Endemic | 48 |
| Anemone fasciculata | Non-Endemic | 8 |
| Antennaria dioica | Non-Endemic | 4 |
| Anthemis marschalliana | Non-Endemic | 4 |
| Anthriscus kotschyi | Non-Endemic | 5 |
| Arabis androsacea | Endemic | 3 |
| Arabis brachycarpa | Endemic | 10 |
| Arabis carduchorum | Endemic | 35 |
| Arabis caucasica | Endemic | 94 |
| Arabis graellsiiformis | Endemic | 2 |
| Arabis lycia | Endemic | 2 |
| Arabis rimarum | Endemic | 3 |
| Arenaria angustifolioides | Endemic | 1 |
| Arenaria angustisepala | Endemic | 1 |
| Arenaria antitaurica | Endemic | 3 |
| Arenaria balansae | Endemic | 15 |
| Arenaria blepharophylla | Endemic | 4 |
| Arenaria bulica | Endemic | 2 |
| Arenaria cucubaloides | Endemic | 22 |
| Arenaria davisii | Endemic | 1 |
| Arenaria dianthoides | Endemic | 27 |
| Arenaria gypsophiloides | Endemic | 104 |
| Arenaria insignis | Non-Endemic | 24 |
| Arenaria ledebouriana | Endemic | 6 |
| Arenaria lychnidea | Non-Endemic | 2 |
| Arenaria minutissima | Endemic | 3 |
| Arenaria mons-cragus | Endemic | 1 |
| Arenaria persica | Endemic | 18 |
| Arenaria rotundifolia | Non-Endemic | 27 |
| Arenaria semiromica | Endemic | 1 |
| Arnebia euchroma | Non-Endemic | 3 |
| Arnebia pulchra | Endemic | 13 |
| Artemisia aucheri | Non-Endemic | 54 |
| Artemisia biennis | Non-Endemic | 15 |
| Artemisia chamaemelifolia | Non-Endemic | 22 |
| Artemisia haussknechtii | Endemic | 22 |
| Artemisia lehmanniana | Non-Endemic | 1 |
| Artemisia melanolepis | Endemic | 11 |
| Artemisia persica | Non-Endemic | 11 |
| Artemisia splendens | Endemic | 49 |
| Asperula affinis | Endemic | 8 |
| Asperula capitellata | Endemic | 4 |
| Asperula fragillima | Endemic | 5 |
| Asperula glomerata | Non-Endemic | 126 |
| Asperula laxiflora | Endemic | 24 |
| Asperula lycia | Non-Endemic | 3 |
| Asperula nitida | Non-Endemic | 8 |
| Asperula pontica | Non-Endemic | 6 |
| Asperula prostrata | Endemic | 24 |
| Asperula rechingeri | Endemic | 12 |
| Asplenium tadei | Endemic | 1 |
| Asplenium viride | Non-Endemic | 1 |
| Aster alpinus | Non-Endemic | 23 |
| Astragalus abditus | Endemic | 5 |
| Astragalus acmophyllus | Endemic | 15 |
| Astragalus aegobromus | Endemic | 40 |
| Astragalus aestivorum | Endemic | 2 |
| Astragalus agassii | Endemic | 2 |
| Astragalus aladagensis | Endemic | 3 |
| Astragalus alamkuhensis | Endemic | 1 |
| Astragalus alpinus | Non-Endemic | 3 |
| Astragalus alyssoides | Endemic | 70 |
| Astragalus argaeus | Endemic | 6 |
| Astragalus atricapillus | Endemic | 3 |
| Astragalus aureus | Endemic | 209 |
| Astragalus azizii | Endemic | 1 |
| Astragalus bahcesarayensis | Endemic | 2 |
| Astragalus barnassari | Endemic | 25 |
| Astragalus bashkalensis | Endemic | 1 |
| Astragalus beckerianus | Non-Endemic | 1 |
| Astragalus beckii | Endemic | 23 |
| Astragalus bounophilus | Endemic | 9 |
| Astragalus brachycalyx | Endemic | 101 |
| Astragalus capax | Endemic | 13 |
| Astragalus capito | Endemic | 14 |
| Astragalus carmanicus | Endemic | 1 |
| Astragalus cataonicus | Endemic | 4 |
| Astragalus chartostegius | Endemic | 7 |
| Astragalus chionobiiformis | Endemic | 3 |
| Astragalus chrysanthus | Endemic | 22 |
| Astragalus ciloensis | Endemic | 1 |
| Astragalus confusus | Endemic | 4 |
| Astragalus czorochensis | Endemic | 4 |
| Astragalus daenaensis | Endemic | 10 |
| Astragalus dasycarpus | Endemic | 3 |
| Astragalus declinatus | Endemic | 16 |
| Astragalus demavendicus | Endemic | 7 |
| Astragalus dieterlei | Endemic | 1 |
| Astragalus dumanii | Endemic | 3 |
| Astragalus dzebrailicus | Endemic | 5 |
| Astragalus eriocalyx | Endemic | 1 |
| Astragalus eriocephalus | Endemic | 18 |
| Astragalus erivanensis | Endemic | 6 |
| Astragalus ermineus | Endemic | 13 |
| Astragalus euoplus | Endemic | 4 |
| Astragalus exspectatus | Endemic | 2 |
| Astragalus fragiferus | Endemic | 14 |
| Astragalus fragrans | Endemic | 74 |
| Astragalus fraxinifolius | Endemic | 26 |
| Astragalus frickii | Endemic | 13 |
| Astragalus gevashensis | Endemic | 4 |
| Astragalus geyikdaghensis | Endemic | 1 |
| Astragalus gezeldarensis | Endemic | 8 |
| Astragalus ghashghaicus | Endemic | 2 |
| Astragalus globosus | Endemic | 38 |
| Astragalus griseus | Endemic | 7 |
| Astragalus hareftae | Endemic | 2 |
| Astragalus hartvigii | Endemic | 2 |
| Astragalus hausknechtii | Endemic | 12 |
| Astragalus herbertii | Endemic | 1 |
| Astragalus heterodoxus | Endemic | 3 |
| Astragalus heterozyx | Endemic | 1 |
| Astragalus hezarensis | Endemic | 2 |
| Astragalus hirticalyx | Endemic | 23 |
| Astragalus horasanicus | Endemic | 3 |
| Astragalus horridus | Endemic | 9 |
| Astragalus humilis | Endemic | 2 |
| Astragalus hyalolepis | Endemic | 23 |
| Astragalus icmadophilus | Endemic | 34 |
| Astragalus incertus | Endemic | 49 |
| Astragalus inexpectatus | Endemic | 2 |
| Astragalus issatissensis | Endemic | 1 |
| Astragalus jodotropis | Endemic | 99 |
| Astragalus johannis | Endemic | 16 |
| Astragalus karabaghensis | Endemic | 16 |
| Astragalus lalesarensis | Endemic | 1 |
| Astragalus lanatus | Endemic | 2 |
| Astragalus laricus | Endemic | 2 |
| Astragalus latistipulatus | Endemic | 1 |
| Astragalus latus | Endemic | 4 |
| Astragalus leiophyllus | Endemic | 19 |
| Astragalus lineatus | Endemic | 188 |
| Astragalus lunatus | Non-Endemic | 1 |
| Astragalus lycioides | Endemic | 28 |
| Astragalus macrosemius | Endemic | 92 |
| Astragalus macrourus | Endemic | 41 |
| Astragalus mahneshanensis | Endemic | 1 |
| Astragalus melanocalyx | Endemic | 2 |
| Astragalus melanocarpus | Endemic | 3 |
| Astragalus melanodon | Endemic | 3 |
| Astragalus modestus | Endemic | 5 |
| Astragalus monanthemus | Endemic | 4 |
| Astragalus montis-alamkuhi | Endemic | 1 |
| Astragalus montis-parrowii | Endemic | 1 |
| Astragalus montis-varvashti | Endemic | 1 |
| Astragalus murinus | Endemic | 30 |
| Astragalus nabelekii | Endemic | 1 |
| Astragalus nezaketiae | Endemic | 2 |
| Astragalus nezva-montis | Endemic | 2 |
| Astragalus nigropedunculatus | Endemic | 2 |
| Astragalus ochrochlorus | Endemic | 39 |
| Astragalus oreades | Non-Endemic | 12 |
| Astragalus ovigerus | Endemic | 1 |
| Astragalus pascuicola | Endemic | 1 |
| Astragalus patrius | Endemic | 12 |
| Astragalus pauperiflorus | Endemic | 38 |
| Astragalus pelliger | Endemic | 16 |
| Astragalus pennatus | Endemic | 5 |
| Astragalus perdurans | Endemic | 6 |
| Astragalus perrarus | Endemic | 3 |
| Astragalus pinetorum | Endemic | 133 |
| Astragalus plagiophacos | Endemic | 3 |
| Astragalus platysematus | Endemic | 7 |
| Astragalus pluriflorus | Endemic | 1 |
| Astragalus podosphaerus | Endemic | 1 |
| Astragalus polyanthus | Endemic | 9 |
| Astragalus polygala | Endemic | 8 |
| Astragalus pseudofragrans | Endemic | 2 |
| Astragalus pseudojohannis | Endemic | 1 |
| Astragalus pseudopinetorum | Endemic | 1 |
| Astragalus pseudoshebarensis | Endemic | 7 |
| Astragalus rechingeri | Endemic | 4 |
| Astragalus remotiflorus | Endemic | 25 |
| Astragalus robertianus | Endemic | 1 |
| Astragalus rubriflorus | Endemic | 31 |
| Astragalus rubrolineatus | Endemic | 2 |
| Astragalus rudimentus | Endemic | 3 |
| Astragalus rufescens | Endemic | 5 |
| Astragalus sachanewii | Endemic | 11 |
| Astragalus sahendi | Endemic | 35 |
| Astragalus savellanicus | Endemic | 2 |
| Astragalus shahsavaranicus | Endemic | 1 |
| Astragalus sphaeranthus | Endemic | 15 |
| Astragalus stenosemioides | Endemic | 4 |
| Astragalus stenostegius | Endemic | 6 |
| Astragalus stridii | Endemic | 6 |
| Astragalus subhanensis | Endemic | 1 |
| Astragalus subsecundus | Endemic | 24 |
| Astragalus taleshensis | Endemic | 1 |
| Astragalus tauricolus | Endemic | 10 |
| Astragalus tenuiscapus | Endemic | 2 |
| Astragalus trabzonicus | Endemic | 1 |
| Astragalus turgidus | Endemic | 1 |
| Astragalus uraniolimneus | Endemic | 25 |
| Astragalus vavilovii | Endemic | 6 |
| Astragalus xerophilus | Endemic | 35 |
| Astragalus zagrosicus | Endemic | 7 |
| Astragalus zerdanus | Endemic | 8 |
| Astragalus zohrabi | Endemic | 20 |
| Asyneuma ekimianum | Endemic | 3 |
| Asyneuma filipes | Endemic | 9 |
| Asyneuma multicaule | Endemic | 7 |
| Asyneuma persicum | Endemic | 25 |
| Asyneuma pulchellum | Endemic | 3 |
| Asyneuma rigidum | Endemic | 10 |
| Athyrium distentifolium | Endemic | 3 |
| Aubrieta anamasica | Endemic | 1 |
| Aurinia calycocarpum | Non-Endemic | 1 |
| Aurinia rupestris | Endemic | 7 |
| Barbamine procumbens | Endemic | 3 |
| Barbarea minor | Endemic | 10 |
| Bellardiochloa argaea | Endemic | 4 |
| Bellardiochloa carica | Endemic | 1 |
| Bellardiochloa polychroa | Endemic | 12 |
| Bellevalia paradoxa | Endemic | 37 |
| Bellevalia rixii | Endemic | 3 |
| Bellevalia tristis | Endemic | 5 |
| Betonica nivea | Endemic | 8 |
| Bornmuellera cappadocica | Endemic | 3 |
| Brachyactis roylei | Non-Endemic | 2 |
| Briza marcowiczii | Non-Endemic | 2 |
| Bromus armenus | Endemic | 3 |
| Bromus biebersteinii | Non-Endemic | 1 |
| Bromus cappadocicus | Endemic | 7 |
| Bromus confinis | Endemic | 2 |
| Bromus frigidus | Endemic | 3 |
| Bromus gracillimus | Non-Endemic | 13 |
| Bromus stenostachyus | Non-Endemic | 2 |
| Bromus tomentosus | Non-Endemic | 20 |
| Bromus variegatus | Non-Endemic | 27 |
| Bufonia koelzii | Endemic | 16 |
| Bufonia kotschyana | Endemic | 51 |
| Bufonia micrantha | Endemic | 1 |
| Bufonia stapfii | Endemic | 1 |
| Bunium brachyactis | Endemic | 3 |
| Bupleurum falcatum | Endemic | 23 |
| Calamagrostis decora | Non-Endemic | 1 |
| Calamagrostis parsana | Endemic | 3 |
| Calamintha caroli-henricana | Endemic | 2 |
| Campanula armena | Endemic | 7 |
| Campanula bayerniana | Endemic | 6 |
| Campanula bornmuelleri | Endemic | 3 |
| Campanula choruhensis | Endemic | 2 |
| Campanula collina | Endemic | 7 |
| Campanula conferta | Endemic | 7 |
| Campanula gilliatii | Endemic | 1 |
| Campanula hedgei | Endemic | 3 |
| Campanula hermannii | Endemic | 1 |
| Campanula humillima | Endemic | 8 |
| Campanula karakuschensis | Endemic | 7 |
| Campanula lourica | Endemic | 12 |
| Campanula luristanica | Endemic | 5 |
| Campanula pulvinaris | Endemic | 1 |
| Campanula saxifraga | Endemic | 26 |
| Campanula stevenii | Endemic | 76 |
| Campanula telephioides | Endemic | 3 |
| Campanula tridentata | Non-Endemic | 18 |
| Campanula zangezura | Endemic | 2 |
| Carduus lanuginosus | Endemic | 8 |
| Carex atherodes | Non-Endemic | 1 |
| Carex atrata | Non-Endemic | 19 |
| Carex brevicollis | Non-Endemic | 9 |
| Carex capitellata | Endemic | 13 |
| Carex caucasica | Non-Endemic | 10 |
| Carex decaulescens | Non-Endemic | 1 |
| Carex disticha | Non-Endemic | 4 |
| Carex flacca | Non-Endemic | 1 |
| Carex magellanica | Non-Endemic | 4 |
| Carex medwedewii | Endemic | 14 |
| Carex melanantha | Non-Endemic | 1 |
| Carex melanorrhyncha | Endemic | 1 |
| Carex michelii | Non-Endemic | 3 |
| Carex microglochin | Non-Endemic | 2 |
| Carex oligantha | Non-Endemic | 4 |
| Carex orbicularis | Non-Endemic | 54 |
| Carex oreophila | Endemic | 17 |
| Carex ornithopoda | Endemic | 1 |
| Carex pauescens | Non-Endemic | 1 |
| Carex pontica | Non-Endemic | 3 |
| Carex pseudofoetida | Non-Endemic | 11 |
| Carex pyrenaica | Non-Endemic | 5 |
| Carex stenophylla | Non-Endemic | 19 |
| Carex tomentosa | Endemic | 1 |
| Carex transcaucasica | Non-Endemic | 2 |
| Carex tristis | Endemic | 16 |
| Carex umbrosa | Non-Endemic | 8 |
| Carum carvi | Non-Endemic | 26 |
| Carum caucasicum | Endemic | 21 |
| Carum komarovii | Endemic | 2 |
| Carum meifolium | Non-Endemic | 10 |
| Carum rupicola | Endemic | 2 |
| Catabrosa aquatica | Endemic | 16 |
| Centaurea appendicigera | Endemic | 6 |
| Centaurea armena | Endemic | 3 |
| Centaurea cheiranthifolia | Non-Endemic | 15 |
| Centaurea congesta | Endemic | 5 |
| Centaurea drabifolia | Endemic | 38 |
| Centaurea elbrusensis | Endemic | 4 |
| Centaurea incanescens | Endemic | 5 |
| Centaurea karduchorum | Endemic | 4 |
| Centaurea lanigera | Endemic | 7 |
| Centaurea macrocephala | Non-Endemic | 3 |
| Centaurea mucronifera | Endemic | 14 |
| Centaurea nigrofimbria | Endemic | 4 |
| Centaurea pichleri | Endemic | 3 |
| Centaurea poluninii | Endemic | 1 |
| Centaurea pulcherrima | Endemic | 10 |
| Centaurea rhizantha | Endemic | 42 |
| Centaurea schelkovnikovii | Endemic | 1 |
| Centaurea sieheana | Endemic | 1 |
| Cephalaria cilodaghensis | Endemic | 1 |
| Cephalaria gigantea | Non-Endemic | 5 |
| Cephalaria kleinii | Endemic | 1 |
| Cephalaria microcephala | Endemic | 75 |
| Cephalaria scoparia | Endemic | 1 |
| Cephalaria sparsipilosa | Endemic | 2 |
| Cerastium araraticum | Endemic | 3 |
| Cerastium cerastoides | Non-Endemic | 46 |
| Cerastium davuricum | Non-Endemic | 6 |
| Cerastium gnaphalodes | Endemic | 12 |
| Cerastium lazicum | Endemic | 1 |
| Cerastium persicum | Endemic | 3 |
| Cerastium pseudokasbek | Endemic | 2 |
| Cerastium purpurascens | Endemic | 30 |
| Cerastium szowitsii | Endemic | 9 |
| Cerasus brachypetala | Endemic | 51 |
| Cerinthe glabra | Non-Endemic | 7 |
| Chaenorhinum grossecostatum | Endemic | 4 |
| Chaerophyllum astrantiae | Non-Endemic | 4 |
| Chaerophyllum hakkiaricum | Endemic | 2 |
| Chaerophyllum Icucolaenum | Endemic | 1 |
| Chaerophyllum khorossanicum | Endemic | 17 |
| Chaerophyllum macrospermum | Endemic | 45 |
| Chaerophyllum nivale | Endemic | 5 |
| Chamaegeron asterellus | Endemic | 8 |
| Chamaesciadium acaule | Endemic | 25 |
| Chenopodium foliosum | Non-Endemic | 122 |
| Cicer anatolicum | Endemic | 51 |
| Cicer incisum | Endemic | 10 |
| Cicer stapfianum | Endemic | 1 |
| Cicer tragacanthoides | Endemic | 68 |
| Cicerbita adenophora | Endemic | 3 |
| Cirsium aggregatum | Non-Endemic | 2 |
| Cirsium ellenbergii | Endemic | 1 |
| Cirsium kosmelii | Endemic | 3 |
| Cirsium lappaceum | Endemic | 25 |
| Cirsium mimitum | Non-Endemic | 2 |
| Cirsium obvallatum | Non-Endemic | 8 |
| Cirsium peshmenianum | Endemic | 1 |
| Cirsium pseudobracteosum | Endemic | 2 |
| Cirsium rhizocephalum | Endemic | 20 |
| Cirsium simplex | Endemic | 5 |
| Cirsium tomentosum | Endemic | 4 |
| Clastopus erubescens | Endemic | 3 |
| Clastopus vestitus | Endemic | 6 |
| Cochlearia aucheri | Endemic | 8 |
| Cochlearia sintenisii | Endemic | 6 |
| Colchicum kurdicum | Endemic | 4 |
| Colpodium araraticum | Endemic | 9 |
| Colpodium fibrosum | Endemic | 6 |
| Colpodium gillettii | Endemic | 3 |
| Colpodium parviflorum | Endemic | 17 |
| Colpodium variegatum | Non-Endemic | 17 |
| Colpodium versicolor | Endemic | 14 |
| Colpodium violaceum | Endemic | 9 |
| Coluteocarpus vesicaria | Endemic | 28 |
| Coronilla orientalis | Non-Endemic | 5 |
| Cortusa matthioli | Endemic | 2 |
| Corydalis alpestris | Non-Endemic | 6 |
| Corydalis conorhiza | Non-Endemic | 2 |
| Corydalis persica | Endemic | 3 |
| Cotoneaster persicus | Endemic | 16 |
| Cotoneaster zangezuricus | Endemic | 1 |
| Cousinia adenosticta | Endemic | 88 |
| Cousinia archibaldii | Endemic | 3 |
| Cousinia bachtiarica | Endemic | 4 |
| Cousinia bornmuelleri | Endemic | 9 |
| Cousinia concinna | Endemic | 1 |
| Cousinia crispa | Endemic | 142 |
| Cousinia decumbens | Endemic | 1 |
| Cousinia eburnea | Endemic | 1 |
| Cousinia elwendensis | Endemic | 2 |
| Cousinia fragilis | Endemic | 2 |
| Cousinia gmelini | Endemic | 4 |
| Cousinia harazensis | Endemic | 4 |
| Cousinia irritans | Endemic | 5 |
| Cousinia karkasensis | Endemic | 2 |
| Cousinia lasiolepis | Non-Endemic | 10 |
| Cousinia longifolia | Endemic | 10 |
| Cousinia multiloba | Non-Endemic | 24 |
| Cousinia ottonis | Endemic | 1 |
| Cousinia pterocaulos | Endemic | 5 |
| Cousinia satdagensis | Endemic | 2 |
| Cousinia shahvarica | Endemic | 6 |
| Cousinia sicigera | Endemic | 3 |
| Cousinia xiphiolepis | Endemic | 3 |
| Crepis armena | Endemic | 7 |
| Crepis asadbarensis | Endemic | 14 |
| Crepis bupleurifolia | Endemic | 5 |
| Crepis connexa | Endemic | 1 |
| Crepis conyzifolia | Endemic | 3 |
| Crepis demavendi | Endemic | 5 |
| Crepis dioritica | Endemic | 3 |
| Crepis elbursensis | Endemic | 10 |
| Crepis flexuosa | Non-Endemic | 2 |
| Crepis frigida | Endemic | 8 |
| Crepis heterotricha | Endemic | 26 |
| Crepis multicaulis | Non-Endemic | 6 |
| Crepis pannonica | Non-Endemic | 2 |
| Crepis sahendi | Endemic | 33 |
| Crepis willdenowii | Endemic | 11 |
| Crocus abracteolus | Endemic | 1 |
| Crocus kotschyanus | Endemic | 10 |
| Crocus scharojanii | Non-Endemic | 4 |
| Crocus vallicola | Non-Endemic | 6 |
| Cyclotrichium straussii | Endemic | 1 |
| Cymbocarpum erythraeum | Endemic | 2 |
| Cynoglossum holosericeum | Non-Endemic | 1 |
| Daphne glomerata | Non-Endemic | 12 |
| Daphne magakjanii | Endemic | 3 |
| Daphne oleoides | Endemic | 12 |
| Delphinium carduchorum | Endemic | 3 |
| Delphinium elbursense | Endemic | 15 |
| Delphinium flexuosum | Non-Endemic | 20 |
| Delphinium foetidum | Endemic | 1 |
| Delphinium lalesaricum | Endemic | 1 |
| Delphinium lanigerum | Endemic | 7 |
| Delphinium linearilobum | Endemic | 9 |
| Delphinium uncinatum | Non-Endemic | 1 |
| Deschampsia cespitosa | Non-Endemic | 36 |
| Dianthus balansae | Endemic | 7 |
| Dianthus brevicaulis | Endemic | 8 |
| Dianthus cretaceus | Endemic | 24 |
| Dianthus denaicus | Endemic | 1 |
| Dianthus diversifolius | Endemic | 1 |
| Dianthus elymaiticus | Endemic | 1 |
| Dianthus erythrocoleus | Endemic | 9 |
| Dianthus goerkii | Endemic | 2 |
| Dianthus lactiflorus | Endemic | 2 |
| Dianthus leucophaeus | Endemic | 1 |
| Dianthus libanotis | Endemic | 29 |
| Dianthus micranthus | Endemic | 22 |
| Dianthus multicaulis | Endemic | 5 |
| Dianthus muschianus | Endemic | 3 |
| Dianthus orientalis | Non-Endemic | 62 |
| Dianthus raddeanus | Endemic | 5 |
| Dianthus recognitus | Endemic | 2 |
| Dianthus sahandicus | Endemic | 1 |
| Dianthus seidlitzii | Endemic | 15 |
| Dianthus sessiliflorus | Endemic | 1 |
| Dianthus vanensis | Endemic | 1 |
| Dichodon alborzensis | Endemic | 1 |
| Didymophysa aucheri | Endemic | 58 |
| Dielsiocharis kotschyi | Endemic | 18 |
| Dionysia archibaldii | Endemic | 3 |
| Dionysia assadii | Endemic | 1 |
| Dionysia aubrietioides | Endemic | 1 |
| Dionysia caespitosa | Endemic | 11 |
| Dionysia cristagalli | Endemic | 2 |
| Dionysia curviflora | Endemic | 3 |
| Dionysia esfandiarii | Endemic | 1 |
| Dionysia iranshahrii | Endemic | 2 |
| Dionysia khatamii | Endemic | 1 |
| Dionysia khuzistanica | Endemic | 1 |
| Dionysia leucotricha | Endemic | 13 |
| Dionysia oreodoxa | Endemic | 3 |
| Dionysia revoluta | Endemic | 31 |
| Dionysia rhaptodes | Endemic | 4 |
| Dionysia termeana | Endemic | 4 |
| Dionysia zagrica | Endemic | 1 |
| Dionysia zetterlundii | Endemic | 2 |
| Dionysia zschummelii | Endemic | 1 |
| Diplotaenia cachrydifolia | Endemic | 29 |
| Diplotaenia damavandica | Endemic | 12 |
| Dolichorrhiza persica | Endemic | 2 |
| Dorema aucheri | Endemic | 24 |
| Doronicum bracteatum | Endemic | 4 |
| Doronicum dolichotrichum | Endemic | 3 |
| Doronicum hakkiaricum | Endemic | 2 |
| Doronicum macrophyllum | Non-Endemic | 22 |
| Doronicum maximum | Endemic | 6 |
| Doronicum oblongifolium | Non-Endemic | 10 |
| Doronicum tobeyi | Endemic | 1 |
| Draba araratica | Endemic | 5 |
| Draba bruniifolia | Endemic | 47 |
| Draba cappadocica | Endemic | 4 |
| Draba nemorosa | Non-Endemic | 16 |
| Draba orientalis | Endemic | 1 |
| Draba polytricha | Endemic | 8 |
| Draba pulchella | Endemic | 107 |
| Draba rosularis | Non-Endemic | 15 |
| Draba siliquosa | Non-Endemic | 8 |
| Draba thylacocarpa | Endemic | 2 |
| Dracocephalum aucheri | Endemic | 62 |
| Dracocephalum botryoides | Non-Endemic | 1 |
| Dracocephalum ghahremanii | Endemic | 1 |
| Dracocephalum kotschyi | Endemic | 67 |
| Dracocephalum multicaule | Endemic | 14 |
| Dracocephalum polychaetum | Endemic | 5 |
| Dracocephalum surmandinum | Endemic | 1 |
| Drymocallis damghanensis | Endemic | 1 |
| Echinophora cinerea | Endemic | 15 |
| Elburzia fenestrata | Endemic | 4 |
| Eleutherospermum cicutarium | Non-Endemic | 11 |
| Elymus longiaristatus | Non-Endemic | 26 |
| Elymus transhyrcanus | Non-Endemic | 3 |
| Empetrum nigrum | Non-Endemic | 7 |
| Epilobium algidum | Non-Endemic | 9 |
| Epilobium anagallidifolium | Non-Endemic | 10 |
| Epilobium frigidum | Endemic | 25 |
| Epilobium palustre | Non-Endemic | 1 |
| Epilobium ponticum | Endemic | 22 |
| Epilobium rechingeri | Endemic | 7 |
| Eremopoa bellula | Non-Endemic | 1 |
| Eremopoa songarica | Non-Endemic | 1 |
| Eremurus persicus | Non-Endemic | 29 |
| Erigeron acris | Non-Endemic | 89 |
| Erigeron caucasicus | Endemic | 46 |
| Erigeron cilicicus | Endemic | 5 |
| Erigeron daenensis | Endemic | 1 |
| Erigeron hyrcanicus | Endemic | 14 |
| Erigeron uniflorus | Non-Endemic | 46 |
| Erigeron zederbaueri | Endemic | 2 |
| Eritrichium gracillimum | Endemic | 1 |
| Erodium absinthoides | Non-Endemic | 18 |
| Erodium cedrorum | Endemic | 3 |
| Erodium dimorphum | Endemic | 2 |
| Erodium hakkiaricum | Endemic | 1 |
| Eryngium bornmuelleri | Endemic | 2 |
| Eryngium ilex | Endemic | 1 |
| Erysimum caespitosum | Endemic | 106 |
| Erysimum damirliense | Endemic | 1 |
| Erysimum elbrusense | Endemic | 89 |
| Erysimum frigidum | Endemic | 2 |
| Erysimum gelidum | Endemic | 59 |
| Erysimum guneri | Endemic | 1 |
| Erysimum hakkiaricum | Endemic | 10 |
| Erysimum hezarense | Endemic | 2 |
| Erysimum ikizdereense | Endemic | 1 |
| Erysimum kotschyanum | Endemic | 25 |
| Erysimum macrostigma | Endemic | 20 |
| Erysimum munzuriense | Endemic | 3 |
| Erysimum nasturtioides | Endemic | 5 |
| Erysimum polatschekii | Endemic | 2 |
| Erysimum rizeense | Endemic | 1 |
| Erysimum sintenisianum | Endemic | 15 |
| Erysimum yildirimlii | Endemic | 1 |
| Euphorbia aucheri | Non-Endemic | 17 |
| Euphorbia belgheisi | Endemic | 1 |
| Euphorbia erythradenia | Endemic | 4 |
| Euphorbia grisophylla | Endemic | 5 |
| Euphorbia hebecarpa | Endemic | 11 |
| Euphorbia herniariifolia | Endemic | 16 |
| Euphorbia iberica | Non-Endemic | 20 |
| Euphorbia khabrica | Endemic | 1 |
| Euphorbia macrocarpa | Endemic | 22 |
| Euphorbia microsciadia | Non-Endemic | 55 |
| Euphorbia mirzakhaniana | Endemic | 1 |
| Euphorbia plebeia | Endemic | 2 |
| Euphorbia sahendi | Endemic | 31 |
| Euphorbia sanasunitensis | Endemic | 2 |
| Euphorbia sulphurea | Endemic | 1 |
| Euphrasia amblyodonta | Endemic | 1 |
| Euphrasia juzepczukii | Endemic | 13 |
| Euphrasia petiolaris | Non-Endemic | 6 |
| Euphrasia sevanensis | Endemic | 12 |
| Ferula haussknechtii | Endemic | 3 |
| Ferula hezarlalehzarica | Endemic | 3 |
| Ferula microcolea | Endemic | 23 |
| Ferula ovina | Non-Endemic | 47 |
| Ferula setifolia | Endemic | 8 |
| Ferulago angulata | Endemic | 64 |
| Ferulago contracta | Endemic | 10 |
| Festuca adanensis | Endemic | 4 |
| Festuca airoides | Non-Endemic | 5 |
| Festuca alaica | Non-Endemic | 3 |
| Festuca anatolica | Endemic | 11 |
| Festuca artvinensis | Endemic | 8 |
| Festuca brunnescens | Endemic | 7 |
| Festuca bushiana | Endemic | 2 |
| Festuca cappadocica | Endemic | 5 |
| Festuca cataonica | Endemic | 4 |
| Festuca chalcophaea | Endemic | 14 |
| Festuca cratericola | Endemic | 2 |
| Festuca elwendiana | Endemic | 3 |
| Festuca iranica | Endemic | 1 |
| Festuca lazistanica | Endemic | 2 |
| Festuca oreophila | Non-Endemic | 6 |
| Festuca ovina | Non-Endemic | 45 |
| Festuca pinifolia | Endemic | 12 |
| Festuca rechingeri | Endemic | 2 |
| Festuca sabalanica | Endemic | 1 |
| Festuca sclerophylla | Non-Endemic | 5 |
| Festuca skvortsovii | Endemic | 7 |
| Festuca sommieri | Non-Endemic | 2 |
| Festuca sulcata | Non-Endemic | 8 |
| Festuca varia | Non-Endemic | 18 |
| Fibigia multicaulis | Endemic | 15 |
| Fibigia umbellata | Endemic | 18 |
| Fritillaria alburyana | Endemic | 5 |
| Fritillaria aurea | Endemic | 7 |
| Fritillaria caucasica | Endemic | 7 |
| Fritillaria chlorantha | Endemic | 5 |
| Fritillaria crassifolia | Endemic | 28 |
| Fritillaria kotschyana | Endemic | 12 |
| Fritillaria latifolia | Endemic | 6 |
| Fritillaria michailovskyi | Endemic | 3 |
| Fritillaria minima | Endemic | 4 |
| Fritillaria minuta | Endemic | 8 |
| Fritillaria olivieri | Endemic | 3 |
| Fritillaria reuteri | Endemic | 5 |
| Fritillaria zagrica | Endemic | 25 |
| Fuernrohria setifolia | Endemic | 7 |
| Gagea alexeenkoana | Endemic | 32 |
| Gagea alexii | Non-Endemic | 1 |
| Gagea anisanthos | Non-Endemic | 7 |
| Gagea capillifolia | Non-Endemic | 2 |
| Gagea caroli-kochii | Endemic | 9 |
| Gagea confusa | Non-Endemic | 32 |
| Gagea dschungarica | Non-Endemic | 17 |
| Gagea exilis | Non-Endemic | 2 |
| Gagea glacialis | Endemic | 18 |
| Gagea joannis | Endemic | 7 |
| Gagea luteoides | Endemic | 10 |
| Gagea menitskyi | Endemic | 1 |
| Gagea setifolia | Non-Endemic | 1 |
| Gagea sulfurea | Non-Endemic | 5 |
| Gagea uliginosa | Endemic | 27 |
| Galium aladaghense | Endemic | 4 |
| Galium aucheri | Endemic | 11 |
| Galium boreale | Endemic | 6 |
| Galium decumbens | Endemic | 34 |
| Galium delicatulum | Endemic | 3 |
| Galium hyrcanicum | Endemic | 45 |
| Galium majmechense | Endemic | 9 |
| Galium nabelekii | Endemic | 11 |
| Galium nigdeense | Endemic | 1 |
| Galium ovitdaghense | Endemic | 1 |
| Galium pseudokurdicum | Endemic | 27 |
| Galium schoenbeck-Temesyae | Endemic | 1 |
| Galium subvelutinum | Endemic | 19 |
| Galium tuncelianum | Endemic | 1 |
| Galium valantioides | Non-Endemic | 1 |
| Gentiana aquatica | Non-Endemic | 11 |
| Gentiana boissieri | Endemic | 3 |
| Gentiana brachyphylla | Non-Endemic | 2 |
| Gentiana gelida | Endemic | 9 |
| Gentiana nivalis | Non-Endemic | 1 |
| Gentiana pyrenaica | Non-Endemic | 10 |
| Gentiana riparia | Non-Endemic | 1 |
| Gentiana septemfida | Non-Endemic | 29 |
| Gentiana umbellata | Non-Endemic | 8 |
| Gentiana verna | Non-Endemic | 30 |
| Gentianella caucasea | Non-Endemic | 7 |
| Gentianella holosteoides | Endemic | 3 |
| Gentianella umbellata | Non-Endemic | 2 |
| Geranium cinereum | Endemic | 18 |
| Geranium kurdicum | Endemic | 4 |
| Geranium persicum | Endemic | 18 |
| Geranium platypetalum | Non-Endemic | 7 |
| Geum iranicum | Endemic | 1 |
| Geum kokanikum | Non-Endemic | 8 |
| Globularia dumulosa | Endemic | 2 |
| Globularia trichosantha | Endemic | 1 |
| Gnaphalium leucopilinum | Endemic | 4 |
| Gnaphalium stewartii | Non-Endemic | 4 |
| Gnaphalium supinum | Non-Endemic | 13 |
| Graellsia isfahan | Endemic | 1 |
| Graellsia saxifragifolia | Non-Endemic | 28 |
| Graellsia stylosa | Endemic | 8 |
| Gypsophila adenophylla | Endemic | 3 |
| Gypsophila aretioides | Endemic | 43 |
| Gypsophila briquetiana | Endemic | 3 |
| Gypsophila graminifolia | Endemic | 1 |
| Gypsophila hakkiarica | Endemic | 1 |
| Gypsophila lipskyi | Endemic | 3 |
| Gypsophila nabelaelekii | Endemic | 2 |
| Gypsophila peshmenii | Endemic | 2 |
| Gypsophila serpylloides | Endemic | 1 |
| Gypsophila silenoides | Non-Endemic | 7 |
| Gypsophila tenuifolia | Non-Endemic | 7 |
| Gypsophila venusta | Endemic | 1 |
| Gypsophila yazdiana | Endemic | 1 |
| Haussknechtia elymaitica | Endemic | 3 |
| Hedysarum caucasicum | Non-Endemic | 9 |
| Hedysarum erythroleucum | Endemic | 9 |
| Hedysarum hedysaroides | Non-Endemic | 8 |
| Hedysarum persicum | Endemic | 1 |
| Hedysarum vanense | Endemic | 2 |
| Heldreichia bupleurifolia | Endemic | 4 |
| Heldreichia rotundifolia | Endemic | 7 |
| Helichrysum athanaton | Endemic | 2 |
| Helichrysum chionophilum | Endemic | 6 |
| Helichrysum davisianum | Endemic | 4 |
| Helichrysum oligocephalum | Endemic | 78 |
| Helichrysum pallasii | Endemic | 20 |
| Helichrysum psychrophilum | Endemic | 205 |
| Helichrysum yurterianum | Endemic | 1 |
| Helictotrichon argaeum | Endemic | 6 |
| Helictotrichon versicolor | Non-Endemic | 4 |
| Heracleum anisactis | Endemic | 10 |
| Heracleum apiifolium | Non-Endemic | 6 |
| Heracleum crenatifolium | Endemic | 3 |
| Heracleum humile | Endemic | 11 |
| Heracleum pastinacifolium | Endemic | 25 |
| Heracleum rawianum | Endemic | 8 |
| Heracleum schelkovnikovii | Endemic | 3 |
| Heracleum sphondylium | Endemic | 1 |
| Herniaria argaea | Endemic | 5 |
| Herniaria caucasica | Non-Endemic | 10 |
| Herniaria incana | Non-Endemic | 62 |
| Herniaria olympica | Endemic | 3 |
| Hesperis borbasii | Endemic | 1 |
| Hesperis leucoclada | Endemic | 3 |
| Hesperis luristanica | Endemic | 1 |
| Hesperis nivalis | Endemic | 7 |
| Hieracium caucasicum | Non-Endemic | 5 |
| Hieracium echioides | Non-Endemic | 17 |
| Hieracium prenanthoides | Non-Endemic | 6 |
| Hieracium procerum | Non-Endemic | 19 |
| Hieracium rigens | Non-Endemic | 3 |
| Hieracium teberdense | Non-Endemic | 2 |
| Hordeum violaceum | Non-Endemic | 30 |
| Hyalopoa hracziana | Endemic | 1 |
| Hyalopoa pontica | Non-Endemic | 3 |
| Hymenocrater sessilifolius | Non-Endemic | 1 |
| Hymenocrater yazdianus | Endemic | 1 |
| Hyoscyamus kotschyanus | Endemic | 14 |
| Hyoscyamus kurdicus | Endemic | 4 |
| Hyoscyamus malekianus | Endemic | 2 |
| Hyoscyamus senecionis | Non-Endemic | 45 |
| Hypericum armenum | Endemic | 10 |
| Hypericum crenulatum | Endemic | 4 |
| Hypericum linarioides | Non-Endemic | 32 |
| Hypericum musadoganii | Endemic | 1 |
| Hypericum nummularioides | Non-Endemic | 2 |
| Inula acaulis | Endemic | 19 |
| Inula mariae | Endemic | 2 |
| Inula orientalis | Non-Endemic | 7 |
| Inula rhizocephala | Non-Endemic | 4 |
| Iranecio elbrusensis | Endemic | 18 |
| Iranecio oligolepis | Endemic | 4 |
| Iranecio paucilobus | Endemic | 31 |
| Iris barnumiae | Endemic | 50 |
| Isatis brachycarpa | Endemic | 1 |
| Isatis nummularia | Non-Endemic | 1 |
| Isatis takhtajanii | Endemic | 8 |
| Jasione supina | Endemic | 10 |
| Johrenia alpina | Endemic | 2 |
| Johreniopsis scoparia | Endemic | 5 |
| Johreniopsis seseloides | Endemic | 10 |
| Juncus alpigenus | Non-Endemic | 15 |
| Juncus filiformis | Endemic | 3 |
| Juncus rechingeri | Non-Endemic | 9 |
| Juniperus communis | Non-Endemic | 21 |
| Juniperus sabina | Non-Endemic | 8 |
| Jurinea meda | Endemic | 15 |
| Jurinea viciosoi | Endemic | 1 |
| Jurinella frigida | Endemic | 42 |
| Jurinella microcephala | Endemic | 9 |
| Jurinella moschus | Endemic | 66 |
| Kelussia odoratissima | Endemic | 2 |
| Kobresia humilis | Non-Endemic | 3 |
| Kobresia schoenoides | Non-Endemic | 5 |
| Kobresia simpliciuscula | Non-Endemic | 4 |
| Koeleria eriostachya | Non-Endemic | 16 |
| Lactuca denaensis | Endemic | 1 |
| Lactuca hazaranensis | Endemic | 1 |
| Lactuca polyclada | Non-Endemic | 3 |
| Lactuca pumila | Non-Endemic | 1 |
| Lactuca scarioloides | Non-Endemic | 44 |
| Lagochilus kotschyanus | Endemic | 24 |
| Lallemantia canescens | Endemic | 1 |
| Lamium armenum | Endemic | 5 |
| Lamium crinitum | Endemic | 9 |
| Lamium eriocephalum | Endemic | 5 |
| Lamium tomentosum | Endemic | 29 |
| Laserpitium carduchorum | Endemic | 2 |
| Lathyrus bitlisicus | Endemic | 1 |
| Lathyrus brachypterus | Endemic | 5 |
| Lathyrus cyaneus | Endemic | 11 |
| Lathyrus nivalis | Endemic | 7 |
| Leontodon oxylepis | Endemic | 13 |
| Leontodon stenocalathius | Endemic | 1 |
| Leonurus cardiaca | Endemic | 55 |
| Lepechiniella fursei | Endemic | 1 |
| Lepechiniella persica | Endemic | 5 |
| Lepidium pabotii | Endemic | 1 |
| Leucopoa pseudosclerophylla | Endemic | 1 |
| Leutea cupularis | Endemic | 16 |
| Leutea petiolaris | Endemic | 47 |
| Leutea rechingeri | Endemic | 4 |
| Levisticum officinale | Endemic | 1 |
| Ligularia persica | Endemic | 17 |
| Ligularia sibirica | Non-Endemic | 5 |
| Ligusticum alatum | Non-Endemic | 15 |
| Linaria karajensis | Endemic | 2 |
| Linaria remotiflora | Endemic | 4 |
| Linaria schelkownikowii | Endemic | 8 |
| Linaria shahroudensis | Endemic | 2 |
| Linum densiflorum | Endemic | 5 |
| Linum empetrifolium | Endemic | 3 |
| Linum hypericifolium | Non-Endemic | 14 |
| Linum meletonis | Endemic | 4 |
| Linum obtusatum | Endemic | 7 |
| Linum punctatum | Endemic | 6 |
| Linum subbiflorum | Endemic | 6 |
| Linum triflorum | Endemic | 3 |
| Lomatogonium carinthiacum | Non-Endemic | 8 |
| Lophanthus turcicus | Endemic | 1 |
| Lotus corniculatus | Non-Endemic | 24 |
| Luzula luzulina | Endemic | 1 |
| Luzula spicata | Non-Endemic | 22 |
| Luzula stenophylla | Endemic | 22 |
| Lythrum thymifolia | Non-Endemic | 1 |
| Malabaila dasyantha | Endemic | 11 |
| Marrubium astracanicum | Endemic | 145 |
| Marrubium cordatum | Endemic | 9 |
| Marrubium eriocephalum | Endemic | 4 |
| Marrubium heterodon | Endemic | 6 |
| Mattiastrum pygmaeum | Endemic | 2 |
| Melica altissima | Non-Endemic | 2 |
| Mesostemma kotschyana | Endemic | 61 |
| Micrantha multicaulis | Endemic | 4 |
| Microsisymbrium minutiflorum | Non-Endemic | 2 |
| Milium schmidtianum | Non-Endemic | 1 |
| Minuartia aizoides | Non-Endemic | 16 |
| Minuartia aucheriana | Endemic | 4 |
| Minuartia circassica | Non-Endemic | 12 |
| Minuartia dianthifolia | Endemic | 12 |
| Minuartia glandulosa | Endemic | 75 |
| Minuartia hamzaoglui | Endemic | 1 |
| Minuartia imbricata | Non-Endemic | 5 |
| Minuartia lineata | Endemic | 66 |
| Minuartia litwinowii | Endemic | 7 |
| Minuartia oreina | Endemic | 43 |
| Minuartia rimarum | Endemic | 5 |
| Minuartia sabalanica | Endemic | 1 |
| Minuartia sublineata | Endemic | 10 |
| Minuartia umbellulifera | Endemic | 9 |
| Minuartia verna | Non-Endemic | 5 |
| Muscari anatolicum | Endemic | 1 |
| Muscari bourgaei | Endemic | 8 |
| Muscari coeleste | Endemic | 6 |
| Myopordon aucheri | Endemic | 1 |
| Myopordon damavandica | Endemic | 1 |
| Myopordon hyrcanum | Endemic | 2 |
| Myopordon persicum | Endemic | 2 |
| Myosotis alpestris | Non-Endemic | 12 |
| Myosotis asiatica | Non-Endemic | 3 |
| Myosotis guneri | Endemic | 1 |
| Myosotis olympica | Endemic | 20 |
| Myosotis platyphylla | Endemic | 1 |
| Nardus stricta | Non-Endemic | 19 |
| Nepeta alaghezi | Endemic | 7 |
| Nepeta allotria | Endemic | 1 |
| Nepeta archibaldii | Endemic | 3 |
| Nepeta assurgens | Endemic | 3 |
| Nepeta azadkouhensis | Endemic | 1 |
| Nepeta binaloudensis | Endemic | 2 |
| Nepeta bornmuelleri | Endemic | 2 |
| Nepeta chionophila | Endemic | 4 |
| Nepeta crispa | Endemic | 8 |
| Nepeta daenensis | Non-Endemic | 15 |
| Nepeta dschuparensis | Endemic | 4 |
| Nepeta elymaitica | Endemic | 7 |
| Nepeta glomerulosa | Endemic | 67 |
| Nepeta iranshahrii | Endemic | 1 |
| Nepeta lamiifolia | Endemic | 3 |
| Nepeta lasiocephala | Endemic | 7 |
| Nepeta macrosiphon | Endemic | 20 |
| Nepeta menthoides | Endemic | 26 |
| Nepeta monocephala | Endemic | 2 |
| Nepeta natanzensis | Endemic | 4 |
| Nepeta oxyodonta | Endemic | 16 |
| Nepeta pilinux | Endemic | 3 |
| Nepeta pogonosperma | Endemic | 6 |
| Nepeta racemosa | Endemic | 138 |
| Nepeta rivularis | Endemic | 2 |
| Nepeta sahandica | Endemic | 10 |
| Nepeta sessilifolia | Endemic | 9 |
| Nepeta stenantha | Endemic | 6 |
| Nepeta supina | Non-Endemic | 6 |
| Nonea macrantha | Endemic | 5 |
| Nonea persica | Endemic | 62 |
| Nonea pulmonarioides | Endemic | 3 |
| Odontites aucheri | Non-Endemic | 43 |
| Omphalodes luciliae | Endemic | 10 |
| Onobrychis argaea | Endemic | 3 |
| Onobrychis arnacantha | Non-Endemic | 2 |
| Onobrychis cornuta | Non-Endemic | 104 |
| Onobrychis garinensis | Endemic | 1 |
| Onobrychis marashensis | Endemic | 7 |
| Onobrychis oxytropoides | Non-Endemic | 5 |
| Onobrychis plantago | Endemic | 2 |
| Onobrychis transcaucasica | Endemic | 10 |
| Ononis sessilifolia | Endemic | 3 |
| Onosma ghahremanii | Endemic | 1 |
| Onosma haussknechtii | Endemic | 4 |
| Onosma kilouyensis | Endemic | 22 |
| Onosma liparioides | Endemic | 2 |
| Onosma mirabilis | Endemic | 1 |
| Onosma moussavi | Endemic | 1 |
| Onosma proballanthera | Endemic | 2 |
| Onosma sabalanica | Endemic | 3 |
| Onosma stenosiphon | Endemic | 41 |
| Oreopoa anatolica | Endemic | 1 |
| Ornithogalum improbum | Endemic | 1 |
| Orobanche gamosepala | Endemic | 1 |
| Oxyria digyna | Non-Endemic | 24 |
| Oxytropis aellenii | Endemic | 1 |
| Oxytropis albana | Non-Endemic | 6 |
| Oxytropis armeniaca | Endemic | 3 |
| Oxytropis binaludensis | Endemic | 1 |
| Oxytropis caraganetorum | Non-Endemic | 1 |
| Oxytropis cinerea | Endemic | 1 |
| Oxytropis compacta | Endemic | 1 |
| Oxytropis czapan-daghi | Endemic | 4 |
| Oxytropis engizekensis | Endemic | 4 |
| Oxytropis gracillima | Endemic | 1 |
| Oxytropis guilanica | Endemic | 1 |
| Oxytropis heratensis | Non-Endemic | 2 |
| Oxytropis hirsutiuscula | Non-Endemic | 3 |
| Oxytropis immersa | Non-Endemic | 5 |
| Oxytropis Iranica | Endemic | 3 |
| Oxytropis javaherdehi | Endemic | 1 |
| Oxytropis karjaginii | Endemic | 9 |
| Oxytropis kermanica | Endemic | 5 |
| Oxytropis lazica | Endemic | 11 |
| Oxytropis mahneshanensis | Endemic | 1 |
| Oxytropis masanderanensis | Endemic | 3 |
| Oxytropis neo-rechingeriana | Endemic | 1 |
| Oxytropis persica | Endemic | 15 |
| Oxytropis pusilloides | Non-Endemic | 4 |
| Oxytropis salukensis | Endemic | 1 |
| Oxytropis savellanica | Endemic | 9 |
| Oxytropis shahvarica | Endemic | 1 |
| Oxytropis shirkuhi | Endemic | 1 |
| Oxytropis sivehensis | Endemic | 1 |
| Oxytropis sojakii | Non-Endemic | 1 |
| Oxytropis surmandehi | Endemic | 1 |
| Oxytropis sutakensis | Endemic | 1 |
| Oxytropis takhti-soleimanii | Endemic | 1 |
| Oxytropis yazdi | Endemic | 2 |
| Papaver armeniacum | Endemic | 10 |
| Papaver bracteatum | Endemic | 21 |
| Papaver fugax | Endemic | 58 |
| Papaver gabrielianae | Endemic | 1 |
| Papaver lateritium | Non-Endemic | 3 |
| Papaver orientale | Endemic | 22 |
| Papaver polychaetum | Endemic | 5 |
| Papaver pseudo-orientale | Endemic | 9 |
| Papaver sjunicicum | Endemic | 1 |
| Paracaryum lalezarense | Endemic | 1 |
| Paracaryum polyanthum | Endemic | 1 |
| Paracolpodium tzvelevii | Endemic | 1 |
| Paraquilegia caespitosa | Endemic | 5 |
| Parnassia cabulica | Non-Endemic | 3 |
| Parnassia palustris | Non-Endemic | 16 |
| Paronychia davisii | Endemic | 2 |
| Paronychia saxatilis | Endemic | 1 |
| Paronychia turcica | Endemic | 1 |
| Pedicularis atropurpurea | Non-Endemic | 2 |
| Pedicularis cabulica | Non-Endemic | 3 |
| Pedicularis cadmea | Endemic | 12 |
| Pedicularis caucasica | Endemic | 28 |
| Pedicularis comosa | Non-Endemic | 13 |
| Pedicularis crassirostris | Non-Endemic | 11 |
| Pedicularis munzurdaghensis | Endemic | 1 |
| Pedicularis nordmanniana | Non-Endemic | 6 |
| Pedicularis pontica | Non-Endemic | 7 |
| Pedicularis pycnantha | Non-Endemic | 18 |
| Pedicularis rhinanthoides | Non-Endemic | 4 |
| Pedicularis sibthorpii | Non-Endemic | 60 |
| Peltariopsis planisiliqua | Endemic | 1 |
| Pentanema kurdistanicum | Endemic | 1 |
| Petrorhagia sarbaghiae | Endemic | 1 |
| Peucedanum alpinum | Non-Endemic | 4 |
| Peucedanum pimpinellifolia | Non-Endemic | 4 |
| Peucedanum ruthenicum | Non-Endemic | 1 |
| Peucedanum translucens | Endemic | 1 |
| Peucedanum zozimioides | Endemic | 2 |
| Phagnalon persicum | Endemic | 13 |
| Phelipanche zangezuri | Endemic | 1 |
| Phleum alpinum | Non-Endemic | 24 |
| Phleum iranicum | Endemic | 5 |
| Phlomis anisodonta | Endemic | 75 |
| Phlomis ghilanensis C. Koch | Endemic | 1 |
| Physoptychis gnaphalodes | Endemic | 20 |
| Pilosella hoppeana | Non-Endemic | 11 |
| Pimpinella deverroides | Endemic | 18 |
| Pimpinella gedrosiaca | Endemic | 5 |
| Pimpinella saxifraga | Non-Endemic | 21 |
| Pimpinella tragium | Non-Endemic | 56 |
| Piptatherum denaense | Endemic | 1 |
| Piptatherum laterale | Non-Endemic | 4 |
| Piptatherum molinioides | Endemic | 7 |
| Plantago atrata | Non-Endemic | 46 |
| Plantago gentianoides | Non-Endemic | 8 |
| Poa aitchisonii | Non-Endemic | 1 |
| Poa akmanii | Endemic | 2 |
| Poa alpina | Non-Endemic | 27 |
| Poa araratica | Non-Endemic | 25 |
| Poa bussmannii | Endemic | 1 |
| Poa cenisia | Non-Endemic | 7 |
| Poa chaixii | Non-Endemic | 2 |
| Poa greuteri | Endemic | 1 |
| Poa longifolia | Endemic | 16 |
| Poa pseudobulbosa | Endemic | 2 |
| Poa sterilis | Non-Endemic | 9 |
| Poa supina | Non-Endemic | 4 |
| Polygonum bistorta | Non-Endemic | 7 |
| Polygonum dumosum | Endemic | 3 |
| Polygonum luzuloides | Endemic | 22 |
| Polygonum molliaeforme | Non-Endemic | 6 |
| Polygonum serpyllaceum | Non-Endemic | 11 |
| Polygonum spinosum | Endemic | 2 |
| Polygonum thymifolium | Non-Endemic | 18 |
| Polylophium involucratum | Endemic | 4 |
| Potamogeton gramineus | Non-Endemic | 1 |
| Potentilla agrimonioides | Non-Endemic | 2 |
| Potentilla aladaghensis | Endemic | 1 |
| Potentilla anatolica | Endemic | 12 |
| Potentilla argaea | Endemic | 29 |
| Potentilla argyroloma | Endemic | 18 |
| Potentilla aucheriana | Endemic | 29 |
| Potentilla bifurca | Non-Endemic | 19 |
| Potentilla cappadocica | Endemic | 7 |
| Potentilla carduchorum | Endemic | 1 |
| Potentilla crantzii | Non-Endemic | 39 |
| Potentilla cryptophila | Endemic | 6 |
| Potentilla diversidentata | Endemic | 1 |
| Potentilla doddsii | Endemic | 2 |
| Potentilla elvendensis | Endemic | 9 |
| Potentilla flaccida | Endemic | 4 |
| Potentilla fruticosa | Non-Endemic | 7 |
| Potentilla geranioides | Endemic | 13 |
| Potentilla hololeuca | Non-Endemic | 4 |
| Potentilla humifusa | Non-Endemic | 8 |
| Potentilla lazica | Endemic | 2 |
| Potentilla lignosa | Endemic | 6 |
| Potentilla mallota | Endemic | 4 |
| Potentilla meyeri | Endemic | 12 |
| Potentilla multifida | Non-Endemic | 2 |
| Potentilla nuda | Endemic | 18 |
| Potentilla nurensis | Endemic | 6 |
| Potentilla oweriniana | Non-Endemic | 4 |
| Potentilla palustris | Non-Endemic | 3 |
| Potentilla pannosa | Endemic | 11 |
| Potentilla polyschista | Endemic | 9 |
| Potentilla porphyrantha | Endemic | 25 |
| Potentilla poteriifolia | Endemic | 9 |
| Potentilla pulvinaris | Endemic | 6 |
| Potentilla rupestris | Non-Endemic | 15 |
| Potentilla ruprechtii | Non-Endemic | 8 |
| Potentilla sangedehensis | Endemic | 1 |
| Potentilla sawalensis | Endemic | 2 |
| Potentilla seidlitziana | Non-Endemic | 2 |
| Potentilla speciosa | Non-Endemic | 12 |
| Potentilla subpalmata | Endemic | 6 |
| Potentilla szovitsii | Non-Endemic | 1 |
| Potentilla thuringiaca | Non-Endemic | 9 |
| Prangos ferulacea | Non-Endemic | 46 |
| Prangos platychlaena | Endemic | 4 |
| Prangos tuberculata | Endemic | 12 |
| Prangos uloptera | Non-Endemic | 51 |
| Prenanthes glareosa | Endemic | 3 |
| Primula algida | Non-Endemic | 23 |
| Primula auriculata | Non-Endemic | 75 |
| Primula capitellata | Non-Endemic | 9 |
| Primula elatior | Non-Endemic | 21 |
| Primula longipes | Non-Endemic | 4 |
| Psathyrostachys fragilis | Non-Endemic | 10 |
| Psephellus khalkhalensis | Endemic | 2 |
| Psephellus transcaucasicus | Endemic | 3 |
| Pseudocamelina aphragmodes | Endemic | 1 |
| Pseudocamelina glaucophylla | Endemic | 18 |
| Pseudocamelina kermanica | Endemic | 1 |
| Psychrogeton aellenii | Endemic | 2 |
| Psychrogeton alexeenkoi | Non-Endemic | 4 |
| Psychrogeton amorphoglossus | Non-Endemic | 29 |
| Psychrogeton andryaloides | Non-Endemic | 1 |
| Psychrogeton aucheri | Non-Endemic | 7 |
| Psychrogeton chionophilus | Endemic | 2 |
| Psychrogeton persicus | Endemic | 8 |
| Pulsatilla albana | Endemic | 25 |
| Puschkinia bilgineri | Endemic | 1 |
| Puschkinia kurdica | Endemic | 1 |
| Puschkinia scilloides | Endemic | 52 |
| Ranunculus anatolicus | Endemic | 1 |
| Ranunculus aragazi | Endemic | 6 |
| Ranunculus aucheri | Endemic | 56 |
| Ranunculus bingoeldaghensis | Endemic | 1 |
| Ranunculus brachylobus | Endemic | 26 |
| Ranunculus bulbilliferus | Endemic | 6 |
| Ranunculus caucasicus | Non-Endemic | 9 |
| Ranunculus crateris | Endemic | 2 |
| Ranunculus crymophilus | Endemic | 69 |
| Ranunculus dalechanensis | Endemic | 1 |
| Ranunculus demissus | Endemic | 8 |
| Ranunculus dissectus | Endemic | 20 |
| Ranunculus divaricatus | Non-Endemic | 3 |
| Ranunculus diversifolius | Endemic | 16 |
| Ranunculus elymaiticus | Endemic | 10 |
| Ranunculus eriorrhizus | Endemic | 5 |
| Ranunculus fenzlii | Endemic | 6 |
| Ranunculus grandiflorus | Endemic | 12 |
| Ranunculus microflorus | Endemic | 1 |
| Ranunculus obesus | Non-Endemic | 2 |
| Ranunculus oreophilus | Non-Endemic | 14 |
| Ranunculus papyrocarpus | Endemic | 6 |
| Ranunculus pichleri | Endemic | 3 |
| Ranunculus polyrhizos | Non-Endemic | 2 |
| Ranunculus renzii | Endemic | 1 |
| Ranunculus sojakii | Endemic | 2 |
| Ranunculus straussii | Endemic | 4 |
| Ranunculus tempskyanus | Endemic | 2 |
| Ranunculus termei | Endemic | 2 |
| Ranunculus transcaucasicus | Endemic | 7 |
| Ranunculus trichocarpus | Endemic | 18 |
| Ranunculus vanensis | Endemic | 2 |
| Ranunculus vermirrhizus | Endemic | 1 |
| Ranunculus zenjanensis | Endemic | 2 |
| Rhabdosciadium anatolyi | Endemic | 1 |
| Rhabdosciadium aucheri | Endemic | 15 |
| Rhabdosciadium petiolare | Endemic | 2 |
| Rhamnus cornifolia | Endemic | 36 |
| Rhamnus prostrata | Non-Endemic | 2 |
| Rhynchocorys kurdica | Endemic | 4 |
| Ribes anatolicum | Endemic | 1 |
| Ribes orientale | Non-Endemic | 3 |
| Ricotia aucheri | Endemic | 4 |
| Ricotia varians | Endemic | 1 |
| Rindera albida | Endemic | 5 |
| Rindera caespitosa | Endemic | 7 |
| Rosa beggeriana | Non-Endemic | 61 |
| Rosularia aizoon | Endemic | 11 |
| Rosularia chrysantha | Endemic | 5 |
| Rosularia davisii | Endemic | 2 |
| Rosularia elymaitica | Endemic | 8 |
| Rosularia persica | Non-Endemic | 22 |
| Rosularia pseudohaussknechtii | Endemic | 1 |
| Rosularia rechingeri | Endemic | 4 |
| Rosularia sempervivum | Endemic | 32 |
| Rosularia serpentinica | Endemic | 3 |
| Rubia caramanica | Endemic | 4 |
| Rubia pauciflora | Endemic | 12 |
| Rumex acetoselloides | Endemic | 12 |
| Rumex alpinus | Non-Endemic | 6 |
| Rumex angustifolius | Endemic | 26 |
| Rumex caucasicus | Non-Endemic | 1 |
| Rumex elbursensis | Endemic | 17 |
| Rumex gracilescens | Endemic | 3 |
| Rumex patientia | Non-Endemic | 8 |
| Rumex ponticus | Endemic | 7 |
| Sagina saginoides | Non-Endemic | 13 |
| Salsola canescens | Endemic | 32 |
| Salvia lachnocalyx | Endemic | 1 |
| Salvia pachystachya | Endemic | 8 |
| Salvia rhytidea | Non-Endemic | 4 |
| Salvia sahendica | Endemic | 8 |
| Salvia staminea | Endemic | 27 |
| Saponaria iranica | Endemic | 1 |
| Saponaria pumilio | Endemic | 6 |
| Satureja kallarica | Endemic | 1 |
| Saussurea salsa | Non-Endemic | 2 |
| Saxifraga exarata | Non-Endemic | 16 |
| Saxifraga hirculus | Non-Endemic | 2 |
| Saxifraga iranica | Endemic | 10 |
| Saxifraga juniperifolia | Non-Endemic | 2 |
| Saxifraga koelzii | Endemic | 1 |
| Saxifraga kolenatiana | Non-Endemic | 1 |
| Saxifraga ramsarica | Endemic | 2 |
| Saxifraga sibirica | Non-Endemic | 26 |
| Saxifraga wendelboi | Endemic | 6 |
| Scabiosa caucasica | Non-Endemic | 14 |
| Scilla alinihatiana | Endemic | 1 |
| Scleranthus uncinatus | Non-Endemic | 25 |
| Sclerochorton haussknechtii | Endemic | 2 |
| Scorzonera cana | Non-Endemic | 40 |
| Scorzonera grossheimii | Endemic | 7 |
| Scorzonera intricata | Endemic | 22 |
| Scorzonera karkasensis | Endemic | 1 |
| Scorzonera kirpicznikovii | Endemic | 1 |
| Scorzonera meyeri | Endemic | 30 |
| Scorzonera nivalis | Endemic | 1 |
| Scorzonera psychrophila | Endemic | 3 |
| Scorzonera pygmaea | Endemic | 4 |
| Scorzonera raddeana | Non-Endemic | 32 |
| Scorzonera radicosa | Endemic | 14 |
| Scorzonera rigida | Endemic | 18 |
| Scorzonera seidlitzii | Endemic | 1 |
| Scorzonera sericea | Endemic | 6 |
| Scorzonera stenocephala | Endemic | 15 |
| Scorzonera subaphylla | Endemic | 6 |
| Scorzonera xylobasis | Endemic | 1 |
| Scrophularia amplexicaulis | Endemic | 36 |
| Scrophularia atroglandulosa | Endemic | 4 |
| Scrophularia catariifolia | Endemic | 15 |
| Scrophularia chlorantha | Endemic | 3 |
| Scrophularia chrysantha | Non-Endemic | 20 |
| Scrophularia crassicaulis | Endemic | 11 |
| Scrophularia crassiuscula | Endemic | 8 |
| Scrophularia fatmae | Endemic | 1 |
| Scrophularia flava | Endemic | 2 |
| Scrophularia frigida | Endemic | 109 |
| Scrophularia gorganica | Endemic | 4 |
| Scrophularia kurdica | Endemic | 2 |
| Scrophularia libanotica | Endemic | 4 |
| Scrophularia olympica | Non-Endemic | 18 |
| Scrophularia pumilio | Endemic | 2 |
| Scrophularia subaequiloba | Endemic | 1 |
| Scrophularia subaphylla | Endemic | 26 |
| Scutellaria araxensis | Endemic | 1 |
| Scutellaria glechomoides | Endemic | 21 |
| Scutellaria heterophylla | Endemic | 11 |
| Scutellaria multicaulis | Endemic | 30 |
| Scutellaria patonii | Endemic | 1 |
| Scutellaria pinnatifida | Endemic | 103 |
| Scutellaria pontica | Non-Endemic | 5 |
| Sedum adscendens | Non-Endemic | 5 |
| Sedum alpestre | Non-Endemic | 5 |
| Sedum annuum | Non-Endemic | 16 |
| Sedum artvinensis | Endemic | 2 |
| Sedum euxinum | Endemic | 3 |
| Sedum exarata | Non-Endemic | 15 |
| Sedum gracile | Non-Endemic | 19 |
| Sedum hewittii | Endemic | 1 |
| Sedum juniperifolia | Non-Endemic | 4 |
| Sedum kotschyanum | Endemic | 20 |
| Sedum luteoviride | Non-Endemic | 3 |
| Sedum moschata | Non-Endemic | 8 |
| Sedum nanum | Endemic | 8 |
| Sedum oppositifolium | Non-Endemic | 12 |
| Sedum paniculatum | Non-Endemic | 14 |
| Sedum sempervivum | Non-Endemic | 1 |
| Sedum sibiricum | Non-Endemic | 11 |
| Sedum subulatum | Non-Endemic | 12 |
| Sedum tenellum | Endemic | 24 |
| Sedum transcaucasicum | Non-Endemic | 1 |
| Semenovia dichotoma | Endemic | 8 |
| Semenovia frigida | Endemic | 15 |
| Semenovia subscaposa | Endemic | 5 |
| Semenovia suffruticosa | Endemic | 6 |
| Semenovia tragioides | Endemic | 25 |
| Sempervivum atropatanum | Endemic | 5 |
| Sempervivum globiferum | Endemic | 1 |
| Sempervivum iranicum | Endemic | 15 |
| Sempervivum pisidicum | Endemic | 4 |
| Sempervivum transcaucasicum | Endemic | 14 |
| Sempervivum tunaekimii | Endemic | 1 |
| Senecio cilicius | Endemic | 10 |
| Senecio davisii | Endemic | 3 |
| Senecio eligulatus | Endemic | 1 |
| Senecio eriospermus | Endemic | 14 |
| Senecio hypochionaeus | Endemic | 4 |
| Senecio integrifolius | Non-Endemic | 12 |
| Senecio iranicus | Endemic | 5 |
| Senecio jurineifolius | Endemic | 3 |
| Senecio kotschyanus | Endemic | 1 |
| Senecio munzurdaglarensis | Endemic | 1 |
| Senecio subnivalis | Endemic | 2 |
| Senecio taraxacifolius | Endemic | 15 |
| Senecio vulcanicus | Endemic | 5 |
| Serratula hakkiarica | Endemic | 1 |
| Serratula haussknechtii | Endemic | 17 |
| Serratula melanocheila | Endemic | 1 |
| Seseli grandivittatum | Endemic | 2 |
| Sesleria araratica | Endemic | 2 |
| Sesleria phleoides | Endemic | 30 |
| Sibbaldia parviflora | Non-Endemic | 52 |
| Sideritis phlomoides | Endemic | 4 |
| Silene araratica | Endemic | 5 |
| Silene argaea | Endemic | 2 |
| Silene azirensis | Endemic | 1 |
| Silene balansae | Endemic | 1 |
| Silene bolanthoides | Endemic | 1 |
| Silene caroli-henrici | Endemic | 2 |
| Silene cartilaginea | Endemic | 2 |
| Silene caryophylloides | Endemic | 3 |
| Silene caucasica | Endemic | 1 |
| Silene cephalantha | Endemic | 12 |
| Silene chustupica | Endemic | 1 |
| Silene daenensis | Endemic | 4 |
| Silene delicatula | Endemic | 1 |
| Silene demawendica | Endemic | 1 |
| Silene dianthoides | Endemic | 19 |
| Silene dschuparensis | Endemic | 1 |
| Silene erciyesdaghensis | Endemic | 1 |
| Silene eremicana | Endemic | 2 |
| Silene ghahremaninejadii | Endemic | 1 |
| Silene goniocaula | Endemic | 10 |
| Silene guntensis | Non-Endemic | 2 |
| Silene gynodioica | Endemic | 37 |
| Silene hirticalyx | Endemic | 1 |
| Silene konuralpii | Endemic | 1 |
| Silene lasiantha | Endemic | 16 |
| Silene laxa | Endemic | 12 |
| Silene lucida | Endemic | 9 |
| Silene marschallii | Endemic | 33 |
| Silene meyeri | Endemic | 34 |
| Silene miksensis | Endemic | 1 |
| Silene nuncupanda | Endemic | 5 |
| Silene nurensis | Endemic | 12 |
| Silene odontopetala | Endemic | 75 |
| Silene oreades | Endemic | 5 |
| Silene orientoalborzensis | Endemic | 1 |
| Silene oxelmanii | Endemic | 1 |
| Silene persica | Endemic | 6 |
| Silene pseudonurensis | Endemic | 2 |
| Silene pungens | Endemic | 13 |
| Silene renzii | Endemic | 1 |
| Silene rhynchocarpa | Endemic | 14 |
| Silene ruprechtii | Non-Endemic | 12 |
| Silene saxatilis | Non-Endemic | 9 |
| Silene tachtensis | Non-Endemic | 1 |
| Silene tragacantha | Endemic | 2 |
| Silene viscosa | Non-Endemic | 20 |
| Silene yildirimlii | Endemic | 1 |
| Solenanthus circinatus | Non-Endemic | 50 |
| Solenanthus stamineus | Non-Endemic | 45 |
| Solidago virgaurea | Non-Endemic | 20 |
| Sorbus tamamschjanae | Endemic | 7 |
| Spergularia lycia | Endemic | 3 |
| Stachys acerosa | Endemic | 25 |
| Stachys balansae | Endemic | 5 |
| Stachys choruhensis | Endemic | 2 |
| Stachys citrina | Endemic | 13 |
| Stachys lanigera | Endemic | 2 |
| Stachys macrantha | Non-Endemic | 7 |
| Stachys obtusicrena | Endemic | 15 |
| Stachys pilifera | Endemic | 25 |
| Stachys rizeensis | Endemic | 1 |
| Stachys subnuda | Endemic | 3 |
| Stefanoffia insoluta | Endemic | 1 |
| Stellaria graminea | Non-Endemic | 3 |
| Stellaria persica | Endemic | 9 |
| Stellaria scaturiginella | Endemic | 1 |
| Stenotaenia elbursensis | Endemic | 1 |
| Stenotaenia haussknechtii | Endemic | 1 |
| Stenotaenia nudicaulis | Endemic | 9 |
| Stipa hohenackeriana | Non-Endemic | 31 |
| Stipa joannis | Non-Endemic | 1 |
| Stipa pennata | Non-Endemic | 9 |
| Stipa tirsa | Non-Endemic | 1 |
| Swertia iberica | Non-Endemic | 6 |
| Swertia lactea | Non-Endemic | 1 |
| Swertia longifolia | Endemic | 24 |
| Tanacetum bachtiaricum | Endemic | 3 |
| Tanacetum balsamita | Non-Endemic | 41 |
| Tanacetum cappadocicum | Endemic | 2 |
| Tanacetum caucasicum | Non-Endemic | 21 |
| Tanacetum chiliophyllum | Non-Endemic | 49 |
| Tanacetum coccineum | Non-Endemic | 10 |
| Tanacetum dumosum | Endemic | 16 |
| Tanacetum fruticulosum | Non-Endemic | 6 |
| Tanacetum hololeucum | Endemic | 23 |
| Tanacetum kotschyi | Endemic | 57 |
| Tanacetum macrophyllum | Non-Endemic | 3 |
| Tanacetum mucroniferum | Endemic | 5 |
| Tanacetum nitens | Endemic | 5 |
| Tanacetum nivale | Endemic | 2 |
| Tanacetum pamiricum | Non-Endemic | 2 |
| Tanacetum persicum | Endemic | 34 |
| Tanacetum polycephalum | Endemic | 71 |
| Tanacetum punctatum | Non-Endemic | 20 |
| Tanacetum tenuisectum | Endemic | 23 |
| Tanacetum zahlbruckneri | Endemic | 6 |
| Tanacetum zangezuricum | Endemic | 1 |
| Taraxacum baltistanicum | Non-Endemic | 2 |
| Taraxacum brevirostre | Non-Endemic | 8 |
| Taraxacum chitralense | Non-Endemic | 1 |
| Taraxacum koelzii | Non-Endemic | 3 |
| Taraxacum neospurium | Endemic | 4 |
| Taraxacum oliganthum | Endemic | 11 |
| Taraxacum primigenium | Endemic | 5 |
| Taraxacum scolopendrinum | Endemic | 1 |
| Taraxacum stenolepium | Non-Endemic | 1 |
| Taraxacum stevenii | Non-Endemic | 33 |
| Tetrataenium lasiopetalum | Endemic | 35 |
| Teucrium ozturkii | Endemic | 1 |
| Thesium cilicicum | Endemic | 3 |
| Thlaspi crassum | Endemic | 1 |
| Thlaspi kurdicum | Endemic | 4 |
| Thlaspi maassoumii | Endemic | 1 |
| Thlaspi papillosum | Endemic | 1 |
| Thlaspi pulvinata | Endemic | 1 |
| Thlaspi pulvinatum | Endemic | 1 |
| Thlaspi pumilum | Non-Endemic | 1 |
| Thlaspi sintenisii | Endemic | 2 |
| Thlaspi stenocarpum | Endemic | 7 |
| Thlaspi tenue | Endemic | 4 |
| Thlaspi valerianoides | Endemic | 2 |
| Thlaspi watsonii | Endemic | 2 |
| Thymus brachychilus | Endemic | 6 |
| Thymus carmanicus | Endemic | 11 |
| Thymus cherlerioides | Endemic | 4 |
| Thymus collinus | Non-Endemic | 8 |
| Thymus fallax | Endemic | 19 |
| Thymus fedtschenkoi | Endemic | 10 |
| Thymus persicus | Endemic | 2 |
| Thymus praecox | Endemic | 13 |
| Thymus pubescens | Endemic | 87 |
| Thymus turkmenii | Endemic | 1 |
| Trachydium depressum | Endemic | 28 |
| Trachydium eriocarpum | Endemic | 4 |
| Trachydium kotschyi | Endemic | 29 |
| Trachydium pauciradiatum | Endemic | 16 |
| Tragopogon erostris | Endemic | 1 |
| Tragopogon jesdianus | Endemic | 10 |
| Tragopogon kotschyi | Endemic | 21 |
| Tragopogon pusillus | Non-Endemic | 4 |
| Tragopogon reticulatus | Endemic | 24 |
| Trichophorum pumilum | Non-Endemic | 15 |
| Trifolium badium | Non-Endemic | 38 |
| Trifolium kurdistanicum | Endemic | 1 |
| Trifolium longidentatum | Endemic | 5 |
| Trifolium montanum | Endemic | 4 |
| Trifolium polyphyllum | Non-Endemic | 2 |
| Trifolium pratense | Non-Endemic | 37 |
| Trifolium radicosum | Endemic | 12 |
| Trifolium repens | Non-Endemic | 17 |
| Trifolium sintenisii | Endemic | 6 |
| Trifolium spadiceum | Non-Endemic | 2 |
| Tripleurospermum caucasicum | Non-Endemic | 19 |
| Tripleurospermum melanolepis | Endemic | 7 |
| Trisetum geghamense | Endemic | 1 |
| Trisetum rigidum | Non-Endemic | 26 |
| Trisetum thospiticum | Endemic | 2 |
| Trisetum turcicum | Endemic | 6 |
| Trollius ranunculoides | Endemic | 10 |
| Tulipa humilis | Endemic | 45 |
| Tulipa koyuncui | Endemic | 1 |
| Valeriana alliariifolia | Non-Endemic | 32 |
| Valeriana alpestris | Endemic | 11 |
| Valeriana bolkarica | Endemic | 1 |
| Valeriana clarkei | Non-Endemic | 5 |
| Valeriana montana | Non-Endemic | 2 |
| Valeriana saxicola | Endemic | 2 |
| Valeriana sisymbriifolia | Non-Endemic | 79 |
| Vania campylophylla | Endemic | 1 |
| Vavilovia formosa | Endemic | 16 |
| Verbascum bornmuellerianum | Endemic | 2 |
| Verbascum bourgeauanum | Endemic | 1 |
| Verbascum carmanicum | Endemic | 5 |
| Verbascum faik-karaveliogullarii | Endemic | 1 |
| Verbascum georgicum | Endemic | 1 |
| Verbascum kurdistanicum | Endemic | 1 |
| Verbascum speciosum | Endemic | 1 |
| Verbascum subnivale | Endemic | 2 |
| Verbascum tauri | Endemic | 1 |
| Veronica allahuekberensis | Endemic | 1 |
| Veronica armena | Endemic | 12 |
| Veronica aucheri | Endemic | 22 |
| Veronica baranetzkii | Endemic | 3 |
| Veronica beccabunga | Endemic | 39 |
| Veronica biloba | Non-Endemic | 62 |
| Veronica bombycina | Endemic | 7 |
| Veronica caespitosa | Endemic | 7 |
| Veronica daranica | Endemic | 2 |
| Veronica davisii | Endemic | 2 |
| Veronica euphrasiifolia Link | Endemic | 1 |
| Veronica fragilis | Endemic | 4 |
| Veronica fridericae | Endemic | 2 |
| Veronica gentianoides | Endemic | 56 |
| Veronica hispidula | Non-Endemic | 15 |
| Veronica kopetdaghensis | Endemic | 2 |
| Veronica kopgecidiensis | Endemic | 5 |
| Veronica kotschyana | Endemic | 2 |
| Veronica kurdica | Endemic | 51 |
| Veronica longipedicellata | Endemic | 2 |
| Veronica mirabilis | Endemic | 3 |
| Veronica montbretii | Endemic | 1 |
| Veronica orientalis | Endemic | 121 |
| Veronica paederotae | Endemic | 7 |
| Veronica polium | Endemic | 2 |
| Veronica pusilla | Non-Endemic | 21 |
| Veronica quezelii | Endemic | 1 |
| Veronica rechingeri | Endemic | 14 |
| Veronica rubrifolia | Endemic | 51 |
| Veronica surculosa | Endemic | 3 |
| Veronica tauricola | Endemic | 9 |
| Veronica telephiifolia | Endemic | 4 |
| Veronica thymoides | Endemic | 2 |
| Vicia akhmaganica | Non-Endemic | 2 |
| Vicia alpestris | Endemic | 19 |
| Vicia canescens | Endemic | 40 |
| Vicia ciceroidea | Endemic | 10 |
| Vicia glareosa | Endemic | 1 |
| Vicia multijuga | Endemic | 7 |
| Viola oreades | Non-Endemic | 3 |
| Viola pachyrrhiza | Endemic | 4 |
| Viola rupestris | Non-Endemic | 10 |
| Viola spathulata | Endemic | 11 |
| Woodsia alpina | Non-Endemic | 3 |
| Xanthogalum purpurascens | Endemic | 18 |
| Zeravschania aucheri | Endemic | 34 |
| Zerdana anchonioides | Endemic | 6 |
| Ziziphora clinopodioides | Endemic | 34 |
|  |  |  |

**Table S2** Species contributing to the score of a consensus area using total alpine species dataset and Endemicity Analysis approach (areas of concordant species distribution patterns).

1. **Taurus AE:**

179 Arabis androsacea (179): (0.000-0.667)

476 Centaurea mucronifera (476): (0.000-0.679)

605 Dianthus brevicaulis (605): (0.500-0.625)

692 Erigeron cilicicus (692): (0.500)

699 Erodium cedrorum (699): (0.625-0.667)

713 Erysimum kotschyanum (713): (0.000-0.531)

866 Helichrysum chionophilum (866): (0.000-0.583)

922 Johrenia alpina (922): (0.000-0.611)

956 Leontodon oxylepis (956): (0.531-0.538)

993 Marrubium heterodon (993): (0.000-0.571)

1124 Papaver polychaetum (1124): (0.625-0.667)

1155 Peucedanum zozimioides (1155): (0.000-0.611)

1272 Ranunculus demissus (1272): (0.682-0.850)

1336 Saponaria pumilio (1336): (0.000-0.591)

1360 Scorzonera pygmaea (1360): (0.000-0.500)

1500 Stachys citrina (1500): (0.000-0.536)

1. **Eastern Taurus AE:**

232 Astragalus aladagensis (232): (0.750)

359 Astragalus stridii (359): (0.750)

612 Dianthus lactiflorus (612): (0.750)

774 Fritillaria aurea (774): (0.375)

818 Gentiana boissieri (818): (0.750)

828 Gentianella holosteoides (828): (0.750)

906 Hypericum crenulatum (906): (0.875)

949 Lamium eriocephalum (949): (0.875)

975 Linum empetrifolium (975): (0.875)

1225 Potentilla pulvinaris (1225): (0.750)

1239 Prenanthes glareosa (1239): (0.750)

1381 Scrophularia libanotica (1381): (0.500)

1475 Silene nuncupanda (1475): (0.500)

1554 Thesium cilicicum (1554): (0.750)

1. onica kotschyana (1640): (0.750)
2. **Anatolian-Armenian AE:**

3 Acantholimon calvertii (3): (0.000-0.700)

6 Acantholimon dianthifolium (6): (0.000-0.833)

26 Achillea latiloba (26): (0.000-0.875)

30 Aconitum cochleare (30): (0.000-0.750)

31 Aethionema caespitosum (31): (0.000-0.607)

38 Aethionema speciosum (38): (0.000-0.636)

50 Agrostis vinealis (50): (0.000-0.625)

57 Alchemilla dura (57): (0.000-0.750)

58 Alchemilla ellenbergiana (58): (0.000-0.656)

61 Alchemilla farinosa (61): (0.000-0.500)

64 Alchemilla grossheimii (64): (0.000-1.000)

73 Alchemilla minusculiflora (73): (0.000-0.688)

75 Alchemilla oriturcica (75): (0.000-0.750)

87 Alchemilla rizensis (87): (0.000-0.833)

88 Alchemilla sedelmeyeriana (88): (0.000-1.000)

90 Alchemilla sericea (90): (0.000-0.700)

94 Alchemilla venosa (94): (0.000-0.833)

100 Allium anacoleum (100): (0.000-0.700)

102 Allium aucheri (102): (0.000-0.786)

110 Allium djimilense (110): (0.000-0.750)

113 Allium egorovae (113): (0.000-0.667)

120 Allium kunthianum (120): (0.000-0.633)

123 Allium mahneshanense (123): (0.000-0.500)

127 Allium pseudoampeloprasum (127): (0.000-0.667)

128 Allium pseudostrictum (128): (0.000-0.875)

130 Allium sabalense (130): (0.000-0.700)

131 Allium sahandicum (131): (0.000-0.667)

134 Allium shatakiense (134): (0.000-0.857)

135 Allium stearnianum (135): (0.000-0.833)

144 Alopecurus dasyanthus (144): (0.000-0.773)

145 Alopecurus glacialis (145): (0.000-0.714)

147 Alopecurus laguroides (147): (0.000-0.682)

157 Alyssum gehamense (157): (0.000-0.875)

161 Alyssum peltarioides (161): (0.000-0.600)

170 Androsace caduca (170): (0.000-1.000)

171 Androsace chamaejasme (171): (0.000-0.750)

172 Androsace intermedia (172): (0.000-0.813)

173 Androsace multiscapa (173): (0.000-0.545)

175 Anemone fasciculata (175): (0.000-0.875)

176 Antennaria dioica (176): (0.000-0.750)

177 Anthemis marschalliana (177): (0.000-0.875)

180 Arabis brachycarpa (180): (0.000-0.643)

181 Arabis carduchorum (181): (0.000-0.722)

183 Arabis graellsiiformis (183): (0.000-0.833)

190 Arenaria blepharophylla (190): (0.000-0.750)

194 Arenaria dianthoides (194): (0.000-0.667)

198 Arenaria lychnidea (198): (0.000-0.643)

214 Asperula affinis (214): (0.000-0.773)

218 Asperula laxiflora (218): (0.000-0.479)

221 Asperula pontica (221): (0.000-0.750)

222 Asperula prostrata (222): (0.000-0.733)

228 Astragalus acmophyllus (228): (0.000-0.722)

231 Astragalus agassii (231): (0.000-0.750)

234 Astragalus alpinus (234): (0.000-0.875)

256 Astragalus czorochensis (256): (0.000-0.750)

258 Astragalus dasycarpus (258): (0.000-0.833)

259 Astragalus declinatus (259): (0.000-0.632)

263 Astragalus dzebrailicus (263): (0.000-0.650)

266 Astragalus erivanensis (266): (0.000-1.000)

267 Astragalus ermineus (267): (0.000-0.833)

268 Astragalus euoplus (268): (0.000-1.000)

272 Astragalus fraxinifolius (272): (0.000-0.725)

273 Astragalus frickii (273): (0.000-0.813)

276 Astragalus gezeldarensis (276): (0.000-1.000)

278 Astragalus globosus (278): (0.000-0.794)

288 Astragalus horasanicus (288): (0.000-0.700)

291 Astragalus hyalolepis (291): (0.000-0.767)

293 Astragalus incertus (293): (0.000-0.733)

298 Astragalus karabaghensis (298): (0.000-0.708)

300 Astragalus lanatus (300): (0.000-0.611)

303 Astragalus latus (303): (0.000-0.833)

304 Astragalus leiophyllus (304): (0.000-1.000)

312 Astragalus melanocarpus (312): (0.000-0.611)

321 Astragalus nezaketiae (321): (0.000-0.667)

325 Astragalus oreades (325): (0.000-0.750)

329 Astragalus pauperiflorus (329): (0.000-0.800)

333 Astragalus perrarus (333): (0.000-0.714)

339 Astragalus polyanthus (339): (0.000-0.727)

340 Astragalus polygala (340): (0.000-1.000)

345 Astragalus rechingeri (345): (0.000-0.833)

352 Astragalus sachanewii (352): (0.000-0.875)

353 Astragalus sahendi (353): (0.000-0.800)

354 Astragalus savellanicus (354): (0.000-0.700)

367 Astragalus uraniolimneus (367): (0.000-0.600)

368 Astragalus vavilovii (368): (0.000-0.750)

372 Astragalus zohrabi (372): (0.000-0.679)

374 Asyneuma filipes (374): (0.000-0.773)

377 Asyneuma pulchellum (377): (0.000-0.833)

379 Athyrium distentifolium (379): (0.000-0.875)

382 Aurinia rupestris (382): (0.000-0.778)

387 Bellardiochloa polychroa (387): (0.000-0.679)

392 Bornmuellera cappadocica (392): (0.000-0.625)

394 Briza marcowiczii (394): (0.000-0.833)

395 Bromus armenus (395): (0.000-0.667)

413 Campanula armena (413): (0.000-0.833)

414 Campanula bayerniana (414): (0.000-0.563)

417 Campanula collina (417): (0.000-0.750)

418 Campanula conferta (418): (0.000-0.538)

420 Campanula hedgei (420): (0.000-0.667)

423 Campanula karakuschensis (423): (0.000-0.682)

427 Campanula saxifraga (427): (0.000-0.833)

431 Campanula zangezura (431): (0.000-0.750)

432 Carduus lanuginosus (432): (0.000-0.611)

434 Carex atrata (434): (0.000-0.643)

435 Carex brevicollis (435): (0.000-0.857)

436 Carex capitellata (436): (0.000-0.769)

439 Carex disticha (439): (0.000-0.875)

441 Carex magellanica (441): (0.000-0.833)

442 Carex medwedewii (442): (0.000-0.553)

445 Carex michelii (445): (0.000-0.750)

447 Carex oligantha (447): (0.000-0.542)

452 Carex pontica (452): (0.000-0.833)

454 Carex pyrenaica (454): (0.000-0.750)

457 Carex transcaucasica (457): (0.000-0.750)

458 Carex tristis (458): (0.000-0.423)

461 Carum caucasicum (461): (0.000-0.667)

462 Carum komarovii (462): (0.000-0.750)

463 Carum meifolium (463): (0.000-0.750)

466 Centaurea appendicigera (466): (0.000-0.750)

467 Centaurea armena (467): (0.000-0.750)

468 Centaurea cheiranthifolia (468): (0.000-0.889)

469 Centaurea congesta (469): (0.000-0.900)

472 Centaurea incanescens (472): (0.000-0.875)

473 Centaurea karduchorum (473): (0.000-0.833)

474 Centaurea lanigera (474): (0.000-0.462)

477 Centaurea nigrofimbria (477): (0.000-0.875)

480 Centaurea pulcherrima (480): (0.000-0.769)

489 Cephalaria sparsipilosa (489): (0.000-0.700)

490 Cerastium araraticum (490): (0.000-0.875)

492 Cerastium davuricum (492): (0.000-0.750)

493 Cerastium gnaphalodes (493): (0.000-0.781)

496 Cerastium pseudokasbek (496): (0.000-0.750)

498 Cerastium szowitsii (498): (0.000-1.000)

502 Chaerophyllum astrantiae (502): (0.000-0.833)

509 Chamaesciadium acaule (509): (0.000-0.632)

515 Cicerbita adenophora (515): (0.000-0.833)

520 Cirsium mimitum (520): (0.000-0.833)

521 Cirsium obvallatum (521): (0.000-1.000)

523 Cirsium pseudobracteosum (523): (0.000-0.833)

525 Cirsium simplex (525): (0.000-0.750)

526 Cirsium tomentosum (526): (0.000-0.688)

529 Cochlearia aucheri (529): (0.000-0.636)

530 Cochlearia sintenisii (530): (0.000-0.750)

531 Colchicum kurdicum (531): (0.000-0.833)

532 Colpodium araraticum (532): (0.000-0.692)

533 Colpodium fibrosum (533): (0.000-0.778)

534 Colpodium gillettii (534): (0.000-0.833)

540 Coronilla orientalis (540): (0.000-0.833)

542 Corydalis alpestris (542): (0.000-0.708)

543 Corydalis conorhiza (543): (0.000-0.667)

544 Corydalis persica (544): (0.000-0.650)

570 Crepis armena (570): (0.000-0.769)

572 Crepis bupleurifolia (572): (0.000-0.750)

574 Crepis conyzifolia (574): (0.000-0.875)

576 Crepis dioritica (576): (0.000-0.714)

582 Crepis pannonica (582): (0.000-0.750)

583 Crepis sahendi (583): (0.000-0.550)

584 Crepis willdenowii (584): (0.000-0.550)

586 Crocus kotschyanus (586): (0.000-0.750)

588 Crocus vallicola (588): (0.000-0.833)

590 Cymbocarpum erythraeum (590): (0.000-0.750)

592 Daphne glomerata (592): (0.000-0.875)

593 Daphne magakjanii (593): (0.000-0.750)

594 Daphne oleoides (594): (0.000-0.600)

595 Delphinium carduchorum (595): (0.000-0.833)

597 Delphinium flexuosum (597): (0.000-0.813)

601 Delphinium linearilobum (601): (0.000-0.733)

616 Dianthus multicaulis (616): (0.000-0.692)

617 Dianthus muschianus (617): (0.000-0.688)

619 Dianthus raddeanus (619): (0.000-0.750)

622 Dianthus seidlitzii (622): (0.000-0.700)

653 Doronicum macrophyllum (653): (0.000-0.559)

654 Doronicum maximum (654): (0.000-0.536)

655 Doronicum oblongifolium (655): (0.000-0.750)

657 Draba araratica (657): (0.000-0.786)

662 Draba polytricha (662): (0.000-0.818)

679 Elymus transhyrcanus (679): (0.000-0.667)

680 Empetrum nigrum (680): (0.000-0.857)

702 Eryngium bornmuelleri (702): (0.000-0.833)

708 Erysimum gelidum (708): (0.000-0.588)

715 Erysimum munzuriense (715): (0.000-0.750)

719 Erysimum sintenisianum (719): (0.000-0.750)

724 Euphorbia grisophylla (724): (0.000-0.875)

733 Euphorbia sahendi (733): (0.000-0.700)

734 Euphorbia sanasunitensis (734): (0.000-0.833)

738 Euphrasia petiolaris (738): (0.000-1.000)

739 Euphrasia sevanensis (739): (0.000-0.553)

740 Ferula haussknechtii (740): (0.000-0.833)

744 Ferula setifolia (744): (0.000-0.714)

751 Festuca artvinensis (751): (0.000-1.000)

752 Festuca brunnescens (752): (0.000-0.700)

753 Festuca bushiana (753): (0.000-0.833)

756 Festuca chalcophaea (756): (0.000-0.618)

758 Festuca elwendiana (758): (0.000-0.615)

761 Festuca oreophila (761): (0.000-0.682)

766 Festuca sclerophylla (766): (0.000-0.750)

767 Festuca skvortsovii (767): (0.000-0.667)

773 Fritillaria alburyana (773): (0.000-0.563)

779 Fritillaria latifolia (779): (0.000-0.875)

780 Fritillaria michailovskyi (780): (0.000-0.700)

782 Fritillaria minuta (782): (0.000-0.708)

786 Fuernrohria setifolia (786): (0.000-0.875)

789 Gagea anisanthos (789): (0.000-1.000)

796 Gagea joannis (796): (0.000-1.000)

800 Gagea sulfurea (800): (0.000-0.750)

808 Galium majmechense (808): (0.000-0.875)

809 Galium nabelekii (809): (0.000-0.813)

820 Gentiana gelida (820): (0.000-0.594)

822 Gentiana pyrenaica (822): (0.000-0.750)

829 Gentianella umbellata (829): (0.000-0.833)

831 Geranium kurdicum (831): (0.000-0.833)

833 Geranium platypetalum (833): (0.000-0.778)

839 Gnaphalium stewartii (839): (0.000-0.625)

844 Gypsophila adenophylla (844): (0.000-0.833)

846 Gypsophila briquetiana (846): (0.000-0.667)

849 Gypsophila lipskyi (849): (0.000-0.750)

850 Gypsophila nabelaelekii (850): (0.000-0.667)

853 Gypsophila silenoides (853): (0.000-0.708)

854 Gypsophila tenuifolia (854): (0.000-0.750)

858 Hedysarum caucasicum (858): (0.000-0.875)

859 Hedysarum erythroleucum (859): (0.000-0.556)

860 Hedysarum hedysaroides (860): (0.000-0.800)

863 Heldreichia bupleurifolia (863): (0.000-0.722)

872 Helictotrichon argaeum (872): (0.000-0.607)

875 Heracleum apiifolium (875): (0.000-0.700)

876 Heracleum crenatifolium (876): (0.000-0.800)

879 Heracleum rawianum (879): (0.000-0.667)

880 Heracleum schelkovnikovii (880): (0.000-0.875)

882 Herniaria argaea (882): (0.000-0.667)

894 Hieracium rigens (894): (0.000-0.750)

895 Hieracium teberdense (895): (0.000-0.700)

909 Hypericum nummularioides (909): (0.000-0.833)

911 Inula mariae (911): (0.000-0.667)

912 Inula orientalis (912): (0.000-0.813)

925 Juncus alpigenus (925): (0.000-0.594)

926 Juncus filiformis (926): (0.000-0.833)

937 Kobresia schoenoides (937): (0.000-0.682)

939 Koeleria eriostachya (939): (0.000-0.615)

947 Lamium armenum (947): (0.000-0.917)

948 Lamium crinitum (948): (0.000-0.679)

951 Laserpitium carduchorum (951): (0.000-0.833)

954 Lathyrus cyaneus (954): (0.000-0.667)

955 Lathyrus nivalis (955): (0.000-0.800)

965 Leutea rechingeri (965): (0.000-0.643)

968 Ligularia sibirica (968): (0.000-0.750)

969 Ligusticum alatum (969): (0.000-0.656)

972 Linaria schelkownikowii (972): (0.000-0.875)

974 Linum densiflorum (974): (0.000-0.875)

976 Linum hypericifolium (976): (0.000-0.808)

977 Linum meletonis (977): (0.000-0.714)

978 Linum obtusatum (978): (0.000-0.591)

979 Linum punctatum (979): (0.000-0.833)

980 Linum subbiflorum (980): (0.000-1.000)

981 Linum triflorum (981): (0.000-0.700)

982 Lomatogonium carinthiacum (982): (0.000-0.773)

989 Malabaila dasyantha (989): (0.000-1.000)

991 Marrubium cordatum (991): (0.000-0.917)

995 Melica altissima (995): (0.000-0.667)

1002 Minuartia circassica (1002): (0.000-0.833)

1006 Minuartia imbricata (1006): (0.000-0.722)

1014 Minuartia verna (1014): (0.000-0.750)

1017 Muscari coeleste (1017): (0.000-0.722)

1022 Myosotis alpestris (1022): (0.000-0.731)

1028 Nepeta alaghezi (1028): (0.000-0.875)

1053 Nepeta sahandica (1053): (0.000-0.800)

1056 Nepeta supina (1056): (0.000-0.786)

1057 Nonea macrantha (1057): (0.000-0.813)

1067 Onobrychis oxytropoides (1067): (0.000-0.750)

1069 Onobrychis transcaucasica (1069): (0.000-0.750)

1072 Onosma haussknechtii (1072): (0.000-0.750)

1077 Onosma proballanthera (1077): (0.000-0.700)

1078 Onosma sabalanica (1078): (0.000-0.700)

1085 Oxytropis albana (1085): (0.000-0.654)

1102 Oxytropis lazica (1102): (0.000-0.792)

1123 Papaver orientale (1123): (0.000-0.364)

1141 Pedicularis crassirostris (1141): (0.000-0.786)

1143 Pedicularis nordmanniana (1143): (0.000-0.667)

1144 Pedicularis pontica (1144): (0.000-0.833)

1152 Peucedanum pimpinellifolia (1152): (0.000-0.688)

1168 Piptatherum laterale (1168): (0.000-1.000)

1180 Poa longifolia (1180): (0.000-0.526)

1208 Potentilla fruticosa (1208): (0.000-0.750)

1209 Potentilla geranioides (1209): (0.000-0.786)

1211 Potentilla humifusa (1211): (0.000-0.525)

1219 Potentilla oweriniana (1219): (0.000-0.833)

1220 Potentilla palustris (1220): (0.000-0.700)

1223 Potentilla porphyrantha (1223): (0.000-0.500)

1227 Potentilla ruprechtii (1227): (0.000-0.813)

1230 Potentilla seidlitziana (1230): (0.000-0.750)

1234 Potentilla thuringiaca (1234): (0.000-0.667)

1240 Primula algida (1240): (0.000-0.923)

1244 Primula longipes (1244): (0.000-0.875)

1245 Psathyrostachys fragilis (1245): (0.000-0.889)

1247 Psephellus transcaucasicus (1247): (0.000-0.875)

1263 Ranunculus aragazi (1263): (0.000-0.875)

1268 Ranunculus caucasicus (1268): (0.000-0.875)

1274 Ranunculus divaricatus (1274): (0.000-0.750)

1275 Ranunculus diversifolius (1275): (0.000-0.500)

1278 Ranunculus fenzlii (1278): (0.000-0.656)

1281 Ranunculus obesus (1281): (0.000-0.750)

1289 Ranunculus tempskyanus (1289): (0.000-0.750)

1291 Ranunculus transcaucasicus (1291): (0.000-0.727)

1301 Rhynchocorys kurdica (1301): (0.000-0.714)

1304 Ricotia aucheri (1304): (0.000-0.833)

1307 Rindera caespitosa (1307): (0.000-0.750)

1309 Rosularia aizoon (1309): (0.000-0.667)

1315 Rosularia rechingeri (1315): (0.000-0.833)

1320 Rumex acetoselloides (1320): (0.000-1.000)

1321 Rumex alpinus (1321): (0.000-0.875)

1327 Rumex ponticus (1327): (0.000-0.577)

1331 Salvia pachystachya (1331): (0.000-0.786)

1333 Salvia sahendica (1333): (0.000-0.700)

1338 Saussurea salsa (1338): (0.000-0.643)

1340 Saxifraga hirculus (1340): (0.000-0.750)

1342 Saxifraga juniperifolia (1342): (0.000-0.750)

1365 Scorzonera sericea (1365): (0.000-0.833)

1371 Scrophularia catariifolia (1371): (0.000-0.500)

1372 Scrophularia chlorantha (1372): (0.000-0.714)

1373 Scrophularia chrysantha (1373): (0.000-0.792)

1388 Scutellaria heterophylla (1388): (0.000-0.611)

1392 Scutellaria pontica (1392): (0.000-0.875)

1399 Sedum gracile (1399): (0.000-0.767)

1401 Sedum juniperifolia (1401): (0.000-0.875)

1406 Sedum oppositifolium (1406): (0.000-1.000)

1418 Sempervivum atropatanum (1418): (0.000-0.800)

1422 Sempervivum transcaucasicum (1422): (0.000-1.000)

1424 Senecio cilicius (1424): (0.000-0.769)

1425 Senecio davisii (1425): (0.000-0.875)

1427 Senecio eriospermus (1427): (0.000-0.800)

1429 Senecio integrifolius (1429): (0.000-0.929)

1435 Senecio taraxacifolius (1435): (0.000-0.618)

1445 Silene araratica (1445): (0.000-1.000)

1451 Silene cartilaginea (1451): (0.000-0.833)

1452 Silene caryophylloides (1452): (0.000-0.667)

1459 Silene dianthoides (1459): (0.000-0.521)

1469 Silene lasiantha (1469): (0.000-0.567)

1471 Silene lucida (1471): (0.000-0.857)

1486 Silene ruprechtii (1486): (0.000-0.727)

1495 Sorbus tamamschjanae (1495): (0.000-0.722)

1498 Stachys balansae (1498): (0.000-0.833)

1502 Stachys macrantha (1502): (0.000-0.667)

1506 Stachys subnuda (1506): (0.000-0.611)

1518 Swertia iberica (1518): (0.000-0.875)

1525 Tanacetum chiliophyllum (1525): (0.000-0.542)

1526 Tanacetum coccineum (1526): (0.000-1.000)

1531 Tanacetum macrophyllum (1531): (0.000-1.000)

1532 Tanacetum mucroniferum (1532): (0.000-0.750)

1533 Tanacetum nitens (1533): (0.000-0.656)

1534 Tanacetum nivale (1534): (0.000-0.833)

1538 Tanacetum punctatum (1538): (0.000-0.767)

1540 Tanacetum zahlbruckneri (1540): (0.000-0.813)

1556 Thlaspi kurdicum (1556): (0.000-0.667)

1562 Thlaspi sintenisii (1562): (0.000-0.750)

1564 Thlaspi tenue (1564): (0.000-0.500)

1565 Thlaspi valerianoides (1565): (0.000-0.833)

1570 Thymus collinus (1570): (0.000-1.000)

1572 Thymus fedtschenkoi (1572): (0.000-0.833)

1584 Tragopogon pusillus (1584): (0.000-0.643)

1585 Tragopogon reticulatus (1585): (0.000-0.765)

1589 Trifolium longidentatum (1589): (0.000-1.000)

1590 Trifolium montanum (1590): (0.000-0.571)

1591 Trifolium polyphyllum (1591): (0.000-0.833)

1595 Trifolium sintenisii (1595): (0.000-0.688)

1596 Trifolium spadiceum (1596): (0.000-0.700)

1597 Tripleurospermum caucasicum (1597): (0.000-0.767)

1598 Tripleurospermum melanolepis (1598): (0.000-0.833)

1602 Trisetum turcicum (1602): (0.000-0.656)

1607 Valeriana alpestris (1607): (0.000-0.643)

1611 Valeriana saxicola (1611): (0.000-0.643)

1625 Veronica armena (1625): (0.000-0.875)

1627 Veronica baranetzkii (1627): (0.000-0.643)

1654 Veronica telephiifolia (1654): (0.000-0.750)

1662 Viola oreades (1662): (0.000-0.750)

1666 Woodsia alpina (1666): (0.000-0.875)

1. **Alborz AE:**

5 Acantholimon demavendicum (5): (0.000-0.600)

11 Acantholimon hohenackeri (11): (0.000-0.571)

24 Achillea aucheri (24): (0.000-0.654)

27 Achillea millefolium (27): (0.000-0.800)

37 Aethionema semnanensis (37): (0.000-0.833)

55 Alchemilla citrina (55): (0.000-0.800)

62 Alchemilla fluminea (62): (0.000-0.615)

63 Alchemilla gigantodus (63): (0.000-0.688)

66 Alchemilla hessii (66): (0.000-0.500)

71 Alchemilla melancholica (71): (0.000-0.700)

72 Alchemilla microscopica (72): (0.000-0.688)

78 Alchemilla pectiniloba (78): (0.000-0.833)

80 Alchemilla plicatissima (80): (0.000-0.750)

98 Allium alamutense (98): (0.000-0.357)

109 Allium derderianum (109): (0.000-0.769)

114 Allium elburzense (114): (0.000-0.800)

153 Alyssopsis mollis (153): (0.000-0.750)

163 Alyssum polycladum (163): (0.000-0.472)

185 Arabis rimarum (185): (0.000-0.500)

211 Artemisia melanolepis (211): (0.000-0.708)

230 Astragalus aestivorum (230): (0.000-0.833)

237 Astragalus atricapillus (237): (0.000-0.500)

244 Astragalus beckii (244): (0.000-0.667)

245 Astragalus bounophilus (245): (0.000-0.600)

247 Astragalus capax (247): (0.000-0.875)

248 Astragalus capito (248): (0.000-1.000)

253 Astragalus chrysanthus (253): (0.000-1.000)

255 Astragalus confusus (255): (0.000-0.563)

260 Astragalus demavendicus (260): (0.000-0.900)

296 Astragalus jodotropis (296): (0.000-0.800)

308 Astragalus macrosemius (308): (0.000-0.500)

314 Astragalus modestus (314): (0.000-0.800)

315 Astragalus monanthemus (315): (0.000-0.833)

322 Astragalus nezva-montis (322): (0.000-0.667)

328 Astragalus patrius (328): (0.000-0.447)

332 Astragalus perdurans (332): (0.000-0.500)

336 Astragalus platysematus (336): (0.000-0.833)

348 Astragalus rubriflorus (348): (0.000-1.000)

391 Betonica nivea (391): (0.000-1.000)

398 Bromus confinis (398): (0.000-0.667)

404 Bufonia koelzii (404): (0.000-0.600)

424 Campanula lourica (424): (0.000-0.600)

465 Catabrosa aquatica (465): (0.000-0.412)

471 Centaurea elbrusensis (471): (0.000-0.682)

528 Clastopus vestitus (528): (0.000-0.429)

557 Cousinia gmelini (557): (0.000-1.000)

565 Cousinia pterocaulos (565): (0.000-0.650)

567 Cousinia shahvarica (567): (0.000-0.500)

571 Crepis asadbarensis (571): (0.000-1.000)

575 Crepis demavendi (575): (0.000-0.833)

577 Crepis elbursensis (577): (0.000-0.800)

596 Delphinium elbursense (596): (0.000-0.607)

647 Diplotaenia damavandica (647): (0.000-0.833)

648 Dolichorrhiza persica (648): (0.000-1.000)

663 Draba pulchella (663): (0.000-0.538)

667 Dracocephalum aucheri (667): (0.000-0.600)

676 Elburzia fenestrata (676): (0.000-0.800)

677 Eleutherospermum cicutarium (677): (0.000-0.529)

694 Erigeron hyrcanicus (694): (0.000-0.600)

700 Erodium dimorphum (700): (0.000-0.667)

704 Erysimum caespitosum (704): (0.000-0.656)

737 Euphrasia juzepczukii (737): (0.000-0.438)

778 Fritillaria kotschyana (778): (0.000-0.750)

791 Gagea caroli-kochii (791): (0.000-0.500)

803 Galium aucheri (803): (0.000-0.688)

805 Galium decumbens (805): (0.000-0.708)

807 Galium hyrcanicum (807): (0.000-0.462)

825 Gentiana umbellata (825): (0.000-0.618)

843 Graellsia stylosa (843): (0.000-0.833)

870 Helichrysum psychrophilum (870): (0.000-0.524)

874 Heracleum anisactis (874): (0.000-0.727)

883 Herniaria caucasica (883): (0.000-0.600)

890 Hieracium caucasicum (890): (0.000-0.571)

892 Hieracium prenanthoides (892): (0.000-0.682)

902 Hyoscyamus kurdicus (902): (0.000-0.538)

914 Iranecio elbrusensis (914): (0.000-0.727)

915 Iranecio oligolepis (915): (0.000-0.833)

917 Iris barnumiae (917): (0.000-0.900)

928 Juniperus communis (928): (0.000-0.524)

929 Juniperus sabina (929): (0.000-0.500)

932 Jurinella frigida (932): (0.000-0.833)

945 Lagochilus kotschyanus (945): (0.000-0.500)

958 Leonurus cardiaca (958): (0.000-0.833)

960 Lepechiniella persica (960): (0.000-0.500)

967 Ligularia persica (967): (0.000-0.833)

1045 Nepeta menthoides (1045): (0.000-0.636)

1098 Oxytropis Iranica (1098): (0.000-0.833)

1118 Papaver armeniacum (1118): (0.000-0.386)

1130 Paraquilegia caespitosa (1130): (0.000-0.833)

1146 Pedicularis rhinanthoides (1146): (0.000-0.563)

1159 Phleum iranicum (1159): (0.000-0.800)

1184 Polygonum bistorta (1184): (0.000-0.324)

1191 Polylophium involucratum (1191): (0.000-1.000)

1214 Potentilla mallota (1214): (0.000-0.500)

1216 Potentilla multifida (1216): (0.000-0.833)

1222 Potentilla polyschista (1222): (0.000-0.536)

1267 Ranunculus bulbilliferus (1267): (0.000-0.545)

1326 Rumex patientia (1326): (0.000-0.500)

1347 Saxifraga wendelboi (1347): (0.000-0.833)

1353 Scorzonera grossheimii (1353): (0.000-0.469)

1357 Scorzonera meyeri (1357): (0.000-0.462)

1374 Scrophularia crassicaulis (1374): (0.000-0.833)

1387 Scutellaria glechomoides (1387): (0.000-0.800)

1420 Sempervivum iranicum (1420): (0.000-0.818)

1436 Senecio vulcanicus (1436): (0.000-0.800)

1472 Silene marschallii (1472): (0.000-0.477)

1473 Silene meyeri (1473): (0.000-0.397)

1508 Stellaria graminea (1508): (0.000-0.615)

1509 Stellaria persica (1509): (0.000-0.600)

1513 Stenotaenia nudicaulis (1513): (0.000-0.692)

1529 Tanacetum hololeucum (1529): (0.000-0.833)

1545 Taraxacum koelzii (1545): (0.000-0.700)

1546 Taraxacum neospurium (1546): (0.000-0.700)

1563 Thlaspi stenocarpum (1563): (0.000-0.750)

1580 Trachydium pauciradiatum (1580): (0.000-0.688)

1583 Tragopogon kotschyi (1583): (0.000-1.000)

1626 Veronica aucheri (1626): (0.000-0.750)

1643 Veronica mirabilis (1643): (0.000-0.833)

1646 Veronica paederotae (1646): (0.000-1.000)

1650 Veronica rechingeri (1650): (0.000-0.917)

1656 Vicia akhmaganica (1656): (0.000-0.700)

1664 Viola rupestris (1664): (0.000-0.654)

1665 Viola spathulata (1665): (0.000-1.000)

1. **Zagros AE:**

2 Acantholimon brachystachyum (2): (0.000-0.467)

9 Acantholimon eschkerense (9): (0.000-0.700)

15 Acantholimon nigricans (15): (0.000-0.500)

16 Acantholimon oliganthum (16): (0.000-0.800)

18 Acantholimon scabrellum (18): (0.000-1.000)

20 Acantholimon tomentellum (20): (0.000-0.875)

42 Aethionema umbellatum (42): (0.000-0.833)

103 Allium austroiranicum (103): (0.000-0.435)

122 Allium longivaginatum (122): (0.000-0.875)

162 Alyssum persicum (162): (0.000-0.875)

201 Arenaria persica (201): (0.000-0.633)

216 Asperula fragillima (216): (0.000-0.800)

223 Asperula rechingeri (223): (0.000-0.875)

251 Astragalus chartostegius (251): (0.000-0.875)

269 Astragalus exspectatus (269): (0.000-0.700)

277 Astragalus ghashghaicus (277): (0.000-0.750)

294 Astragalus inexpectatus (294): (0.000-0.750)

297 Astragalus johannis (297): (0.000-0.643)

313 Astragalus melanodon (313): (0.000-0.700)

319 Astragalus murinus (319): (0.000-0.654)

358 Astragalus stenostegius (358): (0.000-0.583)

390 Bellevalia tristis (390): (0.000-0.875)

422 Campanula humillima (422): (0.000-0.875)

425 Campanula luristanica (425): (0.000-0.875)

507 Chaerophyllum nivale (507): (0.000-0.750)

548 Cousinia archibaldii (548): (0.000-0.875)

561 Cousinia lasiolepis (561): (0.000-0.773)

631 Dionysia caespitosa (631): (0.000-0.900)

632 Dionysia cristagalli (632): (0.000-0.750)

732 Euphorbia plebeia (732): (0.000-0.700)

746 Ferulago contracta (746): (0.000-0.382)

784 Fritillaria reuteri (784): (0.000-0.722)

865 Helichrysum athanaton (865): (0.000-0.625)

889 Hesperis nivalis (889): (0.000-0.773)

930 Jurinea meda (930): (0.000-0.542)

935 Kelussia odoratissima (935): (0.000-0.750)

1001 Minuartia aucheriana (1001): (0.000-0.875)

1021 Myopordon persicum (1021): (0.000-0.750)

1035 Nepeta chionophila (1035): (0.000-0.750)

1048 Nepeta oxyodonta (1048): (0.000-0.433)

1054 Nepeta sessilifolia (1054): (0.000-0.563)

1073 Onosma kilouyensis (1073): (0.000-0.471)

1163 Pimpinella deverroides (1163): (0.000-0.600)

1206 Potentilla elvendensis (1206): (0.000-0.682)

1213 Potentilla lignosa (1213): (0.000-0.500)

1276 Ranunculus elymaiticus (1276): (0.000-0.800)

1284 Ranunculus pichleri (1284): (0.000-0.500)

1288 Ranunculus straussii (1288): (0.000-0.750)

1290 Ranunculus termei (1290): (0.000-0.700)

1297 Rhabdosciadium aucheri (1297): (0.000-0.650)

1319 Rubia pauciflora (1319): (0.000-0.591)

1359 Scorzonera psychrophila (1359): (0.000-0.700)

1367 Scorzonera subaphylla (1367): (0.000-0.750)

1375 Scrophularia crassiuscula (1375): (0.000-0.900)

1413 Semenovia dichotoma (1413): (0.000-0.750)

1414 Semenovia frigida (1414): (0.000-1.000)

1450 Silene caroli-henrici (1450): (0.000-0.750)

1481 Silene persica (1481): (0.000-0.875)

1482 Silene pseudonurensis (1482): (0.000-0.625)

1503 Stachys obtusicrena (1503): (0.000-0.625)

1504 Stachys pilifera (1504): (0.000-0.750)

1527 Tanacetum dumosum (1527): (0.000-0.591)

1579 Trachydium kotschyi (1579): (0.000-0.500)

1634 Veronica fragilis (1634): (0.000-0.750)

1. **Yazd-Kerman AE:**

12 Acantholimon kermanense (12): (0.000-0.786)

14 Acantholimon modestum (14): (0.000-0.700)

19 Acantholimon sirchense (19): (0.000-0.750)

22 Acantholimon zaeifii (22): (0.000-0.714)

108 Allium cathodicarpum (108): (0.000-0.500)

121 Allium lalesaricum (121): (0.000-0.750)

191 Arenaria bulica (191): (0.000-0.643)

227 Astragalus abditus (227): (0.000-0.409)

257 Astragalus daenaensis (257): (0.000-0.611)

284 Astragalus heterodoxus (284): (0.000-0.750)

344 Astragalus pseudoshebarensis (344): (0.000-0.385)

351 Astragalus rufescens (351): (0.000-0.550)

364 Astragalus tenuiscapus (364): (0.000-0.643)

393 Brachyactis roylei (393): (0.000-0.643)

501 Chaenorhinum grossecostatum (501): (0.000-0.650)

508 Chamaegeron asterellus (508): (0.000-0.556)

568 Cousinia sicigera (568): (0.000-0.643)

641 Dionysia rhaptodes (641): (0.000-0.500)

672 Dracocephalum polychaetum (672): (0.000-0.750)

711 Erysimum hezarense (711): (0.000-0.750)

717 Erysimum polatschekii (717): (0.000-0.750)

971 Linaria remotiflora (971): (0.000-0.786)

1031 Nepeta assurgens (1031): (0.000-0.750)

1034 Nepeta bornmuelleri (1034): (0.000-0.750)

1038 Nepeta dschuparensis (1038): (0.000-0.750)

1068 Onobrychis plantago (1068): (0.000-0.643)

1095 Oxytropis heratensis (1095): (0.000-0.643)

1131 Parnassia cabulica (1131): (0.000-0.750)

1137 Pedicularis cabulica (1137): (0.000-0.750)

1185 Polygonum dumosum (1185): (0.000-0.643)

1242 Primula capitellata (1242): (0.000-0.667)

1252 Psychrogeton alexeenkoi (1252): (0.000-0.500)

1277 Ranunculus eriorrhizus (1277): (0.000-0.786)

1283 Ranunculus papyrocarpus (1283): (0.000-0.700)

1332 Salvia rhytidea (1332): (0.000-0.750)

1354 Scorzonera intricata (1354): (0.000-0.438)

1434 Senecio subnivalis (1434): (0.000-0.750)

1456 Silene daenensis (1456): (0.000-0.500)

1617 Verbascum carmanicum (1617): (0.000-0.875)

**Table S3** Species contributing to the score of a consensus area using endemic alpine species dataset and Endemicity Analysis approach (areas of endemism).

1. **Taurus AE:**

131 Arabis androsacea (131): (0.000-0.667)

375 Centaurea mucronifera (375): (0.000-0.679)

479 Dianthus brevicaulis (479): (0.500-0.625)

548 Erigeron cilicicus (548): (0.500)

553 Erodium cedrorum (553): (0.625-0.667)

567 Erysimum kotschyanum (567): (0.000-0.531)

679 Helichrysum chionophilum (679): (0.000-0.583)

716 Johrenia alpina (716): (0.000-0.611)

739 Leontodon oxylepis (739): (0.531-0.538)

769 Marrubium heterodon (769): (0.000-0.571)

875 Papaver polychaetum (875): (0.625-0.667)

892 Peucedanum zozimioides (892): (0.000-0.611)

963 Ranunculus demissus (963): (0.682-0.850)

1014 Saponaria pumilio (1014): (0.000-0.591)

1029 Scorzonera pygmaea (1029): (0.000-0.500)

1. chys citrina (1141): (0.000-0.536)
2. **Eastern Taurus AE:**

169 Astragalus aladagensis (169): (0.750)

292 Astragalus stridii (292): (0.750)

486 Dianthus lactiflorus (486): (0.750)

615 Fritillaria aurea (615): (0.375)

649 Gentiana boissieri (649): (0.750)

651 Gentianella holosteoides (651): (0.750)

705 Hypericum crenulatum (705): (0.875)

732 Lamium eriocephalum (732): (0.875)

756 Linum empetrifolium (756): (0.875)

935 Potentilla pulvinaris (935): (0.750)

941 Prenanthes glareosa (941): (0.750)

1048 Scrophularia libanotica (1048): (0.500)

1123 Silene nuncupanda (1123): (0.500)

1173 Thesium cilicicum (1173): (0.750)

1243 Veronica kotschyana (1243): (0.750)

1. **Anatolian-Armenian AE:**

3 Acantholimon calvertii (3): (0.000-0.600)

6 Acantholimon dianthifolium (6): (0.000-0.833)

25 Achillea latiloba (25): (0.000-0.750)

29 Aconitum cochleare (29): (0.000-0.722)

36 Aethionema speciosum (36): (0.000-0.625)

47 Alchemilla farinosa (47): (0.000-0.500)

57 Alchemilla oriturcica (57): (0.000-0.667)

65 Alchemilla rizensis (65): (0.000-0.667)

73 Allium anacoleum (73): (0.000-0.667)

75 Allium aucheri (75): (0.000-0.722)

83 Allium djimilense (83): (0.000-0.700)

86 Allium egorovae (86): (0.000-0.700)

95 Allium mahneshanense (95): (0.000-0.500)

98 Allium pseudoampeloprasum (98): (0.000-0.700)

100 Allium sabalense (100): (0.000-0.700)

101 Allium sahandicum (101): (0.000-0.667)

103 Allium shatakiense (103): (0.000-0.857)

104 Allium stearnianum (104): (0.000-0.833)

112 Alopecurus laguroides (112): (0.000-0.682)

119 Alyssum gehamense (119): (0.000-0.773)

122 Alyssum peltarioides (122): (0.000-0.600)

130 Androsace multiscapa (130): (0.000-0.545)

132 Arabis brachycarpa (132): (0.000-0.643)

133 Arabis carduchorum (133): (0.000-0.722)

135 Arabis graellsiiformis (135): (0.000-0.833)

142 Arenaria blepharophylla (142): (0.000-0.786)

157 Asperula affinis (157): (0.000-0.773)

160 Asperula laxiflora (160): (0.000-0.420)

161 Asperula prostrata (161): (0.000-0.441)

165 Astragalus acmophyllus (165): (0.000-0.722)

168 Astragalus agassii (168): (0.000-0.750)

191 Astragalus czorochensis (191): (0.000-0.688)

193 Astragalus dasycarpus (193): (0.000-0.833)

194 Astragalus declinatus (194): (0.000-0.556)

198 Astragalus dzebrailicus (198): (0.000-0.500)

201 Astragalus erivanensis (201): (0.000-1.000)

202 Astragalus ermineus (202): (0.000-0.833)

203 Astragalus euoplus (203): (0.000-1.000)

207 Astragalus fraxinifolius (207): (0.000-0.444)

208 Astragalus frickii (208): (0.000-0.750)

211 Astragalus gezeldarensis (211): (0.000-1.000)

213 Astragalus globosus (213): (0.000-0.794)

223 Astragalus horasanicus (223): (0.000-0.643)

226 Astragalus hyalolepis (226): (0.000-0.667)

228 Astragalus incertus (228): (0.000-0.750)

233 Astragalus karabaghensis (233): (0.000-0.367)

235 Astragalus lanatus (235): (0.000-0.667)

238 Astragalus latus (238): (0.000-0.750)

239 Astragalus leiophyllus (239): (0.000-0.875)

246 Astragalus melanocarpus (246): (0.000-0.611)

255 Astragalus nezaketiae (255): (0.000-0.667)

262 Astragalus pauperiflorus (262): (0.000-0.800)

266 Astragalus perrarus (266): (0.000-0.714)

272 Astragalus polyanthus (272): (0.000-0.611)

273 Astragalus polygala (273): (0.000-1.000)

278 Astragalus rechingeri (278): (0.000-0.833)

285 Astragalus sachanewii (285): (0.000-0.875)

286 Astragalus sahendi (286): (0.000-0.800)

287 Astragalus savellanicus (287): (0.000-0.700)

301 Astragalus vavilovii (301): (0.000-0.750)

305 Astragalus zohrabi (305): (0.000-0.679)

307 Asyneuma filipes (307): (0.000-0.750)

310 Asyneuma pulchellum (310): (0.000-0.833)

312 Athyrium distentifolium (312): (0.000-0.750)

314 Aurinia rupestris (314): (0.000-0.778)

319 Bellardiochloa polychroa (319): (0.000-0.679)

324 Bornmuellera cappadocica (324): (0.000-0.625)

325 Bromus armenus (325): (0.000-0.667)

337 Campanula armena (337): (0.000-0.731)

338 Campanula bayerniana (338): (0.000-0.571)

341 Campanula collina (341): (0.000-0.727)

342 Campanula conferta (342): (0.000-0.533)

344 Campanula hedgei (344): (0.000-0.667)

347 Campanula karakuschensis (347): (0.000-0.556)

351 Campanula saxifraga (351): (0.000-0.733)

354 Campanula zangezura (354): (0.000-0.750)

355 Carduus lanuginosus (355): (0.000-0.529)

356 Carex capitellata (356): (0.000-0.818)

357 Carex medwedewii (357): (0.000-0.474)

362 Carex tristis (362): (0.000-0.315)

363 Carum caucasicum (363): (0.000-0.567)

364 Carum komarovii (364): (0.000-0.750)

367 Centaurea appendicigera (367): (0.000-0.750)

368 Centaurea armena (368): (0.000-0.800)

369 Centaurea congesta (369): (0.000-0.900)

372 Centaurea incanescens (372): (0.000-0.800)

373 Centaurea karduchorum (373): (0.000-0.833)

374 Centaurea lanigera (374): (0.000-0.462)

376 Centaurea nigrofimbria (376): (0.000-0.750)

379 Centaurea pulcherrima (379): (0.000-0.818)

387 Cephalaria sparsipilosa (387): (0.000-0.750)

388 Cerastium araraticum (388): (0.000-0.875)

389 Cerastium gnaphalodes (389): (0.000-0.500)

392 Cerastium pseudokasbek (392): (0.000-0.750)

394 Cerastium szowitsii (394): (0.000-1.000)

403 Chamaesciadium acaule (403): (0.000-0.559)

408 Cicerbita adenophora (408): (0.000-0.833)

413 Cirsium pseudobracteosum (413): (0.000-0.833)

415 Cirsium simplex (415): (0.000-0.682)

416 Cirsium tomentosum (416): (0.000-0.667)

419 Cochlearia aucheri (419): (0.000-0.615)

420 Cochlearia sintenisii (420): (0.000-0.667)

421 Colchicum kurdicum (421): (0.000-0.833)

422 Colpodium araraticum (422): (0.000-0.567)

423 Colpodium fibrosum (423): (0.000-0.778)

424 Colpodium gillettii (424): (0.000-0.750)

430 Corydalis persica (430): (0.000-0.500)

454 Crepis armena (454): (0.000-0.769)

456 Crepis bupleurifolia (456): (0.000-0.708)

458 Crepis conyzifolia (458): (0.000-0.750)

460 Crepis dioritica (460): (0.000-0.688)

464 Crepis sahendi (464): (0.000-0.632)

467 Crocus kotschyanus (467): (0.000-0.654)

469 Cymbocarpum erythraeum (469): (0.000-0.750)

470 Daphne magakjanii (470): (0.000-0.750)

471 Daphne oleoides (471): (0.000-0.524)

472 Delphinium carduchorum (472): (0.000-0.833)

477 Delphinium linearilobum (477): (0.000-0.818)

490 Dianthus multicaulis (490): (0.000-0.600)

491 Dianthus muschianus (491): (0.000-0.688)

492 Dianthus raddeanus (492): (0.000-0.750)

495 Dianthus seidlitzii (495): (0.000-0.700)

528 Draba araratica (528): (0.000-0.786)

532 Draba polytricha (532): (0.000-0.818)

556 Eryngium bornmuelleri (556): (0.000-0.833)

562 Erysimum gelidum (562): (0.000-0.500)

569 Erysimum munzuriense (569): (0.000-0.667)

573 Erysimum sintenisianum (573): (0.000-0.667)

577 Euphorbia grisophylla (577): (0.000-0.875)

584 Euphorbia sahendi (584): (0.000-0.700)

585 Euphorbia sanasunitensis (585): (0.000-0.833)

589 Euphrasia sevanensis (589): (0.000-0.474)

590 Ferula haussknechtii (590): (0.000-0.833)

593 Ferula setifolia (593): (0.000-0.625)

598 Festuca artvinensis (598): (0.000-0.688)

599 Festuca brunnescens (599): (0.000-0.700)

600 Festuca bushiana (600): (0.000-0.667)

603 Festuca chalcophaea (603): (0.000-0.529)

605 Festuca elwendiana (605): (0.000-0.500)

611 Festuca skvortsovii (611): (0.000-0.700)

614 Fritillaria alburyana (614): (0.000-0.571)

620 Fritillaria latifolia (620): (0.000-0.800)

621 Fritillaria michailovskyi (621): (0.000-0.643)

623 Fritillaria minuta (623): (0.000-0.692)

627 Fuernrohria setifolia (627): (0.000-0.875)

631 Gagea joannis (631): (0.000-1.000)

641 Galium majmechense (641): (0.000-0.875)

642 Galium nabelekii (642): (0.000-0.813)

650 Gentiana gelida (650): (0.000-0.600)

653 Geranium kurdicum (653): (0.000-0.833)

661 Gypsophila adenophylla (661): (0.000-0.833)

663 Gypsophila briquetiana (663): (0.000-0.667)

666 Gypsophila lipskyi (666): (0.000-0.750)

667 Gypsophila nabelaelekii (667): (0.000-0.667)

673 Hedysarum erythroleucum (673): (0.000-0.474)

676 Heldreichia bupleurifolia (676): (0.000-0.722)

685 Helictotrichon argaeum (685): (0.000-0.500)

687 Heracleum crenatifolium (687): (0.000-0.800)

690 Heracleum rawianum (690): (0.000-0.667)

691 Heracleum schelkovnikovii (691): (0.000-0.875)

693 Herniaria argaea (693): (0.000-0.667)

708 Inula mariae (708): (0.000-0.700)

719 Juncus filiformis (719): (0.000-0.667)

730 Lamium armenum (730): (0.000-0.917)

731 Lamium crinitum (731): (0.000-0.563)

734 Laserpitium carduchorum (734): (0.000-0.833)

737 Lathyrus cyaneus (737): (0.000-0.667)

738 Lathyrus nivalis (738): (0.000-0.750)

748 Leutea rechingeri (748): (0.000-0.700)

753 Linaria schelkownikowii (753): (0.000-0.875)

755 Linum densiflorum (755): (0.000-0.688)

757 Linum meletonis (757): (0.000-0.800)

758 Linum obtusatum (758): (0.000-0.591)

759 Linum punctatum (759): (0.000-0.833)

760 Linum subbiflorum (760): (0.000-1.000)

761 Linum triflorum (761): (0.000-0.700)

765 Malabaila dasyantha (765): (0.000-1.000)

767 Marrubium cordatum (767): (0.000-0.917)

786 Muscari coeleste (786): (0.000-0.682)

794 Nepeta alaghezi (794): (0.000-0.875)

818 Nepeta sahandica (818): (0.000-0.800)

821 Nonea macrantha (821): (0.000-0.813)

829 Onobrychis transcaucasica (829): (0.000-0.643)

832 Onosma haussknechtii (832): (0.000-0.750)

837 Onosma proballanthera (837): (0.000-0.700)

838 Onosma sabalanica (838): (0.000-0.700)

856 Oxytropis lazica (856): (0.000-0.654)

906 Poa longifolia (906): (0.000-0.625)

924 Potentilla geranioides (924): (0.000-0.679)

943 Psephellus transcaucasicus (943): (0.000-0.875)

955 Ranunculus aragazi (955): (0.000-0.875)

965 Ranunculus diversifolius (965): (0.000-0.500)

968 Ranunculus fenzlii (968): (0.000-0.567)

976 Ranunculus tempskyanus (976): (0.000-0.667)

978 Ranunculus transcaucasicus (978): (0.000-0.667)

987 Rhynchocorys kurdica (987): (0.000-0.800)

989 Ricotia aucheri (989): (0.000-0.833)

992 Rindera caespitosa (992): (0.000-0.800)

993 Rosularia aizoon (993): (0.000-0.500)

998 Rosularia rechingeri (998): (0.000-0.833)

1003 Rumex acetoselloides (1003): (0.000-1.000)

1007 Rumex ponticus (1007): (0.000-0.567)

1010 Salvia pachystachya (1010): (0.000-0.722)

1011 Salvia sahendica (1011): (0.000-0.700)

1033 Scorzonera sericea (1033): (0.000-0.833)

1039 Scrophularia catariifolia (1039): (0.000-0.500)

1040 Scrophularia chlorantha (1040): (0.000-0.714)

1069 Sempervivum atropatanum (1069): (0.000-0.800)

1073 Sempervivum transcaucasicum (1073): (0.000-1.000)

1075 Senecio cilicius (1075): (0.000-0.682)

1076 Senecio davisii (1076): (0.000-0.875)

1078 Senecio eriospermus (1078): (0.000-0.750)

1085 Senecio taraxacifolius (1085): (0.000-0.611)

1094 Silene araratica (1094): (0.000-1.000)

1100 Silene cartilaginea (1100): (0.000-0.833)

1101 Silene caryophylloides (1101): (0.000-0.667)

1108 Silene dianthoides (1108): (0.000-0.407)

1117 Silene lasiantha (1117): (0.000-0.472)

1119 Silene lucida (1119): (0.000-0.813)

1136 Sorbus tamamschjanae (1136): (0.000-0.722)

1139 Stachys balansae (1139): (0.000-0.833)

1159 Tanacetum mucroniferum (1159): (0.000-0.750)

1160 Tanacetum nitens (1160): (0.000-0.567)

1161 Tanacetum nivale (1161): (0.000-0.833)

1165 Tanacetum zahlbruckneri (1165): (0.000-0.813)

1175 Thlaspi kurdicum (1175): (0.000-0.667)

1180 Thlaspi sintenisii (1180): (0.000-0.667)

1182 Thlaspi tenue (1182): (0.000-0.500)

1183 Thlaspi valerianoides (1183): (0.000-0.833)

1189 Thymus fedtschenkoi (1189): (0.000-0.667)

1201 Tragopogon reticulatus (1201): (0.000-0.700)

1203 Trifolium longidentatum (1203): (0.000-1.000)

1204 Trifolium montanum (1204): (0.000-0.750)

1206 Trifolium sintenisii (1206): (0.000-0.667)

1207 Tripleurospermum melanolepis (1207): (0.000-0.750)

1210 Trisetum turcicum (1210): (0.000-0.577)

1214 Valeriana alpestris (1214): (0.000-0.643)

1229 Veronica armena (1229): (0.000-0.773)

1256 Veronica telephiifolia (1256): (0.000-0.636)

1. **Alborz AE:**

5 Acantholimon demavendicum (5): (0.000-0.600)

10 Acantholimon hohenackeri (10): (0.000-0.571)

23 Achillea aucheri (23): (0.000-0.682)

26 Achillea millefolium (26): (0.000-0.800)

35 Aethionema semnanensis (35): (0.000-0.833)

44 Alchemilla citrina (44): (0.000-0.800)

48 Alchemilla fluminea (48): (0.000-0.636)

49 Alchemilla gigantodus (49): (0.000-0.667)

51 Alchemilla hessii (51): (0.000-0.500)

55 Alchemilla melancholica (55): (0.000-0.722)

56 Alchemilla microscopica (56): (0.000-0.667)

60 Alchemilla pectiniloba (60): (0.000-0.833)

62 Alchemilla plicatissima (62): (0.000-0.750)

71 Allium alamutense (71): (0.000-0.357)

82 Allium derderianum (82): (0.000-0.769)

87 Allium elburzense (87): (0.000-0.800)

115 Alyssopsis mollis (115): (0.000-0.615)

124 Alyssum polycladum (124): (0.000-0.472)

137 Arabis rimarum (137): (0.000-0.500)

155 Artemisia melanolepis (155): (0.000-0.591)

167 Astragalus aestivorum (167): (0.000-0.833)

173 Astragalus atricapillus (173): (0.000-0.500)

179 Astragalus beckii (179): (0.000-0.692)

180 Astragalus bounophilus (180): (0.000-0.625)

182 Astragalus capax (182): (0.000-0.833)

183 Astragalus capito (183): (0.000-1.000)

188 Astragalus chrysanthus (188): (0.000-1.000)

190 Astragalus confusus (190): (0.000-0.563)

195 Astragalus demavendicus (195): (0.000-0.900)

231 Astragalus jodotropis (231): (0.000-0.800)

242 Astragalus macrosemius (242): (0.000-0.500)

248 Astragalus modestus (248): (0.000-0.800)

249 Astragalus monanthemus (249): (0.000-0.833)

256 Astragalus nezva-montis (256): (0.000-0.667)

261 Astragalus patrius (261): (0.000-0.447)

265 Astragalus perdurans (265): (0.000-0.500)

269 Astragalus platysematus (269): (0.000-0.833)

281 Astragalus rubriflorus (281): (0.000-1.000)

323 Betonica nivea (323): (0.000-1.000)

327 Bromus confinis (327): (0.000-0.667)

329 Bufonia koelzii (329): (0.000-0.600)

348 Campanula lourica (348): (0.000-0.600)

366 Catabrosa aquatica (366): (0.000-0.412)

371 Centaurea elbrusensis (371): (0.000-0.654)

418 Clastopus vestitus (418): (0.000-0.433)

443 Cousinia gmelini (443): (0.000-1.000)

449 Cousinia pterocaulos (449): (0.000-0.650)

451 Cousinia shahvarica (451): (0.000-0.500)

455 Crepis asadbarensis (455): (0.000-1.000)

459 Crepis demavendi (459): (0.000-0.833)

461 Crepis elbursensis (461): (0.000-0.688)

473 Delphinium elbursense (473): (0.000-0.588)

520 Diplotaenia damavandica (520): (0.000-0.833)

521 Dolichorrhiza persica (521): (0.000-1.000)

533 Draba pulchella (533): (0.000-0.542)

535 Dracocephalum aucheri (535): (0.000-0.607)

543 Elburzia fenestrata (543): (0.000-0.800)

550 Erigeron hyrcanicus (550): (0.000-0.600)

554 Erodium dimorphum (554): (0.000-0.667)

558 Erysimum caespitosum (558): (0.000-0.548)

588 Euphrasia juzepczukii (588): (0.000-0.440)

619 Fritillaria kotschyana (619): (0.000-0.722)

629 Gagea caroli-kochii (629): (0.000-0.500)

636 Galium aucheri (636): (0.000-0.667)

638 Galium decumbens (638): (0.000-0.583)

640 Galium hyrcanicum (640): (0.000-0.520)

660 Graellsia stylosa (660): (0.000-0.833)

683 Helichrysum psychrophilum (683): (0.000-0.595)

686 Heracleum anisactis (686): (0.000-0.750)

702 Hyoscyamus kurdicus (702): (0.000-0.550)

709 Iranecio elbrusensis (709): (0.000-0.778)

710 Iranecio oligolepis (710): (0.000-0.833)

712 Iris barnumiae (712): (0.000-0.750)

722 Jurinella frigida (722): (0.000-0.833)

728 Lagochilus kotschyanus (728): (0.000-0.500)

741 Leonurus cardiaca (741): (0.000-0.833)

743 Lepechiniella persica (743): (0.000-0.500)

750 Ligularia persica (750): (0.000-0.833)

810 Nepeta menthoides (810): (0.000-0.650)

852 Oxytropis Iranica (852): (0.000-0.833)

870 Papaver armeniacum (870): (0.000-0.386)

881 Paraquilegia caespitosa (881): (0.000-0.833)

895 Phleum iranicum (895): (0.000-0.800)

911 Polylophium involucratum (911): (0.000-1.000)

927 Potentilla mallota (927): (0.000-0.500)

932 Potentilla polyschista (932): (0.000-0.531)

959 Ranunculus bulbilliferus (959): (0.000-0.550)

1019 Saxifraga wendelboi (1019): (0.000-0.833)

1022 Scorzonera grossheimii (1022): (0.000-0.467)

1026 Scorzonera meyeri (1026): (0.000-0.463)

1041 Scrophularia crassicaulis (1041): (0.000-0.833)

1053 Scutellaria glechomoides (1053): (0.000-0.800)

1071 Sempervivum iranicum (1071): (0.000-0.889)

1086 Senecio vulcanicus (1086): (0.000-0.800)

1120 Silene marschallii (1120): (0.000-0.478)

1121 Silene meyeri (1121): (0.000-0.448)

1148 Stellaria persica (1148): (0.000-0.600)

1152 Stenotaenia nudicaulis (1152): (0.000-0.727)

1157 Tanacetum hololeucum (1157): (0.000-0.833)

1167 Taraxacum neospurium (1167): (0.000-0.625)

1181 Thlaspi stenocarpum (1181): (0.000-0.722)

1197 Trachydium pauciradiatum (1197): (0.000-0.667)

1200 Tragopogon kotschyi (1200): (0.000-1.000)

1230 Veronica aucheri (1230): (0.000-0.722)

1246 Veronica mirabilis (1246): (0.000-0.833)

1249 Veronica paederotae (1249): (0.000-1.000)

1252 Veronica rechingeri (1252): (0.000-0.917)

1264 Viola spathulata (1264): (0.000-1.000)

1. **Zagros AE:**

2 Acantholimon brachystachyum (2): (0.000-0.467)

8 Acantholimon eschkerense (8): (0.000-0.667)

14 Acantholimon nigricans (14): (0.000-0.500)

15 Acantholimon oliganthum (15): (0.000-0.750)

17 Acantholimon scabrellum (17): (0.000-1.000)

19 Acantholimon tomentellum (19): (0.000-0.875)

39 Aethionema umbellatum (39): (0.000-0.833)

76 Allium austroiranicum (76): (0.000-0.435)

94 Allium longivaginatum (94): (0.000-0.875)

123 Alyssum persicum (123): (0.000-0.875)

151 Arenaria persica (151): (0.000-0.643)

159 Asperula fragillima (159): (0.000-0.800)

162 Asperula rechingeri (162): (0.000-0.875)

186 Astragalus chartostegius (186): (0.000-0.875)

204 Astragalus exspectatus (204): (0.000-0.700)

212 Astragalus ghashghaicus (212): (0.000-0.750)

229 Astragalus inexpectatus (229): (0.000-0.750)

232 Astragalus johannis (232): (0.000-0.625)

247 Astragalus melanodon (247): (0.000-0.700)

253 Astragalus murinus (253): (0.000-0.667)

291 Astragalus stenostegius (291): (0.000-0.700)

322 Bellevalia tristis (322): (0.000-0.875)

346 Campanula humillima (346): (0.000-0.929)

349 Campanula luristanica (349): (0.000-0.875)

401 Chaerophyllum nivale (401): (0.000-0.750)

434 Cousinia archibaldii (434): (0.000-0.875)

504 Dionysia caespitosa (504): (0.000-0.900)

505 Dionysia cristagalli (505): (0.000-0.750)

583 Euphorbia plebeia (583): (0.000-0.667)

595 Ferulago contracta (595): (0.000-0.382)

625 Fritillaria reuteri (625): (0.000-0.571)

678 Helichrysum athanaton (678): (0.000-0.625)

698 Hesperis nivalis (698): (0.000-0.875)

720 Jurinea meda (720): (0.000-0.545)

725 Kelussia odoratissima (725): (0.000-0.750)

773 Minuartia aucheriana (773): (0.000-0.875)

790 Myopordon persicum (790): (0.000-0.750)

801 Nepeta chionophila (801): (0.000-0.750)

813 Nepeta oxyodonta (813): (0.000-0.433)

819 Nepeta sessilifolia (819): (0.000-0.563)

833 Onosma kilouyensis (833): (0.000-0.471)

899 Pimpinella deverroides (899): (0.000-0.600)

922 Potentilla elvendensis (922): (0.000-0.750)

926 Potentilla lignosa (926): (0.000-0.500)

966 Ranunculus elymaiticus (966): (0.000-0.800)

972 Ranunculus pichleri (972): (0.000-0.500)

975 Ranunculus straussii (975): (0.000-0.750)

977 Ranunculus termei (977): (0.000-0.700)

984 Rhabdosciadium aucheri (984): (0.000-0.650)

1002 Rubia pauciflora (1002): (0.000-0.591)

1028 Scorzonera psychrophila (1028): (0.000-0.667)

1035 Scorzonera subaphylla (1035): (0.000-0.750)

1042 Scrophularia crassiuscula (1042): (0.000-0.900)

1064 Semenovia dichotoma (1064): (0.000-0.750)

1065 Semenovia frigida (1065): (0.000-1.000)

1099 Silene caroli-henrici (1099): (0.000-0.750)

1129 Silene persica (1129): (0.000-0.875)

1130 Silene pseudonurensis (1130): (0.000-0.625)

1143 Stachys obtusicrena (1143): (0.000-0.625)

1144 Stachys pilifera (1144): (0.000-0.750)

1156 Tanacetum dumosum (1156): (0.000-0.591)

1196 Trachydium kotschyi (1196): (0.000-0.500)

1238 Veronica fragilis (1238): (0.000-0.750)

1. **Yazd-Kerman AE:**

11 Acantholimon kermanense (11): (0.000-0.786)

13 Acantholimon modestum (13): (0.000-0.786)

18 Acantholimon sirchense (18): (0.000-0.750)

21 Acantholimon zaeifii (21): (0.000-0.688)

81 Allium cathodicarpum (81): (0.000-0.333)

93 Allium lalesaricum (93): (0.000-0.750)

143 Arenaria bulica (143): (0.000-0.643)

164 Astragalus abditus (164): (0.000-0.409)

192 Astragalus daenaensis (192): (0.000-0.611)

219 Astragalus heterodoxus (219): (0.000-0.750)

277 Astragalus pseudoshebarensis (277): (0.000-0.300)

284 Astragalus rufescens (284): (0.000-0.545)

297 Astragalus tenuiscapus (297): (0.000-0.643)

396 Chaenorhinum grossecostatum (396): (0.000-0.714)

452 Cousinia sicigera (452): (0.000-0.643)

514 Dionysia rhaptodes (514): (0.000-0.500)

539 Dracocephalum polychaetum (539): (0.000-0.750)

565 Erysimum hezarense (565): (0.000-0.750)

571 Erysimum polatschekii (571): (0.000-0.750)

797 Nepeta assurgens (797): (0.000-0.750)

800 Nepeta bornmuelleri (800): (0.000-0.750)

828 Onobrychis plantago (828): (0.000-0.643)

967 Ranunculus eriorrhizus (967): (0.000-0.786)

971 Ranunculus papyrocarpus (971): (0.000-0.556)

1084 Senecio subnivalis (1084): (0.000-0.750)

1105 Silene daenensis (1105): (0.000-0.500)

1221 Verbascum carmanicum (1221): (0.000-0.875)

**Table S4** Species contributing to the bioregions using total alpine species dataset and Network-Clustering approach.

| **Bioregions** | **Taurus** | **Anatolian-Armenian** | **Alborz** | **Zagros** | **Yazd-Kerman** | Transitional Zones | | | |
| --- | --- | --- | --- | --- | --- | --- | --- | --- | --- |
|  | **1** | **2** | **3** | **4** | **5** | 6 | 8 | 9 | 10 |
| Acantholimon albocalycinum | 0 | 0 | 0 | 0 | 1 | 0 | 0 | 0 | 0 |
| Acantholimon artosense | 0 | 1 | 0 | 0 | 0 | 0 | 0 | 0 | 0 |
| Acantholimon brachystachyum | 0 | 0 | 1 | 1 | 0 | 0 | 0 | 0 | 0 |
| Acantholimon calvertii | 1 | 1 | 0 | 0 | 0 | 0 | 0 | 0 | 0 |
| Acantholimon cupreo-olivascens | 0 | 0 | 0 | 0 | 1 | 0 | 0 | 0 | 0 |
| Acantholimon demavendicum | 0 | 0 | 1 | 0 | 0 | 0 | 0 | 0 | 0 |
| Acantholimon dianthifolium | 0 | 1 | 0 | 0 | 0 | 0 | 0 | 0 | 0 |
| Acantholimon ekimii | 0 | 1 | 0 | 0 | 0 | 0 | 0 | 0 | 0 |
| Acantholimon erinaceum | 0 | 1 | 1 | 1 | 0 | 0 | 0 | 0 | 0 |
| Acantholimon eschkerense | 0 | 0 | 0 | 1 | 0 | 0 | 0 | 0 | 0 |
| Acantholimon haesarensis | 0 | 0 | 0 | 0 | 1 | 0 | 0 | 0 | 0 |
| Acantholimon hohenackeri | 0 | 1 | 1 | 0 | 0 | 1 | 0 | 0 | 0 |
| Acantholimon kermanense | 0 | 0 | 0 | 1 | 1 | 0 | 0 | 0 | 0 |
| Acantholimon mirtadzadinii | 0 | 0 | 0 | 0 | 1 | 0 | 0 | 0 | 0 |
| Acantholimon modestum | 0 | 0 | 0 | 1 | 1 | 0 | 0 | 0 | 0 |
| Acantholimon nigricans | 0 | 0 | 0 | 1 | 0 | 0 | 0 | 0 | 0 |
| Acantholimon oliganthum | 0 | 0 | 0 | 1 | 0 | 0 | 0 | 0 | 0 |
| Acantholimon sahendicum | 0 | 1 | 0 | 0 | 0 | 1 | 0 | 0 | 0 |
| Acantholimon scabrellum | 0 | 0 | 0 | 1 | 0 | 0 | 0 | 0 | 0 |
| Acantholimon sirchense | 0 | 0 | 0 | 0 | 1 | 0 | 0 | 0 | 0 |
| Acantholimon tomentellum | 0 | 0 | 0 | 1 | 0 | 0 | 0 | 0 | 0 |
| Acantholimon ulicinum | 1 | 1 | 0 | 0 | 0 | 0 | 0 | 0 | 0 |
| Acantholimon zaeifii | 0 | 0 | 0 | 0 | 1 | 0 | 0 | 0 | 0 |
| Achillea armenorum | 1 | 0 | 0 | 0 | 0 | 0 | 0 | 0 | 0 |
| Achillea aucheri | 0 | 0 | 1 | 0 | 0 | 1 | 0 | 0 | 0 |
| Achillea kellalensis | 0 | 0 | 0 | 1 | 0 | 0 | 0 | 0 | 0 |
| Achillea latiloba | 0 | 1 | 0 | 0 | 0 | 0 | 0 | 0 | 0 |
| Achillea millefolium | 0 | 0 | 1 | 0 | 0 | 0 | 0 | 0 | 0 |
| Achillea vermicularis | 0 | 1 | 1 | 1 | 0 | 1 | 1 | 0 | 0 |
| Aconitum anthora | 0 | 1 | 0 | 0 | 0 | 0 | 0 | 0 | 0 |
| Aconitum cochleare | 0 | 1 | 0 | 0 | 0 | 1 | 0 | 0 | 0 |
| Aethionema caespitosum | 0 | 1 | 0 | 0 | 0 | 0 | 0 | 0 | 0 |
| Aethionema fimbriatum | 0 | 1 | 1 | 1 | 0 | 1 | 1 | 0 | 0 |
| Aethionema munzurense | 0 | 1 | 0 | 0 | 0 | 0 | 0 | 0 | 0 |
| Aethionema oppositifolium | 1 | 1 | 0 | 0 | 0 | 0 | 0 | 0 | 1 |
| Aethionema papillosum | 1 | 0 | 0 | 0 | 0 | 0 | 0 | 0 | 0 |
| Aethionema rotundifolium | 0 | 1 | 0 | 0 | 0 | 0 | 0 | 0 | 0 |
| Aethionema semnanensis | 0 | 0 | 1 | 0 | 0 | 0 | 0 | 0 | 0 |
| Aethionema speciosum | 1 | 1 | 0 | 0 | 0 | 0 | 0 | 0 | 0 |
| Aethionema stenopterum | 0 | 0 | 1 | 1 | 0 | 0 | 0 | 0 | 0 |
| Aethionema subulatum | 1 | 0 | 0 | 0 | 0 | 0 | 0 | 0 | 0 |
| Aethionema trinervium | 0 | 1 | 1 | 1 | 0 | 1 | 1 | 0 | 0 |
| Aethionema umbellatum | 0 | 0 | 0 | 1 | 0 | 0 | 0 | 0 | 0 |
| Aethionema virgatum | 0 | 1 | 1 | 1 | 0 | 1 | 0 | 0 | 0 |
| Agropyron canaliculatum | 0 | 0 | 1 | 0 | 0 | 0 | 0 | 0 | 0 |
| Agropyron cognatum | 0 | 0 | 0 | 1 | 0 | 0 | 1 | 0 | 0 |
| Agropyron imbricatum | 0 | 1 | 1 | 0 | 0 | 1 | 1 | 0 | 0 |
| Agrostis canina | 0 | 0 | 0 | 0 | 0 | 1 | 0 | 0 | 0 |
| Agrostis lazica | 1 | 1 | 0 | 0 | 0 | 0 | 0 | 0 | 0 |
| Agrostis olympica | 1 | 1 | 1 | 1 | 1 | 1 | 1 | 0 | 0 |
| Agrostis vinealis | 0 | 1 | 0 | 0 | 0 | 1 | 0 | 0 | 0 |
| Alchemilla amardica | 0 | 0 | 1 | 0 | 0 | 0 | 0 | 0 | 0 |
| Alchemilla basakii | 0 | 1 | 0 | 0 | 0 | 0 | 0 | 0 | 0 |
| Alchemilla caucasica | 0 | 1 | 0 | 0 | 0 | 0 | 0 | 0 | 1 |
| Alchemilla ciminensis | 0 | 1 | 0 | 0 | 0 | 0 | 0 | 0 | 0 |
| Alchemilla citrina | 0 | 0 | 1 | 0 | 0 | 1 | 0 | 0 | 0 |
| Alchemilla compactilis | 1 | 1 | 1 | 0 | 0 | 0 | 0 | 0 | 0 |
| Alchemilla dura | 0 | 1 | 0 | 0 | 0 | 0 | 0 | 0 | 0 |
| Alchemilla ellenbergiana | 1 | 1 | 0 | 0 | 0 | 0 | 0 | 0 | 0 |
| Alchemilla erythropoda | 1 | 1 | 1 | 0 | 0 | 0 | 0 | 0 | 0 |
| Alchemilla erzincanensis | 0 | 1 | 0 | 0 | 0 | 0 | 0 | 0 | 0 |
| Alchemilla farinosa | 0 | 0 | 1 | 0 | 0 | 1 | 0 | 0 | 0 |
| Alchemilla fluminea | 0 | 0 | 1 | 0 | 0 | 1 | 0 | 0 | 0 |
| Alchemilla gigantodus | 0 | 0 | 1 | 0 | 0 | 0 | 0 | 0 | 0 |
| Alchemilla grossheimii | 0 | 1 | 0 | 0 | 0 | 0 | 0 | 0 | 0 |
| Alchemilla hemsinica | 0 | 1 | 0 | 0 | 0 | 0 | 0 | 0 | 0 |
| Alchemilla hessii | 0 | 1 | 1 | 0 | 0 | 1 | 0 | 0 | 0 |
| Alchemilla heterophylla | 1 | 1 | 0 | 0 | 0 | 0 | 0 | 0 | 0 |
| Alchemilla kackarensis | 0 | 1 | 0 | 0 | 0 | 0 | 0 | 0 | 0 |
| Alchemilla kurdica | 0 | 0 | 1 | 1 | 0 | 0 | 1 | 0 | 0 |
| Alchemilla mazandarana | 0 | 0 | 1 | 0 | 0 | 0 | 0 | 0 | 0 |
| Alchemilla melancholica | 0 | 0 | 1 | 0 | 0 | 0 | 0 | 0 | 0 |
| Alchemilla microscopica | 0 | 0 | 1 | 0 | 0 | 0 | 0 | 0 | 0 |
| Alchemilla minusculiflora | 0 | 1 | 0 | 0 | 0 | 0 | 0 | 0 | 0 |
| Alchemilla oriturcica | 0 | 1 | 0 | 0 | 0 | 0 | 0 | 0 | 0 |
| Alchemilla ovitensis | 0 | 1 | 0 | 0 | 0 | 0 | 0 | 0 | 0 |
| Alchemilla paracompactilis | 1 | 0 | 0 | 0 | 0 | 0 | 0 | 0 | 0 |
| Alchemilla pectiniloba | 0 | 0 | 1 | 0 | 0 | 0 | 0 | 0 | 0 |
| Alchemilla persica | 0 | 1 | 1 | 1 | 0 | 1 | 1 | 0 | 0 |
| Alchemilla plicatissima | 0 | 0 | 1 | 0 | 0 | 0 | 0 | 0 | 0 |
| Alchemilla plicatula | 0 | 0 | 0 | 0 | 0 | 0 | 0 | 0 | 1 |
| Alchemilla procerrima | 0 | 1 | 0 | 0 | 0 | 0 | 0 | 0 | 0 |
| Alchemilla pseudocartalinica | 1 | 1 | 0 | 0 | 0 | 0 | 0 | 0 | 1 |
| Alchemilla raddeana | 0 | 1 | 0 | 0 | 0 | 0 | 0 | 0 | 0 |
| Alchemilla retinervis | 1 | 1 | 0 | 0 | 0 | 0 | 0 | 0 | 0 |
| Alchemilla rivularis | 1 | 0 | 0 | 0 | 0 | 0 | 0 | 0 | 0 |
| Alchemilla rizensis | 0 | 1 | 0 | 0 | 0 | 0 | 0 | 0 | 0 |
| Alchemilla sedelmeyeriana | 0 | 1 | 0 | 0 | 0 | 0 | 0 | 0 | 0 |
| Alchemilla sericata | 0 | 1 | 0 | 0 | 0 | 0 | 0 | 0 | 0 |
| Alchemilla sericea | 0 | 1 | 0 | 0 | 0 | 0 | 0 | 0 | 0 |
| Alchemilla straminea | 0 | 1 | 0 | 0 | 0 | 0 | 0 | 0 | 0 |
| Alchemilla surculosa | 0 | 1 | 1 | 0 | 0 | 0 | 0 | 0 | 0 |
| Alchemilla tiryalensis | 0 | 1 | 0 | 0 | 0 | 0 | 0 | 0 | 0 |
| Alchemilla venosa | 0 | 1 | 0 | 0 | 0 | 0 | 0 | 0 | 0 |
| Alkanna bracteosa | 0 | 1 | 1 | 1 | 0 | 1 | 0 | 0 | 0 |
| Alkanna frigida | 0 | 0 | 1 | 1 | 0 | 0 | 0 | 0 | 0 |
| Allium akaka | 0 | 1 | 1 | 0 | 0 | 1 | 0 | 0 | 0 |
| Allium alamutense | 0 | 0 | 1 | 0 | 0 | 0 | 0 | 0 | 0 |
| Allium alpinarii | 1 | 0 | 0 | 0 | 0 | 0 | 0 | 0 | 0 |
| Allium anacoleum | 0 | 1 | 0 | 0 | 0 | 0 | 0 | 0 | 0 |
| Allium arlgirdense | 0 | 1 | 0 | 0 | 0 | 0 | 0 | 0 | 0 |
| Allium aucheri | 0 | 1 | 0 | 0 | 0 | 0 | 0 | 0 | 0 |
| Allium austroiranicum | 0 | 0 | 0 | 1 | 0 | 0 | 0 | 0 | 0 |
| Allium balansae | 0 | 1 | 0 | 0 | 0 | 0 | 0 | 0 | 1 |
| Allium brachyodon | 0 | 0 | 1 | 1 | 0 | 0 | 0 | 0 | 0 |
| Allium breviscapum | 0 | 0 | 0 | 1 | 0 | 0 | 0 | 1 | 0 |
| Allium capitellatum | 0 | 0 | 1 | 1 | 0 | 0 | 0 | 0 | 0 |
| Allium cathodicarpum | 0 | 0 | 0 | 1 | 1 | 0 | 0 | 0 | 0 |
| Allium derderianum | 0 | 1 | 1 | 0 | 0 | 0 | 0 | 0 | 0 |
| Allium djimilense | 0 | 1 | 0 | 0 | 0 | 0 | 0 | 0 | 0 |
| Allium dumanii | 1 | 0 | 0 | 0 | 0 | 0 | 0 | 0 | 0 |
| Allium egorovae | 0 | 1 | 0 | 0 | 0 | 1 | 0 | 0 | 0 |
| Allium elburzense | 0 | 0 | 1 | 0 | 0 | 0 | 0 | 0 | 0 |
| Allium flavum | 0 | 0 | 0 | 0 | 0 | 0 | 0 | 0 | 1 |
| Allium hoshabicum | 0 | 1 | 0 | 0 | 0 | 0 | 0 | 0 | 0 |
| Allium hymenorhizum | 0 | 0 | 1 | 0 | 0 | 0 | 0 | 0 | 0 |
| Allium iranshahrii | 0 | 1 | 0 | 0 | 0 | 0 | 1 | 0 | 0 |
| Allium kuhrangense | 0 | 0 | 0 | 1 | 0 | 0 | 0 | 0 | 0 |
| Allium kunthianum | 0 | 1 | 0 | 0 | 0 | 1 | 0 | 0 | 0 |
| Allium lalesaricum | 0 | 0 | 0 | 0 | 1 | 0 | 0 | 0 | 0 |
| Allium longivaginatum | 0 | 0 | 0 | 1 | 0 | 0 | 0 | 0 | 0 |
| Allium mahneshanense | 0 | 0 | 0 | 0 | 0 | 1 | 0 | 1 | 0 |
| Allium microspathum | 0 | 1 | 0 | 0 | 0 | 0 | 0 | 0 | 0 |
| Allium montelburzense | 0 | 0 | 1 | 0 | 0 | 0 | 0 | 0 | 0 |
| Allium oreophilum | 0 | 1 | 0 | 0 | 0 | 0 | 0 | 0 | 0 |
| Allium pseudoampeloprasum | 0 | 1 | 0 | 0 | 0 | 0 | 0 | 0 | 0 |
| Allium pseudostrictum | 0 | 1 | 0 | 0 | 0 | 0 | 0 | 0 | 0 |
| Allium rhetoreanum | 0 | 1 | 0 | 0 | 0 | 0 | 0 | 0 | 0 |
| Allium sabalense | 0 | 0 | 1 | 0 | 0 | 1 | 0 | 0 | 0 |
| Allium sahandicum | 0 | 0 | 0 | 0 | 0 | 1 | 0 | 0 | 0 |
| Allium schoenoprasum | 1 | 1 | 1 | 0 | 0 | 1 | 0 | 0 | 0 |
| Allium scotostemon | 0 | 0 | 1 | 0 | 0 | 0 | 0 | 0 | 0 |
| Allium shatakiense | 0 | 1 | 0 | 0 | 0 | 0 | 0 | 0 | 0 |
| Allium stearnianum | 0 | 1 | 0 | 0 | 0 | 0 | 0 | 0 | 0 |
| Allium straussii | 0 | 0 | 0 | 1 | 0 | 0 | 0 | 0 | 0 |
| Allium szovitsii | 0 | 1 | 0 | 0 | 0 | 0 | 0 | 0 | 0 |
| Allium talyschense | 0 | 1 | 0 | 0 | 0 | 0 | 0 | 0 | 0 |
| Allium tauricola | 1 | 1 | 0 | 0 | 0 | 0 | 0 | 0 | 0 |
| Allium tuchalense | 0 | 0 | 1 | 0 | 0 | 0 | 0 | 0 | 0 |
| Alopecurus apiatus | 0 | 1 | 1 | 0 | 0 | 0 | 0 | 0 | 0 |
| Alopecurus aucheri | 0 | 1 | 1 | 0 | 0 | 1 | 0 | 0 | 0 |
| Alopecurus dasyanthus | 0 | 1 | 0 | 0 | 0 | 1 | 0 | 0 | 0 |
| Alopecurus glacialis | 0 | 1 | 0 | 0 | 0 | 0 | 0 | 0 | 0 |
| Alopecurus himalaicus | 0 | 0 | 1 | 0 | 0 | 0 | 0 | 0 | 0 |
| Alopecurus laguroides | 0 | 1 | 0 | 0 | 0 | 0 | 0 | 0 | 0 |
| Alopecurus lanatus | 1 | 0 | 0 | 0 | 0 | 0 | 0 | 0 | 0 |
| Alopecurus seravschanicus | 0 | 0 | 1 | 0 | 0 | 0 | 0 | 0 | 0 |
| Alopecurus textilis | 1 | 1 | 1 | 1 | 0 | 1 | 1 | 0 | 0 |
| Alopecurus vaginatus | 1 | 1 | 1 | 0 | 0 | 1 | 1 | 0 | 0 |
| Alyssopsis mollis | 0 | 0 | 1 | 0 | 0 | 1 | 0 | 0 | 0 |
| Alyssum aizoides | 1 | 1 | 0 | 0 | 0 | 0 | 0 | 0 | 0 |
| Alyssum armenum | 0 | 1 | 0 | 0 | 0 | 0 | 0 | 0 | 0 |
| Alyssum aurantiacum | 1 | 0 | 0 | 0 | 0 | 0 | 0 | 0 | 0 |
| Alyssum gehamense | 0 | 1 | 0 | 0 | 0 | 0 | 0 | 0 | 0 |
| Alyssum haussknechtii | 1 | 0 | 0 | 0 | 0 | 0 | 0 | 0 | 0 |
| Alyssum lanceolatum | 0 | 0 | 1 | 0 | 0 | 0 | 0 | 0 | 0 |
| Alyssum muelleri | 0 | 1 | 1 | 0 | 0 | 1 | 0 | 0 | 0 |
| Alyssum peltarioides | 1 | 1 | 0 | 0 | 0 | 1 | 0 | 0 | 0 |
| Alyssum persicum | 0 | 0 | 0 | 1 | 0 | 0 | 0 | 0 | 0 |
| Alyssum polycladum | 0 | 0 | 1 | 1 | 0 | 1 | 0 | 0 | 0 |
| Alyssum propinquum | 1 | 0 | 0 | 0 | 0 | 0 | 0 | 0 | 0 |
| Amygdalus carduchorum | 0 | 1 | 0 | 0 | 0 | 0 | 0 | 0 | 0 |
| Amygdalus elaeagnifolia | 0 | 0 | 0 | 1 | 1 | 0 | 0 | 0 | 0 |
| Anchonium elichrysifolium | 1 | 1 | 1 | 0 | 0 | 1 | 1 | 0 | 0 |
| Androsace albana | 0 | 1 | 0 | 0 | 0 | 0 | 0 | 0 | 0 |
| Androsace armeniaca | 0 | 1 | 0 | 0 | 0 | 0 | 0 | 0 | 0 |
| Androsace caduca | 0 | 1 | 0 | 0 | 0 | 0 | 0 | 0 | 0 |
| Androsace chamaejasme | 0 | 1 | 0 | 0 | 0 | 0 | 0 | 0 | 0 |
| Androsace intermedia | 0 | 1 | 0 | 0 | 0 | 0 | 0 | 0 | 0 |
| Androsace multiscapa | 1 | 1 | 0 | 0 | 0 | 0 | 0 | 0 | 0 |
| Androsace villosa | 1 | 1 | 1 | 0 | 0 | 0 | 0 | 0 | 0 |
| Anemone fasciculata | 0 | 1 | 0 | 0 | 0 | 0 | 0 | 0 | 0 |
| Antennaria dioica | 0 | 1 | 0 | 0 | 0 | 0 | 0 | 0 | 0 |
| Anthemis marschalliana | 0 | 1 | 0 | 0 | 0 | 0 | 0 | 0 | 0 |
| Anthriscus kotschyi | 1 | 1 | 0 | 0 | 0 | 0 | 0 | 0 | 0 |
| Arabis androsacea | 1 | 0 | 0 | 0 | 0 | 0 | 0 | 0 | 0 |
| Arabis brachycarpa | 0 | 1 | 0 | 0 | 0 | 0 | 0 | 0 | 0 |
| Arabis carduchorum | 0 | 1 | 0 | 0 | 0 | 1 | 0 | 0 | 0 |
| Arabis caucasica | 1 | 1 | 1 | 1 | 0 | 1 | 1 | 1 | 1 |
| Arabis graellsiiformis | 0 | 1 | 0 | 0 | 0 | 0 | 0 | 0 | 0 |
| Arabis lycia | 1 | 0 | 0 | 0 | 0 | 0 | 0 | 0 | 0 |
| Arabis rimarum | 0 | 0 | 1 | 0 | 0 | 0 | 0 | 0 | 0 |
| Arenaria angustisepala | 0 | 1 | 0 | 0 | 0 | 0 | 0 | 0 | 0 |
| Arenaria antitaurica | 1 | 0 | 0 | 0 | 0 | 0 | 0 | 0 | 0 |
| Arenaria balansae | 1 | 1 | 0 | 1 | 0 | 0 | 0 | 0 | 0 |
| Arenaria blepharophylla | 0 | 1 | 0 | 0 | 0 | 0 | 0 | 0 | 0 |
| Arenaria bulica | 0 | 0 | 0 | 1 | 1 | 0 | 0 | 0 | 0 |
| Arenaria cucubaloides | 1 | 1 | 0 | 1 | 0 | 1 | 0 | 0 | 0 |
| Arenaria davisii | 0 | 1 | 0 | 0 | 0 | 0 | 0 | 0 | 0 |
| Arenaria dianthoides | 0 | 1 | 0 | 0 | 0 | 1 | 0 | 0 | 0 |
| Arenaria gypsophiloides | 0 | 1 | 1 | 1 | 0 | 1 | 1 | 0 | 0 |
| Arenaria insignis | 0 | 0 | 1 | 0 | 0 | 0 | 0 | 0 | 0 |
| Arenaria ledebouriana | 1 | 0 | 0 | 0 | 0 | 0 | 0 | 0 | 0 |
| Arenaria lychnidea | 0 | 1 | 0 | 0 | 0 | 0 | 0 | 0 | 0 |
| Arenaria minutissima | 0 | 0 | 0 | 1 | 1 | 0 | 0 | 0 | 0 |
| Arenaria persica | 0 | 0 | 0 | 1 | 0 | 0 | 0 | 0 | 0 |
| Arenaria rotundifolia | 1 | 1 | 1 | 0 | 0 | 0 | 0 | 0 | 0 |
| Arenaria semiromica | 0 | 0 | 0 | 1 | 0 | 0 | 0 | 0 | 0 |
| Arnebia euchroma | 0 | 0 | 0 | 1 | 1 | 0 | 0 | 0 | 0 |
| Arnebia pulchra | 0 | 1 | 1 | 1 | 0 | 0 | 0 | 0 | 0 |
| Artemisia aucheri | 0 | 0 | 1 | 1 | 1 | 0 | 0 | 0 | 0 |
| Artemisia biennis | 0 | 1 | 1 | 1 | 1 | 1 | 0 | 0 | 0 |
| Artemisia chamaemelifolia | 0 | 1 | 1 | 0 | 0 | 1 | 1 | 0 | 0 |
| Artemisia haussknechtii | 0 | 1 | 0 | 1 | 0 | 0 | 0 | 0 | 0 |
| Artemisia melanolepis | 0 | 0 | 1 | 0 | 0 | 1 | 0 | 0 | 0 |
| Artemisia persica | 0 | 0 | 0 | 1 | 1 | 0 | 0 | 0 | 0 |
| Artemisia splendens | 0 | 1 | 1 | 0 | 0 | 1 | 0 | 0 | 0 |
| Asperula affinis | 0 | 1 | 0 | 0 | 0 | 0 | 0 | 0 | 0 |
| Asperula capitellata | 1 | 1 | 0 | 0 | 0 | 0 | 0 | 0 | 1 |
| Asperula fragillima | 0 | 0 | 0 | 1 | 0 | 0 | 0 | 0 | 0 |
| Asperula glomerata | 1 | 1 | 1 | 1 | 1 | 0 | 1 | 0 | 0 |
| Asperula laxiflora | 0 | 1 | 0 | 0 | 0 | 0 | 0 | 0 | 0 |
| Asperula lycia | 1 | 0 | 0 | 0 | 0 | 0 | 0 | 0 | 0 |
| Asperula nitida | 0 | 0 | 0 | 0 | 0 | 0 | 0 | 0 | 1 |
| Asperula pontica | 0 | 1 | 0 | 0 | 0 | 0 | 0 | 0 | 0 |
| Asperula prostrata | 0 | 1 | 0 | 0 | 0 | 1 | 1 | 0 | 0 |
| Asperula rechingeri | 0 | 0 | 0 | 1 | 0 | 0 | 0 | 0 | 0 |
| Asplenium tadei | 1 | 0 | 0 | 0 | 0 | 0 | 0 | 0 | 0 |
| Asplenium viride | 0 | 0 | 0 | 0 | 1 | 0 | 0 | 0 | 0 |
| Aster alpinus | 1 | 1 | 1 | 0 | 0 | 1 | 0 | 0 | 0 |
| Astragalus abditus | 0 | 0 | 0 | 1 | 0 | 0 | 0 | 0 | 0 |
| Astragalus acmophyllus | 1 | 1 | 0 | 0 | 0 | 0 | 0 | 0 | 0 |
| Astragalus aegobromus | 0 | 1 | 1 | 1 | 0 | 1 | 1 | 1 | 0 |
| Astragalus aestivorum | 0 | 0 | 1 | 0 | 0 | 0 | 0 | 0 | 0 |
| Astragalus agassii | 0 | 1 | 0 | 0 | 0 | 0 | 0 | 0 | 0 |
| Astragalus aladagensis | 1 | 0 | 0 | 0 | 0 | 0 | 0 | 0 | 0 |
| Astragalus alamkuhensis | 0 | 0 | 1 | 0 | 0 | 0 | 0 | 0 | 0 |
| Astragalus alpinus | 0 | 1 | 0 | 0 | 0 | 0 | 0 | 0 | 0 |
| Astragalus alyssoides | 0 | 1 | 1 | 1 | 0 | 1 | 0 | 1 | 0 |
| Astragalus argaeus | 1 | 0 | 0 | 0 | 0 | 0 | 0 | 0 | 0 |
| Astragalus atricapillus | 0 | 0 | 1 | 0 | 0 | 0 | 0 | 0 | 0 |
| Astragalus aureus | 0 | 1 | 1 | 0 | 0 | 1 | 0 | 1 | 0 |
| Astragalus azizii | 0 | 0 | 0 | 0 | 0 | 1 | 0 | 0 | 0 |
| Astragalus bahcesarayensis | 0 | 1 | 0 | 0 | 0 | 0 | 0 | 0 | 0 |
| Astragalus barnassari | 0 | 1 | 1 | 0 | 0 | 1 | 1 | 0 | 0 |
| Astragalus bashkalensis | 0 | 0 | 0 | 0 | 0 | 1 | 0 | 0 | 0 |
| Astragalus beckerianus | 0 | 1 | 0 | 0 | 0 | 0 | 0 | 0 | 0 |
| Astragalus beckii | 0 | 1 | 1 | 0 | 0 | 1 | 0 | 0 | 0 |
| Astragalus bounophilus | 0 | 0 | 1 | 0 | 0 | 0 | 0 | 1 | 0 |
| Astragalus brachycalyx | 0 | 1 | 0 | 1 | 0 | 1 | 1 | 0 | 0 |
| Astragalus capax | 0 | 0 | 1 | 0 | 0 | 0 | 0 | 0 | 0 |
| Astragalus capito | 0 | 0 | 1 | 0 | 0 | 0 | 0 | 0 | 0 |
| Astragalus carmanicus | 0 | 0 | 0 | 0 | 1 | 0 | 0 | 0 | 0 |
| Astragalus cataonicus | 1 | 0 | 0 | 0 | 0 | 0 | 0 | 0 | 0 |
| Astragalus chartostegius | 0 | 0 | 0 | 1 | 0 | 0 | 0 | 0 | 0 |
| Astragalus chionobiiformis | 0 | 0 | 0 | 0 | 0 | 0 | 1 | 0 | 0 |
| Astragalus chrysanthus | 0 | 0 | 1 | 0 | 0 | 0 | 0 | 0 | 0 |
| Astragalus ciloensis | 0 | 1 | 0 | 0 | 0 | 0 | 0 | 0 | 0 |
| Astragalus confusus | 0 | 0 | 1 | 0 | 0 | 0 | 0 | 0 | 0 |
| Astragalus czorochensis | 0 | 1 | 0 | 0 | 0 | 0 | 0 | 0 | 0 |
| Astragalus daenaensis | 0 | 0 | 0 | 1 | 0 | 0 | 0 | 0 | 0 |
| Astragalus dasycarpus | 0 | 1 | 0 | 0 | 0 | 0 | 0 | 0 | 0 |
| Astragalus declinatus | 0 | 1 | 0 | 0 | 0 | 0 | 0 | 0 | 0 |
| Astragalus demavendicus | 0 | 0 | 1 | 0 | 0 | 0 | 0 | 0 | 0 |
| Astragalus dieterlei | 0 | 0 | 0 | 0 | 1 | 0 | 0 | 0 | 0 |
| Astragalus dumanii | 1 | 0 | 0 | 0 | 0 | 0 | 0 | 0 | 0 |
| Astragalus dzebrailicus | 0 | 1 | 0 | 0 | 0 | 1 | 0 | 0 | 0 |
| Astragalus eriocalyx | 0 | 0 | 0 | 0 | 0 | 1 | 0 | 0 | 0 |
| Astragalus eriocephalus | 0 | 1 | 0 | 0 | 0 | 0 | 0 | 0 | 1 |
| Astragalus erivanensis | 0 | 1 | 0 | 0 | 0 | 0 | 0 | 0 | 0 |
| Astragalus ermineus | 0 | 1 | 0 | 0 | 0 | 0 | 0 | 0 | 0 |
| Astragalus euoplus | 0 | 1 | 0 | 0 | 0 | 0 | 0 | 0 | 0 |
| Astragalus exspectatus | 0 | 0 | 0 | 1 | 0 | 0 | 0 | 0 | 0 |
| Astragalus fragiferus | 0 | 0 | 0 | 1 | 1 | 0 | 0 | 0 | 0 |
| Astragalus fragrans | 0 | 1 | 1 | 0 | 0 | 1 | 0 | 0 | 0 |
| Astragalus fraxinifolius | 1 | 1 | 0 | 0 | 0 | 0 | 0 | 0 | 0 |
| Astragalus frickii | 0 | 1 | 0 | 0 | 0 | 0 | 0 | 0 | 0 |
| Astragalus gevashensis | 0 | 1 | 0 | 0 | 0 | 0 | 0 | 0 | 0 |
| Astragalus geyikdaghensis | 1 | 0 | 0 | 0 | 0 | 0 | 0 | 0 | 0 |
| Astragalus gezeldarensis | 0 | 1 | 0 | 0 | 0 | 0 | 0 | 0 | 0 |
| Astragalus ghashghaicus | 0 | 0 | 0 | 1 | 0 | 0 | 0 | 0 | 0 |
| Astragalus globosus | 1 | 1 | 0 | 0 | 0 | 0 | 0 | 0 | 0 |
| Astragalus griseus | 0 | 0 | 0 | 1 | 0 | 0 | 0 | 0 | 0 |
| Astragalus hareftae | 0 | 1 | 0 | 0 | 0 | 1 | 0 | 0 | 0 |
| Astragalus hausknechtii | 1 | 1 | 0 | 0 | 0 | 0 | 0 | 0 | 0 |
| Astragalus herbertii | 0 | 0 | 1 | 0 | 0 | 0 | 0 | 0 | 0 |
| Astragalus heterozyx | 0 | 0 | 0 | 0 | 1 | 0 | 0 | 0 | 0 |
| Astragalus hezarensis | 0 | 0 | 0 | 0 | 1 | 0 | 0 | 0 | 0 |
| Astragalus hirticalyx | 0 | 1 | 0 | 0 | 0 | 1 | 1 | 0 | 0 |
| Astragalus horasanicus | 0 | 1 | 0 | 0 | 0 | 0 | 0 | 0 | 0 |
| Astragalus horridus | 0 | 0 | 0 | 1 | 1 | 0 | 0 | 0 | 0 |
| Astragalus humilis | 0 | 1 | 0 | 0 | 0 | 0 | 0 | 0 | 0 |
| Astragalus hyalolepis | 0 | 1 | 0 | 0 | 0 | 0 | 0 | 0 | 0 |
| Astragalus icmadophilus | 0 | 1 | 1 | 0 | 0 | 1 | 1 | 0 | 0 |
| Astragalus incertus | 0 | 1 | 0 | 0 | 0 | 0 | 0 | 0 | 0 |
| Astragalus inexpectatus | 0 | 0 | 0 | 1 | 0 | 0 | 0 | 0 | 0 |
| Astragalus issatissensis | 0 | 0 | 0 | 1 | 0 | 0 | 0 | 0 | 0 |
| Astragalus jodotropis | 0 | 0 | 1 | 0 | 0 | 0 | 0 | 0 | 0 |
| Astragalus johannis | 0 | 0 | 0 | 1 | 0 | 0 | 0 | 0 | 0 |
| Astragalus karabaghensis | 0 | 1 | 1 | 0 | 0 | 1 | 0 | 0 | 0 |
| Astragalus lalesarensis | 0 | 0 | 0 | 0 | 1 | 0 | 0 | 0 | 0 |
| Astragalus lanatus | 0 | 1 | 0 | 0 | 0 | 0 | 0 | 0 | 0 |
| Astragalus laricus | 0 | 0 | 1 | 0 | 0 | 0 | 0 | 0 | 0 |
| Astragalus latistipulatus | 0 | 1 | 0 | 0 | 0 | 0 | 0 | 0 | 0 |
| Astragalus latus | 0 | 1 | 0 | 0 | 0 | 0 | 0 | 0 | 0 |
| Astragalus leiophyllus | 0 | 1 | 0 | 0 | 0 | 1 | 1 | 0 | 0 |
| Astragalus lineatus | 1 | 1 | 1 | 0 | 0 | 1 | 1 | 1 | 0 |
| Astragalus lunatus | 1 | 0 | 0 | 0 | 0 | 0 | 0 | 0 | 0 |
| Astragalus lycioides | 0 | 0 | 1 | 1 | 1 | 0 | 0 | 0 | 0 |
| Astragalus macrosemius | 0 | 0 | 1 | 0 | 0 | 0 | 0 | 0 | 0 |
| Astragalus macrourus | 0 | 1 | 0 | 1 | 0 | 1 | 0 | 0 | 0 |
| Astragalus mahneshanensis | 0 | 0 | 0 | 0 | 0 | 0 | 0 | 1 | 0 |
| Astragalus melanocalyx | 0 | 0 | 0 | 1 | 0 | 0 | 0 | 0 | 0 |
| Astragalus melanocarpus | 1 | 1 | 0 | 0 | 0 | 0 | 0 | 0 | 0 |
| Astragalus melanodon | 0 | 0 | 0 | 1 | 0 | 0 | 0 | 0 | 0 |
| Astragalus modestus | 0 | 0 | 1 | 0 | 0 | 0 | 0 | 0 | 0 |
| Astragalus monanthemus | 0 | 0 | 1 | 0 | 0 | 0 | 0 | 0 | 0 |
| Astragalus montis-alamkuhi | 0 | 0 | 1 | 0 | 0 | 0 | 0 | 0 | 0 |
| Astragalus montis-parrowii | 0 | 0 | 0 | 1 | 0 | 0 | 0 | 0 | 0 |
| Astragalus montis-varvashti | 0 | 0 | 1 | 0 | 0 | 0 | 0 | 0 | 0 |
| Astragalus murinus | 0 | 0 | 0 | 1 | 0 | 0 | 0 | 0 | 0 |
| Astragalus nabelekii | 0 | 1 | 0 | 0 | 0 | 0 | 0 | 0 | 0 |
| Astragalus nezaketiae | 0 | 1 | 0 | 0 | 0 | 0 | 0 | 0 | 0 |
| Astragalus nezva-montis | 0 | 0 | 1 | 0 | 0 | 0 | 0 | 0 | 0 |
| Astragalus nigropedunculatus | 0 | 1 | 0 | 0 | 0 | 0 | 0 | 0 | 0 |
| Astragalus ochrochlorus | 0 | 1 | 1 | 1 | 0 | 0 | 0 | 0 | 0 |
| Astragalus oreades | 0 | 1 | 0 | 0 | 0 | 0 | 0 | 0 | 0 |
| Astragalus ovigerus | 0 | 0 | 0 | 1 | 0 | 0 | 0 | 0 | 0 |
| Astragalus pascuicola | 0 | 1 | 0 | 0 | 0 | 0 | 0 | 0 | 0 |
| Astragalus patrius | 0 | 0 | 1 | 1 | 0 | 1 | 0 | 1 | 0 |
| Astragalus pauperiflorus | 0 | 0 | 0 | 0 | 0 | 1 | 0 | 0 | 0 |
| Astragalus pelliger | 1 | 1 | 0 | 0 | 0 | 0 | 0 | 0 | 0 |
| Astragalus pennatus | 1 | 1 | 0 | 0 | 0 | 0 | 0 | 0 | 0 |
| Astragalus perdurans | 0 | 0 | 1 | 0 | 0 | 0 | 0 | 0 | 0 |
| Astragalus perrarus | 0 | 1 | 0 | 0 | 0 | 0 | 0 | 0 | 0 |
| Astragalus pinetorum | 1 | 1 | 1 | 1 | 0 | 1 | 1 | 0 | 0 |
| Astragalus plagiophacos | 0 | 0 | 1 | 0 | 0 | 0 | 0 | 0 | 0 |
| Astragalus platysematus | 0 | 0 | 1 | 0 | 0 | 0 | 0 | 0 | 0 |
| Astragalus pluriflorus | 0 | 0 | 0 | 0 | 0 | 0 | 0 | 1 | 0 |
| Astragalus podosphaerus | 0 | 0 | 0 | 1 | 0 | 0 | 0 | 0 | 0 |
| Astragalus polyanthus | 0 | 1 | 0 | 0 | 0 | 1 | 0 | 0 | 0 |
| Astragalus polygala | 0 | 1 | 0 | 0 | 0 | 0 | 0 | 0 | 0 |
| Astragalus pseudofragrans | 0 | 0 | 0 | 0 | 0 | 0 | 1 | 0 | 0 |
| Astragalus pseudopinetorum | 0 | 0 | 0 | 0 | 0 | 0 | 0 | 0 | 1 |
| Astragalus pseudoshebarensis | 0 | 0 | 0 | 1 | 0 | 0 | 0 | 0 | 0 |
| Astragalus rechingeri | 0 | 1 | 0 | 0 | 0 | 0 | 0 | 0 | 0 |
| Astragalus remotiflorus | 0 | 0 | 1 | 1 | 1 | 0 | 0 | 0 | 0 |
| Astragalus robertianus | 0 | 1 | 0 | 0 | 0 | 0 | 0 | 0 | 0 |
| Astragalus rubriflorus | 0 | 0 | 1 | 0 | 0 | 0 | 0 | 0 | 0 |
| Astragalus rubrolineatus | 0 | 0 | 1 | 0 | 0 | 0 | 0 | 0 | 0 |
| Astragalus rudimentus | 0 | 0 | 0 | 0 | 0 | 1 | 0 | 0 | 0 |
| Astragalus sachanewii | 0 | 1 | 0 | 0 | 0 | 0 | 0 | 0 | 0 |
| Astragalus sahendi | 0 | 0 | 0 | 0 | 0 | 1 | 0 | 0 | 0 |
| Astragalus savellanicus | 0 | 0 | 0 | 0 | 0 | 1 | 0 | 0 | 0 |
| Astragalus shahsavaranicus | 0 | 0 | 0 | 1 | 0 | 0 | 0 | 0 | 0 |
| Astragalus sphaeranthus | 0 | 1 | 0 | 1 | 0 | 0 | 1 | 0 | 0 |
| Astragalus stenosemioides | 1 | 1 | 0 | 0 | 0 | 0 | 0 | 0 | 0 |
| Astragalus stenostegius | 0 | 0 | 0 | 1 | 0 | 0 | 0 | 0 | 0 |
| Astragalus stridii | 1 | 0 | 0 | 0 | 0 | 0 | 0 | 0 | 0 |
| Astragalus subhanensis | 0 | 1 | 0 | 0 | 0 | 0 | 0 | 0 | 0 |
| Astragalus subsecundus | 0 | 1 | 1 | 1 | 0 | 1 | 0 | 0 | 0 |
| Astragalus taleshensis | 0 | 0 | 1 | 0 | 0 | 0 | 0 | 0 | 0 |
| Astragalus tauricolus | 1 | 1 | 0 | 0 | 0 | 0 | 0 | 0 | 0 |
| Astragalus tenuiscapus | 0 | 0 | 0 | 1 | 1 | 0 | 0 | 0 | 0 |
| Astragalus trabzonicus | 0 | 1 | 0 | 0 | 0 | 0 | 0 | 0 | 0 |
| Astragalus turgidus | 0 | 0 | 0 | 1 | 0 | 0 | 0 | 0 | 0 |
| Astragalus uraniolimneus | 0 | 1 | 0 | 0 | 0 | 1 | 0 | 0 | 0 |
| Astragalus vavilovii | 0 | 1 | 0 | 0 | 0 | 0 | 0 | 0 | 0 |
| Astragalus xerophilus | 1 | 1 | 0 | 0 | 0 | 1 | 0 | 0 | 0 |
| Astragalus zagrosicus | 0 | 0 | 0 | 1 | 0 | 0 | 0 | 0 | 0 |
| Astragalus zerdanus | 0 | 0 | 0 | 1 | 1 | 0 | 0 | 0 | 0 |
| Astragalus zohrabi | 0 | 1 | 0 | 0 | 0 | 1 | 0 | 0 | 0 |
| Asyneuma ekimianum | 1 | 0 | 0 | 0 | 0 | 0 | 0 | 0 | 0 |
| Asyneuma filipes | 0 | 1 | 0 | 0 | 0 | 0 | 0 | 0 | 0 |
| Asyneuma multicaule | 0 | 0 | 0 | 1 | 0 | 0 | 1 | 0 | 0 |
| Asyneuma persicum | 0 | 1 | 0 | 1 | 0 | 0 | 1 | 0 | 0 |
| Asyneuma pulchellum | 0 | 1 | 0 | 0 | 0 | 0 | 0 | 0 | 0 |
| Asyneuma rigidum | 0 | 1 | 0 | 0 | 0 | 0 | 0 | 0 | 0 |
| Athyrium distentifolium | 0 | 1 | 0 | 0 | 0 | 0 | 0 | 0 | 0 |
| Aubrieta anamasica | 1 | 0 | 0 | 0 | 0 | 0 | 0 | 0 | 0 |
| Aurinia calycocarpum | 0 | 1 | 0 | 0 | 0 | 0 | 0 | 0 | 0 |
| Aurinia rupestris | 1 | 1 | 0 | 0 | 0 | 0 | 0 | 0 | 0 |
| Barbamine procumbens | 0 | 1 | 0 | 0 | 0 | 0 | 0 | 0 | 0 |
| Barbarea minor | 1 | 1 | 0 | 1 | 0 | 0 | 0 | 0 | 0 |
| Bellardiochloa argaea | 1 | 0 | 0 | 0 | 0 | 0 | 0 | 0 | 1 |
| Bellardiochloa polychroa | 0 | 1 | 0 | 0 | 0 | 0 | 0 | 0 | 0 |
| Bellevalia paradoxa | 0 | 1 | 1 | 1 | 0 | 1 | 1 | 1 | 0 |
| Bellevalia rixii | 0 | 1 | 0 | 0 | 0 | 0 | 0 | 0 | 0 |
| Bellevalia tristis | 0 | 0 | 0 | 1 | 0 | 0 | 0 | 0 | 0 |
| Betonica nivea | 0 | 0 | 1 | 0 | 0 | 0 | 0 | 0 | 0 |
| Bornmuellera cappadocica | 0 | 1 | 0 | 0 | 0 | 0 | 0 | 0 | 0 |
| Brachyactis roylei | 0 | 0 | 0 | 1 | 1 | 0 | 0 | 0 | 0 |
| Briza marcowiczii | 0 | 1 | 0 | 0 | 0 | 0 | 0 | 0 | 0 |
| Bromus armenus | 0 | 1 | 0 | 0 | 0 | 0 | 0 | 0 | 0 |
| Bromus biebersteinii | 0 | 0 | 0 | 0 | 0 | 1 | 0 | 0 | 0 |
| Bromus cappadocicus | 1 | 1 | 0 | 0 | 0 | 0 | 0 | 0 | 1 |
| Bromus confinis | 0 | 0 | 1 | 0 | 0 | 0 | 0 | 0 | 0 |
| Bromus frigidus | 0 | 0 | 1 | 1 | 0 | 0 | 0 | 0 | 0 |
| Bromus gracillimus | 0 | 0 | 1 | 1 | 1 | 1 | 0 | 0 | 0 |
| Bromus stenostachyus | 0 | 0 | 1 | 0 | 0 | 0 | 0 | 0 | 0 |
| Bromus tomentosus | 0 | 1 | 1 | 0 | 0 | 0 | 0 | 0 | 0 |
| Bromus variegatus | 0 | 1 | 1 | 1 | 0 | 1 | 1 | 0 | 0 |
| Bufonia koelzii | 0 | 0 | 1 | 0 | 0 | 0 | 0 | 0 | 0 |
| Bufonia kotschyana | 0 | 1 | 1 | 1 | 0 | 1 | 0 | 1 | 0 |
| Bufonia micrantha | 0 | 0 | 0 | 1 | 0 | 0 | 0 | 0 | 0 |
| Bufonia stapfii | 0 | 0 | 0 | 1 | 0 | 0 | 0 | 0 | 0 |
| Bunium brachyactis | 1 | 1 | 0 | 0 | 0 | 0 | 0 | 0 | 0 |
| Bupleurum falcatum | 1 | 1 | 0 | 0 | 0 | 0 | 0 | 0 | 1 |
| Calamagrostis decora | 0 | 0 | 0 | 0 | 0 | 1 | 0 | 0 | 0 |
| Calamagrostis parsana | 0 | 1 | 1 | 0 | 0 | 0 | 0 | 0 | 0 |
| Calamintha caroli-henricana | 0 | 1 | 0 | 0 | 0 | 0 | 0 | 0 | 0 |
| Campanula armena | 0 | 1 | 0 | 0 | 0 | 0 | 0 | 0 | 0 |
| Campanula bayerniana | 0 | 1 | 0 | 0 | 0 | 1 | 0 | 0 | 0 |
| Campanula bornmuelleri | 0 | 1 | 0 | 0 | 0 | 0 | 0 | 0 | 0 |
| Campanula choruhensis | 0 | 1 | 0 | 0 | 0 | 0 | 0 | 0 | 0 |
| Campanula collina | 0 | 1 | 0 | 0 | 0 | 0 | 0 | 0 | 0 |
| Campanula conferta | 0 | 1 | 0 | 0 | 0 | 0 | 0 | 0 | 0 |
| Campanula gilliatii | 0 | 0 | 0 | 0 | 0 | 1 | 0 | 0 | 0 |
| Campanula hedgei | 0 | 1 | 0 | 0 | 0 | 0 | 0 | 0 | 0 |
| Campanula hermannii | 0 | 0 | 0 | 1 | 0 | 0 | 0 | 0 | 0 |
| Campanula humillima | 0 | 0 | 0 | 1 | 0 | 0 | 0 | 0 | 0 |
| Campanula karakuschensis | 0 | 1 | 0 | 0 | 0 | 1 | 0 | 0 | 0 |
| Campanula lourica | 0 | 0 | 1 | 0 | 0 | 0 | 0 | 0 | 0 |
| Campanula luristanica | 0 | 0 | 0 | 1 | 0 | 0 | 0 | 0 | 0 |
| Campanula saxifraga | 0 | 1 | 0 | 0 | 0 | 0 | 0 | 0 | 0 |
| Campanula stevenii | 1 | 1 | 1 | 0 | 0 | 1 | 0 | 0 | 0 |
| Campanula telephioides | 1 | 0 | 0 | 0 | 0 | 0 | 0 | 0 | 0 |
| Campanula tridentata | 1 | 1 | 0 | 0 | 0 | 0 | 0 | 0 | 0 |
| Campanula zangezura | 0 | 1 | 0 | 0 | 0 | 0 | 0 | 0 | 0 |
| Carduus lanuginosus | 1 | 1 | 0 | 0 | 0 | 0 | 0 | 0 | 0 |
| Carex atherodes | 0 | 1 | 0 | 0 | 0 | 0 | 0 | 0 | 0 |
| Carex atrata | 1 | 1 | 0 | 0 | 0 | 0 | 0 | 0 | 0 |
| Carex brevicollis | 0 | 1 | 0 | 0 | 0 | 0 | 0 | 0 | 0 |
| Carex capitellata | 0 | 1 | 0 | 0 | 0 | 0 | 0 | 0 | 0 |
| Carex caucasica | 0 | 1 | 1 | 0 | 0 | 0 | 0 | 0 | 0 |
| Carex decaulescens | 0 | 0 | 1 | 0 | 0 | 0 | 0 | 0 | 0 |
| Carex disticha | 0 | 1 | 0 | 0 | 0 | 0 | 0 | 0 | 0 |
| Carex flacca | 0 | 1 | 0 | 0 | 0 | 0 | 0 | 0 | 0 |
| Carex magellanica | 0 | 1 | 0 | 0 | 0 | 0 | 0 | 0 | 0 |
| Carex medwedewii | 0 | 1 | 0 | 0 | 0 | 0 | 1 | 0 | 0 |
| Carex melanantha | 0 | 0 | 1 | 0 | 0 | 0 | 0 | 0 | 0 |
| Carex melanorrhyncha | 0 | 1 | 0 | 0 | 0 | 0 | 0 | 0 | 0 |
| Carex michelii | 0 | 1 | 0 | 0 | 0 | 0 | 0 | 0 | 0 |
| Carex microglochin | 0 | 1 | 0 | 0 | 0 | 0 | 0 | 0 | 0 |
| Carex oligantha | 0 | 1 | 0 | 0 | 0 | 0 | 0 | 0 | 0 |
| Carex orbicularis | 0 | 1 | 1 | 1 | 1 | 1 | 1 | 0 | 0 |
| Carex oreophila | 1 | 1 | 1 | 1 | 0 | 1 | 0 | 0 | 0 |
| Carex ornithopoda | 0 | 1 | 0 | 0 | 0 | 0 | 0 | 0 | 0 |
| Carex pauescens | 0 | 1 | 0 | 0 | 0 | 0 | 0 | 0 | 0 |
| Carex pontica | 0 | 1 | 0 | 0 | 0 | 0 | 0 | 0 | 0 |
| Carex pseudofoetida | 0 | 0 | 1 | 0 | 1 | 1 | 0 | 0 | 0 |
| Carex pyrenaica | 0 | 1 | 0 | 0 | 0 | 0 | 0 | 0 | 0 |
| Carex stenophylla | 0 | 0 | 1 | 1 | 0 | 1 | 0 | 0 | 0 |
| Carex tomentosa | 0 | 1 | 0 | 0 | 0 | 0 | 0 | 0 | 0 |
| Carex transcaucasica | 0 | 1 | 0 | 0 | 0 | 0 | 0 | 0 | 0 |
| Carex tristis | 1 | 1 | 0 | 0 | 0 | 0 | 0 | 0 | 0 |
| Carex umbrosa | 0 | 1 | 0 | 0 | 0 | 0 | 0 | 0 | 1 |
| Carum carvi | 0 | 1 | 1 | 1 | 1 | 1 | 0 | 0 | 0 |
| Carum caucasicum | 0 | 1 | 0 | 0 | 0 | 1 | 0 | 0 | 0 |
| Carum komarovii | 0 | 1 | 0 | 0 | 0 | 0 | 0 | 0 | 0 |
| Carum meifolium | 0 | 1 | 0 | 0 | 0 | 0 | 0 | 0 | 0 |
| Carum rupicola | 1 | 0 | 0 | 0 | 0 | 0 | 0 | 0 | 0 |
| Catabrosa aquatica | 0 | 1 | 1 | 0 | 0 | 0 | 0 | 0 | 0 |
| Centaurea appendicigera | 0 | 1 | 0 | 0 | 0 | 0 | 0 | 0 | 0 |
| Centaurea armena | 0 | 1 | 0 | 0 | 0 | 0 | 0 | 0 | 0 |
| Centaurea cheiranthifolia | 0 | 1 | 0 | 0 | 0 | 0 | 0 | 0 | 0 |
| Centaurea congesta | 0 | 0 | 1 | 0 | 0 | 1 | 0 | 0 | 0 |
| Centaurea drabifolia | 1 | 1 | 0 | 0 | 0 | 0 | 0 | 0 | 1 |
| Centaurea elbrusensis | 0 | 1 | 1 | 0 | 0 | 1 | 0 | 0 | 0 |
| Centaurea incanescens | 0 | 0 | 0 | 0 | 0 | 1 | 1 | 0 | 0 |
| Centaurea karduchorum | 0 | 1 | 0 | 0 | 0 | 0 | 0 | 0 | 0 |
| Centaurea lanigera | 1 | 1 | 0 | 0 | 0 | 0 | 0 | 0 | 0 |
| Centaurea macrocephala | 0 | 1 | 0 | 0 | 0 | 0 | 0 | 0 | 0 |
| Centaurea mucronifera | 1 | 1 | 0 | 0 | 0 | 0 | 0 | 0 | 0 |
| Centaurea nigrofimbria | 0 | 1 | 0 | 0 | 0 | 0 | 0 | 0 | 0 |
| Centaurea pichleri | 1 | 1 | 0 | 0 | 0 | 0 | 0 | 0 | 0 |
| Centaurea poluninii | 0 | 1 | 0 | 0 | 0 | 0 | 0 | 0 | 0 |
| Centaurea pulcherrima | 0 | 1 | 0 | 0 | 0 | 0 | 0 | 0 | 0 |
| Centaurea rhizantha | 0 | 1 | 1 | 0 | 0 | 1 | 1 | 0 | 0 |
| Centaurea schelkovnikovii | 0 | 1 | 0 | 0 | 0 | 0 | 0 | 0 | 0 |
| Centaurea sieheana | 1 | 0 | 0 | 0 | 0 | 0 | 0 | 0 | 0 |
| Cephalaria cilodaghensis | 0 | 1 | 0 | 0 | 0 | 0 | 0 | 0 | 0 |
| Cephalaria gigantea | 0 | 1 | 0 | 0 | 0 | 0 | 0 | 0 | 0 |
| Cephalaria kleinii | 0 | 0 | 1 | 0 | 0 | 0 | 0 | 0 | 0 |
| Cephalaria microcephala | 0 | 1 | 1 | 1 | 0 | 1 | 1 | 1 | 0 |
| Cephalaria scoparia | 1 | 0 | 0 | 0 | 0 | 0 | 0 | 0 | 0 |
| Cephalaria sparsipilosa | 0 | 1 | 0 | 0 | 0 | 0 | 0 | 0 | 0 |
| Cerastium araraticum | 0 | 1 | 0 | 0 | 0 | 0 | 0 | 0 | 0 |
| Cerastium cerastoides | 1 | 1 | 1 | 1 | 1 | 1 | 1 | 0 | 1 |
| Cerastium davuricum | 0 | 1 | 0 | 0 | 0 | 0 | 0 | 0 | 0 |
| Cerastium gnaphalodes | 1 | 1 | 0 | 0 | 0 | 0 | 0 | 0 | 0 |
| Cerastium lazicum | 0 | 1 | 0 | 0 | 0 | 0 | 0 | 0 | 0 |
| Cerastium persicum | 0 | 0 | 1 | 1 | 0 | 0 | 0 | 0 | 0 |
| Cerastium pseudokasbek | 0 | 1 | 0 | 0 | 0 | 0 | 0 | 0 | 0 |
| Cerastium purpurascens | 1 | 1 | 1 | 0 | 0 | 1 | 0 | 0 | 1 |
| Cerastium szowitsii | 0 | 1 | 0 | 0 | 0 | 0 | 0 | 0 | 0 |
| Cerasus brachypetala | 0 | 1 | 0 | 1 | 0 | 0 | 1 | 0 | 0 |
| Cerinthe glabra | 0 | 1 | 0 | 0 | 0 | 0 | 0 | 0 | 0 |
| Chaenorhinum grossecostatum | 0 | 0 | 0 | 1 | 1 | 0 | 0 | 0 | 0 |
| Chaerophyllum astrantiae | 0 | 1 | 0 | 0 | 0 | 0 | 0 | 0 | 0 |
| Chaerophyllum hakkiaricum | 0 | 1 | 0 | 0 | 0 | 0 | 0 | 0 | 0 |
| Chaerophyllum khorossanicum | 0 | 0 | 1 | 0 | 0 | 0 | 0 | 0 | 0 |
| Chaerophyllum macrospermum | 1 | 1 | 1 | 1 | 0 | 1 | 1 | 0 | 0 |
| Chaerophyllum nivale | 0 | 0 | 0 | 1 | 0 | 0 | 0 | 0 | 0 |
| Chamaegeron asterellus | 0 | 0 | 0 | 1 | 1 | 0 | 0 | 0 | 0 |
| Chamaesciadium acaule | 0 | 1 | 0 | 0 | 0 | 1 | 0 | 0 | 0 |
| Chenopodium foliosum | 1 | 1 | 1 | 1 | 1 | 1 | 1 | 0 | 1 |
| Cicer anatolicum | 1 | 1 | 0 | 1 | 0 | 0 | 0 | 1 | 0 |
| Cicer incisum | 1 | 1 | 0 | 0 | 0 | 0 | 0 | 0 | 0 |
| Cicer stapfianum | 0 | 0 | 0 | 1 | 0 | 0 | 0 | 0 | 0 |
| Cicer tragacanthoides | 0 | 0 | 1 | 1 | 1 | 0 | 0 | 0 | 0 |
| Cicerbita adenophora | 0 | 1 | 0 | 0 | 0 | 0 | 0 | 0 | 0 |
| Cirsium aggregatum | 0 | 1 | 0 | 0 | 0 | 0 | 0 | 0 | 0 |
| Cirsium ellenbergii | 1 | 0 | 0 | 0 | 0 | 0 | 0 | 0 | 0 |
| Cirsium kosmelii | 0 | 1 | 0 | 0 | 0 | 0 | 0 | 0 | 0 |
| Cirsium lappaceum | 0 | 1 | 1 | 1 | 0 | 1 | 0 | 0 | 0 |
| Cirsium mimitum | 0 | 1 | 0 | 0 | 0 | 0 | 0 | 0 | 0 |
| Cirsium obvallatum | 0 | 1 | 0 | 0 | 0 | 0 | 0 | 0 | 0 |
| Cirsium pseudobracteosum | 0 | 1 | 0 | 0 | 0 | 0 | 0 | 0 | 0 |
| Cirsium rhizocephalum | 1 | 1 | 1 | 1 | 1 | 1 | 0 | 0 | 0 |
| Cirsium simplex | 0 | 1 | 0 | 0 | 0 | 0 | 0 | 0 | 0 |
| Cirsium tomentosum | 0 | 1 | 0 | 0 | 0 | 0 | 0 | 0 | 0 |
| Clastopus erubescens | 0 | 0 | 0 | 1 | 0 | 0 | 1 | 0 | 0 |
| Clastopus vestitus | 0 | 0 | 1 | 1 | 0 | 0 | 0 | 1 | 0 |
| Cochlearia aucheri | 0 | 1 | 0 | 0 | 0 | 0 | 0 | 0 | 0 |
| Cochlearia sintenisii | 0 | 1 | 0 | 0 | 0 | 0 | 0 | 0 | 0 |
| Colchicum kurdicum | 0 | 1 | 0 | 0 | 0 | 0 | 0 | 0 | 0 |
| Colpodium araraticum | 0 | 1 | 0 | 0 | 0 | 0 | 0 | 0 | 0 |
| Colpodium fibrosum | 0 | 1 | 0 | 0 | 0 | 0 | 0 | 0 | 0 |
| Colpodium gillettii | 0 | 1 | 0 | 0 | 0 | 0 | 1 | 0 | 0 |
| Colpodium parviflorum | 0 | 1 | 1 | 1 | 0 | 0 | 0 | 0 | 0 |
| Colpodium variegatum | 1 | 1 | 0 | 0 | 0 | 0 | 0 | 0 | 0 |
| Colpodium versicolor | 0 | 1 | 1 | 0 | 0 | 1 | 0 | 0 | 0 |
| Colpodium violaceum | 0 | 0 | 0 | 1 | 0 | 0 | 1 | 0 | 0 |
| Coluteocarpus vesicaria | 0 | 1 | 0 | 1 | 0 | 0 | 0 | 0 | 0 |
| Coronilla orientalis | 0 | 1 | 0 | 0 | 0 | 0 | 0 | 0 | 0 |
| Cortusa matthioli | 0 | 0 | 1 | 0 | 0 | 0 | 0 | 0 | 0 |
| Corydalis alpestris | 0 | 1 | 0 | 0 | 0 | 0 | 0 | 0 | 0 |
| Corydalis conorhiza | 0 | 1 | 0 | 0 | 0 | 0 | 0 | 0 | 0 |
| Corydalis persica | 0 | 1 | 0 | 0 | 0 | 1 | 0 | 0 | 0 |
| Cotoneaster persicus | 0 | 0 | 0 | 1 | 1 | 0 | 0 | 0 | 0 |
| Cotoneaster zangezuricus | 0 | 1 | 0 | 0 | 0 | 0 | 0 | 0 | 0 |
| Cousinia adenosticta | 0 | 0 | 1 | 0 | 0 | 0 | 0 | 0 | 0 |
| Cousinia archibaldii | 0 | 0 | 0 | 1 | 0 | 0 | 0 | 0 | 0 |
| Cousinia bachtiarica | 0 | 0 | 1 | 1 | 0 | 0 | 0 | 0 | 0 |
| Cousinia bornmuelleri | 0 | 0 | 0 | 1 | 1 | 0 | 0 | 0 | 0 |
| Cousinia concinna | 0 | 0 | 0 | 1 | 0 | 0 | 0 | 0 | 0 |
| Cousinia crispa | 0 | 0 | 1 | 0 | 0 | 0 | 0 | 0 | 0 |
| Cousinia decumbens | 0 | 0 | 1 | 0 | 0 | 0 | 0 | 0 | 0 |
| Cousinia eburnea | 0 | 0 | 0 | 1 | 0 | 0 | 0 | 0 | 0 |
| Cousinia elwendensis | 0 | 0 | 0 | 1 | 0 | 0 | 0 | 0 | 0 |
| Cousinia fragilis | 0 | 0 | 0 | 0 | 1 | 0 | 0 | 0 | 0 |
| Cousinia gmelini | 0 | 0 | 1 | 0 | 0 | 0 | 0 | 0 | 0 |
| Cousinia harazensis | 0 | 0 | 1 | 0 | 0 | 0 | 0 | 0 | 0 |
| Cousinia irritans | 0 | 0 | 1 | 0 | 0 | 0 | 0 | 0 | 0 |
| Cousinia karkasensis | 0 | 0 | 0 | 1 | 0 | 0 | 0 | 0 | 0 |
| Cousinia lasiolepis | 0 | 0 | 0 | 1 | 0 | 0 | 0 | 0 | 0 |
| Cousinia longifolia | 0 | 0 | 0 | 1 | 1 | 0 | 0 | 0 | 0 |
| Cousinia multiloba | 0 | 0 | 1 | 1 | 1 | 0 | 0 | 0 | 0 |
| Cousinia ottonis | 0 | 0 | 0 | 1 | 0 | 0 | 0 | 0 | 0 |
| Cousinia pterocaulos | 0 | 0 | 1 | 0 | 0 | 1 | 0 | 0 | 0 |
| Cousinia satdagensis | 0 | 1 | 0 | 0 | 0 | 0 | 0 | 0 | 0 |
| Cousinia shahvarica | 0 | 0 | 1 | 0 | 0 | 0 | 0 | 0 | 0 |
| Cousinia sicigera | 0 | 0 | 0 | 1 | 1 | 0 | 0 | 0 | 0 |
| Cousinia xiphiolepis | 0 | 0 | 1 | 0 | 0 | 0 | 0 | 0 | 0 |
| Crepis armena | 1 | 1 | 0 | 0 | 0 | 0 | 0 | 0 | 0 |
| Crepis asadbarensis | 0 | 0 | 1 | 0 | 0 | 0 | 0 | 0 | 0 |
| Crepis bupleurifolia | 0 | 1 | 0 | 0 | 0 | 0 | 0 | 0 | 0 |
| Crepis conyzifolia | 0 | 1 | 0 | 0 | 0 | 0 | 0 | 0 | 0 |
| Crepis demavendi | 0 | 0 | 1 | 0 | 0 | 0 | 0 | 0 | 0 |
| Crepis dioritica | 0 | 1 | 0 | 0 | 0 | 0 | 0 | 0 | 0 |
| Crepis elbursensis | 0 | 0 | 1 | 0 | 0 | 1 | 1 | 0 | 0 |
| Crepis flexuosa | 0 | 0 | 1 | 0 | 0 | 0 | 0 | 0 | 0 |
| Crepis frigida | 1 | 1 | 1 | 0 | 0 | 0 | 0 | 0 | 0 |
| Crepis heterotricha | 0 | 0 | 1 | 1 | 1 | 0 | 0 | 0 | 0 |
| Crepis multicaulis | 0 | 0 | 1 | 0 | 0 | 0 | 0 | 0 | 0 |
| Crepis pannonica | 0 | 1 | 0 | 0 | 0 | 0 | 0 | 0 | 0 |
| Crepis sahendi | 0 | 1 | 0 | 0 | 0 | 1 | 1 | 0 | 0 |
| Crepis willdenowii | 1 | 1 | 0 | 0 | 0 | 0 | 0 | 0 | 0 |
| Crocus abracteolus | 1 | 0 | 0 | 0 | 0 | 0 | 0 | 0 | 0 |
| Crocus kotschyanus | 0 | 1 | 0 | 0 | 0 | 0 | 0 | 0 | 0 |
| Crocus scharojanii | 0 | 1 | 0 | 0 | 0 | 0 | 0 | 0 | 1 |
| Crocus vallicola | 0 | 1 | 0 | 0 | 0 | 0 | 0 | 0 | 0 |
| Cyclotrichium straussii | 0 | 0 | 0 | 1 | 0 | 0 | 0 | 0 | 0 |
| Cymbocarpum erythraeum | 0 | 1 | 0 | 0 | 0 | 0 | 0 | 0 | 0 |
| Cynoglossum holosericeum | 0 | 1 | 0 | 0 | 0 | 0 | 0 | 0 | 0 |
| Daphne glomerata | 0 | 1 | 0 | 0 | 0 | 0 | 0 | 0 | 0 |
| Daphne magakjanii | 0 | 1 | 0 | 0 | 0 | 1 | 0 | 0 | 0 |
| Daphne oleoides | 1 | 1 | 0 | 0 | 0 | 1 | 0 | 0 | 0 |
| Delphinium carduchorum | 0 | 1 | 0 | 0 | 0 | 1 | 0 | 0 | 0 |
| Delphinium elbursense | 0 | 0 | 1 | 0 | 0 | 1 | 0 | 0 | 0 |
| Delphinium flexuosum | 0 | 1 | 0 | 0 | 0 | 0 | 0 | 0 | 0 |
| Delphinium foetidum | 0 | 1 | 0 | 0 | 0 | 0 | 0 | 0 | 0 |
| Delphinium lalesaricum | 0 | 0 | 0 | 0 | 1 | 0 | 0 | 0 | 0 |
| Delphinium lanigerum | 0 | 0 | 1 | 1 | 0 | 0 | 0 | 0 | 0 |
| Delphinium linearilobum | 0 | 1 | 0 | 0 | 0 | 0 | 0 | 0 | 0 |
| Delphinium uncinatum | 0 | 0 | 0 | 0 | 1 | 0 | 0 | 0 | 0 |
| Deschampsia cespitosa | 1 | 1 | 1 | 1 | 1 | 1 | 0 | 0 | 0 |
| Dianthus balansae | 1 | 1 | 0 | 0 | 0 | 0 | 0 | 0 | 0 |
| Dianthus brevicaulis | 1 | 0 | 0 | 0 | 0 | 0 | 0 | 0 | 0 |
| Dianthus cretaceus | 0 | 1 | 1 | 1 | 0 | 1 | 1 | 0 | 0 |
| Dianthus denaicus | 0 | 0 | 0 | 1 | 0 | 0 | 0 | 0 | 0 |
| Dianthus diversifolius | 0 | 0 | 0 | 1 | 0 | 0 | 0 | 0 | 0 |
| Dianthus elymaiticus | 0 | 0 | 0 | 1 | 0 | 0 | 0 | 0 | 0 |
| Dianthus erythrocoleus | 0 | 1 | 1 | 0 | 0 | 1 | 0 | 0 | 0 |
| Dianthus goerkii | 1 | 0 | 0 | 0 | 0 | 0 | 0 | 0 | 0 |
| Dianthus lactiflorus | 1 | 0 | 0 | 0 | 0 | 0 | 0 | 0 | 0 |
| Dianthus libanotis | 0 | 1 | 1 | 1 | 0 | 1 | 1 | 0 | 0 |
| Dianthus micranthus | 1 | 1 | 0 | 0 | 0 | 0 | 0 | 0 | 1 |
| Dianthus multicaulis | 0 | 1 | 0 | 0 | 0 | 0 | 0 | 0 | 0 |
| Dianthus muschianus | 0 | 1 | 0 | 0 | 0 | 0 | 0 | 0 | 0 |
| Dianthus orientalis | 1 | 1 | 1 | 1 | 0 | 1 | 1 | 0 | 0 |
| Dianthus raddeanus | 0 | 1 | 0 | 0 | 0 | 0 | 0 | 0 | 0 |
| Dianthus recognitus | 0 | 1 | 0 | 0 | 0 | 0 | 0 | 0 | 1 |
| Dianthus sahandicus | 0 | 0 | 0 | 1 | 0 | 0 | 0 | 0 | 0 |
| Dianthus seidlitzii | 0 | 0 | 0 | 0 | 0 | 1 | 0 | 0 | 0 |
| Dianthus vanensis | 0 | 1 | 0 | 0 | 0 | 0 | 0 | 0 | 0 |
| Dichodon alborzensis | 0 | 0 | 1 | 0 | 0 | 0 | 0 | 0 | 0 |
| Didymophysa aucheri | 0 | 1 | 1 | 1 | 0 | 1 | 1 | 0 | 0 |
| Dielsiocharis kotschyi | 0 | 0 | 1 | 1 | 1 | 0 | 0 | 0 | 0 |
| Dionysia archibaldii | 0 | 0 | 0 | 1 | 0 | 0 | 0 | 0 | 0 |
| Dionysia aubrietioides | 0 | 0 | 0 | 1 | 0 | 0 | 0 | 0 | 0 |
| Dionysia caespitosa | 0 | 0 | 0 | 1 | 0 | 0 | 0 | 0 | 0 |
| Dionysia cristagalli | 0 | 0 | 0 | 1 | 0 | 0 | 0 | 0 | 0 |
| Dionysia curviflora | 0 | 0 | 0 | 1 | 0 | 0 | 0 | 0 | 0 |
| Dionysia esfandiarii | 0 | 0 | 0 | 1 | 0 | 0 | 0 | 0 | 0 |
| Dionysia iranshahrii | 0 | 0 | 0 | 1 | 0 | 0 | 0 | 0 | 0 |
| Dionysia khatamii | 0 | 0 | 0 | 1 | 0 | 0 | 0 | 0 | 0 |
| Dionysia khuzistanica | 0 | 0 | 0 | 1 | 0 | 0 | 0 | 0 | 0 |
| Dionysia leucotricha | 0 | 0 | 0 | 1 | 0 | 0 | 0 | 0 | 0 |
| Dionysia oreodoxa | 0 | 0 | 0 | 0 | 1 | 0 | 0 | 0 | 0 |
| Dionysia revoluta | 0 | 0 | 0 | 1 | 0 | 0 | 0 | 0 | 0 |
| Dionysia rhaptodes | 0 | 0 | 0 | 0 | 1 | 0 | 0 | 0 | 0 |
| Dionysia termeana | 0 | 0 | 0 | 1 | 0 | 0 | 0 | 0 | 0 |
| Dionysia zagrica | 0 | 0 | 0 | 1 | 0 | 0 | 0 | 0 | 0 |
| Dionysia zetterlundii | 0 | 0 | 0 | 1 | 0 | 0 | 0 | 0 | 0 |
| Dionysia zschummelii | 0 | 0 | 0 | 1 | 0 | 0 | 0 | 0 | 0 |
| Diplotaenia cachrydifolia | 0 | 1 | 1 | 0 | 0 | 0 | 0 | 0 | 0 |
| Diplotaenia damavandica | 0 | 0 | 1 | 0 | 0 | 0 | 0 | 0 | 0 |
| Dolichorrhiza persica | 0 | 0 | 1 | 0 | 0 | 0 | 0 | 0 | 0 |
| Dorema aucheri | 0 | 0 | 0 | 1 | 1 | 0 | 0 | 0 | 0 |
| Doronicum bracteatum | 0 | 0 | 0 | 1 | 0 | 0 | 1 | 0 | 0 |
| Doronicum dolichotrichum | 0 | 1 | 0 | 0 | 0 | 0 | 0 | 0 | 0 |
| Doronicum hakkiaricum | 0 | 1 | 0 | 0 | 0 | 0 | 0 | 0 | 0 |
| Doronicum macrophyllum | 0 | 1 | 0 | 0 | 0 | 0 | 0 | 0 | 0 |
| Doronicum maximum | 0 | 1 | 0 | 0 | 0 | 0 | 0 | 0 | 0 |
| Doronicum oblongifolium | 0 | 1 | 0 | 0 | 0 | 0 | 0 | 0 | 0 |
| Doronicum tobeyi | 0 | 1 | 0 | 0 | 0 | 0 | 0 | 0 | 0 |
| Draba araratica | 0 | 1 | 0 | 0 | 0 | 0 | 0 | 0 | 0 |
| Draba bruniifolia | 1 | 1 | 0 | 0 | 0 | 1 | 1 | 0 | 1 |
| Draba cappadocica | 1 | 1 | 0 | 0 | 0 | 0 | 0 | 0 | 0 |
| Draba nemorosa | 0 | 1 | 1 | 0 | 0 | 1 | 0 | 0 | 0 |
| Draba orientalis | 0 | 1 | 0 | 0 | 0 | 0 | 0 | 0 | 0 |
| Draba polytricha | 0 | 1 | 0 | 0 | 0 | 0 | 0 | 0 | 0 |
| Draba pulchella | 0 | 0 | 1 | 0 | 0 | 0 | 1 | 0 | 0 |
| Draba rosularis | 0 | 1 | 1 | 1 | 1 | 1 | 0 | 0 | 0 |
| Draba siliquosa | 0 | 1 | 1 | 0 | 0 | 0 | 0 | 0 | 0 |
| Draba thylacocarpa | 0 | 1 | 0 | 0 | 0 | 0 | 0 | 0 | 0 |
| Dracocephalum aucheri | 0 | 1 | 1 | 0 | 0 | 1 | 0 | 0 | 0 |
| Dracocephalum botryoides | 0 | 1 | 0 | 0 | 0 | 0 | 0 | 0 | 0 |
| Dracocephalum ghahremanii | 0 | 0 | 1 | 0 | 0 | 0 | 0 | 0 | 0 |
| Dracocephalum kotschyi | 0 | 0 | 1 | 1 | 0 | 0 | 0 | 0 | 0 |
| Dracocephalum multicaule | 1 | 1 | 1 | 0 | 0 | 1 | 0 | 0 | 0 |
| Dracocephalum polychaetum | 0 | 0 | 0 | 0 | 1 | 0 | 0 | 0 | 0 |
| Dracocephalum surmandinum | 0 | 0 | 0 | 1 | 0 | 0 | 0 | 0 | 0 |
| Drymocallis damghanensis | 0 | 0 | 1 | 0 | 0 | 0 | 0 | 0 | 0 |
| Echinophora cinerea | 0 | 0 | 0 | 1 | 0 | 0 | 0 | 0 | 0 |
| Elburzia fenestrata | 0 | 0 | 1 | 0 | 0 | 0 | 0 | 0 | 0 |
| Eleutherospermum cicutarium | 0 | 1 | 1 | 0 | 0 | 0 | 0 | 0 | 0 |
| Elymus longiaristatus | 0 | 0 | 1 | 1 | 1 | 0 | 1 | 0 | 0 |
| Elymus transhyrcanus | 0 | 1 | 0 | 0 | 0 | 0 | 0 | 0 | 0 |
| Empetrum nigrum | 0 | 1 | 0 | 0 | 0 | 0 | 0 | 0 | 0 |
| Epilobium algidum | 0 | 1 | 1 | 0 | 0 | 0 | 0 | 0 | 0 |
| Epilobium anagallidifolium | 0 | 1 | 0 | 0 | 0 | 0 | 0 | 0 | 0 |
| Epilobium frigidum | 1 | 1 | 1 | 1 | 0 | 1 | 1 | 0 | 0 |
| Epilobium palustre | 0 | 0 | 1 | 0 | 0 | 0 | 0 | 0 | 0 |
| Epilobium ponticum | 1 | 1 | 1 | 0 | 0 | 0 | 1 | 0 | 1 |
| Epilobium rechingeri | 0 | 0 | 1 | 0 | 0 | 0 | 1 | 0 | 0 |
| Eremopoa bellula | 0 | 0 | 1 | 0 | 0 | 0 | 0 | 0 | 0 |
| Eremopoa songarica | 0 | 0 | 0 | 0 | 0 | 0 | 0 | 0 | 1 |
| Eremurus persicus | 0 | 0 | 1 | 1 | 1 | 0 | 0 | 0 | 0 |
| Erigeron acris | 1 | 1 | 1 | 1 | 1 | 1 | 0 | 0 | 0 |
| Erigeron caucasicus | 0 | 1 | 1 | 0 | 0 | 1 | 0 | 0 | 0 |
| Erigeron cilicicus | 1 | 0 | 0 | 0 | 0 | 0 | 0 | 0 | 0 |
| Erigeron daenensis | 0 | 1 | 0 | 0 | 0 | 0 | 0 | 0 | 0 |
| Erigeron hyrcanicus | 0 | 0 | 1 | 0 | 0 | 0 | 0 | 0 | 0 |
| Erigeron uniflorus | 0 | 1 | 1 | 1 | 0 | 0 | 0 | 0 | 0 |
| Erigeron zederbaueri | 1 | 0 | 0 | 0 | 0 | 0 | 0 | 0 | 0 |
| Eritrichium gracillimum | 0 | 0 | 1 | 0 | 0 | 0 | 0 | 0 | 0 |
| Erodium absinthoides | 0 | 1 | 0 | 0 | 0 | 0 | 0 | 0 | 1 |
| Erodium cedrorum | 1 | 0 | 0 | 0 | 0 | 0 | 0 | 0 | 0 |
| Erodium dimorphum | 0 | 0 | 1 | 0 | 0 | 0 | 0 | 0 | 0 |
| Erodium hakkiaricum | 0 | 1 | 0 | 0 | 0 | 0 | 0 | 0 | 0 |
| Eryngium bornmuelleri | 0 | 1 | 0 | 0 | 0 | 0 | 0 | 0 | 0 |
| Eryngium ilex | 0 | 1 | 0 | 0 | 0 | 0 | 0 | 0 | 0 |
| Erysimum caespitosum | 0 | 0 | 1 | 1 | 0 | 1 | 0 | 0 | 0 |
| Erysimum damirliense | 0 | 0 | 0 | 0 | 0 | 0 | 0 | 1 | 0 |
| Erysimum elbrusense | 0 | 1 | 1 | 1 | 0 | 0 | 1 | 0 | 0 |
| Erysimum frigidum | 0 | 0 | 1 | 1 | 0 | 0 | 0 | 0 | 0 |
| Erysimum gelidum | 0 | 1 | 0 | 0 | 0 | 1 | 1 | 0 | 0 |
| Erysimum guneri | 0 | 1 | 0 | 0 | 0 | 0 | 0 | 0 | 0 |
| Erysimum hakkiaricum | 0 | 1 | 0 | 0 | 0 | 0 | 0 | 0 | 0 |
| Erysimum hezarense | 0 | 0 | 0 | 0 | 1 | 0 | 0 | 0 | 0 |
| Erysimum ikizdereense | 0 | 1 | 0 | 0 | 0 | 0 | 0 | 0 | 0 |
| Erysimum kotschyanum | 1 | 0 | 0 | 0 | 0 | 0 | 0 | 0 | 0 |
| Erysimum macrostigma | 0 | 1 | 1 | 0 | 0 | 1 | 0 | 0 | 0 |
| Erysimum munzuriense | 0 | 1 | 0 | 0 | 0 | 0 | 0 | 0 | 0 |
| Erysimum nasturtioides | 0 | 0 | 0 | 1 | 0 | 0 | 1 | 0 | 0 |
| Erysimum polatschekii | 0 | 0 | 0 | 0 | 1 | 0 | 0 | 0 | 0 |
| Erysimum rizeense | 0 | 1 | 0 | 0 | 0 | 0 | 0 | 0 | 0 |
| Erysimum sintenisianum | 1 | 1 | 0 | 0 | 0 | 0 | 0 | 0 | 0 |
| Erysimum yildirimlii | 0 | 1 | 0 | 0 | 0 | 0 | 0 | 0 | 0 |
| Euphorbia aucheri | 0 | 0 | 1 | 1 | 0 | 1 | 0 | 0 | 0 |
| Euphorbia belgheisi | 0 | 0 | 0 | 0 | 0 | 0 | 0 | 1 | 0 |
| Euphorbia erythradenia | 0 | 0 | 0 | 1 | 1 | 0 | 0 | 0 | 0 |
| Euphorbia grisophylla | 0 | 1 | 0 | 0 | 0 | 0 | 0 | 0 | 0 |
| Euphorbia hebecarpa | 0 | 0 | 0 | 1 | 1 | 1 | 1 | 0 | 0 |
| Euphorbia herniariifolia | 1 | 1 | 0 | 0 | 0 | 0 | 0 | 0 | 1 |
| Euphorbia iberica | 0 | 1 | 1 | 0 | 0 | 1 | 0 | 0 | 0 |
| Euphorbia macrocarpa | 0 | 1 | 0 | 1 | 0 | 1 | 0 | 0 | 0 |
| Euphorbia microsciadia | 0 | 0 | 1 | 1 | 1 | 0 | 0 | 0 | 0 |
| Euphorbia mirzakhaniana | 0 | 0 | 1 | 0 | 0 | 0 | 0 | 0 | 0 |
| Euphorbia plebeia | 0 | 0 | 0 | 1 | 0 | 0 | 0 | 0 | 0 |
| Euphorbia sahendi | 0 | 0 | 0 | 0 | 0 | 1 | 0 | 0 | 0 |
| Euphorbia sanasunitensis | 0 | 1 | 0 | 0 | 0 | 0 | 0 | 0 | 0 |
| Euphrasia amblyodonta | 0 | 1 | 0 | 0 | 0 | 0 | 0 | 0 | 0 |
| Euphrasia juzepczukii | 0 | 1 | 1 | 0 | 0 | 1 | 0 | 0 | 0 |
| Euphrasia petiolaris | 0 | 1 | 0 | 0 | 0 | 0 | 0 | 0 | 0 |
| Euphrasia sevanensis | 0 | 1 | 0 | 0 | 0 | 1 | 0 | 0 | 0 |
| Ferula haussknechtii | 0 | 1 | 0 | 0 | 0 | 0 | 0 | 0 | 0 |
| Ferula hezarlalehzarica | 0 | 0 | 0 | 0 | 1 | 0 | 0 | 0 | 0 |
| Ferula microcolea | 0 | 0 | 1 | 1 | 0 | 1 | 0 | 0 | 0 |
| Ferula ovina | 0 | 1 | 1 | 1 | 1 | 0 | 0 | 0 | 0 |
| Ferula setifolia | 0 | 1 | 0 | 0 | 0 | 0 | 0 | 0 | 0 |
| Ferulago angulata | 0 | 1 | 1 | 1 | 1 | 1 | 1 | 0 | 0 |
| Ferulago contracta | 0 | 0 | 0 | 1 | 0 | 0 | 0 | 0 | 0 |
| Festuca adanensis | 1 | 1 | 0 | 0 | 0 | 0 | 0 | 0 | 0 |
| Festuca airoides | 0 | 1 | 0 | 0 | 0 | 0 | 0 | 0 | 0 |
| Festuca alaica | 0 | 0 | 1 | 1 | 0 | 0 | 0 | 0 | 0 |
| Festuca anatolica | 1 | 1 | 0 | 0 | 0 | 0 | 0 | 0 | 0 |
| Festuca artvinensis | 0 | 1 | 0 | 0 | 0 | 0 | 0 | 0 | 0 |
| Festuca brunnescens | 0 | 1 | 0 | 0 | 0 | 0 | 0 | 0 | 0 |
| Festuca bushiana | 0 | 1 | 0 | 0 | 0 | 0 | 0 | 0 | 0 |
| Festuca cappadocica | 1 | 1 | 0 | 0 | 0 | 0 | 0 | 0 | 1 |
| Festuca cataonica | 1 | 0 | 0 | 0 | 0 | 0 | 0 | 0 | 1 |
| Festuca chalcophaea | 0 | 1 | 0 | 0 | 0 | 0 | 0 | 0 | 0 |
| Festuca cratericola | 1 | 0 | 0 | 0 | 0 | 0 | 0 | 0 | 0 |
| Festuca elwendiana | 0 | 1 | 0 | 0 | 0 | 0 | 0 | 0 | 0 |
| Festuca iranica | 0 | 0 | 0 | 1 | 0 | 0 | 0 | 0 | 0 |
| Festuca lazistanica | 0 | 1 | 0 | 0 | 0 | 0 | 0 | 0 | 0 |
| Festuca oreophila | 0 | 1 | 0 | 0 | 0 | 0 | 0 | 0 | 0 |
| Festuca ovina | 0 | 1 | 1 | 1 | 0 | 1 | 0 | 0 | 0 |
| Festuca pinifolia | 1 | 1 | 0 | 0 | 0 | 0 | 0 | 0 | 0 |
| Festuca rechingeri | 0 | 0 | 1 | 0 | 0 | 0 | 0 | 0 | 0 |
| Festuca sabalanica | 0 | 0 | 0 | 0 | 0 | 1 | 0 | 0 | 0 |
| Festuca sclerophylla | 0 | 1 | 0 | 0 | 0 | 0 | 0 | 0 | 0 |
| Festuca skvortsovii | 0 | 1 | 0 | 0 | 0 | 1 | 0 | 0 | 0 |
| Festuca sommieri | 0 | 1 | 0 | 0 | 0 | 0 | 0 | 0 | 0 |
| Festuca sulcata | 0 | 0 | 1 | 1 | 0 | 1 | 0 | 0 | 0 |
| Festuca varia | 1 | 1 | 0 | 0 | 0 | 0 | 0 | 0 | 0 |
| Fibigia multicaulis | 0 | 0 | 1 | 1 | 1 | 0 | 1 | 0 | 0 |
| Fibigia umbellata | 0 | 0 | 1 | 1 | 1 | 0 | 0 | 0 | 0 |
| Fritillaria alburyana | 0 | 1 | 0 | 0 | 0 | 0 | 0 | 0 | 0 |
| Fritillaria aurea | 1 | 1 | 0 | 0 | 0 | 0 | 0 | 0 | 0 |
| Fritillaria caucasica | 0 | 1 | 0 | 0 | 0 | 1 | 0 | 0 | 0 |
| Fritillaria chlorantha | 0 | 0 | 0 | 1 | 0 | 0 | 0 | 0 | 0 |
| Fritillaria crassifolia | 0 | 1 | 0 | 1 | 0 | 1 | 1 | 0 | 0 |
| Fritillaria kotschyana | 0 | 0 | 1 | 0 | 0 | 0 | 0 | 0 | 0 |
| Fritillaria latifolia | 0 | 1 | 0 | 0 | 0 | 0 | 0 | 0 | 0 |
| Fritillaria michailovskyi | 0 | 1 | 0 | 0 | 0 | 0 | 0 | 0 | 0 |
| Fritillaria minima | 0 | 1 | 0 | 0 | 0 | 0 | 0 | 0 | 0 |
| Fritillaria minuta | 0 | 1 | 0 | 0 | 0 | 0 | 0 | 0 | 0 |
| Fritillaria olivieri | 0 | 0 | 0 | 1 | 0 | 0 | 0 | 0 | 0 |
| Fritillaria reuteri | 0 | 0 | 0 | 1 | 0 | 0 | 0 | 0 | 0 |
| Fritillaria zagrica | 0 | 0 | 1 | 1 | 0 | 1 | 0 | 0 | 0 |
| Fuernrohria setifolia | 0 | 1 | 0 | 0 | 0 | 0 | 0 | 0 | 0 |
| Gagea alexeenkoana | 0 | 1 | 1 | 1 | 0 | 1 | 0 | 0 | 0 |
| Gagea alexii | 0 | 0 | 0 | 0 | 1 | 0 | 0 | 0 | 0 |
| Gagea anisanthos | 0 | 1 | 0 | 0 | 0 | 0 | 0 | 0 | 0 |
| Gagea capillifolia | 0 | 0 | 1 | 0 | 0 | 0 | 0 | 0 | 0 |
| Gagea caroli-kochii | 0 | 1 | 1 | 0 | 0 | 0 | 0 | 0 | 0 |
| Gagea confusa | 1 | 1 | 1 | 1 | 0 | 1 | 0 | 0 | 0 |
| Gagea dschungarica | 0 | 0 | 1 | 1 | 1 | 0 | 0 | 0 | 0 |
| Gagea exilis | 0 | 0 | 0 | 0 | 1 | 0 | 0 | 0 | 0 |
| Gagea glacialis | 1 | 1 | 0 | 0 | 0 | 0 | 0 | 0 | 0 |
| Gagea joannis | 0 | 1 | 0 | 0 | 0 | 0 | 0 | 0 | 0 |
| Gagea luteoides | 1 | 1 | 0 | 0 | 0 | 0 | 0 | 0 | 0 |
| Gagea sulfurea | 0 | 1 | 0 | 0 | 0 | 0 | 0 | 0 | 0 |
| Gagea uliginosa | 1 | 1 | 1 | 0 | 0 | 1 | 0 | 0 | 0 |
| Galium aladaghense | 1 | 0 | 0 | 0 | 0 | 0 | 0 | 0 | 0 |
| Galium aucheri | 0 | 0 | 1 | 0 | 0 | 0 | 0 | 0 | 0 |
| Galium boreale | 0 | 1 | 1 | 0 | 0 | 1 | 0 | 0 | 0 |
| Galium decumbens | 0 | 0 | 1 | 0 | 0 | 1 | 0 | 0 | 0 |
| Galium delicatulum | 0 | 0 | 1 | 0 | 0 | 0 | 0 | 0 | 0 |
| Galium hyrcanicum | 0 | 1 | 1 | 0 | 0 | 1 | 0 | 0 | 0 |
| Galium majmechense | 0 | 1 | 0 | 0 | 0 | 0 | 0 | 0 | 0 |
| Galium nabelekii | 0 | 1 | 0 | 0 | 0 | 0 | 0 | 0 | 0 |
| Galium nigdeense | 1 | 0 | 0 | 0 | 0 | 0 | 0 | 0 | 0 |
| Galium ovitdaghense | 0 | 1 | 0 | 0 | 0 | 0 | 0 | 0 | 0 |
| Galium pseudokurdicum | 0 | 0 | 0 | 1 | 0 | 0 | 1 | 0 | 0 |
| Galium schoenbeck-Temesyae | 0 | 0 | 0 | 1 | 0 | 0 | 0 | 0 | 0 |
| Galium subvelutinum | 0 | 1 | 1 | 1 | 0 | 1 | 0 | 1 | 0 |
| Galium tuncelianum | 0 | 1 | 0 | 0 | 0 | 0 | 0 | 0 | 0 |
| Galium valantioides | 0 | 1 | 0 | 0 | 0 | 0 | 0 | 0 | 0 |
| Gentiana aquatica | 0 | 1 | 1 | 0 | 1 | 0 | 0 | 0 | 0 |
| Gentiana boissieri | 1 | 0 | 0 | 0 | 0 | 0 | 0 | 0 | 0 |
| Gentiana brachyphylla | 1 | 0 | 0 | 0 | 0 | 0 | 0 | 0 | 0 |
| Gentiana gelida | 0 | 1 | 0 | 0 | 0 | 1 | 0 | 0 | 0 |
| Gentiana nivalis | 0 | 1 | 0 | 0 | 0 | 0 | 0 | 0 | 0 |
| Gentiana pyrenaica | 0 | 1 | 0 | 0 | 0 | 0 | 0 | 0 | 0 |
| Gentiana riparia | 0 | 0 | 0 | 0 | 1 | 0 | 0 | 0 | 0 |
| Gentiana septemfida | 1 | 1 | 1 | 0 | 0 | 0 | 0 | 0 | 0 |
| Gentiana umbellata | 0 | 1 | 1 | 0 | 0 | 1 | 0 | 0 | 0 |
| Gentiana verna | 0 | 1 | 1 | 0 | 1 | 0 | 1 | 0 | 0 |
| Gentianella caucasea | 0 | 1 | 0 | 0 | 0 | 0 | 0 | 0 | 1 |
| Gentianella holosteoides | 1 | 0 | 0 | 0 | 0 | 0 | 0 | 0 | 0 |
| Gentianella umbellata | 0 | 1 | 0 | 0 | 0 | 0 | 0 | 0 | 0 |
| Geranium cinereum | 1 | 1 | 0 | 0 | 0 | 0 | 0 | 0 | 0 |
| Geranium kurdicum | 0 | 1 | 0 | 0 | 0 | 0 | 0 | 0 | 0 |
| Geranium persicum | 0 | 1 | 1 | 1 | 0 | 1 | 0 | 0 | 0 |
| Geranium platypetalum | 0 | 1 | 0 | 0 | 0 | 0 | 0 | 0 | 0 |
| Geum kokanikum | 0 | 0 | 1 | 0 | 0 | 0 | 0 | 0 | 0 |
| Globularia trichosantha | 1 | 0 | 0 | 0 | 0 | 0 | 0 | 0 | 0 |
| Gnaphalium leucopilinum | 1 | 1 | 0 | 0 | 0 | 0 | 0 | 0 | 0 |
| Gnaphalium stewartii | 1 | 1 | 0 | 0 | 0 | 0 | 0 | 0 | 0 |
| Gnaphalium supinum | 0 | 1 | 1 | 0 | 0 | 1 | 0 | 0 | 0 |
| Graellsia isfahan | 0 | 0 | 0 | 1 | 0 | 0 | 0 | 0 | 0 |
| Graellsia saxifragifolia | 0 | 0 | 1 | 1 | 1 | 0 | 0 | 0 | 0 |
| Graellsia stylosa | 0 | 0 | 1 | 0 | 0 | 0 | 0 | 0 | 0 |
| Gypsophila adenophylla | 0 | 1 | 0 | 0 | 0 | 0 | 0 | 0 | 0 |
| Gypsophila aretioides | 0 | 1 | 1 | 0 | 0 | 0 | 0 | 0 | 0 |
| Gypsophila briquetiana | 0 | 1 | 0 | 0 | 0 | 0 | 0 | 0 | 0 |
| Gypsophila graminifolia | 0 | 0 | 0 | 0 | 0 | 1 | 0 | 0 | 0 |
| Gypsophila hakkiarica | 0 | 1 | 0 | 0 | 0 | 0 | 0 | 0 | 0 |
| Gypsophila lipskyi | 0 | 1 | 0 | 0 | 0 | 0 | 0 | 0 | 0 |
| Gypsophila nabelaelekii | 0 | 1 | 0 | 0 | 0 | 0 | 0 | 0 | 0 |
| Gypsophila peshmenii | 0 | 1 | 0 | 0 | 0 | 0 | 0 | 0 | 0 |
| Gypsophila serpylloides | 1 | 0 | 0 | 0 | 0 | 0 | 0 | 0 | 0 |
| Gypsophila silenoides | 0 | 1 | 0 | 0 | 0 | 0 | 0 | 0 | 0 |
| Gypsophila tenuifolia | 0 | 1 | 0 | 0 | 0 | 0 | 0 | 0 | 0 |
| Gypsophila venusta | 0 | 1 | 0 | 0 | 0 | 0 | 0 | 0 | 0 |
| Gypsophila yazdiana | 0 | 0 | 0 | 1 | 0 | 0 | 0 | 0 | 0 |
| Haussknechtia elymaitica | 0 | 0 | 0 | 1 | 0 | 0 | 0 | 0 | 0 |
| Hedysarum caucasicum | 0 | 1 | 0 | 0 | 0 | 0 | 0 | 0 | 0 |
| Hedysarum erythroleucum | 1 | 1 | 0 | 0 | 0 | 0 | 0 | 0 | 0 |
| Hedysarum hedysaroides | 0 | 1 | 0 | 0 | 0 | 0 | 0 | 0 | 0 |
| Hedysarum persicum | 0 | 0 | 1 | 0 | 0 | 0 | 0 | 0 | 0 |
| Hedysarum vanense | 0 | 1 | 0 | 0 | 0 | 1 | 0 | 0 | 0 |
| Heldreichia bupleurifolia | 1 | 1 | 0 | 0 | 0 | 0 | 0 | 0 | 0 |
| Heldreichia rotundifolia | 1 | 1 | 0 | 0 | 0 | 0 | 0 | 0 | 0 |
| Helichrysum athanaton | 0 | 0 | 0 | 1 | 0 | 0 | 0 | 0 | 0 |
| Helichrysum chionophilum | 1 | 0 | 0 | 0 | 0 | 0 | 0 | 0 | 0 |
| Helichrysum davisianum | 0 | 0 | 0 | 1 | 0 | 0 | 0 | 0 | 0 |
| Helichrysum oligocephalum | 0 | 0 | 1 | 1 | 0 | 1 | 0 | 1 | 0 |
| Helichrysum pallasii | 1 | 1 | 0 | 0 | 0 | 0 | 0 | 0 | 0 |
| Helichrysum psychrophilum | 0 | 1 | 1 | 0 | 0 | 1 | 1 | 1 | 0 |
| Helichrysum yurterianum | 0 | 1 | 0 | 0 | 0 | 0 | 0 | 0 | 0 |
| Helictotrichon argaeum | 1 | 1 | 0 | 0 | 0 | 0 | 0 | 0 | 0 |
| Helictotrichon versicolor | 0 | 1 | 0 | 0 | 0 | 0 | 0 | 0 | 0 |
| Heracleum anisactis | 0 | 0 | 1 | 0 | 0 | 1 | 0 | 0 | 0 |
| Heracleum apiifolium | 0 | 1 | 0 | 0 | 0 | 0 | 0 | 0 | 0 |
| Heracleum crenatifolium | 0 | 1 | 0 | 0 | 0 | 0 | 0 | 0 | 0 |
| Heracleum humile | 1 | 1 | 0 | 0 | 0 | 0 | 0 | 0 | 1 |
| Heracleum pastinacifolium | 0 | 1 | 0 | 0 | 0 | 1 | 0 | 0 | 0 |
| Heracleum rawianum | 0 | 1 | 0 | 0 | 0 | 1 | 1 | 0 | 0 |
| Heracleum schelkovnikovii | 0 | 1 | 0 | 0 | 0 | 0 | 0 | 0 | 0 |
| Heracleum sphondylium | 0 | 1 | 0 | 0 | 0 | 0 | 0 | 0 | 0 |
| Herniaria argaea | 1 | 1 | 0 | 0 | 0 | 0 | 0 | 0 | 0 |
| Herniaria caucasica | 0 | 1 | 1 | 0 | 0 | 0 | 0 | 0 | 0 |
| Herniaria incana | 1 | 1 | 1 | 1 | 0 | 1 | 1 | 0 | 1 |
| Herniaria olympica | 1 | 1 | 0 | 0 | 0 | 0 | 0 | 0 | 1 |
| Hesperis borbasii | 0 | 0 | 0 | 1 | 0 | 0 | 0 | 0 | 0 |
| Hesperis leucoclada | 0 | 0 | 0 | 1 | 0 | 0 | 0 | 0 | 0 |
| Hesperis luristanica | 0 | 0 | 0 | 1 | 0 | 0 | 0 | 0 | 0 |
| Hesperis nivalis | 0 | 0 | 0 | 1 | 0 | 0 | 0 | 0 | 0 |
| Hieracium caucasicum | 0 | 1 | 1 | 0 | 0 | 1 | 0 | 0 | 0 |
| Hieracium echioides | 1 | 1 | 0 | 0 | 0 | 0 | 0 | 0 | 0 |
| Hieracium prenanthoides | 0 | 0 | 1 | 0 | 0 | 1 | 0 | 0 | 0 |
| Hieracium procerum | 0 | 1 | 1 | 0 | 0 | 1 | 1 | 0 | 0 |
| Hieracium rigens | 0 | 1 | 0 | 0 | 0 | 0 | 0 | 0 | 0 |
| Hieracium teberdense | 0 | 1 | 0 | 0 | 0 | 0 | 0 | 0 | 0 |
| Hordeum violaceum | 0 | 1 | 1 | 1 | 1 | 1 | 0 | 0 | 0 |
| Hyalopoa hracziana | 0 | 1 | 0 | 0 | 0 | 0 | 0 | 0 | 0 |
| Hyalopoa pontica | 0 | 1 | 0 | 0 | 0 | 0 | 0 | 0 | 0 |
| Hymenocrater yazdianus | 0 | 0 | 0 | 1 | 0 | 0 | 0 | 0 | 0 |
| Hyoscyamus kotschyanus | 0 | 0 | 0 | 1 | 0 | 0 | 0 | 0 | 0 |
| Hyoscyamus kurdicus | 0 | 0 | 1 | 0 | 0 | 1 | 1 | 0 | 0 |
| Hyoscyamus senecionis | 0 | 1 | 1 | 1 | 1 | 0 | 0 | 0 | 0 |
| Hypericum armenum | 0 | 1 | 1 | 0 | 0 | 1 | 0 | 0 | 0 |
| Hypericum crenulatum | 1 | 0 | 0 | 0 | 0 | 0 | 0 | 0 | 0 |
| Hypericum linarioides | 1 | 1 | 1 | 0 | 0 | 1 | 0 | 0 | 1 |
| Hypericum musadoganii | 1 | 0 | 0 | 0 | 0 | 0 | 0 | 0 | 0 |
| Hypericum nummularioides | 0 | 1 | 0 | 0 | 0 | 0 | 0 | 0 | 0 |
| Inula acaulis | 1 | 1 | 1 | 0 | 0 | 0 | 0 | 0 | 0 |
| Inula mariae | 0 | 1 | 0 | 0 | 0 | 0 | 0 | 0 | 0 |
| Inula orientalis | 0 | 1 | 0 | 0 | 0 | 0 | 0 | 0 | 0 |
| Inula rhizocephala | 0 | 0 | 1 | 0 | 1 | 0 | 0 | 0 | 0 |
| Iranecio elbrusensis | 0 | 0 | 1 | 0 | 0 | 0 | 0 | 0 | 0 |
| Iranecio oligolepis | 0 | 0 | 1 | 0 | 0 | 0 | 0 | 0 | 0 |
| Iranecio paucilobus | 0 | 1 | 1 | 1 | 0 | 0 | 1 | 0 | 0 |
| Iris barnumiae | 0 | 1 | 1 | 0 | 0 | 1 | 1 | 0 | 0 |
| Isatis brachycarpa | 0 | 1 | 0 | 0 | 0 | 0 | 0 | 0 | 0 |
| Isatis nummularia | 0 | 1 | 0 | 0 | 0 | 0 | 0 | 0 | 0 |
| Isatis takhtajanii | 1 | 1 | 0 | 1 | 0 | 0 | 0 | 0 | 0 |
| Jasione supina | 1 | 1 | 0 | 0 | 0 | 0 | 0 | 0 | 1 |
| Johrenia alpina | 1 | 0 | 0 | 0 | 0 | 0 | 0 | 0 | 0 |
| Johreniopsis scoparia | 0 | 0 | 1 | 1 | 0 | 0 | 0 | 0 | 0 |
| Johreniopsis seseloides | 0 | 0 | 1 | 1 | 0 | 1 | 0 | 0 | 0 |
| Juncus alpigenus | 0 | 1 | 0 | 0 | 0 | 0 | 0 | 0 | 0 |
| Juncus filiformis | 0 | 1 | 0 | 0 | 0 | 0 | 0 | 0 | 0 |
| Juncus rechingeri | 0 | 1 | 1 | 1 | 0 | 0 | 0 | 0 | 0 |
| Juniperus communis | 0 | 1 | 1 | 0 | 0 | 1 | 0 | 0 | 0 |
| Juniperus sabina | 0 | 1 | 1 | 0 | 0 | 0 | 0 | 0 | 0 |
| Jurinea meda | 0 | 0 | 0 | 1 | 0 | 0 | 0 | 0 | 0 |
| Jurinea viciosoi | 0 | 0 | 0 | 1 | 0 | 0 | 0 | 0 | 0 |
| Jurinella frigida | 0 | 0 | 1 | 0 | 0 | 0 | 0 | 0 | 0 |
| Jurinella microcephala | 0 | 0 | 1 | 0 | 0 | 0 | 0 | 0 | 0 |
| Jurinella moschus | 1 | 1 | 1 | 0 | 0 | 1 | 1 | 0 | 0 |
| Kelussia odoratissima | 0 | 0 | 0 | 1 | 0 | 0 | 0 | 0 | 0 |
| Kobresia humilis | 0 | 0 | 0 | 0 | 1 | 1 | 1 | 0 | 0 |
| Kobresia schoenoides | 0 | 1 | 0 | 0 | 0 | 1 | 0 | 0 | 0 |
| Kobresia simpliciuscula | 1 | 1 | 0 | 0 | 0 | 0 | 0 | 0 | 0 |
| Koeleria eriostachya | 0 | 1 | 0 | 0 | 0 | 1 | 0 | 0 | 0 |
| Lactuca denaensis | 0 | 0 | 0 | 1 | 0 | 0 | 0 | 0 | 0 |
| Lactuca hazaranensis | 0 | 0 | 0 | 0 | 1 | 0 | 0 | 0 | 0 |
| Lactuca polyclada | 0 | 0 | 1 | 1 | 0 | 0 | 0 | 0 | 0 |
| Lactuca pumila | 0 | 0 | 0 | 0 | 1 | 0 | 0 | 0 | 0 |
| Lactuca scarioloides | 0 | 1 | 1 | 1 | 0 | 1 | 1 | 0 | 0 |
| Lagochilus kotschyanus | 0 | 0 | 1 | 0 | 0 | 0 | 1 | 0 | 0 |
| Lallemantia canescens | 0 | 0 | 0 | 0 | 0 | 1 | 0 | 0 | 0 |
| Lamium armenum | 0 | 1 | 0 | 0 | 0 | 0 | 0 | 0 | 0 |
| Lamium crinitum | 0 | 1 | 0 | 0 | 0 | 0 | 0 | 0 | 0 |
| Lamium eriocephalum | 1 | 0 | 0 | 0 | 0 | 0 | 0 | 0 | 0 |
| Lamium tomentosum | 0 | 1 | 1 | 0 | 0 | 0 | 1 | 0 | 0 |
| Laserpitium carduchorum | 0 | 1 | 0 | 0 | 0 | 0 | 0 | 0 | 0 |
| Lathyrus bitlisicus | 0 | 1 | 0 | 0 | 0 | 0 | 0 | 0 | 0 |
| Lathyrus brachypterus | 0 | 1 | 0 | 0 | 0 | 0 | 0 | 0 | 0 |
| Lathyrus cyaneus | 0 | 1 | 0 | 0 | 0 | 0 | 0 | 0 | 0 |
| Lathyrus nivalis | 0 | 1 | 0 | 0 | 0 | 0 | 0 | 0 | 0 |
| Leontodon oxylepis | 1 | 0 | 0 | 0 | 0 | 0 | 0 | 0 | 0 |
| Leontodon stenocalathius | 0 | 0 | 1 | 0 | 0 | 0 | 0 | 0 | 0 |
| Leonurus cardiaca | 0 | 0 | 1 | 0 | 0 | 0 | 0 | 0 | 0 |
| Lepechiniella fursei | 0 | 0 | 1 | 0 | 0 | 0 | 0 | 0 | 0 |
| Lepechiniella persica | 0 | 0 | 1 | 0 | 0 | 0 | 0 | 0 | 0 |
| Lepidium pabotii | 0 | 0 | 0 | 1 | 0 | 0 | 0 | 0 | 0 |
| Leucopoa pseudosclerophylla | 0 | 0 | 0 | 0 | 1 | 0 | 0 | 0 | 0 |
| Leutea cupularis | 0 | 0 | 1 | 1 | 0 | 0 | 0 | 0 | 0 |
| Leutea petiolaris | 0 | 0 | 1 | 1 | 0 | 1 | 0 | 0 | 0 |
| Leutea rechingeri | 0 | 0 | 0 | 0 | 0 | 0 | 1 | 0 | 0 |
| Levisticum officinale | 0 | 0 | 0 | 0 | 1 | 0 | 0 | 0 | 0 |
| Ligularia persica | 0 | 0 | 1 | 0 | 0 | 0 | 0 | 0 | 0 |
| Ligularia sibirica | 0 | 1 | 0 | 0 | 0 | 0 | 0 | 0 | 0 |
| Ligusticum alatum | 0 | 1 | 0 | 0 | 0 | 0 | 0 | 0 | 0 |
| Linaria karajensis | 0 | 0 | 1 | 0 | 0 | 0 | 0 | 0 | 0 |
| Linaria remotiflora | 0 | 0 | 0 | 1 | 1 | 0 | 0 | 0 | 0 |
| Linaria schelkownikowii | 0 | 1 | 0 | 0 | 0 | 0 | 0 | 0 | 0 |
| Linaria shahroudensis | 0 | 0 | 1 | 0 | 0 | 0 | 0 | 0 | 0 |
| Linum densiflorum | 0 | 1 | 0 | 0 | 0 | 0 | 0 | 0 | 0 |
| Linum empetrifolium | 1 | 0 | 0 | 0 | 0 | 0 | 0 | 0 | 0 |
| Linum hypericifolium | 0 | 1 | 0 | 0 | 0 | 0 | 0 | 0 | 0 |
| Linum meletonis | 0 | 1 | 0 | 0 | 0 | 0 | 0 | 0 | 0 |
| Linum obtusatum | 1 | 1 | 0 | 0 | 0 | 0 | 0 | 0 | 0 |
| Linum punctatum | 0 | 1 | 0 | 0 | 0 | 1 | 1 | 0 | 0 |
| Linum subbiflorum | 0 | 1 | 0 | 0 | 0 | 0 | 0 | 0 | 0 |
| Linum triflorum | 0 | 1 | 0 | 0 | 0 | 0 | 0 | 0 | 0 |
| Lomatogonium carinthiacum | 0 | 1 | 0 | 0 | 0 | 1 | 0 | 0 | 0 |
| Lophanthus turcicus | 0 | 1 | 0 | 0 | 0 | 0 | 0 | 0 | 0 |
| Lotus corniculatus | 1 | 1 | 0 | 0 | 0 | 0 | 0 | 0 | 1 |
| Luzula luzulina | 0 | 1 | 0 | 0 | 0 | 0 | 0 | 0 | 0 |
| Luzula spicata | 1 | 1 | 0 | 0 | 0 | 1 | 0 | 0 | 1 |
| Luzula stenophylla | 1 | 1 | 0 | 0 | 0 | 0 | 0 | 0 | 1 |
| Malabaila dasyantha | 0 | 1 | 0 | 0 | 0 | 0 | 0 | 0 | 0 |
| Marrubium astracanicum | 1 | 1 | 1 | 1 | 0 | 1 | 1 | 0 | 1 |
| Marrubium cordatum | 0 | 1 | 0 | 0 | 0 | 1 | 1 | 0 | 0 |
| Marrubium eriocephalum | 0 | 0 | 0 | 0 | 0 | 0 | 1 | 0 | 0 |
| Marrubium heterodon | 1 | 0 | 0 | 0 | 0 | 0 | 0 | 0 | 0 |
| Mattiastrum pygmaeum | 0 | 0 | 1 | 0 | 0 | 0 | 0 | 0 | 0 |
| Melica altissima | 0 | 1 | 0 | 0 | 0 | 0 | 0 | 0 | 0 |
| Mesostemma kotschyana | 0 | 1 | 1 | 1 | 0 | 0 | 0 | 0 | 0 |
| Micrantha multicaulis | 0 | 0 | 0 | 1 | 0 | 0 | 0 | 0 | 0 |
| Microsisymbrium minutiflorum | 0 | 0 | 1 | 1 | 0 | 0 | 0 | 0 | 0 |
| Milium schmidtianum | 0 | 0 | 0 | 0 | 0 | 1 | 0 | 0 | 0 |
| Minuartia aizoides | 0 | 1 | 0 | 0 | 0 | 1 | 0 | 0 | 0 |
| Minuartia aucheriana | 0 | 0 | 0 | 1 | 0 | 0 | 0 | 0 | 0 |
| Minuartia circassica | 0 | 1 | 0 | 0 | 0 | 0 | 0 | 0 | 0 |
| Minuartia dianthifolia | 1 | 1 | 0 | 0 | 0 | 0 | 0 | 0 | 0 |
| Minuartia glandulosa | 0 | 1 | 1 | 1 | 0 | 1 | 1 | 0 | 0 |
| Minuartia hamzaoglui | 1 | 0 | 0 | 0 | 0 | 0 | 0 | 0 | 0 |
| Minuartia imbricata | 0 | 1 | 0 | 0 | 0 | 0 | 0 | 0 | 0 |
| Minuartia lineata | 0 | 1 | 1 | 0 | 0 | 1 | 0 | 0 | 0 |
| Minuartia litwinowii | 0 | 0 | 1 | 0 | 0 | 0 | 0 | 0 | 0 |
| Minuartia oreina | 1 | 1 | 1 | 1 | 0 | 1 | 1 | 0 | 0 |
| Minuartia rimarum | 1 | 1 | 0 | 0 | 0 | 0 | 0 | 0 | 0 |
| Minuartia sabalanica | 0 | 0 | 0 | 0 | 0 | 1 | 0 | 0 | 0 |
| Minuartia sublineata | 0 | 0 | 0 | 1 | 0 | 1 | 1 | 0 | 0 |
| Minuartia umbellulifera | 1 | 1 | 0 | 0 | 0 | 0 | 0 | 0 | 0 |
| Minuartia verna | 0 | 1 | 0 | 0 | 0 | 0 | 0 | 0 | 0 |
| Muscari anatolicum | 1 | 0 | 0 | 0 | 0 | 0 | 0 | 0 | 0 |
| Muscari bourgaei | 1 | 0 | 0 | 0 | 0 | 0 | 0 | 0 | 1 |
| Muscari coeleste | 1 | 1 | 0 | 0 | 0 | 0 | 0 | 0 | 0 |
| Myopordon aucheri | 0 | 0 | 0 | 1 | 0 | 0 | 0 | 0 | 0 |
| Myopordon damavandica | 0 | 0 | 1 | 0 | 0 | 0 | 0 | 0 | 0 |
| Myopordon hyrcanum | 0 | 0 | 1 | 0 | 0 | 0 | 0 | 0 | 0 |
| Myopordon persicum | 0 | 0 | 0 | 1 | 0 | 0 | 0 | 0 | 0 |
| Myosotis alpestris | 0 | 1 | 0 | 0 | 0 | 0 | 1 | 0 | 0 |
| Myosotis asiatica | 0 | 0 | 1 | 0 | 0 | 1 | 0 | 0 | 0 |
| Myosotis guneri | 0 | 1 | 0 | 0 | 0 | 0 | 0 | 0 | 0 |
| Myosotis olympica | 0 | 1 | 1 | 1 | 0 | 1 | 0 | 0 | 1 |
| Myosotis platyphylla | 0 | 1 | 0 | 0 | 0 | 0 | 0 | 0 | 0 |
| Nardus stricta | 1 | 1 | 0 | 0 | 0 | 1 | 0 | 0 | 1 |
| Nepeta alaghezi | 0 | 1 | 0 | 0 | 0 | 0 | 0 | 0 | 0 |
| Nepeta allotria | 0 | 0 | 1 | 0 | 0 | 0 | 0 | 0 | 0 |
| Nepeta archibaldii | 0 | 0 | 0 | 1 | 0 | 0 | 0 | 0 | 0 |
| Nepeta assurgens | 0 | 0 | 0 | 0 | 1 | 0 | 0 | 0 | 0 |
| Nepeta azadkouhensis | 0 | 0 | 1 | 0 | 0 | 0 | 0 | 0 | 0 |
| Nepeta binaloudensis | 0 | 0 | 1 | 0 | 0 | 0 | 0 | 0 | 0 |
| Nepeta bornmuelleri | 0 | 0 | 0 | 0 | 1 | 0 | 0 | 0 | 0 |
| Nepeta chionophila | 0 | 0 | 0 | 1 | 0 | 0 | 0 | 0 | 0 |
| Nepeta crispa | 0 | 0 | 1 | 1 | 0 | 0 | 0 | 0 | 0 |
| Nepeta daenensis | 0 | 0 | 1 | 1 | 0 | 0 | 0 | 0 | 0 |
| Nepeta dschuparensis | 0 | 0 | 0 | 1 | 1 | 0 | 0 | 0 | 0 |
| Nepeta elymaitica | 0 | 0 | 0 | 1 | 0 | 0 | 1 | 0 | 0 |
| Nepeta glomerulosa | 0 | 0 | 1 | 1 | 1 | 0 | 0 | 0 | 0 |
| Nepeta iranshahrii | 0 | 0 | 0 | 1 | 0 | 0 | 0 | 0 | 0 |
| Nepeta lamiifolia | 0 | 1 | 0 | 0 | 0 | 0 | 0 | 0 | 0 |
| Nepeta lasiocephala | 0 | 0 | 0 | 1 | 1 | 0 | 0 | 0 | 0 |
| Nepeta macrosiphon | 0 | 1 | 0 | 1 | 0 | 1 | 1 | 0 | 0 |
| Nepeta menthoides | 0 | 0 | 1 | 0 | 0 | 1 | 1 | 0 | 0 |
| Nepeta monocephala | 0 | 0 | 0 | 1 | 0 | 0 | 0 | 0 | 0 |
| Nepeta natanzensis | 0 | 0 | 0 | 1 | 1 | 0 | 0 | 0 | 0 |
| Nepeta oxyodonta | 0 | 0 | 0 | 1 | 0 | 0 | 0 | 0 | 0 |
| Nepeta pilinux | 1 | 0 | 0 | 0 | 0 | 0 | 0 | 0 | 0 |
| Nepeta pogonosperma | 0 | 0 | 1 | 0 | 0 | 0 | 0 | 1 | 0 |
| Nepeta racemosa | 0 | 1 | 1 | 0 | 0 | 1 | 0 | 0 | 0 |
| Nepeta rivularis | 0 | 0 | 0 | 0 | 1 | 0 | 0 | 0 | 0 |
| Nepeta sahandica | 0 | 0 | 1 | 0 | 0 | 1 | 0 | 0 | 0 |
| Nepeta sessilifolia | 0 | 0 | 0 | 1 | 0 | 0 | 0 | 0 | 0 |
| Nepeta stenantha | 0 | 1 | 1 | 0 | 0 | 0 | 0 | 0 | 0 |
| Nepeta supina | 0 | 1 | 0 | 0 | 0 | 0 | 0 | 0 | 0 |
| Nonea macrantha | 0 | 1 | 0 | 0 | 0 | 0 | 0 | 0 | 0 |
| Nonea persica | 0 | 1 | 1 | 1 | 1 | 1 | 1 | 0 | 0 |
| Nonea pulmonarioides | 0 | 1 | 0 | 0 | 0 | 0 | 0 | 0 | 0 |
| Odontites aucheri | 0 | 1 | 1 | 1 | 0 | 1 | 1 | 0 | 0 |
| Omphalodes luciliae | 1 | 1 | 0 | 1 | 0 | 0 | 1 | 0 | 0 |
| Onobrychis argaea | 1 | 0 | 0 | 0 | 0 | 0 | 0 | 0 | 0 |
| Onobrychis arnacantha | 0 | 0 | 1 | 0 | 0 | 0 | 0 | 0 | 0 |
| Onobrychis cornuta | 1 | 1 | 1 | 1 | 1 | 1 | 1 | 0 | 0 |
| Onobrychis garinensis | 0 | 0 | 0 | 1 | 0 | 0 | 0 | 0 | 0 |
| Onobrychis marashensis | 1 | 0 | 0 | 0 | 0 | 0 | 0 | 0 | 0 |
| Onobrychis oxytropoides | 0 | 1 | 0 | 0 | 0 | 0 | 0 | 0 | 0 |
| Onobrychis plantago | 0 | 0 | 0 | 1 | 1 | 0 | 0 | 0 | 0 |
| Onobrychis transcaucasica | 0 | 1 | 0 | 0 | 0 | 0 | 0 | 0 | 0 |
| Ononis sessilifolia | 1 | 0 | 0 | 0 | 0 | 0 | 0 | 0 | 0 |
| Onosma ghahremanii | 0 | 0 | 1 | 0 | 0 | 0 | 0 | 0 | 0 |
| Onosma haussknechtii | 0 | 1 | 0 | 0 | 0 | 0 | 0 | 0 | 0 |
| Onosma kilouyensis | 0 | 0 | 1 | 1 | 0 | 0 | 0 | 0 | 0 |
| Onosma liparioides | 0 | 1 | 0 | 0 | 0 | 0 | 0 | 0 | 0 |
| Onosma mirabilis | 0 | 1 | 0 | 0 | 0 | 0 | 0 | 0 | 0 |
| Onosma moussavi | 0 | 0 | 0 | 0 | 0 | 1 | 0 | 0 | 0 |
| Onosma proballanthera | 0 | 1 | 0 | 0 | 0 | 0 | 0 | 0 | 0 |
| Onosma sabalanica | 0 | 0 | 0 | 0 | 0 | 1 | 0 | 0 | 0 |
| Onosma stenosiphon | 0 | 0 | 1 | 1 | 1 | 0 | 0 | 0 | 0 |
| Oreopoa anatolica | 1 | 0 | 0 | 0 | 0 | 0 | 0 | 0 | 0 |
| Orobanche gamosepala | 0 | 1 | 0 | 0 | 0 | 0 | 0 | 0 | 0 |
| Oxyria digyna | 1 | 1 | 1 | 0 | 0 | 1 | 1 | 0 | 1 |
| Oxytropis aellenii | 0 | 0 | 1 | 0 | 0 | 0 | 0 | 0 | 0 |
| Oxytropis albana | 1 | 1 | 0 | 0 | 0 | 0 | 0 | 0 | 0 |
| Oxytropis armeniaca | 0 | 1 | 0 | 0 | 0 | 0 | 0 | 0 | 0 |
| Oxytropis caraganetorum | 0 | 0 | 0 | 1 | 0 | 0 | 0 | 0 | 0 |
| Oxytropis cinerea | 0 | 0 | 1 | 0 | 0 | 0 | 0 | 0 | 0 |
| Oxytropis compacta | 0 | 0 | 1 | 0 | 0 | 0 | 0 | 0 | 0 |
| Oxytropis czapan-daghi | 0 | 0 | 1 | 0 | 0 | 0 | 0 | 0 | 0 |
| Oxytropis gracillima | 0 | 0 | 1 | 0 | 0 | 0 | 0 | 0 | 0 |
| Oxytropis guilanica | 0 | 0 | 1 | 0 | 0 | 0 | 0 | 0 | 0 |
| Oxytropis heratensis | 0 | 0 | 0 | 1 | 1 | 0 | 0 | 0 | 0 |
| Oxytropis hirsutiuscula | 0 | 0 | 1 | 1 | 1 | 0 | 0 | 0 | 0 |
| Oxytropis immersa | 0 | 1 | 1 | 0 | 0 | 1 | 0 | 0 | 0 |
| Oxytropis Iranica | 0 | 0 | 1 | 0 | 0 | 0 | 0 | 0 | 0 |
| Oxytropis javaherdehi | 0 | 0 | 1 | 0 | 0 | 0 | 0 | 0 | 0 |
| Oxytropis karjaginii | 0 | 1 | 1 | 1 | 0 | 1 | 0 | 1 | 0 |
| Oxytropis kermanica | 0 | 0 | 1 | 1 | 1 | 0 | 0 | 0 | 0 |
| Oxytropis lazica | 0 | 1 | 0 | 0 | 0 | 0 | 0 | 0 | 0 |
| Oxytropis mahneshanensis | 0 | 0 | 0 | 0 | 0 | 0 | 0 | 1 | 0 |
| Oxytropis masanderanensis | 0 | 0 | 1 | 1 | 0 | 0 | 0 | 0 | 0 |
| Oxytropis persica | 1 | 1 | 1 | 0 | 0 | 0 | 1 | 0 | 0 |
| Oxytropis pusilloides | 0 | 0 | 1 | 1 | 0 | 0 | 0 | 0 | 0 |
| Oxytropis savellanica | 0 | 1 | 0 | 0 | 0 | 1 | 1 | 0 | 0 |
| Oxytropis shahvarica | 0 | 0 | 1 | 0 | 0 | 0 | 0 | 0 | 0 |
| Oxytropis shirkuhi | 0 | 0 | 0 | 1 | 0 | 0 | 0 | 0 | 0 |
| Oxytropis sivehensis | 0 | 0 | 0 | 0 | 0 | 0 | 1 | 0 | 0 |
| Oxytropis sojakii | 0 | 0 | 0 | 0 | 1 | 0 | 0 | 0 | 0 |
| Oxytropis surmandehi | 0 | 0 | 0 | 1 | 0 | 0 | 0 | 0 | 0 |
| Oxytropis sutakensis | 0 | 0 | 1 | 0 | 0 | 0 | 0 | 0 | 0 |
| Oxytropis takhti-soleimanii | 0 | 0 | 1 | 0 | 0 | 0 | 0 | 0 | 0 |
| Oxytropis yazdi | 0 | 0 | 0 | 1 | 0 | 0 | 0 | 0 | 0 |
| Papaver armeniacum | 0 | 1 | 1 | 0 | 0 | 1 | 0 | 0 | 0 |
| Papaver bracteatum | 1 | 1 | 1 | 1 | 0 | 1 | 0 | 0 | 0 |
| Papaver fugax | 1 | 1 | 1 | 1 | 0 | 1 | 1 | 1 | 0 |
| Papaver gabrielianae | 0 | 1 | 0 | 0 | 0 | 0 | 0 | 0 | 0 |
| Papaver lateritium | 0 | 1 | 0 | 0 | 0 | 0 | 0 | 0 | 0 |
| Papaver orientale | 0 | 1 | 1 | 0 | 0 | 1 | 0 | 0 | 0 |
| Papaver polychaetum | 1 | 0 | 0 | 0 | 0 | 0 | 0 | 0 | 0 |
| Papaver pseudo-orientale | 0 | 1 | 1 | 0 | 0 | 1 | 0 | 1 | 0 |
| Papaver sjunicicum | 0 | 1 | 0 | 0 | 0 | 0 | 0 | 0 | 0 |
| Paracaryum lalezarense | 0 | 0 | 0 | 0 | 1 | 0 | 0 | 0 | 0 |
| Paracaryum polyanthum | 0 | 0 | 1 | 0 | 0 | 0 | 0 | 0 | 0 |
| Paracolpodium tzvelevii | 0 | 1 | 0 | 0 | 0 | 0 | 0 | 0 | 0 |
| Paraquilegia caespitosa | 0 | 0 | 1 | 0 | 0 | 0 | 0 | 0 | 0 |
| Parnassia cabulica | 0 | 0 | 0 | 0 | 1 | 0 | 0 | 0 | 0 |
| Parnassia palustris | 1 | 1 | 0 | 0 | 0 | 1 | 0 | 0 | 0 |
| Paronychia davisii | 1 | 0 | 0 | 0 | 0 | 0 | 0 | 0 | 0 |
| Paronychia saxatilis | 0 | 1 | 0 | 0 | 0 | 0 | 0 | 0 | 0 |
| Paronychia turcica | 0 | 1 | 0 | 0 | 0 | 0 | 0 | 0 | 0 |
| Pedicularis atropurpurea | 0 | 1 | 0 | 0 | 0 | 0 | 0 | 0 | 0 |
| Pedicularis cabulica | 0 | 0 | 0 | 0 | 1 | 0 | 0 | 0 | 0 |
| Pedicularis cadmea | 1 | 1 | 0 | 0 | 0 | 0 | 0 | 0 | 0 |
| Pedicularis caucasica | 0 | 1 | 1 | 0 | 0 | 1 | 1 | 0 | 0 |
| Pedicularis comosa | 1 | 1 | 0 | 0 | 0 | 0 | 0 | 0 | 1 |
| Pedicularis crassirostris | 0 | 1 | 0 | 0 | 0 | 0 | 0 | 0 | 0 |
| Pedicularis munzurdaghensis | 0 | 1 | 0 | 0 | 0 | 0 | 0 | 0 | 0 |
| Pedicularis nordmanniana | 0 | 1 | 0 | 0 | 0 | 0 | 0 | 0 | 0 |
| Pedicularis pontica | 0 | 1 | 0 | 0 | 0 | 0 | 0 | 0 | 0 |
| Pedicularis pycnantha | 0 | 0 | 1 | 1 | 0 | 0 | 1 | 0 | 0 |
| Pedicularis rhinanthoides | 0 | 0 | 1 | 0 | 0 | 0 | 0 | 0 | 0 |
| Pedicularis sibthorpii | 0 | 1 | 1 | 1 | 0 | 1 | 1 | 1 | 0 |
| Peltariopsis planisiliqua | 0 | 0 | 0 | 0 | 0 | 1 | 0 | 0 | 0 |
| Pentanema kurdistanicum | 0 | 0 | 0 | 1 | 0 | 0 | 0 | 0 | 0 |
| Petrorhagia sarbaghiae | 0 | 0 | 0 | 1 | 0 | 0 | 0 | 0 | 0 |
| Peucedanum alpinum | 1 | 0 | 0 | 0 | 0 | 0 | 0 | 0 | 0 |
| Peucedanum pimpinellifolia | 0 | 1 | 0 | 0 | 0 | 0 | 0 | 0 | 0 |
| Peucedanum ruthenicum | 0 | 0 | 0 | 1 | 0 | 0 | 0 | 0 | 0 |
| Peucedanum translucens | 0 | 0 | 1 | 0 | 0 | 0 | 0 | 0 | 0 |
| Peucedanum zozimioides | 1 | 0 | 0 | 0 | 0 | 0 | 0 | 0 | 0 |
| Phagnalon persicum | 0 | 0 | 0 | 1 | 1 | 0 | 0 | 0 | 0 |
| Phelipanche zangezuri | 0 | 1 | 0 | 0 | 0 | 0 | 0 | 0 | 0 |
| Phleum alpinum | 1 | 1 | 0 | 1 | 0 | 1 | 1 | 0 | 0 |
| Phleum iranicum | 0 | 0 | 1 | 0 | 0 | 0 | 0 | 0 | 0 |
| Phlomis anisodonta | 0 | 0 | 1 | 1 | 0 | 0 | 1 | 0 | 0 |
| Physoptychis gnaphalodes | 0 | 1 | 1 | 1 | 0 | 1 | 1 | 0 | 0 |
| Pilosella hoppeana | 0 | 1 | 0 | 0 | 0 | 0 | 0 | 0 | 0 |
| Pimpinella deverroides | 0 | 0 | 0 | 1 | 0 | 0 | 0 | 0 | 0 |
| Pimpinella saxifraga | 0 | 1 | 0 | 0 | 0 | 0 | 0 | 0 | 1 |
| Pimpinella tragium | 0 | 1 | 1 | 1 | 0 | 1 | 1 | 0 | 0 |
| Piptatherum denaense | 0 | 0 | 0 | 1 | 0 | 0 | 0 | 0 | 0 |
| Piptatherum laterale | 0 | 1 | 0 | 0 | 0 | 0 | 0 | 0 | 0 |
| Piptatherum molinioides | 0 | 0 | 0 | 1 | 1 | 0 | 0 | 0 | 0 |
| Plantago atrata | 1 | 1 | 1 | 1 | 0 | 1 | 1 | 0 | 0 |
| Plantago gentianoides | 0 | 0 | 1 | 0 | 1 | 0 | 0 | 0 | 0 |
| Poa aitchisonii | 0 | 0 | 1 | 0 | 0 | 0 | 0 | 0 | 0 |
| Poa alpina | 1 | 1 | 0 | 0 | 0 | 1 | 0 | 0 | 1 |
| Poa araratica | 0 | 1 | 1 | 1 | 0 | 1 | 1 | 0 | 0 |
| Poa bussmannii | 1 | 0 | 0 | 0 | 0 | 0 | 0 | 0 | 0 |
| Poa cenisia | 1 | 1 | 0 | 0 | 0 | 0 | 0 | 0 | 0 |
| Poa chaixii | 0 | 1 | 0 | 0 | 0 | 0 | 0 | 0 | 0 |
| Poa greuteri | 0 | 1 | 0 | 0 | 0 | 0 | 0 | 0 | 0 |
| Poa longifolia | 0 | 1 | 0 | 0 | 0 | 0 | 1 | 0 | 0 |
| Poa pseudobulbosa | 1 | 0 | 0 | 0 | 0 | 0 | 0 | 0 | 0 |
| Poa sterilis | 0 | 0 | 1 | 0 | 1 | 0 | 0 | 0 | 0 |
| Poa supina | 1 | 1 | 0 | 0 | 0 | 0 | 0 | 0 | 0 |
| Polygonum bistorta | 0 | 1 | 1 | 1 | 0 | 1 | 0 | 1 | 0 |
| Polygonum dumosum | 0 | 0 | 0 | 1 | 1 | 0 | 0 | 0 | 0 |
| Polygonum luzuloides | 0 | 1 | 0 | 1 | 0 | 1 | 1 | 0 | 0 |
| Polygonum molliaeforme | 0 | 0 | 1 | 1 | 1 | 0 | 0 | 0 | 0 |
| Polygonum serpyllaceum | 0 | 0 | 1 | 1 | 0 | 0 | 0 | 0 | 0 |
| Polygonum spinosum | 0 | 0 | 0 | 0 | 1 | 0 | 0 | 0 | 0 |
| Polygonum thymifolium | 0 | 0 | 1 | 1 | 1 | 1 | 0 | 0 | 0 |
| Polylophium involucratum | 0 | 0 | 1 | 0 | 0 | 0 | 0 | 0 | 0 |
| Potamogeton gramineus | 0 | 0 | 0 | 0 | 0 | 1 | 0 | 0 | 0 |
| Potentilla agrimonioides | 0 | 1 | 0 | 0 | 0 | 1 | 0 | 0 | 0 |
| Potentilla aladaghensis | 1 | 0 | 0 | 0 | 0 | 0 | 0 | 0 | 0 |
| Potentilla anatolica | 0 | 1 | 0 | 0 | 0 | 1 | 0 | 0 | 0 |
| Potentilla argaea | 1 | 1 | 1 | 0 | 0 | 1 | 0 | 0 | 0 |
| Potentilla argyroloma | 0 | 0 | 1 | 1 | 0 | 0 | 0 | 0 | 0 |
| Potentilla aucheriana | 0 | 1 | 1 | 0 | 0 | 1 | 0 | 0 | 0 |
| Potentilla bifurca | 0 | 0 | 0 | 1 | 0 | 1 | 0 | 0 | 0 |
| Potentilla cappadocica | 0 | 1 | 0 | 0 | 0 | 0 | 0 | 0 | 0 |
| Potentilla carduchorum | 0 | 1 | 0 | 0 | 0 | 0 | 0 | 0 | 0 |
| Potentilla crantzii | 0 | 1 | 1 | 0 | 0 | 0 | 0 | 0 | 0 |
| Potentilla cryptophila | 0 | 1 | 1 | 0 | 0 | 1 | 0 | 0 | 0 |
| Potentilla diversidentata | 0 | 0 | 1 | 0 | 0 | 0 | 0 | 0 | 0 |
| Potentilla doddsii | 0 | 1 | 0 | 0 | 0 | 0 | 0 | 0 | 0 |
| Potentilla elvendensis | 0 | 0 | 0 | 1 | 0 | 0 | 0 | 0 | 0 |
| Potentilla flaccida | 0 | 0 | 1 | 1 | 0 | 0 | 0 | 0 | 0 |
| Potentilla fruticosa | 0 | 1 | 0 | 0 | 0 | 0 | 0 | 0 | 0 |
| Potentilla geranioides | 0 | 1 | 0 | 0 | 0 | 0 | 0 | 0 | 0 |
| Potentilla hololeuca | 0 | 1 | 1 | 0 | 0 | 0 | 0 | 0 | 0 |
| Potentilla humifusa | 0 | 1 | 0 | 0 | 0 | 0 | 0 | 0 | 0 |
| Potentilla lazica | 0 | 1 | 0 | 0 | 0 | 0 | 0 | 0 | 0 |
| Potentilla lignosa | 0 | 0 | 1 | 1 | 0 | 0 | 0 | 0 | 0 |
| Potentilla mallota | 0 | 0 | 1 | 1 | 0 | 0 | 0 | 0 | 0 |
| Potentilla meyeri | 1 | 1 | 1 | 0 | 0 | 1 | 0 | 0 | 0 |
| Potentilla multifida | 0 | 0 | 1 | 0 | 0 | 0 | 0 | 0 | 0 |
| Potentilla nuda | 0 | 0 | 1 | 1 | 1 | 1 | 0 | 0 | 0 |
| Potentilla nurensis | 0 | 0 | 0 | 1 | 0 | 1 | 0 | 0 | 0 |
| Potentilla oweriniana | 0 | 1 | 0 | 0 | 0 | 0 | 0 | 0 | 0 |
| Potentilla palustris | 0 | 1 | 0 | 0 | 0 | 0 | 0 | 0 | 0 |
| Potentilla pannosa | 0 | 1 | 1 | 1 | 0 | 0 | 0 | 0 | 0 |
| Potentilla polyschista | 0 | 1 | 1 | 0 | 0 | 0 | 0 | 0 | 0 |
| Potentilla porphyrantha | 0 | 1 | 1 | 0 | 0 | 1 | 0 | 0 | 0 |
| Potentilla poteriifolia | 0 | 0 | 1 | 1 | 0 | 0 | 0 | 0 | 0 |
| Potentilla pulvinaris | 1 | 0 | 0 | 0 | 0 | 0 | 0 | 0 | 0 |
| Potentilla rupestris | 0 | 1 | 0 | 0 | 0 | 0 | 0 | 0 | 0 |
| Potentilla ruprechtii | 0 | 1 | 0 | 0 | 0 | 0 | 0 | 0 | 0 |
| Potentilla sangedehensis | 0 | 0 | 1 | 0 | 0 | 0 | 0 | 0 | 0 |
| Potentilla sawalensis | 0 | 1 | 0 | 0 | 0 | 0 | 0 | 0 | 0 |
| Potentilla seidlitziana | 0 | 1 | 0 | 0 | 0 | 0 | 0 | 0 | 0 |
| Potentilla speciosa | 1 | 1 | 0 | 0 | 0 | 0 | 0 | 0 | 0 |
| Potentilla subpalmata | 0 | 1 | 0 | 0 | 0 | 0 | 0 | 0 | 0 |
| Potentilla szovitsii | 0 | 0 | 1 | 0 | 0 | 0 | 0 | 0 | 0 |
| Potentilla thuringiaca | 0 | 1 | 0 | 0 | 0 | 0 | 0 | 0 | 0 |
| Prangos ferulacea | 1 | 1 | 1 | 1 | 0 | 1 | 1 | 0 | 0 |
| Prangos platychlaena | 0 | 0 | 0 | 0 | 0 | 0 | 0 | 0 | 1 |
| Prangos tuberculata | 0 | 0 | 0 | 1 | 0 | 0 | 0 | 0 | 0 |
| Prangos uloptera | 0 | 1 | 1 | 1 | 0 | 1 | 1 | 0 | 0 |
| Prenanthes glareosa | 1 | 0 | 0 | 0 | 0 | 0 | 0 | 0 | 0 |
| Primula algida | 0 | 1 | 0 | 0 | 0 | 0 | 0 | 0 | 0 |
| Primula auriculata | 1 | 1 | 1 | 1 | 0 | 1 | 1 | 0 | 1 |
| Primula capitellata | 0 | 0 | 0 | 1 | 1 | 0 | 0 | 0 | 0 |
| Primula elatior | 0 | 1 | 0 | 0 | 0 | 0 | 0 | 0 | 0 |
| Primula longipes | 0 | 1 | 0 | 0 | 0 | 0 | 0 | 0 | 0 |
| Psathyrostachys fragilis | 0 | 1 | 0 | 0 | 0 | 1 | 0 | 0 | 0 |
| Psephellus khalkhalensis | 0 | 0 | 1 | 0 | 0 | 0 | 0 | 0 | 0 |
| Psephellus transcaucasicus | 0 | 1 | 0 | 0 | 0 | 0 | 0 | 0 | 0 |
| Pseudocamelina aphragmodes | 0 | 0 | 0 | 1 | 0 | 0 | 0 | 0 | 0 |
| Pseudocamelina glaucophylla | 0 | 0 | 1 | 1 | 0 | 1 | 0 | 0 | 0 |
| Pseudocamelina kermanica | 0 | 0 | 0 | 0 | 1 | 0 | 0 | 0 | 0 |
| Psychrogeton aellenii | 0 | 0 | 1 | 0 | 0 | 0 | 0 | 0 | 0 |
| Psychrogeton alexeenkoi | 0 | 0 | 0 | 1 | 1 | 0 | 0 | 0 | 0 |
| Psychrogeton amorphoglossus | 0 | 1 | 1 | 1 | 1 | 0 | 0 | 0 | 0 |
| Psychrogeton aucheri | 0 | 0 | 1 | 1 | 0 | 0 | 0 | 0 | 0 |
| Psychrogeton chionophilus | 0 | 0 | 0 | 1 | 1 | 0 | 0 | 0 | 0 |
| Psychrogeton persicus | 0 | 0 | 1 | 1 | 1 | 0 | 0 | 0 | 0 |
| Pulsatilla albana | 0 | 1 | 1 | 0 | 0 | 1 | 0 | 0 | 0 |
| Puschkinia bilgineri | 0 | 1 | 0 | 0 | 0 | 0 | 0 | 0 | 0 |
| Puschkinia kurdica | 0 | 1 | 0 | 0 | 0 | 0 | 0 | 0 | 0 |
| Puschkinia scilloides | 0 | 1 | 1 | 1 | 0 | 1 | 1 | 0 | 0 |
| Ranunculus anatolicus | 0 | 1 | 0 | 0 | 0 | 0 | 0 | 0 | 0 |
| Ranunculus aragazi | 0 | 1 | 0 | 0 | 0 | 0 | 0 | 0 | 0 |
| Ranunculus aucheri | 0 | 1 | 1 | 1 | 1 | 0 | 0 | 0 | 0 |
| Ranunculus bingoeldaghensis | 0 | 1 | 0 | 0 | 0 | 0 | 0 | 0 | 0 |
| Ranunculus brachylobus | 1 | 1 | 1 | 1 | 0 | 1 | 0 | 0 | 0 |
| Ranunculus bulbilliferus | 0 | 0 | 1 | 0 | 0 | 0 | 1 | 0 | 0 |
| Ranunculus caucasicus | 0 | 1 | 0 | 0 | 0 | 0 | 0 | 0 | 0 |
| Ranunculus crateris | 0 | 1 | 0 | 0 | 0 | 0 | 0 | 0 | 0 |
| Ranunculus crymophilus | 0 | 1 | 1 | 1 | 0 | 1 | 0 | 0 | 0 |
| Ranunculus dalechanensis | 0 | 0 | 0 | 1 | 0 | 0 | 0 | 0 | 0 |
| Ranunculus demissus | 1 | 0 | 0 | 0 | 0 | 0 | 0 | 0 | 0 |
| Ranunculus dissectus | 0 | 1 | 0 | 0 | 0 | 0 | 0 | 0 | 1 |
| Ranunculus divaricatus | 0 | 1 | 0 | 0 | 0 | 0 | 0 | 0 | 0 |
| Ranunculus diversifolius | 0 | 1 | 0 | 1 | 0 | 0 | 1 | 0 | 0 |
| Ranunculus elymaiticus | 0 | 0 | 0 | 1 | 0 | 0 | 0 | 0 | 0 |
| Ranunculus eriorrhizus | 0 | 0 | 0 | 1 | 1 | 0 | 0 | 0 | 0 |
| Ranunculus fenzlii | 1 | 1 | 0 | 0 | 0 | 0 | 0 | 0 | 0 |
| Ranunculus grandiflorus | 1 | 1 | 0 | 0 | 0 | 1 | 0 | 0 | 0 |
| Ranunculus microflorus | 0 | 0 | 0 | 1 | 0 | 0 | 0 | 0 | 0 |
| Ranunculus obesus | 0 | 1 | 0 | 0 | 0 | 0 | 0 | 0 | 0 |
| Ranunculus oreophilus | 0 | 1 | 0 | 0 | 0 | 1 | 0 | 0 | 1 |
| Ranunculus papyrocarpus | 0 | 0 | 0 | 1 | 1 | 0 | 0 | 0 | 0 |
| Ranunculus pichleri | 0 | 0 | 0 | 1 | 0 | 0 | 0 | 0 | 0 |
| Ranunculus polyrhizos | 0 | 1 | 0 | 0 | 0 | 0 | 0 | 0 | 0 |
| Ranunculus renzii | 0 | 1 | 0 | 0 | 0 | 0 | 0 | 0 | 0 |
| Ranunculus sojakii | 0 | 0 | 1 | 0 | 0 | 0 | 0 | 0 | 0 |
| Ranunculus straussii | 0 | 0 | 0 | 1 | 0 | 0 | 0 | 0 | 0 |
| Ranunculus tempskyanus | 0 | 1 | 0 | 0 | 0 | 0 | 0 | 0 | 0 |
| Ranunculus termei | 0 | 0 | 0 | 1 | 0 | 0 | 0 | 0 | 0 |
| Ranunculus transcaucasicus | 0 | 1 | 0 | 0 | 0 | 1 | 0 | 0 | 0 |
| Ranunculus trichocarpus | 0 | 1 | 1 | 0 | 0 | 1 | 1 | 0 | 0 |
| Ranunculus vanensis | 0 | 1 | 0 | 0 | 0 | 0 | 0 | 0 | 0 |
| Ranunculus vermirrhizus | 0 | 1 | 0 | 0 | 0 | 0 | 0 | 0 | 0 |
| Ranunculus zenjanensis | 0 | 0 | 0 | 1 | 0 | 0 | 0 | 1 | 0 |
| Rhabdosciadium anatolyi | 0 | 1 | 0 | 0 | 0 | 0 | 0 | 0 | 0 |
| Rhabdosciadium aucheri | 0 | 0 | 0 | 1 | 0 | 0 | 1 | 0 | 0 |
| Rhabdosciadium petiolare | 0 | 0 | 0 | 1 | 0 | 0 | 0 | 0 | 0 |
| Rhamnus cornifolia | 1 | 1 | 0 | 1 | 0 | 1 | 1 | 0 | 0 |
| Rhamnus prostrata | 0 | 0 | 0 | 0 | 1 | 0 | 0 | 0 | 0 |
| Rhynchocorys kurdica | 0 | 1 | 0 | 0 | 0 | 0 | 0 | 0 | 0 |
| Ribes anatolicum | 0 | 1 | 0 | 0 | 0 | 0 | 0 | 0 | 0 |
| Ribes orientale | 0 | 0 | 1 | 0 | 1 | 1 | 0 | 0 | 0 |
| Ricotia aucheri | 0 | 1 | 0 | 0 | 0 | 0 | 0 | 0 | 0 |
| Ricotia varians | 1 | 0 | 0 | 0 | 0 | 0 | 0 | 0 | 0 |
| Rindera albida | 0 | 1 | 0 | 1 | 0 | 0 | 0 | 0 | 0 |
| Rindera caespitosa | 1 | 1 | 0 | 0 | 0 | 0 | 0 | 0 | 0 |
| Rosa beggeriana | 0 | 0 | 1 | 1 | 1 | 0 | 0 | 0 | 0 |
| Rosularia aizoon | 1 | 1 | 0 | 0 | 0 | 0 | 0 | 0 | 0 |
| Rosularia chrysantha | 1 | 0 | 0 | 0 | 0 | 0 | 0 | 0 | 0 |
| Rosularia davisii | 0 | 1 | 0 | 0 | 0 | 0 | 0 | 0 | 0 |
| Rosularia elymaitica | 0 | 1 | 0 | 1 | 0 | 0 | 0 | 0 | 0 |
| Rosularia persica | 0 | 0 | 1 | 1 | 0 | 1 | 1 | 0 | 0 |
| Rosularia rechingeri | 0 | 1 | 0 | 0 | 0 | 0 | 0 | 0 | 0 |
| Rosularia sempervivum | 1 | 1 | 0 | 0 | 0 | 0 | 0 | 0 | 0 |
| Rubia caramanica | 0 | 0 | 0 | 0 | 1 | 0 | 0 | 0 | 0 |
| Rubia pauciflora | 0 | 0 | 0 | 1 | 0 | 0 | 0 | 0 | 0 |
| Rumex acetoselloides | 0 | 1 | 0 | 0 | 0 | 0 | 0 | 0 | 0 |
| Rumex alpinus | 0 | 1 | 0 | 0 | 0 | 0 | 0 | 0 | 0 |
| Rumex angustifolius | 1 | 1 | 0 | 1 | 0 | 0 | 1 | 0 | 0 |
| Rumex caucasicus | 0 | 1 | 0 | 0 | 0 | 0 | 0 | 0 | 0 |
| Rumex elbursensis | 0 | 0 | 1 | 1 | 0 | 1 | 0 | 0 | 0 |
| Rumex gracilescens | 0 | 1 | 0 | 0 | 0 | 0 | 0 | 0 | 0 |
| Rumex patientia | 0 | 0 | 1 | 1 | 0 | 1 | 0 | 0 | 0 |
| Rumex ponticus | 1 | 1 | 0 | 0 | 0 | 0 | 0 | 0 | 0 |
| Sagina saginoides | 0 | 1 | 1 | 1 | 1 | 1 | 0 | 0 | 0 |
| Salsola canescens | 0 | 1 | 1 | 1 | 0 | 0 | 1 | 0 | 0 |
| Salvia lachnocalyx | 0 | 0 | 0 | 1 | 0 | 0 | 0 | 0 | 0 |
| Salvia pachystachya | 0 | 1 | 0 | 0 | 0 | 0 | 0 | 0 | 0 |
| Salvia rhytidea | 0 | 0 | 0 | 0 | 1 | 0 | 0 | 0 | 0 |
| Salvia sahendica | 0 | 0 | 0 | 0 | 0 | 1 | 0 | 0 | 0 |
| Salvia staminea | 0 | 1 | 1 | 0 | 0 | 1 | 0 | 0 | 0 |
| Saponaria iranica | 0 | 0 | 0 | 0 | 1 | 0 | 0 | 0 | 0 |
| Saponaria pumilio | 1 | 0 | 0 | 0 | 0 | 0 | 0 | 0 | 0 |
| Satureja kallarica | 0 | 0 | 0 | 1 | 0 | 0 | 0 | 0 | 0 |
| Saussurea salsa | 0 | 1 | 0 | 0 | 0 | 1 | 0 | 0 | 0 |
| Saxifraga exarata | 1 | 1 | 0 | 0 | 0 | 0 | 0 | 0 | 0 |
| Saxifraga hirculus | 0 | 1 | 0 | 0 | 0 | 0 | 0 | 0 | 0 |
| Saxifraga iranica | 0 | 0 | 1 | 0 | 0 | 0 | 0 | 0 | 0 |
| Saxifraga juniperifolia | 0 | 1 | 0 | 0 | 0 | 0 | 0 | 0 | 0 |
| Saxifraga koelzii | 0 | 0 | 1 | 0 | 0 | 0 | 0 | 0 | 0 |
| Saxifraga kolenatiana | 0 | 1 | 0 | 0 | 0 | 0 | 0 | 0 | 0 |
| Saxifraga ramsarica | 0 | 0 | 1 | 0 | 0 | 0 | 0 | 0 | 0 |
| Saxifraga sibirica | 0 | 1 | 0 | 0 | 0 | 1 | 0 | 0 | 1 |
| Saxifraga wendelboi | 0 | 0 | 1 | 0 | 0 | 0 | 0 | 0 | 0 |
| Scabiosa caucasica | 0 | 1 | 1 | 0 | 0 | 1 | 0 | 0 | 0 |
| Scilla alinihatiana | 0 | 1 | 0 | 0 | 0 | 0 | 0 | 0 | 0 |
| Scleranthus uncinatus | 1 | 1 | 0 | 0 | 0 | 0 | 0 | 0 | 0 |
| Sclerochorton haussknechtii | 0 | 0 | 0 | 1 | 0 | 0 | 0 | 0 | 0 |
| Scorzonera cana | 1 | 1 | 0 | 0 | 0 | 0 | 0 | 0 | 0 |
| Scorzonera grossheimii | 0 | 0 | 1 | 1 | 0 | 1 | 0 | 0 | 0 |
| Scorzonera intricata | 0 | 0 | 0 | 1 | 1 | 0 | 0 | 0 | 0 |
| Scorzonera karkasensis | 0 | 0 | 0 | 1 | 0 | 0 | 0 | 0 | 0 |
| Scorzonera kirpicznikovii | 0 | 0 | 1 | 0 | 0 | 0 | 0 | 0 | 0 |
| Scorzonera meyeri | 0 | 1 | 1 | 0 | 0 | 1 | 0 | 0 | 0 |
| Scorzonera nivalis | 0 | 0 | 0 | 1 | 0 | 0 | 0 | 0 | 0 |
| Scorzonera psychrophila | 0 | 0 | 0 | 1 | 0 | 0 | 0 | 0 | 0 |
| Scorzonera pygmaea | 1 | 0 | 0 | 0 | 0 | 0 | 0 | 0 | 1 |
| Scorzonera raddeana | 0 | 0 | 1 | 0 | 1 | 0 | 0 | 0 | 0 |
| Scorzonera radicosa | 0 | 0 | 1 | 0 | 0 | 1 | 1 | 0 | 0 |
| Scorzonera rigida | 1 | 1 | 1 | 0 | 0 | 1 | 0 | 0 | 0 |
| Scorzonera seidlitzii | 0 | 0 | 1 | 0 | 0 | 0 | 0 | 0 | 0 |
| Scorzonera sericea | 1 | 1 | 0 | 0 | 0 | 0 | 0 | 0 | 0 |
| Scorzonera stenocephala | 0 | 0 | 1 | 1 | 0 | 0 | 0 | 0 | 0 |
| Scorzonera subaphylla | 0 | 0 | 0 | 1 | 0 | 0 | 0 | 0 | 0 |
| Scorzonera xylobasis | 0 | 0 | 1 | 0 | 0 | 0 | 0 | 0 | 0 |
| Scrophularia amplexicaulis | 0 | 1 | 1 | 1 | 0 | 1 | 0 | 0 | 0 |
| Scrophularia atroglandulosa | 0 | 0 | 0 | 0 | 0 | 0 | 1 | 0 | 0 |
| Scrophularia catariifolia | 0 | 1 | 0 | 0 | 0 | 0 | 1 | 0 | 0 |
| Scrophularia chlorantha | 0 | 1 | 0 | 0 | 0 | 0 | 0 | 0 | 0 |
| Scrophularia chrysantha | 0 | 1 | 0 | 0 | 0 | 0 | 0 | 0 | 0 |
| Scrophularia crassicaulis | 0 | 0 | 1 | 0 | 0 | 0 | 0 | 0 | 0 |
| Scrophularia crassiuscula | 0 | 0 | 0 | 1 | 0 | 0 | 0 | 0 | 0 |
| Scrophularia fatmae | 0 | 1 | 0 | 0 | 0 | 0 | 0 | 0 | 0 |
| Scrophularia flava | 0 | 0 | 0 | 1 | 0 | 0 | 0 | 0 | 0 |
| Scrophularia frigida | 0 | 1 | 1 | 1 | 1 | 0 | 0 | 0 | 0 |
| Scrophularia gorganica | 0 | 0 | 1 | 0 | 0 | 0 | 0 | 0 | 0 |
| Scrophularia kurdica | 0 | 1 | 0 | 0 | 0 | 0 | 0 | 0 | 0 |
| Scrophularia libanotica | 1 | 0 | 0 | 0 | 0 | 0 | 0 | 0 | 0 |
| Scrophularia olympica | 1 | 1 | 0 | 0 | 0 | 1 | 0 | 0 | 1 |
| Scrophularia pumilio | 0 | 1 | 0 | 0 | 0 | 0 | 0 | 0 | 0 |
| Scrophularia subaequiloba | 0 | 1 | 0 | 0 | 0 | 0 | 0 | 0 | 0 |
| Scrophularia subaphylla | 0 | 0 | 1 | 1 | 1 | 1 | 0 | 0 | 0 |
| Scutellaria araxensis | 0 | 0 | 0 | 1 | 0 | 0 | 0 | 0 | 0 |
| Scutellaria glechomoides | 0 | 0 | 1 | 0 | 0 | 0 | 0 | 0 | 0 |
| Scutellaria heterophylla | 1 | 1 | 0 | 0 | 0 | 0 | 0 | 0 | 0 |
| Scutellaria multicaulis | 0 | 0 | 0 | 1 | 1 | 0 | 0 | 0 | 0 |
| Scutellaria patonii | 0 | 0 | 0 | 1 | 0 | 0 | 0 | 0 | 0 |
| Scutellaria pinnatifida | 0 | 1 | 1 | 1 | 0 | 1 | 1 | 0 | 0 |
| Scutellaria pontica | 0 | 1 | 0 | 0 | 0 | 0 | 0 | 0 | 0 |
| Sedum adscendens | 1 | 1 | 0 | 0 | 0 | 0 | 0 | 0 | 0 |
| Sedum alpestre | 0 | 1 | 0 | 0 | 0 | 0 | 0 | 0 | 0 |
| Sedum annuum | 1 | 1 | 0 | 0 | 0 | 0 | 0 | 0 | 0 |
| Sedum artvinensis | 0 | 1 | 0 | 0 | 0 | 0 | 0 | 0 | 0 |
| Sedum euxinum | 1 | 1 | 0 | 0 | 0 | 0 | 0 | 0 | 0 |
| Sedum exarata | 1 | 1 | 0 | 0 | 0 | 0 | 0 | 0 | 1 |
| Sedum gracile | 0 | 1 | 0 | 0 | 0 | 0 | 0 | 0 | 0 |
| Sedum hewittii | 0 | 1 | 0 | 0 | 0 | 0 | 0 | 0 | 0 |
| Sedum juniperifolia | 0 | 1 | 0 | 0 | 0 | 0 | 0 | 0 | 0 |
| Sedum kotschyanum | 1 | 1 | 0 | 1 | 0 | 0 | 0 | 0 | 0 |
| Sedum luteoviride | 0 | 1 | 0 | 0 | 0 | 0 | 0 | 0 | 1 |
| Sedum moschata | 0 | 1 | 0 | 0 | 0 | 0 | 0 | 0 | 0 |
| Sedum nanum | 0 | 1 | 0 | 0 | 0 | 0 | 0 | 0 | 0 |
| Sedum oppositifolium | 0 | 1 | 0 | 0 | 0 | 0 | 0 | 0 | 0 |
| Sedum paniculatum | 0 | 1 | 0 | 0 | 0 | 0 | 0 | 0 | 1 |
| Sedum sempervivum | 0 | 0 | 0 | 0 | 0 | 0 | 0 | 0 | 1 |
| Sedum sibiricum | 0 | 1 | 0 | 0 | 0 | 0 | 0 | 0 | 1 |
| Sedum subulatum | 0 | 1 | 0 | 0 | 0 | 0 | 0 | 0 | 0 |
| Sedum tenellum | 1 | 1 | 1 | 0 | 0 | 0 | 0 | 0 | 0 |
| Sedum transcaucasicum | 0 | 1 | 0 | 0 | 0 | 0 | 0 | 0 | 0 |
| Semenovia dichotoma | 0 | 0 | 0 | 1 | 0 | 0 | 0 | 0 | 0 |
| Semenovia frigida | 0 | 0 | 0 | 1 | 0 | 0 | 0 | 0 | 0 |
| Semenovia subscaposa | 0 | 0 | 1 | 0 | 0 | 0 | 0 | 0 | 0 |
| Semenovia suffruticosa | 0 | 0 | 0 | 0 | 1 | 0 | 0 | 0 | 0 |
| Semenovia tragioides | 0 | 0 | 1 | 1 | 0 | 1 | 0 | 0 | 0 |
| Sempervivum atropatanum | 0 | 0 | 1 | 0 | 0 | 1 | 0 | 0 | 0 |
| Sempervivum globiferum | 0 | 1 | 0 | 0 | 0 | 0 | 0 | 0 | 0 |
| Sempervivum iranicum | 0 | 0 | 1 | 0 | 0 | 0 | 0 | 0 | 0 |
| Sempervivum pisidicum | 1 | 0 | 0 | 0 | 0 | 0 | 0 | 0 | 0 |
| Sempervivum transcaucasicum | 0 | 1 | 0 | 0 | 0 | 0 | 0 | 0 | 0 |
| Sempervivum tunaekimii | 0 | 1 | 0 | 0 | 0 | 0 | 0 | 0 | 0 |
| Senecio cilicius | 0 | 1 | 0 | 0 | 0 | 0 | 0 | 0 | 0 |
| Senecio davisii | 0 | 1 | 0 | 0 | 0 | 0 | 1 | 0 | 0 |
| Senecio eligulatus | 0 | 0 | 0 | 0 | 1 | 0 | 0 | 0 | 0 |
| Senecio eriospermus | 0 | 1 | 0 | 0 | 0 | 0 | 0 | 0 | 0 |
| Senecio hypochionaeus | 1 | 1 | 0 | 0 | 0 | 0 | 0 | 0 | 1 |
| Senecio integrifolius | 0 | 1 | 0 | 0 | 0 | 0 | 0 | 0 | 0 |
| Senecio iranicus | 0 | 0 | 1 | 0 | 0 | 0 | 0 | 0 | 0 |
| Senecio jurineifolius | 1 | 0 | 0 | 0 | 0 | 0 | 0 | 0 | 0 |
| Senecio kotschyanus | 0 | 0 | 0 | 1 | 0 | 0 | 0 | 0 | 0 |
| Senecio munzurdaglarensis | 0 | 1 | 0 | 0 | 0 | 0 | 0 | 0 | 0 |
| Senecio subnivalis | 0 | 0 | 0 | 0 | 1 | 0 | 0 | 0 | 0 |
| Senecio taraxacifolius | 0 | 1 | 0 | 0 | 0 | 1 | 0 | 0 | 0 |
| Senecio vulcanicus | 0 | 0 | 1 | 0 | 0 | 0 | 0 | 0 | 0 |
| Serratula hakkiarica | 0 | 1 | 0 | 0 | 0 | 0 | 0 | 0 | 0 |
| Serratula haussknechtii | 0 | 1 | 1 | 1 | 0 | 1 | 0 | 0 | 0 |
| Serratula melanocheila | 0 | 0 | 0 | 1 | 0 | 0 | 0 | 0 | 0 |
| Seseli grandivittatum | 1 | 0 | 0 | 0 | 0 | 0 | 0 | 0 | 0 |
| Sesleria araratica | 0 | 1 | 0 | 0 | 0 | 0 | 0 | 0 | 0 |
| Sesleria phleoides | 1 | 1 | 1 | 0 | 0 | 1 | 0 | 0 | 0 |
| Sibbaldia parviflora | 1 | 1 | 1 | 0 | 0 | 1 | 0 | 0 | 1 |
| Sideritis phlomoides | 1 | 0 | 0 | 0 | 0 | 0 | 0 | 0 | 0 |
| Silene araratica | 0 | 1 | 0 | 0 | 0 | 1 | 0 | 0 | 0 |
| Silene argaea | 1 | 0 | 0 | 0 | 0 | 0 | 0 | 0 | 0 |
| Silene azirensis | 0 | 1 | 0 | 0 | 0 | 0 | 0 | 0 | 0 |
| Silene balansae | 1 | 0 | 0 | 0 | 0 | 0 | 0 | 0 | 0 |
| Silene caroli-henrici | 0 | 0 | 0 | 1 | 0 | 0 | 0 | 0 | 0 |
| Silene cartilaginea | 0 | 1 | 0 | 0 | 0 | 0 | 0 | 0 | 0 |
| Silene caryophylloides | 1 | 1 | 0 | 0 | 0 | 0 | 0 | 0 | 0 |
| Silene caucasica | 0 | 1 | 0 | 0 | 0 | 0 | 0 | 0 | 0 |
| Silene cephalantha | 0 | 1 | 0 | 1 | 0 | 1 | 0 | 0 | 0 |
| Silene chustupica | 0 | 1 | 0 | 0 | 0 | 0 | 0 | 0 | 0 |
| Silene daenensis | 0 | 0 | 0 | 1 | 1 | 0 | 0 | 0 | 0 |
| Silene demawendica | 0 | 0 | 1 | 0 | 0 | 0 | 0 | 0 | 0 |
| Silene dianthoides | 1 | 1 | 0 | 0 | 0 | 0 | 0 | 0 | 0 |
| Silene dschuparensis | 0 | 0 | 0 | 0 | 1 | 0 | 0 | 0 | 0 |
| Silene erciyesdaghensis | 1 | 0 | 0 | 0 | 0 | 0 | 0 | 0 | 0 |
| Silene eremicana | 0 | 1 | 0 | 1 | 0 | 0 | 0 | 0 | 0 |
| Silene ghahremaninejadii | 0 | 0 | 0 | 1 | 0 | 0 | 0 | 0 | 0 |
| Silene goniocaula | 0 | 0 | 0 | 1 | 0 | 1 | 1 | 0 | 0 |
| Silene guntensis | 0 | 0 | 1 | 1 | 0 | 0 | 0 | 0 | 0 |
| Silene gynodioica | 0 | 0 | 1 | 1 | 0 | 0 | 0 | 0 | 0 |
| Silene hirticalyx | 0 | 0 | 0 | 1 | 0 | 0 | 0 | 0 | 0 |
| Silene konuralpii | 0 | 1 | 0 | 0 | 0 | 0 | 0 | 0 | 0 |
| Silene lasiantha | 0 | 1 | 0 | 0 | 0 | 0 | 0 | 0 | 0 |
| Silene laxa | 1 | 1 | 1 | 1 | 0 | 0 | 0 | 0 | 0 |
| Silene lucida | 0 | 1 | 0 | 0 | 0 | 0 | 0 | 0 | 0 |
| Silene marschallii | 0 | 0 | 1 | 0 | 0 | 1 | 1 | 0 | 0 |
| Silene meyeri | 0 | 1 | 1 | 1 | 0 | 1 | 0 | 1 | 0 |
| Silene miksensis | 0 | 1 | 0 | 0 | 0 | 0 | 0 | 0 | 0 |
| Silene nuncupanda | 1 | 0 | 0 | 0 | 0 | 0 | 0 | 0 | 0 |
| Silene nurensis | 0 | 0 | 0 | 1 | 1 | 0 | 0 | 1 | 0 |
| Silene odontopetala | 1 | 1 | 1 | 0 | 0 | 1 | 1 | 0 | 0 |
| Silene oreades | 1 | 0 | 0 | 0 | 0 | 0 | 0 | 0 | 1 |
| Silene orientoalborzensis | 0 | 0 | 1 | 0 | 0 | 0 | 0 | 0 | 0 |
| Silene oxelmanii | 0 | 0 | 0 | 1 | 0 | 0 | 0 | 0 | 0 |
| Silene persica | 0 | 0 | 0 | 1 | 0 | 0 | 0 | 0 | 0 |
| Silene pseudonurensis | 0 | 0 | 0 | 1 | 0 | 0 | 0 | 0 | 0 |
| Silene pungens | 0 | 1 | 0 | 0 | 0 | 1 | 1 | 0 | 1 |
| Silene rhynchocarpa | 1 | 1 | 0 | 1 | 0 | 0 | 0 | 0 | 1 |
| Silene ruprechtii | 0 | 1 | 0 | 0 | 0 | 1 | 0 | 0 | 0 |
| Silene saxatilis | 0 | 1 | 0 | 0 | 0 | 1 | 0 | 0 | 0 |
| Silene tachtensis | 0 | 0 | 1 | 0 | 0 | 0 | 0 | 0 | 0 |
| Silene tragacantha | 0 | 0 | 0 | 1 | 0 | 0 | 0 | 0 | 0 |
| Silene viscosa | 0 | 1 | 1 | 1 | 1 | 1 | 0 | 0 | 0 |
| Silene yildirimlii | 0 | 1 | 0 | 0 | 0 | 0 | 0 | 0 | 0 |
| Solenanthus circinatus | 0 | 1 | 1 | 1 | 1 | 1 | 1 | 0 | 0 |
| Solenanthus stamineus | 1 | 1 | 1 | 1 | 0 | 1 | 0 | 0 | 0 |
| Solidago virgaurea | 1 | 1 | 0 | 0 | 0 | 0 | 0 | 0 | 0 |
| Sorbus tamamschjanae | 0 | 1 | 0 | 0 | 0 | 0 | 0 | 0 | 0 |
| Stachys acerosa | 0 | 0 | 0 | 1 | 1 | 0 | 0 | 0 | 0 |
| Stachys balansae | 0 | 1 | 0 | 0 | 0 | 0 | 0 | 0 | 0 |
| Stachys choruhensis | 0 | 1 | 0 | 0 | 0 | 0 | 0 | 0 | 0 |
| Stachys citrina | 1 | 0 | 0 | 0 | 0 | 0 | 0 | 0 | 1 |
| Stachys lanigera | 0 | 0 | 0 | 1 | 0 | 0 | 1 | 0 | 0 |
| Stachys macrantha | 0 | 1 | 0 | 0 | 0 | 0 | 0 | 0 | 0 |
| Stachys obtusicrena | 0 | 0 | 0 | 1 | 0 | 0 | 0 | 0 | 0 |
| Stachys pilifera | 0 | 0 | 0 | 1 | 0 | 0 | 0 | 0 | 0 |
| Stachys rizeensis | 0 | 1 | 0 | 0 | 0 | 0 | 0 | 0 | 0 |
| Stachys subnuda | 0 | 1 | 0 | 0 | 0 | 0 | 0 | 0 | 0 |
| Stefanoffia insoluta | 1 | 0 | 0 | 0 | 0 | 0 | 0 | 0 | 0 |
| Stellaria graminea | 0 | 0 | 1 | 0 | 0 | 1 | 0 | 0 | 0 |
| Stellaria persica | 0 | 0 | 1 | 1 | 0 | 1 | 0 | 0 | 0 |
| Stellaria scaturiginella | 0 | 0 | 1 | 0 | 0 | 0 | 0 | 0 | 0 |
| Stenotaenia elbursensis | 0 | 0 | 1 | 0 | 0 | 0 | 0 | 0 | 0 |
| Stenotaenia haussknechtii | 0 | 0 | 0 | 1 | 0 | 0 | 0 | 0 | 0 |
| Stenotaenia nudicaulis | 0 | 0 | 1 | 0 | 0 | 1 | 0 | 0 | 0 |
| Stipa hohenackeriana | 0 | 1 | 1 | 1 | 0 | 1 | 1 | 0 | 0 |
| Stipa joannis | 0 | 1 | 0 | 0 | 0 | 0 | 0 | 0 | 0 |
| Stipa pennata | 0 | 1 | 1 | 0 | 1 | 0 | 1 | 0 | 0 |
| Stipa tirsa | 0 | 1 | 0 | 0 | 0 | 0 | 0 | 0 | 0 |
| Swertia iberica | 0 | 1 | 0 | 0 | 0 | 0 | 0 | 0 | 0 |
| Swertia lactea | 0 | 0 | 1 | 0 | 0 | 0 | 0 | 0 | 0 |
| Swertia longifolia | 1 | 1 | 1 | 1 | 0 | 0 | 0 | 0 | 0 |
| Tanacetum bachtiaricum | 0 | 0 | 0 | 1 | 0 | 0 | 0 | 0 | 0 |
| Tanacetum balsamita | 1 | 1 | 1 | 1 | 0 | 1 | 1 | 0 | 0 |
| Tanacetum cappadocicum | 0 | 1 | 0 | 0 | 0 | 0 | 0 | 0 | 0 |
| Tanacetum caucasicum | 0 | 1 | 0 | 0 | 0 | 1 | 1 | 0 | 0 |
| Tanacetum chiliophyllum | 0 | 1 | 1 | 0 | 0 | 1 | 1 | 0 | 0 |
| Tanacetum coccineum | 0 | 1 | 0 | 0 | 0 | 0 | 0 | 0 | 0 |
| Tanacetum dumosum | 0 | 0 | 0 | 1 | 0 | 0 | 0 | 0 | 0 |
| Tanacetum fruticulosum | 0 | 0 | 1 | 1 | 1 | 0 | 0 | 0 | 0 |
| Tanacetum hololeucum | 0 | 0 | 1 | 0 | 0 | 0 | 0 | 0 | 0 |
| Tanacetum kotschyi | 1 | 1 | 1 | 1 | 1 | 1 | 1 | 0 | 0 |
| Tanacetum macrophyllum | 0 | 1 | 0 | 0 | 0 | 0 | 0 | 0 | 0 |
| Tanacetum mucroniferum | 0 | 1 | 0 | 0 | 0 | 0 | 0 | 0 | 0 |
| Tanacetum nitens | 1 | 1 | 0 | 0 | 0 | 0 | 0 | 0 | 0 |
| Tanacetum nivale | 0 | 1 | 0 | 0 | 0 | 0 | 0 | 0 | 0 |
| Tanacetum pamiricum | 0 | 0 | 0 | 0 | 1 | 0 | 0 | 0 | 0 |
| Tanacetum persicum | 0 | 0 | 1 | 1 | 0 | 0 | 1 | 1 | 0 |
| Tanacetum polycephalum | 0 | 1 | 1 | 1 | 0 | 1 | 0 | 0 | 0 |
| Tanacetum punctatum | 0 | 1 | 0 | 0 | 0 | 0 | 0 | 0 | 0 |
| Tanacetum tenuisectum | 0 | 0 | 1 | 1 | 0 | 0 | 0 | 0 | 0 |
| Tanacetum zahlbruckneri | 0 | 1 | 0 | 0 | 0 | 0 | 0 | 0 | 0 |
| Tanacetum zangezuricum | 0 | 1 | 0 | 0 | 0 | 0 | 0 | 0 | 0 |
| Taraxacum baltistanicum | 0 | 0 | 1 | 0 | 0 | 0 | 0 | 0 | 0 |
| Taraxacum brevirostre | 0 | 1 | 1 | 0 | 0 | 0 | 0 | 0 | 0 |
| Taraxacum chitralense | 0 | 0 | 0 | 0 | 1 | 0 | 0 | 0 | 0 |
| Taraxacum koelzii | 0 | 0 | 1 | 0 | 0 | 1 | 0 | 0 | 0 |
| Taraxacum neospurium | 0 | 0 | 1 | 0 | 0 | 0 | 1 | 0 | 0 |
| Taraxacum oliganthum | 1 | 1 | 1 | 0 | 0 | 0 | 0 | 0 | 0 |
| Taraxacum primigenium | 0 | 0 | 0 | 0 | 1 | 0 | 1 | 0 | 0 |
| Taraxacum scolopendrinum | 0 | 1 | 0 | 0 | 0 | 0 | 0 | 0 | 0 |
| Taraxacum stenolepium | 0 | 0 | 0 | 1 | 0 | 0 | 0 | 0 | 0 |
| Taraxacum stevenii | 1 | 1 | 1 | 0 | 0 | 1 | 0 | 0 | 0 |
| Tetrataenium lasiopetalum | 0 | 1 | 0 | 1 | 0 | 0 | 1 | 1 | 0 |
| Teucrium ozturkii | 0 | 1 | 0 | 0 | 0 | 0 | 0 | 0 | 0 |
| Thesium cilicicum | 1 | 0 | 0 | 0 | 0 | 0 | 0 | 0 | 0 |
| Thlaspi crassum | 1 | 0 | 0 | 0 | 0 | 0 | 0 | 0 | 0 |
| Thlaspi kurdicum | 0 | 1 | 0 | 0 | 0 | 0 | 1 | 0 | 0 |
| Thlaspi maassoumii | 0 | 0 | 1 | 0 | 0 | 0 | 0 | 0 | 0 |
| Thlaspi pulvinata | 0 | 0 | 0 | 0 | 1 | 0 | 0 | 0 | 0 |
| Thlaspi pulvinatum | 0 | 1 | 0 | 0 | 0 | 0 | 0 | 0 | 0 |
| Thlaspi pumilum | 0 | 0 | 1 | 0 | 0 | 0 | 0 | 0 | 0 |
| Thlaspi sintenisii | 0 | 1 | 0 | 0 | 0 | 0 | 0 | 0 | 0 |
| Thlaspi stenocarpum | 0 | 0 | 1 | 0 | 0 | 0 | 0 | 0 | 0 |
| Thlaspi tenue | 0 | 0 | 0 | 0 | 0 | 1 | 0 | 1 | 0 |
| Thlaspi valerianoides | 0 | 1 | 0 | 0 | 0 | 1 | 0 | 0 | 0 |
| Thlaspi watsonii | 0 | 1 | 0 | 0 | 0 | 0 | 0 | 0 | 0 |
| Thymus brachychilus | 1 | 1 | 0 | 0 | 0 | 0 | 0 | 0 | 0 |
| Thymus carmanicus | 0 | 0 | 1 | 1 | 1 | 0 | 1 | 0 | 0 |
| Thymus cherlerioides | 1 | 0 | 0 | 0 | 0 | 0 | 0 | 0 | 0 |
| Thymus collinus | 0 | 1 | 0 | 0 | 0 | 0 | 0 | 0 | 0 |
| Thymus fallax | 0 | 0 | 1 | 1 | 0 | 0 | 0 | 0 | 0 |
| Thymus fedtschenkoi | 0 | 1 | 0 | 0 | 0 | 0 | 0 | 0 | 0 |
| Thymus praecox | 0 | 1 | 1 | 1 | 0 | 1 | 0 | 0 | 0 |
| Thymus pubescens | 0 | 1 | 1 | 1 | 0 | 1 | 0 | 1 | 0 |
| Trachydium depressum | 0 | 1 | 1 | 1 | 1 | 0 | 1 | 0 | 0 |
| Trachydium eriocarpum | 0 | 0 | 1 | 0 | 0 | 0 | 0 | 0 | 0 |
| Trachydium kotschyi | 0 | 0 | 0 | 1 | 0 | 0 | 0 | 0 | 0 |
| Trachydium pauciradiatum | 0 | 0 | 1 | 0 | 0 | 0 | 0 | 0 | 0 |
| Tragopogon erostris | 0 | 0 | 0 | 1 | 0 | 0 | 0 | 0 | 0 |
| Tragopogon jesdianus | 0 | 0 | 1 | 1 | 0 | 0 | 0 | 0 | 0 |
| Tragopogon kotschyi | 0 | 0 | 1 | 0 | 0 | 0 | 0 | 0 | 0 |
| Tragopogon pusillus | 0 | 1 | 0 | 0 | 0 | 0 | 0 | 0 | 0 |
| Tragopogon reticulatus | 0 | 1 | 0 | 0 | 0 | 0 | 0 | 0 | 0 |
| Trichophorum pumilum | 0 | 0 | 1 | 1 | 0 | 1 | 0 | 0 | 0 |
| Trifolium badium | 0 | 1 | 1 | 0 | 0 | 1 | 1 | 0 | 0 |
| Trifolium kurdistanicum | 0 | 0 | 0 | 1 | 0 | 0 | 0 | 0 | 0 |
| Trifolium longidentatum | 0 | 1 | 0 | 0 | 0 | 1 | 0 | 0 | 0 |
| Trifolium montanum | 0 | 1 | 0 | 0 | 0 | 0 | 0 | 0 | 0 |
| Trifolium polyphyllum | 0 | 1 | 0 | 0 | 0 | 0 | 0 | 0 | 0 |
| Trifolium pratense | 0 | 1 | 1 | 1 | 1 | 1 | 1 | 0 | 0 |
| Trifolium radicosum | 0 | 0 | 1 | 0 | 0 | 0 | 1 | 0 | 0 |
| Trifolium repens | 0 | 0 | 1 | 1 | 1 | 0 | 1 | 0 | 0 |
| Trifolium sintenisii | 0 | 1 | 0 | 0 | 0 | 0 | 0 | 0 | 0 |
| Trifolium spadiceum | 0 | 0 | 0 | 0 | 0 | 1 | 0 | 0 | 0 |
| Tripleurospermum caucasicum | 0 | 1 | 0 | 0 | 0 | 1 | 0 | 0 | 0 |
| Tripleurospermum melanolepis | 0 | 1 | 0 | 0 | 0 | 0 | 0 | 0 | 0 |
| Trisetum geghamense | 0 | 1 | 0 | 0 | 0 | 0 | 0 | 0 | 0 |
| Trisetum rigidum | 0 | 1 | 1 | 0 | 0 | 1 | 0 | 0 | 0 |
| Trisetum thospiticum | 0 | 1 | 0 | 0 | 0 | 0 | 0 | 0 | 0 |
| Trisetum turcicum | 1 | 1 | 0 | 0 | 0 | 0 | 0 | 0 | 0 |
| Trollius ranunculoides | 1 | 1 | 0 | 0 | 0 | 0 | 0 | 0 | 0 |
| Tulipa humilis | 1 | 1 | 1 | 1 | 0 | 1 | 0 | 0 | 0 |
| Tulipa koyuncui | 0 | 0 | 0 | 0 | 0 | 1 | 0 | 0 | 0 |
| Valeriana alliariifolia | 1 | 1 | 1 | 0 | 0 | 1 | 1 | 0 | 0 |
| Valeriana alpestris | 0 | 1 | 0 | 0 | 0 | 0 | 0 | 0 | 0 |
| Valeriana bolkarica | 1 | 0 | 0 | 0 | 0 | 0 | 0 | 0 | 0 |
| Valeriana clarkei | 0 | 1 | 1 | 1 | 0 | 0 | 0 | 0 | 0 |
| Valeriana montana | 0 | 1 | 0 | 0 | 0 | 0 | 0 | 0 | 0 |
| Valeriana saxicola | 0 | 1 | 0 | 0 | 0 | 0 | 0 | 0 | 0 |
| Valeriana sisymbriifolia | 1 | 1 | 1 | 1 | 0 | 1 | 1 | 0 | 0 |
| Vania campylophylla | 0 | 1 | 0 | 0 | 0 | 0 | 0 | 0 | 0 |
| Vavilovia formosa | 1 | 1 | 1 | 0 | 0 | 0 | 1 | 0 | 0 |
| Verbascum bornmuellerianum | 0 | 0 | 0 | 1 | 0 | 0 | 1 | 0 | 0 |
| Verbascum bourgeauanum | 1 | 0 | 0 | 0 | 0 | 0 | 0 | 0 | 0 |
| Verbascum carmanicum | 0 | 0 | 0 | 0 | 1 | 0 | 0 | 0 | 0 |
| Verbascum faik-karaveliogullarii | 0 | 1 | 0 | 0 | 0 | 0 | 0 | 0 | 0 |
| Verbascum georgicum | 0 | 1 | 0 | 0 | 0 | 0 | 0 | 0 | 0 |
| Verbascum kurdistanicum | 0 | 1 | 0 | 0 | 0 | 0 | 0 | 0 | 0 |
| Verbascum speciosum | 0 | 1 | 0 | 0 | 0 | 0 | 0 | 0 | 0 |
| Verbascum subnivale | 1 | 0 | 0 | 0 | 0 | 0 | 0 | 0 | 0 |
| Verbascum tauri | 1 | 0 | 0 | 0 | 0 | 0 | 0 | 0 | 0 |
| Veronica allahuekberensis | 0 | 1 | 0 | 0 | 0 | 0 | 0 | 0 | 0 |
| Veronica armena | 0 | 1 | 0 | 0 | 0 | 0 | 0 | 0 | 0 |
| Veronica aucheri | 0 | 0 | 1 | 0 | 0 | 0 | 0 | 0 | 0 |
| Veronica baranetzkii | 0 | 1 | 0 | 0 | 0 | 0 | 0 | 0 | 0 |
| Veronica beccabunga | 1 | 1 | 1 | 0 | 0 | 1 | 1 | 0 | 0 |
| Veronica biloba | 1 | 1 | 1 | 1 | 1 | 1 | 1 | 0 | 0 |
| Veronica bombycina | 1 | 1 | 0 | 0 | 0 | 0 | 0 | 0 | 0 |
| Veronica caespitosa | 1 | 1 | 0 | 0 | 0 | 0 | 0 | 0 | 1 |
| Veronica daranica | 0 | 0 | 0 | 1 | 1 | 0 | 0 | 0 | 0 |
| Veronica davisii | 0 | 1 | 0 | 0 | 0 | 0 | 0 | 0 | 0 |
| Veronica fragilis | 0 | 0 | 0 | 1 | 0 | 0 | 0 | 0 | 0 |
| Veronica fridericae | 0 | 1 | 0 | 0 | 0 | 0 | 0 | 0 | 0 |
| Veronica gentianoides | 1 | 1 | 1 | 0 | 0 | 1 | 1 | 0 | 1 |
| Veronica hispidula | 1 | 1 | 1 | 1 | 0 | 0 | 1 | 0 | 0 |
| Veronica kopetdaghensis | 0 | 0 | 1 | 0 | 0 | 0 | 0 | 0 | 0 |
| Veronica kopgecidiensis | 0 | 1 | 0 | 0 | 0 | 0 | 0 | 0 | 0 |
| Veronica kotschyana | 1 | 0 | 0 | 0 | 0 | 0 | 0 | 0 | 0 |
| Veronica kurdica | 0 | 0 | 1 | 1 | 1 | 0 | 0 | 0 | 0 |
| Veronica longipedicellata | 0 | 0 | 1 | 0 | 0 | 0 | 0 | 0 | 0 |
| Veronica mirabilis | 0 | 0 | 1 | 0 | 0 | 0 | 0 | 0 | 0 |
| Veronica montbretii | 0 | 1 | 0 | 0 | 0 | 0 | 0 | 0 | 0 |
| Veronica orientalis | 0 | 1 | 1 | 1 | 0 | 1 | 1 | 1 | 0 |
| Veronica paederotae | 0 | 0 | 1 | 0 | 0 | 0 | 0 | 0 | 0 |
| Veronica polium | 0 | 1 | 0 | 0 | 0 | 0 | 0 | 0 | 0 |
| Veronica pusilla | 1 | 1 | 1 | 1 | 1 | 1 | 1 | 0 | 0 |
| Veronica quezelii | 1 | 0 | 0 | 0 | 0 | 0 | 0 | 0 | 0 |
| Veronica rechingeri | 0 | 0 | 1 | 0 | 0 | 0 | 0 | 0 | 0 |
| Veronica rubrifolia | 0 | 0 | 1 | 1 | 1 | 0 | 0 | 0 | 0 |
| Veronica surculosa | 1 | 0 | 0 | 0 | 0 | 0 | 0 | 0 | 1 |
| Veronica tauricola | 1 | 1 | 0 | 0 | 0 | 0 | 0 | 0 | 1 |
| Veronica telephiifolia | 0 | 1 | 0 | 0 | 0 | 0 | 0 | 0 | 0 |
| Veronica thymoides | 0 | 1 | 0 | 0 | 0 | 0 | 0 | 0 | 0 |
| Vicia akhmaganica | 0 | 0 | 1 | 0 | 0 | 1 | 0 | 0 | 0 |
| Vicia alpestris | 1 | 1 | 0 | 0 | 0 | 0 | 0 | 0 | 0 |
| Vicia canescens | 1 | 1 | 1 | 0 | 0 | 1 | 1 | 0 | 0 |
| Vicia ciceroidea | 0 | 1 | 1 | 1 | 0 | 0 | 1 | 0 | 0 |
| Vicia glareosa | 0 | 1 | 0 | 0 | 0 | 0 | 0 | 0 | 0 |
| Vicia multijuga | 0 | 0 | 1 | 0 | 0 | 1 | 0 | 0 | 0 |
| Viola oreades | 0 | 1 | 0 | 0 | 0 | 0 | 0 | 0 | 0 |
| Viola pachyrrhiza | 0 | 0 | 0 | 1 | 0 | 0 | 0 | 0 | 0 |
| Viola rupestris | 0 | 0 | 1 | 0 | 0 | 1 | 0 | 0 | 0 |
| Viola spathulata | 0 | 0 | 1 | 0 | 0 | 0 | 0 | 0 | 0 |
| Woodsia alpina | 0 | 1 | 0 | 0 | 0 | 0 | 0 | 0 | 0 |
| Xanthogalum purpurascens | 1 | 1 | 1 | 0 | 0 | 1 | 0 | 0 | 0 |
| Zeravschania aucheri | 0 | 1 | 1 | 1 | 0 | 1 | 0 | 0 | 0 |
| Zerdana anchonioides | 0 | 0 | 0 | 1 | 0 | 0 | 0 | 0 | 0 |
| Ziziphora clinopodioides | 1 | 1 | 1 | 0 | 0 | 1 | 0 | 0 | 0 |
| **Total species** | **300** | **910** | **519** | **446** | **161** | **326** | **162** | **36** | **68** |

**Table S5** Species contributing to the Bioregions using endemic alpine species dataset and Network-Clustering approach.

| **Bioregions** | **Taurus** | **Anatolian-Armenian** | **Alborz** | **Zagros** | Transitional Zone | | | | |
| --- | --- | --- | --- | --- | --- | --- | --- | --- | --- |
|  | **1** | **2** | **3** | **4** | 6 | 7 | 8 | 9 | 11 |
| Acantholimon artosense | 0 | 0 | 0 | 0 | 0 | 1 | 0 | 0 | 0 |
| Acantholimon brachystachyum | 0 | 0 | 1 | 1 | 0 | 0 | 0 | 0 | 0 |
| Acantholimon calvertii | 1 | 1 | 0 | 0 | 0 | 1 | 0 | 0 | 0 |
| Acantholimon cupreo-olivascens | 0 | 0 | 0 | 1 | 0 | 0 | 0 | 0 | 0 |
| Acantholimon demavendicum | 0 | 0 | 1 | 0 | 0 | 0 | 0 | 0 | 0 |
| Acantholimon dianthifolium | 0 | 0 | 0 | 0 | 0 | 1 | 0 | 0 | 0 |
| Acantholimon ekimii | 0 | 1 | 0 | 0 | 0 | 0 | 0 | 0 | 0 |
| Acantholimon eschkerense | 0 | 0 | 0 | 1 | 0 | 0 | 0 | 0 | 0 |
| Acantholimon haesarensis | 0 | 0 | 0 | 1 | 0 | 0 | 0 | 0 | 0 |
| Acantholimon hohenackeri | 0 | 1 | 1 | 0 | 1 | 1 | 0 | 0 | 0 |
| Acantholimon kermanense | 0 | 0 | 0 | 1 | 0 | 0 | 0 | 0 | 0 |
| Acantholimon mirtadzadinii | 0 | 0 | 0 | 1 | 0 | 0 | 0 | 0 | 0 |
| Acantholimon modestum | 0 | 0 | 0 | 1 | 0 | 0 | 0 | 0 | 0 |
| Acantholimon nigricans | 0 | 0 | 0 | 1 | 0 | 0 | 0 | 0 | 0 |
| Acantholimon oliganthum | 0 | 0 | 0 | 1 | 0 | 0 | 0 | 0 | 0 |
| Acantholimon sahendicum | 0 | 1 | 0 | 0 | 1 | 1 | 0 | 0 | 0 |
| Acantholimon scabrellum | 0 | 0 | 0 | 1 | 0 | 0 | 0 | 0 | 0 |
| Acantholimon tomentellum | 0 | 0 | 0 | 1 | 0 | 0 | 0 | 0 | 0 |
| Acantholimon ulicinum | 1 | 1 | 0 | 0 | 0 | 1 | 0 | 0 | 0 |
| Acantholimon zaeifii | 0 | 0 | 0 | 1 | 0 | 0 | 0 | 0 | 0 |
| Achillea armenorum | 1 | 0 | 0 | 0 | 0 | 0 | 0 | 0 | 0 |
| Achillea aucheri | 0 | 0 | 1 | 0 | 1 | 0 | 0 | 0 | 0 |
| Achillea kellalensis | 0 | 0 | 0 | 1 | 0 | 0 | 0 | 0 | 0 |
| Achillea latiloba | 0 | 1 | 0 | 0 | 0 | 0 | 0 | 0 | 0 |
| Achillea millefolium | 0 | 0 | 1 | 0 | 0 | 0 | 0 | 0 | 0 |
| Achillea vermicularis | 0 | 1 | 1 | 1 | 1 | 1 | 1 | 0 | 0 |
| Aconitum anthora | 0 | 1 | 0 | 0 | 0 | 0 | 0 | 0 | 0 |
| Aconitum cochleare | 0 | 1 | 0 | 0 | 1 | 1 | 0 | 0 | 0 |
| Aethionema caespitosum | 0 | 1 | 0 | 0 | 0 | 0 | 0 | 0 | 0 |
| Aethionema fimbriatum | 0 | 0 | 1 | 1 | 0 | 1 | 1 | 0 | 0 |
| Aethionema munzurense | 0 | 1 | 0 | 0 | 0 | 0 | 0 | 0 | 0 |
| Aethionema oppositifolium | 1 | 1 | 0 | 0 | 0 | 0 | 0 | 0 | 0 |
| Aethionema papillosum | 1 | 0 | 0 | 0 | 0 | 0 | 0 | 0 | 0 |
| Aethionema semnanensis | 0 | 0 | 1 | 0 | 0 | 0 | 0 | 0 | 0 |
| Aethionema speciosum | 1 | 1 | 0 | 0 | 0 | 1 | 0 | 0 | 0 |
| Aethionema stenopterum | 0 | 0 | 1 | 0 | 0 | 0 | 0 | 0 | 0 |
| Aethionema subulatum | 1 | 0 | 0 | 0 | 0 | 0 | 0 | 0 | 0 |
| Aethionema umbellatum | 0 | 0 | 0 | 1 | 0 | 0 | 0 | 0 | 0 |
| Aethionema virgatum | 0 | 0 | 1 | 1 | 1 | 1 | 0 | 0 | 0 |
| Alchemilla amardica | 0 | 0 | 1 | 0 | 0 | 0 | 0 | 0 | 0 |
| Alchemilla basakii | 0 | 1 | 0 | 0 | 0 | 0 | 0 | 0 | 0 |
| Alchemilla ciminensis | 0 | 1 | 0 | 0 | 0 | 0 | 0 | 0 | 0 |
| Alchemilla citrina | 0 | 0 | 1 | 0 | 1 | 0 | 0 | 0 | 0 |
| Alchemilla compactilis | 1 | 1 | 1 | 0 | 0 | 1 | 0 | 0 | 1 |
| Alchemilla erzincanensis | 0 | 1 | 0 | 0 | 0 | 0 | 0 | 0 | 0 |
| Alchemilla farinosa | 0 | 0 | 1 | 0 | 1 | 0 | 0 | 0 | 0 |
| Alchemilla fluminea | 0 | 0 | 1 | 0 | 1 | 0 | 0 | 0 | 0 |
| Alchemilla gigantodus | 0 | 0 | 1 | 0 | 0 | 0 | 0 | 0 | 0 |
| Alchemilla hemsinica | 0 | 1 | 0 | 0 | 0 | 0 | 0 | 0 | 0 |
| Alchemilla hessii | 0 | 0 | 1 | 0 | 1 | 1 | 0 | 0 | 0 |
| Alchemilla kackarensis | 0 | 1 | 0 | 0 | 0 | 0 | 0 | 0 | 0 |
| Alchemilla kurdica | 0 | 0 | 1 | 1 | 0 | 0 | 1 | 0 | 0 |
| Alchemilla melancholica | 0 | 0 | 1 | 0 | 0 | 0 | 0 | 0 | 0 |
| Alchemilla microscopica | 0 | 0 | 1 | 0 | 0 | 0 | 0 | 0 | 0 |
| Alchemilla oriturcica | 0 | 1 | 0 | 0 | 0 | 0 | 0 | 0 | 0 |
| Alchemilla ovitensis | 0 | 1 | 0 | 0 | 0 | 0 | 0 | 0 | 0 |
| Alchemilla paracompactilis | 1 | 0 | 0 | 0 | 0 | 0 | 0 | 0 | 0 |
| Alchemilla pectiniloba | 0 | 0 | 1 | 0 | 0 | 0 | 0 | 0 | 0 |
| Alchemilla persica | 0 | 1 | 1 | 1 | 1 | 1 | 1 | 0 | 0 |
| Alchemilla plicatissima | 0 | 0 | 1 | 0 | 0 | 0 | 0 | 0 | 0 |
| Alchemilla procerrima | 0 | 1 | 0 | 0 | 0 | 0 | 0 | 0 | 1 |
| Alchemilla rivularis | 1 | 0 | 0 | 0 | 0 | 0 | 0 | 0 | 0 |
| Alchemilla rizensis | 0 | 1 | 0 | 0 | 0 | 0 | 0 | 0 | 0 |
| Alchemilla surculosa | 0 | 1 | 1 | 0 | 0 | 1 | 0 | 0 | 0 |
| Alchemilla tiryalensis | 0 | 1 | 0 | 0 | 0 | 0 | 0 | 0 | 0 |
| Alkanna bracteosa | 0 | 0 | 1 | 1 | 1 | 1 | 0 | 0 | 0 |
| Alkanna frigida | 0 | 0 | 1 | 1 | 0 | 0 | 0 | 0 | 0 |
| Allium akaka | 0 | 1 | 1 | 0 | 1 | 1 | 0 | 0 | 0 |
| Allium alamutense | 0 | 0 | 1 | 0 | 0 | 0 | 0 | 0 | 0 |
| Allium alpinarii | 1 | 0 | 0 | 0 | 0 | 0 | 0 | 0 | 0 |
| Allium anacoleum | 0 | 0 | 0 | 0 | 0 | 1 | 0 | 0 | 0 |
| Allium arlgirdense | 0 | 0 | 0 | 0 | 0 | 1 | 0 | 0 | 0 |
| Allium aucheri | 0 | 1 | 0 | 0 | 0 | 1 | 0 | 0 | 0 |
| Allium austroiranicum | 0 | 0 | 0 | 1 | 0 | 0 | 0 | 0 | 0 |
| Allium balansae | 1 | 1 | 0 | 0 | 0 | 0 | 0 | 0 | 0 |
| Allium brachyodon | 0 | 0 | 0 | 1 | 0 | 0 | 0 | 0 | 0 |
| Allium breviscapum | 0 | 0 | 0 | 1 | 0 | 0 | 0 | 1 | 0 |
| Allium capitellatum | 0 | 0 | 1 | 1 | 0 | 0 | 0 | 0 | 0 |
| Allium cathodicarpum | 0 | 0 | 0 | 1 | 0 | 0 | 0 | 0 | 0 |
| Allium derderianum | 0 | 1 | 1 | 0 | 0 | 0 | 0 | 0 | 0 |
| Allium djimilense | 0 | 1 | 0 | 0 | 0 | 0 | 0 | 0 | 0 |
| Allium dumanii | 1 | 0 | 0 | 0 | 0 | 0 | 0 | 0 | 0 |
| Allium egorovae | 0 | 1 | 0 | 0 | 1 | 0 | 0 | 0 | 0 |
| Allium elburzense | 0 | 0 | 1 | 0 | 0 | 0 | 0 | 0 | 0 |
| Allium flavum | 1 | 0 | 0 | 0 | 0 | 0 | 0 | 0 | 0 |
| Allium hoshabicum | 0 | 0 | 0 | 0 | 0 | 1 | 0 | 0 | 0 |
| Allium hymenorhizum | 0 | 0 | 1 | 0 | 0 | 0 | 0 | 0 | 0 |
| Allium iranshahrii | 0 | 0 | 0 | 0 | 0 | 1 | 1 | 0 | 0 |
| Allium kuhrangense | 0 | 0 | 0 | 1 | 0 | 0 | 0 | 0 | 0 |
| Allium lalesaricum | 0 | 0 | 0 | 1 | 0 | 0 | 0 | 0 | 0 |
| Allium longivaginatum | 0 | 0 | 0 | 1 | 0 | 0 | 0 | 0 | 0 |
| Allium mahneshanense | 0 | 0 | 0 | 0 | 1 | 0 | 0 | 1 | 0 |
| Allium microspathum | 0 | 0 | 0 | 0 | 0 | 1 | 0 | 0 | 0 |
| Allium montelburzense | 0 | 0 | 1 | 0 | 0 | 0 | 0 | 0 | 0 |
| Allium pseudoampeloprasum | 0 | 1 | 0 | 0 | 0 | 1 | 0 | 0 | 0 |
| Allium rhetoreanum | 0 | 0 | 0 | 0 | 0 | 1 | 0 | 0 | 0 |
| Allium sabalense | 0 | 0 | 0 | 0 | 1 | 0 | 0 | 0 | 0 |
| Allium sahandicum | 0 | 0 | 0 | 0 | 1 | 0 | 0 | 0 | 0 |
| Allium scotostemon | 0 | 0 | 1 | 0 | 0 | 0 | 0 | 0 | 0 |
| Allium shatakiense | 0 | 1 | 0 | 0 | 0 | 1 | 0 | 0 | 0 |
| Allium stearnianum | 0 | 0 | 0 | 0 | 0 | 1 | 0 | 0 | 0 |
| Allium straussii | 0 | 0 | 0 | 1 | 0 | 0 | 0 | 0 | 0 |
| Allium szovitsii | 0 | 1 | 0 | 0 | 0 | 1 | 0 | 0 | 0 |
| Allium talyschense | 0 | 1 | 0 | 0 | 0 | 0 | 0 | 0 | 0 |
| Allium tauricola | 1 | 1 | 0 | 0 | 0 | 1 | 0 | 0 | 0 |
| Allium tuchalense | 0 | 0 | 1 | 0 | 0 | 0 | 0 | 0 | 0 |
| Alopecurus aucheri | 0 | 1 | 1 | 0 | 1 | 1 | 0 | 0 | 0 |
| Alopecurus laguroides | 0 | 1 | 0 | 0 | 0 | 1 | 0 | 0 | 0 |
| Alopecurus lanatus | 1 | 0 | 0 | 0 | 0 | 0 | 0 | 0 | 0 |
| Alopecurus textilis | 1 | 1 | 1 | 1 | 1 | 1 | 1 | 0 | 0 |
| Alyssopsis mollis | 0 | 0 | 1 | 0 | 1 | 0 | 0 | 0 | 0 |
| Alyssum aizoides | 1 | 1 | 0 | 0 | 0 | 0 | 0 | 0 | 0 |
| Alyssum armenum | 0 | 1 | 0 | 0 | 0 | 1 | 0 | 0 | 0 |
| Alyssum aurantiacum | 1 | 0 | 0 | 0 | 0 | 0 | 0 | 0 | 0 |
| Alyssum gehamense | 0 | 1 | 0 | 0 | 0 | 0 | 0 | 0 | 0 |
| Alyssum haussknechtii | 1 | 0 | 0 | 0 | 0 | 0 | 0 | 0 | 0 |
| Alyssum muelleri | 0 | 1 | 1 | 0 | 1 | 0 | 0 | 0 | 0 |
| Alyssum peltarioides | 1 | 1 | 0 | 0 | 1 | 1 | 0 | 0 | 1 |
| Alyssum persicum | 0 | 0 | 0 | 1 | 0 | 0 | 0 | 0 | 0 |
| Alyssum polycladum | 0 | 0 | 1 | 1 | 1 | 0 | 0 | 0 | 0 |
| Alyssum propinquum | 1 | 0 | 0 | 0 | 0 | 0 | 0 | 0 | 0 |
| Amygdalus carduchorum | 0 | 0 | 0 | 0 | 0 | 1 | 0 | 0 | 0 |
| Amygdalus elaeagnifolia | 0 | 0 | 0 | 1 | 0 | 0 | 0 | 0 | 0 |
| Anchonium elichrysifolium | 1 | 1 | 1 | 0 | 1 | 1 | 1 | 0 | 0 |
| Androsace armeniaca | 0 | 1 | 0 | 0 | 0 | 0 | 0 | 0 | 0 |
| Androsace multiscapa | 1 | 1 | 0 | 0 | 0 | 0 | 0 | 0 | 0 |
| Arabis androsacea | 1 | 0 | 0 | 0 | 0 | 0 | 0 | 0 | 0 |
| Arabis brachycarpa | 0 | 1 | 0 | 0 | 0 | 0 | 0 | 0 | 0 |
| Arabis carduchorum | 0 | 1 | 0 | 0 | 1 | 1 | 0 | 0 | 0 |
| Arabis caucasica | 1 | 1 | 1 | 1 | 1 | 1 | 1 | 1 | 0 |
| Arabis graellsiiformis | 0 | 0 | 0 | 0 | 0 | 1 | 0 | 0 | 0 |
| Arabis rimarum | 0 | 0 | 1 | 0 | 0 | 0 | 0 | 0 | 0 |
| Arenaria angustisepala | 0 | 0 | 0 | 0 | 0 | 1 | 0 | 0 | 0 |
| Arenaria antitaurica | 1 | 0 | 0 | 0 | 0 | 0 | 0 | 0 | 0 |
| Arenaria balansae | 1 | 0 | 0 | 1 | 0 | 1 | 0 | 0 | 0 |
| Arenaria blepharophylla | 0 | 1 | 0 | 0 | 0 | 1 | 0 | 0 | 0 |
| Arenaria bulica | 0 | 0 | 0 | 1 | 0 | 0 | 0 | 0 | 0 |
| Arenaria cucubaloides | 1 | 1 | 0 | 1 | 1 | 1 | 0 | 0 | 0 |
| Arenaria davisii | 0 | 0 | 0 | 0 | 0 | 1 | 0 | 0 | 0 |
| Arenaria dianthoides | 0 | 1 | 0 | 0 | 1 | 1 | 0 | 0 | 0 |
| Arenaria gypsophiloides | 0 | 1 | 1 | 1 | 1 | 1 | 1 | 0 | 1 |
| Arenaria ledebouriana | 1 | 0 | 0 | 0 | 0 | 0 | 0 | 0 | 0 |
| Arenaria minutissima | 0 | 0 | 0 | 1 | 0 | 0 | 0 | 0 | 0 |
| Arenaria persica | 0 | 0 | 0 | 1 | 0 | 0 | 0 | 0 | 0 |
| Arenaria semiromica | 0 | 0 | 0 | 1 | 0 | 0 | 0 | 0 | 0 |
| Arnebia pulchra | 0 | 1 | 1 | 1 | 0 | 0 | 0 | 0 | 0 |
| Artemisia haussknechtii | 0 | 0 | 0 | 1 | 0 | 1 | 0 | 0 | 0 |
| Artemisia melanolepis | 0 | 0 | 1 | 0 | 1 | 0 | 0 | 0 | 0 |
| Artemisia splendens | 0 | 1 | 1 | 0 | 1 | 1 | 0 | 0 | 0 |
| Asperula affinis | 0 | 1 | 0 | 0 | 0 | 0 | 0 | 0 | 0 |
| Asperula capitellata | 1 | 0 | 0 | 0 | 0 | 1 | 0 | 0 | 0 |
| Asperula fragillima | 0 | 0 | 0 | 1 | 0 | 0 | 0 | 0 | 0 |
| Asperula laxiflora | 0 | 1 | 0 | 0 | 0 | 1 | 0 | 0 | 0 |
| Asperula prostrata | 0 | 1 | 0 | 0 | 1 | 1 | 1 | 0 | 0 |
| Asperula rechingeri | 0 | 0 | 0 | 1 | 0 | 0 | 0 | 0 | 0 |
| Astragalus abditus | 0 | 0 | 0 | 1 | 0 | 0 | 0 | 0 | 0 |
| Astragalus acmophyllus | 1 | 1 | 0 | 0 | 0 | 0 | 0 | 0 | 0 |
| Astragalus aegobromus | 0 | 1 | 1 | 1 | 1 | 1 | 1 | 1 | 0 |
| Astragalus aestivorum | 0 | 0 | 1 | 0 | 0 | 0 | 0 | 0 | 0 |
| Astragalus agassii | 0 | 1 | 0 | 0 | 0 | 0 | 0 | 0 | 0 |
| Astragalus aladagensis | 1 | 0 | 0 | 0 | 0 | 0 | 0 | 0 | 0 |
| Astragalus alamkuhensis | 0 | 0 | 1 | 0 | 0 | 0 | 0 | 0 | 0 |
| Astragalus alyssoides | 0 | 1 | 1 | 1 | 1 | 1 | 0 | 1 | 1 |
| Astragalus argaeus | 1 | 0 | 0 | 0 | 0 | 0 | 0 | 0 | 0 |
| Astragalus atricapillus | 0 | 0 | 1 | 0 | 0 | 0 | 0 | 0 | 0 |
| Astragalus aureus | 0 | 1 | 1 | 0 | 1 | 1 | 0 | 1 | 1 |
| Astragalus azizii | 0 | 0 | 0 | 0 | 1 | 0 | 0 | 0 | 0 |
| Astragalus bahcesarayensis | 0 | 0 | 0 | 0 | 0 | 1 | 0 | 0 | 0 |
| Astragalus barnassari | 0 | 0 | 1 | 0 | 1 | 1 | 1 | 0 | 0 |
| Astragalus bashkalensis | 0 | 0 | 0 | 0 | 1 | 0 | 0 | 0 | 0 |
| Astragalus beckii | 0 | 0 | 1 | 0 | 1 | 1 | 0 | 0 | 0 |
| Astragalus bounophilus | 0 | 0 | 1 | 0 | 0 | 0 | 0 | 1 | 0 |
| Astragalus brachycalyx | 0 | 0 | 0 | 1 | 1 | 1 | 1 | 0 | 0 |
| Astragalus capax | 0 | 0 | 1 | 0 | 0 | 0 | 0 | 0 | 0 |
| Astragalus capito | 0 | 0 | 1 | 0 | 0 | 0 | 0 | 0 | 0 |
| Astragalus carmanicus | 0 | 0 | 0 | 1 | 0 | 0 | 0 | 0 | 0 |
| Astragalus cataonicus | 1 | 0 | 0 | 0 | 0 | 0 | 0 | 0 | 0 |
| Astragalus chartostegius | 0 | 0 | 0 | 1 | 0 | 0 | 0 | 0 | 0 |
| Astragalus chionobiiformis | 0 | 0 | 0 | 0 | 0 | 0 | 1 | 0 | 0 |
| Astragalus chrysanthus | 0 | 0 | 1 | 0 | 0 | 0 | 0 | 0 | 0 |
| Astragalus ciloensis | 0 | 0 | 0 | 0 | 0 | 1 | 0 | 0 | 0 |
| Astragalus confusus | 0 | 0 | 1 | 0 | 0 | 0 | 0 | 0 | 0 |
| Astragalus czorochensis | 0 | 1 | 0 | 0 | 0 | 0 | 0 | 0 | 1 |
| Astragalus daenaensis | 0 | 0 | 0 | 1 | 0 | 0 | 0 | 0 | 0 |
| Astragalus dasycarpus | 0 | 0 | 0 | 0 | 0 | 1 | 0 | 0 | 0 |
| Astragalus declinatus | 0 | 1 | 0 | 0 | 0 | 1 | 0 | 0 | 1 |
| Astragalus demavendicus | 0 | 0 | 1 | 0 | 0 | 0 | 0 | 0 | 0 |
| Astragalus dieterlei | 0 | 0 | 0 | 1 | 0 | 0 | 0 | 0 | 0 |
| Astragalus dumanii | 1 | 0 | 0 | 0 | 0 | 0 | 0 | 0 | 0 |
| Astragalus dzebrailicus | 0 | 1 | 0 | 0 | 1 | 1 | 0 | 0 | 0 |
| Astragalus eriocalyx | 0 | 0 | 0 | 0 | 1 | 0 | 0 | 0 | 0 |
| Astragalus eriocephalus | 1 | 1 | 0 | 0 | 0 | 1 | 0 | 0 | 0 |
| Astragalus erivanensis | 0 | 1 | 0 | 0 | 0 | 0 | 0 | 0 | 0 |
| Astragalus ermineus | 0 | 0 | 0 | 0 | 0 | 1 | 0 | 0 | 0 |
| Astragalus euoplus | 0 | 1 | 0 | 0 | 0 | 0 | 0 | 0 | 0 |
| Astragalus exspectatus | 0 | 0 | 0 | 1 | 0 | 0 | 0 | 0 | 0 |
| Astragalus fragiferus | 0 | 0 | 0 | 1 | 0 | 0 | 0 | 0 | 0 |
| Astragalus fragrans | 0 | 1 | 1 | 0 | 1 | 1 | 0 | 0 | 1 |
| Astragalus fraxinifolius | 1 | 1 | 0 | 0 | 0 | 1 | 0 | 0 | 0 |
| Astragalus frickii | 0 | 1 | 0 | 0 | 0 | 0 | 0 | 0 | 0 |
| Astragalus gevashensis | 0 | 0 | 0 | 0 | 0 | 1 | 0 | 0 | 0 |
| Astragalus gezeldarensis | 0 | 1 | 0 | 0 | 0 | 0 | 0 | 0 | 0 |
| Astragalus globosus | 1 | 1 | 0 | 0 | 0 | 1 | 0 | 0 | 0 |
| Astragalus griseus | 0 | 0 | 0 | 1 | 0 | 0 | 0 | 0 | 0 |
| Astragalus hareftae | 0 | 0 | 0 | 0 | 1 | 1 | 0 | 0 | 0 |
| Astragalus hausknechtii | 1 | 1 | 0 | 0 | 0 | 0 | 0 | 0 | 0 |
| Astragalus hezarensis | 0 | 0 | 0 | 1 | 0 | 0 | 0 | 0 | 0 |
| Astragalus hirticalyx | 0 | 1 | 0 | 0 | 1 | 1 | 1 | 0 | 0 |
| Astragalus horasanicus | 0 | 1 | 0 | 0 | 0 | 0 | 0 | 0 | 0 |
| Astragalus horridus | 0 | 0 | 0 | 1 | 0 | 0 | 0 | 0 | 0 |
| Astragalus humilis | 0 | 0 | 0 | 0 | 0 | 0 | 0 | 0 | 1 |
| Astragalus hyalolepis | 0 | 1 | 0 | 0 | 0 | 1 | 0 | 0 | 1 |
| Astragalus icmadophilus | 0 | 1 | 1 | 0 | 1 | 1 | 1 | 0 | 0 |
| Astragalus incertus | 0 | 1 | 0 | 0 | 0 | 1 | 0 | 0 | 1 |
| Astragalus inexpectatus | 0 | 0 | 0 | 1 | 0 | 0 | 0 | 0 | 0 |
| Astragalus issatissensis | 0 | 0 | 0 | 1 | 0 | 0 | 0 | 0 | 0 |
| Astragalus jodotropis | 0 | 0 | 1 | 0 | 0 | 0 | 0 | 0 | 0 |
| Astragalus johannis | 0 | 0 | 0 | 1 | 0 | 0 | 0 | 0 | 0 |
| Astragalus karabaghensis | 0 | 1 | 1 | 0 | 1 | 0 | 0 | 0 | 0 |
| Astragalus lalesarensis | 0 | 0 | 0 | 1 | 0 | 0 | 0 | 0 | 0 |
| Astragalus lanatus | 1 | 1 | 0 | 0 | 0 | 0 | 0 | 0 | 0 |
| Astragalus laricus | 0 | 0 | 1 | 0 | 0 | 0 | 0 | 0 | 0 |
| Astragalus latistipulatus | 0 | 0 | 0 | 0 | 0 | 1 | 0 | 0 | 0 |
| Astragalus latus | 0 | 0 | 0 | 0 | 0 | 1 | 0 | 0 | 0 |
| Astragalus leiophyllus | 0 | 0 | 0 | 0 | 1 | 1 | 1 | 0 | 0 |
| Astragalus lineatus | 1 | 1 | 1 | 0 | 1 | 1 | 1 | 1 | 0 |
| Astragalus lycioides | 0 | 0 | 0 | 1 | 0 | 0 | 0 | 0 | 0 |
| Astragalus macrosemius | 0 | 0 | 1 | 0 | 0 | 0 | 0 | 0 | 0 |
| Astragalus macrourus | 0 | 1 | 0 | 1 | 1 | 1 | 0 | 0 | 0 |
| Astragalus mahneshanensis | 0 | 0 | 0 | 0 | 0 | 0 | 0 | 1 | 0 |
| Astragalus melanocalyx | 0 | 0 | 0 | 1 | 0 | 0 | 0 | 0 | 0 |
| Astragalus melanocarpus | 1 | 1 | 0 | 0 | 0 | 0 | 0 | 0 | 0 |
| Astragalus melanodon | 0 | 0 | 0 | 1 | 0 | 0 | 0 | 0 | 0 |
| Astragalus modestus | 0 | 0 | 1 | 0 | 0 | 0 | 0 | 0 | 0 |
| Astragalus monanthemus | 0 | 0 | 1 | 0 | 0 | 0 | 0 | 0 | 0 |
| Astragalus montis-alamkuhi | 0 | 0 | 1 | 0 | 0 | 0 | 0 | 0 | 0 |
| Astragalus montis-parrowii | 0 | 0 | 0 | 1 | 0 | 0 | 0 | 0 | 0 |
| Astragalus montis-varvashti | 0 | 0 | 1 | 0 | 0 | 0 | 0 | 0 | 0 |
| Astragalus murinus | 0 | 0 | 0 | 1 | 0 | 0 | 0 | 0 | 0 |
| Astragalus nezaketiae | 0 | 1 | 0 | 0 | 0 | 0 | 0 | 0 | 0 |
| Astragalus nezva-montis | 0 | 0 | 1 | 0 | 0 | 0 | 0 | 0 | 0 |
| Astragalus nigropedunculatus | 0 | 0 | 0 | 0 | 0 | 1 | 0 | 0 | 0 |
| Astragalus ochrochlorus | 0 | 0 | 1 | 1 | 0 | 1 | 0 | 0 | 0 |
| Astragalus ovigerus | 0 | 0 | 0 | 1 | 0 | 0 | 0 | 0 | 0 |
| Astragalus pascuicola | 0 | 0 | 0 | 0 | 0 | 1 | 0 | 0 | 0 |
| Astragalus patrius | 0 | 0 | 1 | 1 | 1 | 0 | 0 | 1 | 0 |
| Astragalus pauperiflorus | 0 | 0 | 0 | 0 | 1 | 0 | 0 | 0 | 0 |
| Astragalus pelliger | 1 | 1 | 0 | 0 | 0 | 0 | 0 | 0 | 0 |
| Astragalus pennatus | 1 | 0 | 0 | 0 | 0 | 1 | 0 | 0 | 0 |
| Astragalus perdurans | 0 | 0 | 1 | 0 | 0 | 0 | 0 | 0 | 0 |
| Astragalus perrarus | 0 | 1 | 0 | 0 | 0 | 1 | 0 | 0 | 0 |
| Astragalus pinetorum | 1 | 1 | 1 | 1 | 1 | 1 | 1 | 0 | 1 |
| Astragalus plagiophacos | 0 | 0 | 1 | 0 | 0 | 0 | 0 | 0 | 0 |
| Astragalus platysematus | 0 | 0 | 1 | 0 | 0 | 0 | 0 | 0 | 0 |
| Astragalus pluriflorus | 0 | 0 | 0 | 0 | 0 | 0 | 0 | 1 | 0 |
| Astragalus podosphaerus | 0 | 0 | 0 | 1 | 0 | 0 | 0 | 0 | 0 |
| Astragalus polyanthus | 0 | 1 | 0 | 0 | 1 | 1 | 0 | 0 | 0 |
| Astragalus polygala | 0 | 1 | 0 | 0 | 0 | 0 | 0 | 0 | 0 |
| Astragalus pseudofragrans | 0 | 0 | 0 | 0 | 0 | 0 | 1 | 0 | 0 |
| Astragalus pseudopinetorum | 1 | 0 | 0 | 0 | 0 | 0 | 0 | 0 | 0 |
| Astragalus pseudoshebarensis | 0 | 0 | 0 | 1 | 0 | 0 | 0 | 0 | 0 |
| Astragalus rechingeri | 0 | 0 | 0 | 0 | 0 | 1 | 0 | 0 | 0 |
| Astragalus remotiflorus | 0 | 0 | 0 | 1 | 0 | 0 | 0 | 0 | 0 |
| Astragalus robertianus | 0 | 0 | 0 | 0 | 0 | 0 | 0 | 0 | 1 |
| Astragalus rubriflorus | 0 | 0 | 1 | 0 | 0 | 0 | 0 | 0 | 0 |
| Astragalus rubrolineatus | 0 | 0 | 1 | 0 | 0 | 0 | 0 | 0 | 0 |
| Astragalus rudimentus | 0 | 0 | 0 | 0 | 1 | 0 | 0 | 0 | 0 |
| Astragalus sachanewii | 0 | 0 | 0 | 0 | 0 | 1 | 0 | 0 | 0 |
| Astragalus sahendi | 0 | 0 | 0 | 0 | 1 | 0 | 0 | 0 | 0 |
| Astragalus savellanicus | 0 | 0 | 0 | 0 | 1 | 0 | 0 | 0 | 0 |
| Astragalus shahsavaranicus | 0 | 0 | 0 | 1 | 0 | 0 | 0 | 0 | 0 |
| Astragalus sphaeranthus | 0 | 0 | 0 | 1 | 0 | 1 | 1 | 0 | 0 |
| Astragalus stenosemioides | 1 | 0 | 0 | 0 | 0 | 1 | 0 | 0 | 0 |
| Astragalus stenostegius | 0 | 0 | 0 | 1 | 0 | 0 | 0 | 0 | 0 |
| Astragalus stridii | 1 | 0 | 0 | 0 | 0 | 0 | 0 | 0 | 0 |
| Astragalus subhanensis | 0 | 0 | 0 | 0 | 0 | 1 | 0 | 0 | 0 |
| Astragalus subsecundus | 0 | 0 | 1 | 1 | 1 | 1 | 0 | 0 | 0 |
| Astragalus taleshensis | 0 | 0 | 1 | 0 | 0 | 0 | 0 | 0 | 0 |
| Astragalus tauricolus | 1 | 1 | 0 | 0 | 0 | 1 | 0 | 0 | 0 |
| Astragalus tenuiscapus | 0 | 0 | 0 | 1 | 0 | 0 | 0 | 0 | 0 |
| Astragalus trabzonicus | 0 | 1 | 0 | 0 | 0 | 0 | 0 | 0 | 0 |
| Astragalus turgidus | 0 | 0 | 0 | 1 | 0 | 0 | 0 | 0 | 0 |
| Astragalus uraniolimneus | 0 | 1 | 0 | 0 | 1 | 1 | 0 | 0 | 0 |
| Astragalus vavilovii | 0 | 1 | 0 | 0 | 0 | 0 | 0 | 0 | 0 |
| Astragalus xerophilus | 1 | 1 | 0 | 0 | 1 | 1 | 0 | 0 | 1 |
| Astragalus zagrosicus | 0 | 0 | 0 | 1 | 0 | 0 | 0 | 0 | 0 |
| Astragalus zerdanus | 0 | 0 | 0 | 1 | 0 | 0 | 0 | 0 | 0 |
| Astragalus zohrabi | 0 | 1 | 0 | 0 | 1 | 1 | 0 | 0 | 0 |
| Asyneuma ekimianum | 1 | 0 | 0 | 0 | 0 | 0 | 0 | 0 | 0 |
| Asyneuma filipes | 0 | 1 | 0 | 0 | 0 | 1 | 0 | 0 | 0 |
| Asyneuma multicaule | 0 | 0 | 0 | 1 | 0 | 0 | 1 | 0 | 0 |
| Asyneuma persicum | 0 | 0 | 0 | 1 | 0 | 1 | 1 | 0 | 0 |
| Asyneuma pulchellum | 0 | 0 | 0 | 0 | 0 | 1 | 0 | 0 | 0 |
| Asyneuma rigidum | 0 | 1 | 0 | 0 | 0 | 0 | 0 | 0 | 0 |
| Athyrium distentifolium | 0 | 1 | 0 | 0 | 0 | 0 | 0 | 0 | 0 |
| Aubrieta anamasica | 1 | 0 | 0 | 0 | 0 | 0 | 0 | 0 | 0 |
| Aurinia rupestris | 1 | 1 | 0 | 0 | 0 | 0 | 0 | 0 | 0 |
| Barbamine procumbens | 0 | 1 | 0 | 0 | 0 | 0 | 0 | 0 | 0 |
| Barbarea minor | 1 | 1 | 0 | 1 | 0 | 1 | 0 | 0 | 0 |
| Bellardiochloa argaea | 1 | 0 | 0 | 0 | 0 | 0 | 0 | 0 | 0 |
| Bellardiochloa polychroa | 0 | 1 | 0 | 0 | 0 | 1 | 0 | 0 | 0 |
| Bellevalia paradoxa | 0 | 1 | 1 | 1 | 1 | 1 | 1 | 1 | 0 |
| Bellevalia rixii | 0 | 0 | 0 | 0 | 0 | 1 | 0 | 0 | 0 |
| Bellevalia tristis | 0 | 0 | 0 | 1 | 0 | 0 | 0 | 0 | 0 |
| Betonica nivea | 0 | 0 | 1 | 0 | 0 | 0 | 0 | 0 | 0 |
| Bornmuellera cappadocica | 0 | 0 | 0 | 0 | 0 | 1 | 0 | 0 | 0 |
| Bromus armenus | 0 | 1 | 0 | 0 | 0 | 0 | 0 | 0 | 0 |
| Bromus cappadocicus | 1 | 0 | 0 | 0 | 0 | 1 | 0 | 0 | 0 |
| Bromus confinis | 0 | 0 | 1 | 0 | 0 | 0 | 0 | 0 | 0 |
| Bromus frigidus | 0 | 0 | 1 | 1 | 0 | 0 | 0 | 0 | 0 |
| Bufonia koelzii | 0 | 0 | 1 | 0 | 0 | 0 | 0 | 0 | 0 |
| Bufonia kotschyana | 0 | 0 | 1 | 1 | 1 | 1 | 0 | 1 | 0 |
| Bufonia micrantha | 0 | 0 | 0 | 1 | 0 | 0 | 0 | 0 | 0 |
| Bufonia stapfii | 0 | 0 | 0 | 1 | 0 | 0 | 0 | 0 | 0 |
| Bunium brachyactis | 1 | 0 | 0 | 0 | 0 | 1 | 0 | 0 | 0 |
| Bupleurum falcatum | 1 | 1 | 0 | 0 | 0 | 1 | 0 | 0 | 0 |
| Calamagrostis parsana | 0 | 0 | 1 | 0 | 0 | 1 | 0 | 0 | 0 |
| Calamintha caroli-henricana | 0 | 0 | 0 | 0 | 0 | 1 | 0 | 0 | 1 |
| Campanula armena | 0 | 1 | 0 | 0 | 0 | 0 | 0 | 0 | 1 |
| Campanula bayerniana | 0 | 1 | 0 | 0 | 1 | 0 | 0 | 0 | 0 |
| Campanula bornmuelleri | 0 | 0 | 0 | 0 | 0 | 1 | 0 | 0 | 0 |
| Campanula choruhensis | 0 | 1 | 0 | 0 | 0 | 0 | 0 | 0 | 0 |
| Campanula collina | 0 | 1 | 0 | 0 | 0 | 0 | 0 | 0 | 0 |
| Campanula conferta | 0 | 1 | 0 | 0 | 0 | 1 | 0 | 0 | 0 |
| Campanula gilliatii | 0 | 0 | 0 | 0 | 1 | 0 | 0 | 0 | 0 |
| Campanula hedgei | 0 | 1 | 0 | 0 | 0 | 0 | 0 | 0 | 0 |
| Campanula hermannii | 0 | 0 | 0 | 1 | 0 | 0 | 0 | 0 | 0 |
| Campanula humillima | 0 | 0 | 0 | 1 | 0 | 0 | 0 | 0 | 0 |
| Campanula karakuschensis | 0 | 1 | 0 | 0 | 1 | 1 | 0 | 0 | 0 |
| Campanula lourica | 0 | 0 | 1 | 0 | 0 | 0 | 0 | 0 | 0 |
| Campanula luristanica | 0 | 0 | 0 | 1 | 0 | 0 | 0 | 0 | 0 |
| Campanula saxifraga | 0 | 1 | 0 | 0 | 0 | 1 | 0 | 0 | 1 |
| Campanula stevenii | 1 | 1 | 1 | 0 | 1 | 1 | 0 | 0 | 1 |
| Campanula telephioides | 1 | 0 | 0 | 0 | 0 | 0 | 0 | 0 | 0 |
| Campanula zangezura | 0 | 1 | 0 | 0 | 0 | 0 | 0 | 0 | 0 |
| Carduus lanuginosus | 1 | 1 | 0 | 0 | 0 | 0 | 0 | 0 | 0 |
| Carex capitellata | 0 | 1 | 0 | 0 | 0 | 0 | 0 | 0 | 0 |
| Carex medwedewii | 0 | 1 | 0 | 0 | 0 | 1 | 1 | 0 | 0 |
| Carex melanorrhyncha | 0 | 1 | 0 | 0 | 0 | 0 | 0 | 0 | 0 |
| Carex oreophila | 1 | 1 | 1 | 1 | 1 | 1 | 0 | 0 | 0 |
| Carex ornithopoda | 0 | 1 | 0 | 0 | 0 | 0 | 0 | 0 | 0 |
| Carex tomentosa | 0 | 1 | 0 | 0 | 0 | 0 | 0 | 0 | 0 |
| Carex tristis | 1 | 1 | 0 | 0 | 0 | 1 | 0 | 0 | 0 |
| Carum caucasicum | 0 | 1 | 0 | 0 | 1 | 1 | 0 | 0 | 0 |
| Carum komarovii | 0 | 1 | 0 | 0 | 0 | 0 | 0 | 0 | 0 |
| Carum rupicola | 1 | 0 | 0 | 0 | 0 | 0 | 0 | 0 | 0 |
| Catabrosa aquatica | 0 | 1 | 1 | 0 | 0 | 1 | 0 | 0 | 0 |
| Centaurea appendicigera | 0 | 1 | 0 | 0 | 0 | 0 | 0 | 0 | 0 |
| Centaurea armena | 0 | 1 | 0 | 0 | 0 | 0 | 0 | 0 | 0 |
| Centaurea congesta | 0 | 0 | 1 | 0 | 1 | 0 | 0 | 0 | 0 |
| Centaurea drabifolia | 1 | 1 | 0 | 0 | 0 | 0 | 0 | 0 | 0 |
| Centaurea elbrusensis | 0 | 1 | 1 | 0 | 1 | 0 | 0 | 0 | 0 |
| Centaurea incanescens | 0 | 0 | 0 | 0 | 1 | 0 | 1 | 0 | 0 |
| Centaurea karduchorum | 0 | 0 | 0 | 0 | 0 | 1 | 0 | 0 | 0 |
| Centaurea lanigera | 1 | 1 | 0 | 0 | 0 | 0 | 0 | 0 | 0 |
| Centaurea mucronifera | 1 | 1 | 0 | 0 | 0 | 0 | 0 | 0 | 0 |
| Centaurea nigrofimbria | 0 | 1 | 0 | 0 | 0 | 0 | 0 | 0 | 0 |
| Centaurea pichleri | 1 | 0 | 0 | 0 | 0 | 0 | 0 | 0 | 0 |
| Centaurea poluninii | 0 | 0 | 0 | 0 | 0 | 1 | 0 | 0 | 0 |
| Centaurea pulcherrima | 0 | 1 | 0 | 0 | 0 | 1 | 0 | 0 | 0 |
| Centaurea rhizantha | 0 | 1 | 1 | 0 | 1 | 1 | 1 | 0 | 1 |
| Centaurea schelkovnikovii | 0 | 1 | 0 | 0 | 0 | 0 | 0 | 0 | 0 |
| Centaurea sieheana | 1 | 0 | 0 | 0 | 0 | 0 | 0 | 0 | 0 |
| Cephalaria cilodaghensis | 0 | 0 | 0 | 0 | 0 | 1 | 0 | 0 | 0 |
| Cephalaria kleinii | 0 | 0 | 1 | 0 | 0 | 0 | 0 | 0 | 0 |
| Cephalaria microcephala | 0 | 0 | 1 | 1 | 1 | 1 | 1 | 1 | 0 |
| Cephalaria sparsipilosa | 0 | 0 | 0 | 0 | 0 | 1 | 0 | 0 | 1 |
| Cerastium araraticum | 0 | 1 | 0 | 0 | 0 | 1 | 0 | 0 | 0 |
| Cerastium gnaphalodes | 1 | 1 | 0 | 0 | 0 | 1 | 0 | 0 | 0 |
| Cerastium lazicum | 0 | 1 | 0 | 0 | 0 | 0 | 0 | 0 | 0 |
| Cerastium persicum | 0 | 0 | 1 | 1 | 0 | 0 | 0 | 0 | 0 |
| Cerastium pseudokasbek | 0 | 1 | 0 | 0 | 0 | 0 | 0 | 0 | 0 |
| Cerastium purpurascens | 1 | 1 | 1 | 0 | 1 | 1 | 0 | 0 | 0 |
| Cerastium szowitsii | 0 | 1 | 0 | 0 | 0 | 0 | 0 | 0 | 0 |
| Cerasus brachypetala | 0 | 0 | 0 | 1 | 0 | 1 | 1 | 0 | 0 |
| Chaenorhinum grossecostatum | 0 | 0 | 0 | 1 | 0 | 0 | 0 | 0 | 0 |
| Chaerophyllum hakkiaricum | 0 | 0 | 0 | 0 | 0 | 1 | 0 | 0 | 0 |
| Chaerophyllum khorossanicum | 0 | 0 | 1 | 0 | 0 | 0 | 0 | 0 | 0 |
| Chaerophyllum macrospermum | 1 | 1 | 1 | 1 | 1 | 1 | 1 | 0 | 0 |
| Chaerophyllum nivale | 0 | 0 | 0 | 1 | 0 | 0 | 0 | 0 | 0 |
| Chamaegeron asterellus | 0 | 0 | 0 | 1 | 0 | 0 | 0 | 0 | 0 |
| Chamaesciadium acaule | 0 | 1 | 0 | 0 | 1 | 1 | 0 | 0 | 1 |
| Cicer anatolicum | 1 | 1 | 0 | 1 | 0 | 1 | 0 | 1 | 0 |
| Cicer incisum | 1 | 1 | 0 | 0 | 0 | 0 | 0 | 0 | 0 |
| Cicer stapfianum | 0 | 0 | 0 | 1 | 0 | 0 | 0 | 0 | 0 |
| Cicer tragacanthoides | 0 | 0 | 1 | 1 | 0 | 0 | 0 | 0 | 0 |
| Cicerbita adenophora | 0 | 0 | 0 | 0 | 0 | 1 | 0 | 0 | 0 |
| Cirsium ellenbergii | 1 | 0 | 0 | 0 | 0 | 0 | 0 | 0 | 0 |
| Cirsium kosmelii | 0 | 1 | 0 | 0 | 0 | 0 | 0 | 0 | 0 |
| Cirsium lappaceum | 0 | 1 | 1 | 1 | 1 | 1 | 0 | 0 | 0 |
| Cirsium pseudobracteosum | 0 | 0 | 0 | 0 | 0 | 1 | 0 | 0 | 0 |
| Cirsium rhizocephalum | 1 | 1 | 1 | 1 | 1 | 1 | 0 | 0 | 0 |
| Cirsium simplex | 0 | 1 | 0 | 0 | 0 | 1 | 0 | 0 | 0 |
| Cirsium tomentosum | 0 | 1 | 0 | 0 | 0 | 1 | 0 | 0 | 0 |
| Clastopus erubescens | 0 | 0 | 0 | 1 | 0 | 0 | 1 | 0 | 0 |
| Clastopus vestitus | 0 | 0 | 1 | 1 | 0 | 0 | 0 | 1 | 0 |
| Cochlearia aucheri | 0 | 1 | 0 | 0 | 0 | 0 | 0 | 0 | 1 |
| Cochlearia sintenisii | 0 | 1 | 0 | 0 | 0 | 0 | 0 | 0 | 0 |
| Colchicum kurdicum | 0 | 0 | 0 | 0 | 0 | 1 | 0 | 0 | 0 |
| Colpodium araraticum | 0 | 1 | 0 | 0 | 0 | 1 | 0 | 0 | 0 |
| Colpodium fibrosum | 0 | 1 | 0 | 0 | 0 | 1 | 0 | 0 | 0 |
| Colpodium gillettii | 0 | 0 | 0 | 0 | 0 | 1 | 1 | 0 | 0 |
| Colpodium parviflorum | 0 | 1 | 1 | 1 | 0 | 1 | 0 | 0 | 1 |
| Colpodium versicolor | 0 | 1 | 1 | 0 | 1 | 1 | 0 | 0 | 0 |
| Colpodium violaceum | 0 | 0 | 0 | 1 | 0 | 0 | 1 | 0 | 0 |
| Coluteocarpus vesicaria | 0 | 1 | 0 | 1 | 0 | 1 | 0 | 0 | 0 |
| Cortusa matthioli | 0 | 0 | 1 | 0 | 0 | 0 | 0 | 0 | 0 |
| Corydalis persica | 0 | 1 | 0 | 0 | 1 | 0 | 0 | 0 | 0 |
| Cotoneaster persicus | 0 | 0 | 0 | 1 | 0 | 0 | 0 | 0 | 0 |
| Cotoneaster zangezuricus | 0 | 1 | 0 | 0 | 0 | 0 | 0 | 0 | 0 |
| Cousinia adenosticta | 0 | 0 | 1 | 0 | 0 | 0 | 0 | 0 | 0 |
| Cousinia archibaldii | 0 | 0 | 0 | 1 | 0 | 0 | 0 | 0 | 0 |
| Cousinia bachtiarica | 0 | 0 | 1 | 1 | 0 | 0 | 0 | 0 | 0 |
| Cousinia bornmuelleri | 0 | 0 | 0 | 1 | 0 | 0 | 0 | 0 | 0 |
| Cousinia concinna | 0 | 0 | 0 | 1 | 0 | 0 | 0 | 0 | 0 |
| Cousinia crispa | 0 | 0 | 1 | 0 | 0 | 0 | 0 | 0 | 0 |
| Cousinia decumbens | 0 | 0 | 1 | 0 | 0 | 0 | 0 | 0 | 0 |
| Cousinia eburnea | 0 | 0 | 0 | 1 | 0 | 0 | 0 | 0 | 0 |
| Cousinia elwendensis | 0 | 0 | 0 | 1 | 0 | 0 | 0 | 0 | 0 |
| Cousinia fragilis | 0 | 0 | 0 | 1 | 0 | 0 | 0 | 0 | 0 |
| Cousinia gmelini | 0 | 0 | 1 | 0 | 0 | 0 | 0 | 0 | 0 |
| Cousinia harazensis | 0 | 0 | 1 | 0 | 0 | 0 | 0 | 0 | 0 |
| Cousinia irritans | 0 | 0 | 1 | 0 | 0 | 0 | 0 | 0 | 0 |
| Cousinia karkasensis | 0 | 0 | 0 | 1 | 0 | 0 | 0 | 0 | 0 |
| Cousinia longifolia | 0 | 0 | 0 | 1 | 0 | 0 | 0 | 0 | 0 |
| Cousinia ottonis | 0 | 0 | 0 | 1 | 0 | 0 | 0 | 0 | 0 |
| Cousinia pterocaulos | 0 | 0 | 1 | 0 | 1 | 0 | 0 | 0 | 0 |
| Cousinia satdagensis | 0 | 0 | 0 | 0 | 0 | 1 | 0 | 0 | 0 |
| Cousinia shahvarica | 0 | 0 | 1 | 0 | 0 | 0 | 0 | 0 | 0 |
| Cousinia sicigera | 0 | 0 | 0 | 1 | 0 | 0 | 0 | 0 | 0 |
| Cousinia xiphiolepis | 0 | 0 | 1 | 0 | 0 | 0 | 0 | 0 | 0 |
| Crepis armena | 1 | 1 | 0 | 0 | 0 | 1 | 0 | 0 | 0 |
| Crepis asadbarensis | 0 | 0 | 1 | 0 | 0 | 0 | 0 | 0 | 0 |
| Crepis bupleurifolia | 0 | 1 | 0 | 0 | 0 | 1 | 0 | 0 | 0 |
| Crepis conyzifolia | 0 | 1 | 0 | 0 | 0 | 0 | 0 | 0 | 0 |
| Crepis demavendi | 0 | 0 | 1 | 0 | 0 | 0 | 0 | 0 | 0 |
| Crepis dioritica | 0 | 1 | 0 | 0 | 0 | 1 | 0 | 0 | 0 |
| Crepis elbursensis | 0 | 0 | 1 | 0 | 1 | 0 | 1 | 0 | 0 |
| Crepis frigida | 1 | 0 | 1 | 0 | 0 | 1 | 0 | 0 | 1 |
| Crepis heterotricha | 0 | 0 | 1 | 1 | 0 | 0 | 0 | 0 | 0 |
| Crepis sahendi | 0 | 1 | 0 | 0 | 1 | 1 | 1 | 0 | 0 |
| Crepis willdenowii | 1 | 1 | 0 | 0 | 0 | 1 | 0 | 0 | 0 |
| Crocus abracteolus | 1 | 0 | 0 | 0 | 0 | 0 | 0 | 0 | 0 |
| Crocus kotschyanus | 0 | 1 | 0 | 0 | 0 | 1 | 0 | 0 | 0 |
| Cyclotrichium straussii | 0 | 0 | 0 | 1 | 0 | 0 | 0 | 0 | 0 |
| Cymbocarpum erythraeum | 0 | 1 | 0 | 0 | 0 | 0 | 0 | 0 | 1 |
| Daphne magakjanii | 0 | 0 | 0 | 0 | 1 | 1 | 0 | 0 | 1 |
| Daphne oleoides | 1 | 1 | 0 | 0 | 1 | 1 | 0 | 0 | 0 |
| Delphinium carduchorum | 0 | 0 | 0 | 0 | 1 | 1 | 0 | 0 | 0 |
| Delphinium elbursense | 0 | 0 | 1 | 0 | 1 | 0 | 0 | 0 | 0 |
| Delphinium foetidum | 0 | 1 | 0 | 0 | 0 | 0 | 0 | 0 | 0 |
| Delphinium lalesaricum | 0 | 0 | 0 | 1 | 0 | 0 | 0 | 0 | 0 |
| Delphinium lanigerum | 0 | 0 | 1 | 1 | 0 | 0 | 0 | 0 | 0 |
| Delphinium linearilobum | 0 | 1 | 0 | 0 | 0 | 1 | 0 | 0 | 0 |
| Dianthus balansae | 1 | 1 | 0 | 0 | 0 | 0 | 0 | 0 | 0 |
| Dianthus brevicaulis | 1 | 0 | 0 | 0 | 0 | 0 | 0 | 0 | 0 |
| Dianthus cretaceus | 0 | 1 | 1 | 1 | 1 | 1 | 1 | 0 | 0 |
| Dianthus denaicus | 0 | 0 | 0 | 1 | 0 | 0 | 0 | 0 | 0 |
| Dianthus diversifolius | 0 | 0 | 0 | 1 | 0 | 0 | 0 | 0 | 0 |
| Dianthus elymaiticus | 0 | 0 | 0 | 1 | 0 | 0 | 0 | 0 | 0 |
| Dianthus erythrocoleus | 0 | 1 | 1 | 0 | 1 | 1 | 0 | 0 | 0 |
| Dianthus goerkii | 1 | 0 | 0 | 0 | 0 | 0 | 0 | 0 | 0 |
| Dianthus lactiflorus | 1 | 0 | 0 | 0 | 0 | 0 | 0 | 0 | 0 |
| Dianthus libanotis | 0 | 0 | 1 | 1 | 1 | 1 | 1 | 0 | 0 |
| Dianthus micranthus | 1 | 1 | 0 | 0 | 0 | 0 | 0 | 0 | 0 |
| Dianthus multicaulis | 0 | 1 | 0 | 0 | 0 | 0 | 0 | 0 | 1 |
| Dianthus muschianus | 0 | 0 | 0 | 0 | 0 | 1 | 0 | 0 | 0 |
| Dianthus raddeanus | 0 | 1 | 0 | 0 | 0 | 0 | 0 | 0 | 0 |
| Dianthus recognitus | 1 | 1 | 0 | 0 | 0 | 0 | 0 | 0 | 0 |
| Dianthus sahandicus | 0 | 0 | 0 | 1 | 0 | 0 | 0 | 0 | 0 |
| Dianthus seidlitzii | 0 | 0 | 0 | 0 | 1 | 0 | 0 | 0 | 0 |
| Dianthus vanensis | 0 | 0 | 0 | 0 | 0 | 1 | 0 | 0 | 0 |
| Dichodon alborzensis | 0 | 0 | 1 | 0 | 0 | 0 | 0 | 0 | 0 |
| Didymophysa aucheri | 0 | 1 | 1 | 1 | 1 | 1 | 1 | 0 | 0 |
| Dielsiocharis kotschyi | 0 | 0 | 1 | 1 | 0 | 0 | 0 | 0 | 0 |
| Dionysia archibaldii | 0 | 0 | 0 | 1 | 0 | 0 | 0 | 0 | 0 |
| Dionysia aubrietioides | 0 | 0 | 0 | 1 | 0 | 0 | 0 | 0 | 0 |
| Dionysia caespitosa | 0 | 0 | 0 | 1 | 0 | 0 | 0 | 0 | 0 |
| Dionysia cristagalli | 0 | 0 | 0 | 1 | 0 | 0 | 0 | 0 | 0 |
| Dionysia curviflora | 0 | 0 | 0 | 1 | 0 | 0 | 0 | 0 | 0 |
| Dionysia esfandiarii | 0 | 0 | 0 | 1 | 0 | 0 | 0 | 0 | 0 |
| Dionysia iranshahrii | 0 | 0 | 0 | 1 | 0 | 0 | 0 | 0 | 0 |
| Dionysia khatamii | 0 | 0 | 0 | 1 | 0 | 0 | 0 | 0 | 0 |
| Dionysia khuzistanica | 0 | 0 | 0 | 1 | 0 | 0 | 0 | 0 | 0 |
| Dionysia leucotricha | 0 | 0 | 0 | 1 | 0 | 0 | 0 | 0 | 0 |
| Dionysia oreodoxa | 0 | 0 | 0 | 1 | 0 | 0 | 0 | 0 | 0 |
| Dionysia revoluta | 0 | 0 | 0 | 1 | 0 | 0 | 0 | 0 | 0 |
| Dionysia rhaptodes | 0 | 0 | 0 | 1 | 0 | 0 | 0 | 0 | 0 |
| Dionysia termeana | 0 | 0 | 0 | 1 | 0 | 0 | 0 | 0 | 0 |
| Dionysia zagrica | 0 | 0 | 0 | 1 | 0 | 0 | 0 | 0 | 0 |
| Dionysia zetterlundii | 0 | 0 | 0 | 1 | 0 | 0 | 0 | 0 | 0 |
| Dionysia zschummelii | 0 | 0 | 0 | 1 | 0 | 0 | 0 | 0 | 0 |
| Diplotaenia cachrydifolia | 0 | 0 | 1 | 0 | 0 | 1 | 0 | 0 | 0 |
| Diplotaenia damavandica | 0 | 0 | 1 | 0 | 0 | 0 | 0 | 0 | 0 |
| Dolichorrhiza persica | 0 | 0 | 1 | 0 | 0 | 0 | 0 | 0 | 0 |
| Dorema aucheri | 0 | 0 | 0 | 1 | 0 | 0 | 0 | 0 | 0 |
| Doronicum bracteatum | 0 | 0 | 0 | 1 | 0 | 0 | 1 | 0 | 0 |
| Doronicum dolichotrichum | 0 | 1 | 0 | 0 | 0 | 0 | 0 | 0 | 0 |
| Doronicum hakkiaricum | 0 | 0 | 0 | 0 | 0 | 1 | 0 | 0 | 0 |
| Doronicum maximum | 0 | 1 | 0 | 0 | 0 | 1 | 0 | 0 | 0 |
| Doronicum tobeyi | 0 | 1 | 0 | 0 | 0 | 0 | 0 | 0 | 0 |
| Draba araratica | 0 | 1 | 0 | 0 | 0 | 1 | 0 | 0 | 0 |
| Draba bruniifolia | 1 | 1 | 0 | 0 | 1 | 1 | 1 | 0 | 0 |
| Draba cappadocica | 1 | 0 | 0 | 0 | 0 | 1 | 0 | 0 | 0 |
| Draba orientalis | 0 | 0 | 0 | 0 | 0 | 0 | 0 | 0 | 1 |
| Draba polytricha | 0 | 1 | 0 | 0 | 0 | 0 | 0 | 0 | 1 |
| Draba pulchella | 0 | 0 | 1 | 0 | 0 | 0 | 1 | 0 | 0 |
| Draba thylacocarpa | 0 | 0 | 0 | 0 | 0 | 1 | 0 | 0 | 0 |
| Dracocephalum aucheri | 0 | 0 | 1 | 0 | 1 | 1 | 0 | 0 | 0 |
| Dracocephalum ghahremanii | 0 | 0 | 1 | 0 | 0 | 0 | 0 | 0 | 0 |
| Dracocephalum kotschyi | 0 | 0 | 1 | 1 | 0 | 0 | 0 | 0 | 0 |
| Dracocephalum multicaule | 1 | 1 | 0 | 0 | 1 | 1 | 0 | 0 | 0 |
| Dracocephalum polychaetum | 0 | 0 | 0 | 1 | 0 | 0 | 0 | 0 | 0 |
| Dracocephalum surmandinum | 0 | 0 | 0 | 1 | 0 | 0 | 0 | 0 | 0 |
| Drymocallis damghanensis | 0 | 0 | 1 | 0 | 0 | 0 | 0 | 0 | 0 |
| Echinophora cinerea | 0 | 0 | 0 | 1 | 0 | 0 | 0 | 0 | 0 |
| Elburzia fenestrata | 0 | 0 | 1 | 0 | 0 | 0 | 0 | 0 | 0 |
| Epilobium frigidum | 1 | 1 | 1 | 1 | 1 | 1 | 1 | 0 | 0 |
| Epilobium ponticum | 1 | 1 | 1 | 0 | 0 | 1 | 1 | 0 | 0 |
| Epilobium rechingeri | 0 | 0 | 1 | 0 | 0 | 0 | 1 | 0 | 0 |
| Erigeron caucasicus | 0 | 1 | 1 | 0 | 1 | 1 | 0 | 0 | 0 |
| Erigeron cilicicus | 1 | 0 | 0 | 0 | 0 | 0 | 0 | 0 | 0 |
| Erigeron daenensis | 0 | 0 | 0 | 0 | 0 | 1 | 0 | 0 | 0 |
| Erigeron hyrcanicus | 0 | 0 | 1 | 0 | 0 | 0 | 0 | 0 | 0 |
| Erigeron zederbaueri | 1 | 0 | 0 | 0 | 0 | 0 | 0 | 0 | 0 |
| Eritrichium gracillimum | 0 | 0 | 1 | 0 | 0 | 0 | 0 | 0 | 0 |
| Erodium cedrorum | 1 | 0 | 0 | 0 | 0 | 0 | 0 | 0 | 0 |
| Erodium dimorphum | 0 | 0 | 1 | 0 | 0 | 0 | 0 | 0 | 0 |
| Erodium hakkiaricum | 0 | 0 | 0 | 0 | 0 | 1 | 0 | 0 | 0 |
| Eryngium bornmuelleri | 0 | 0 | 0 | 0 | 0 | 1 | 0 | 0 | 0 |
| Eryngium ilex | 0 | 1 | 0 | 0 | 0 | 0 | 0 | 0 | 0 |
| Erysimum caespitosum | 0 | 0 | 1 | 1 | 1 | 0 | 0 | 0 | 0 |
| Erysimum damirliense | 0 | 0 | 0 | 0 | 0 | 0 | 0 | 1 | 0 |
| Erysimum elbrusense | 0 | 0 | 1 | 1 | 0 | 1 | 1 | 0 | 0 |
| Erysimum frigidum | 0 | 0 | 1 | 1 | 0 | 0 | 0 | 0 | 0 |
| Erysimum gelidum | 0 | 1 | 0 | 0 | 1 | 0 | 1 | 0 | 1 |
| Erysimum guneri | 0 | 0 | 0 | 0 | 0 | 1 | 0 | 0 | 0 |
| Erysimum hakkiaricum | 0 | 0 | 0 | 0 | 0 | 1 | 0 | 0 | 0 |
| Erysimum hezarense | 0 | 0 | 0 | 1 | 0 | 0 | 0 | 0 | 0 |
| Erysimum ikizdereense | 0 | 1 | 0 | 0 | 0 | 0 | 0 | 0 | 0 |
| Erysimum kotschyanum | 1 | 0 | 0 | 0 | 0 | 0 | 0 | 0 | 0 |
| Erysimum macrostigma | 0 | 1 | 1 | 0 | 1 | 1 | 0 | 0 | 0 |
| Erysimum munzuriense | 0 | 1 | 0 | 0 | 0 | 0 | 0 | 0 | 0 |
| Erysimum nasturtioides | 0 | 0 | 0 | 1 | 0 | 0 | 1 | 0 | 0 |
| Erysimum polatschekii | 0 | 0 | 0 | 1 | 0 | 0 | 0 | 0 | 0 |
| Erysimum rizeense | 0 | 1 | 0 | 0 | 0 | 0 | 0 | 0 | 0 |
| Erysimum sintenisianum | 1 | 1 | 0 | 0 | 0 | 1 | 0 | 0 | 1 |
| Erysimum yildirimlii | 0 | 1 | 0 | 0 | 0 | 0 | 0 | 0 | 0 |
| Euphorbia belgheisi | 0 | 0 | 0 | 0 | 0 | 0 | 0 | 1 | 0 |
| Euphorbia erythradenia | 0 | 0 | 0 | 1 | 0 | 0 | 0 | 0 | 0 |
| Euphorbia grisophylla | 0 | 0 | 0 | 0 | 0 | 1 | 0 | 0 | 0 |
| Euphorbia hebecarpa | 0 | 0 | 0 | 1 | 1 | 0 | 1 | 0 | 0 |
| Euphorbia herniariifolia | 1 | 1 | 0 | 0 | 0 | 0 | 0 | 0 | 0 |
| Euphorbia macrocarpa | 0 | 0 | 0 | 1 | 0 | 1 | 0 | 0 | 0 |
| Euphorbia mirzakhaniana | 0 | 0 | 1 | 0 | 0 | 0 | 0 | 0 | 0 |
| Euphorbia plebeia | 0 | 0 | 0 | 1 | 0 | 0 | 0 | 0 | 0 |
| Euphorbia sahendi | 0 | 0 | 0 | 0 | 1 | 0 | 0 | 0 | 0 |
| Euphorbia sanasunitensis | 0 | 0 | 0 | 0 | 0 | 1 | 0 | 0 | 0 |
| Euphrasia amblyodonta | 0 | 1 | 0 | 0 | 0 | 0 | 0 | 0 | 0 |
| Euphrasia juzepczukii | 0 | 1 | 1 | 0 | 1 | 1 | 0 | 0 | 0 |
| Euphrasia sevanensis | 0 | 1 | 0 | 0 | 1 | 1 | 0 | 0 | 0 |
| Ferula haussknechtii | 0 | 0 | 0 | 0 | 0 | 1 | 0 | 0 | 0 |
| Ferula hezarlalehzarica | 0 | 0 | 0 | 1 | 0 | 0 | 0 | 0 | 0 |
| Ferula microcolea | 0 | 0 | 1 | 1 | 1 | 0 | 0 | 0 | 0 |
| Ferula setifolia | 0 | 1 | 0 | 0 | 0 | 1 | 0 | 0 | 0 |
| Ferulago angulata | 0 | 0 | 1 | 1 | 1 | 1 | 1 | 0 | 0 |
| Ferulago contracta | 0 | 0 | 0 | 1 | 0 | 0 | 0 | 0 | 0 |
| Festuca adanensis | 1 | 1 | 0 | 0 | 0 | 0 | 0 | 0 | 0 |
| Festuca anatolica | 1 | 1 | 0 | 0 | 0 | 0 | 0 | 0 | 0 |
| Festuca artvinensis | 0 | 1 | 0 | 0 | 0 | 0 | 0 | 0 | 0 |
| Festuca brunnescens | 0 | 1 | 0 | 0 | 0 | 1 | 0 | 0 | 0 |
| Festuca bushiana | 0 | 1 | 0 | 0 | 0 | 0 | 0 | 0 | 0 |
| Festuca cappadocica | 1 | 0 | 0 | 0 | 0 | 1 | 0 | 0 | 0 |
| Festuca cataonica | 1 | 0 | 0 | 0 | 0 | 0 | 0 | 0 | 0 |
| Festuca chalcophaea | 0 | 1 | 0 | 0 | 0 | 1 | 0 | 0 | 0 |
| Festuca cratericola | 1 | 0 | 0 | 0 | 0 | 0 | 0 | 0 | 0 |
| Festuca elwendiana | 0 | 1 | 0 | 0 | 0 | 1 | 0 | 0 | 0 |
| Festuca iranica | 0 | 0 | 0 | 1 | 0 | 0 | 0 | 0 | 0 |
| Festuca lazistanica | 0 | 1 | 0 | 0 | 0 | 0 | 0 | 0 | 0 |
| Festuca pinifolia | 1 | 1 | 0 | 0 | 0 | 0 | 0 | 0 | 0 |
| Festuca rechingeri | 0 | 0 | 1 | 0 | 0 | 0 | 0 | 0 | 0 |
| Festuca sabalanica | 0 | 0 | 0 | 0 | 1 | 0 | 0 | 0 | 0 |
| Festuca skvortsovii | 0 | 1 | 0 | 0 | 1 | 0 | 0 | 0 | 0 |
| Fibigia multicaulis | 0 | 0 | 1 | 1 | 0 | 0 | 1 | 0 | 0 |
| Fibigia umbellata | 0 | 0 | 1 | 1 | 0 | 0 | 0 | 0 | 0 |
| Fritillaria alburyana | 0 | 1 | 0 | 0 | 0 | 0 | 0 | 0 | 0 |
| Fritillaria aurea | 1 | 1 | 0 | 0 | 0 | 0 | 0 | 0 | 0 |
| Fritillaria caucasica | 0 | 1 | 0 | 0 | 1 | 0 | 0 | 0 | 1 |
| Fritillaria chlorantha | 0 | 0 | 0 | 1 | 0 | 0 | 0 | 0 | 0 |
| Fritillaria crassifolia | 0 | 1 | 0 | 1 | 1 | 1 | 1 | 0 | 0 |
| Fritillaria kotschyana | 0 | 0 | 1 | 0 | 0 | 0 | 0 | 0 | 0 |
| Fritillaria latifolia | 0 | 1 | 0 | 0 | 0 | 0 | 0 | 0 | 0 |
| Fritillaria michailovskyi | 0 | 1 | 0 | 0 | 0 | 0 | 0 | 0 | 0 |
| Fritillaria minima | 0 | 0 | 0 | 0 | 0 | 1 | 0 | 0 | 0 |
| Fritillaria minuta | 0 | 1 | 0 | 0 | 0 | 1 | 0 | 0 | 0 |
| Fritillaria olivieri | 0 | 0 | 0 | 1 | 0 | 0 | 0 | 0 | 0 |
| Fritillaria reuteri | 0 | 0 | 0 | 1 | 0 | 0 | 0 | 0 | 0 |
| Fritillaria zagrica | 0 | 0 | 1 | 1 | 1 | 0 | 0 | 0 | 0 |
| Fuernrohria setifolia | 0 | 1 | 0 | 0 | 0 | 0 | 0 | 0 | 0 |
| Gagea alexeenkoana | 0 | 1 | 1 | 1 | 1 | 0 | 0 | 0 | 0 |
| Gagea caroli-kochii | 0 | 1 | 1 | 0 | 0 | 0 | 0 | 0 | 0 |
| Gagea glacialis | 1 | 1 | 0 | 0 | 0 | 1 | 0 | 0 | 0 |
| Gagea joannis | 0 | 1 | 0 | 0 | 0 | 0 | 0 | 0 | 0 |
| Gagea luteoides | 1 | 1 | 0 | 0 | 0 | 1 | 0 | 0 | 0 |
| Gagea uliginosa | 1 | 0 | 1 | 0 | 1 | 1 | 0 | 0 | 0 |
| Galium aladaghense | 1 | 0 | 0 | 0 | 0 | 0 | 0 | 0 | 0 |
| Galium aucheri | 0 | 0 | 1 | 0 | 0 | 0 | 0 | 0 | 0 |
| Galium boreale | 0 | 1 | 1 | 0 | 1 | 0 | 0 | 0 | 0 |
| Galium decumbens | 0 | 0 | 1 | 0 | 1 | 0 | 0 | 0 | 0 |
| Galium delicatulum | 0 | 0 | 1 | 0 | 0 | 0 | 0 | 0 | 0 |
| Galium hyrcanicum | 0 | 1 | 1 | 0 | 1 | 1 | 0 | 0 | 0 |
| Galium majmechense | 0 | 1 | 0 | 0 | 0 | 0 | 0 | 0 | 0 |
| Galium nabelekii | 0 | 1 | 0 | 0 | 0 | 1 | 0 | 0 | 0 |
| Galium nigdeense | 1 | 0 | 0 | 0 | 0 | 0 | 0 | 0 | 0 |
| Galium ovitdaghense | 0 | 1 | 0 | 0 | 0 | 0 | 0 | 0 | 0 |
| Galium pseudokurdicum | 0 | 0 | 0 | 1 | 0 | 0 | 1 | 0 | 0 |
| Galium schoenbeck-Temesyae | 0 | 0 | 0 | 1 | 0 | 0 | 0 | 0 | 0 |
| Galium subvelutinum | 0 | 0 | 1 | 1 | 0 | 1 | 0 | 1 | 0 |
| Galium tuncelianum | 0 | 1 | 0 | 0 | 0 | 0 | 0 | 0 | 0 |
| Gentiana boissieri | 1 | 0 | 0 | 0 | 0 | 0 | 0 | 0 | 0 |
| Gentiana gelida | 0 | 1 | 0 | 0 | 1 | 1 | 0 | 0 | 0 |
| Gentianella holosteoides | 1 | 0 | 0 | 0 | 0 | 0 | 0 | 0 | 0 |
| Geranium cinereum | 1 | 1 | 0 | 0 | 0 | 0 | 0 | 0 | 0 |
| Geranium kurdicum | 0 | 0 | 0 | 0 | 0 | 1 | 0 | 0 | 0 |
| Geranium persicum | 0 | 0 | 1 | 1 | 1 | 1 | 0 | 0 | 0 |
| Gnaphalium leucopilinum | 1 | 0 | 0 | 0 | 0 | 1 | 0 | 0 | 0 |
| Graellsia isfahan | 0 | 0 | 0 | 1 | 0 | 0 | 0 | 0 | 0 |
| Graellsia stylosa | 0 | 0 | 1 | 0 | 0 | 0 | 0 | 0 | 0 |
| Gypsophila adenophylla | 0 | 0 | 0 | 0 | 0 | 1 | 0 | 0 | 0 |
| Gypsophila aretioides | 0 | 1 | 1 | 0 | 0 | 0 | 0 | 0 | 0 |
| Gypsophila briquetiana | 0 | 1 | 0 | 0 | 0 | 0 | 0 | 0 | 0 |
| Gypsophila graminifolia | 0 | 0 | 0 | 0 | 1 | 0 | 0 | 0 | 0 |
| Gypsophila hakkiarica | 0 | 0 | 0 | 0 | 0 | 1 | 0 | 0 | 0 |
| Gypsophila lipskyi | 0 | 1 | 0 | 0 | 0 | 0 | 0 | 0 | 0 |
| Gypsophila nabelaelekii | 0 | 0 | 0 | 0 | 0 | 1 | 0 | 0 | 1 |
| Gypsophila peshmenii | 0 | 0 | 0 | 0 | 0 | 1 | 0 | 0 | 0 |
| Gypsophila yazdiana | 0 | 0 | 0 | 1 | 0 | 0 | 0 | 0 | 0 |
| Haussknechtia elymaitica | 0 | 0 | 0 | 1 | 0 | 0 | 0 | 0 | 0 |
| Hedysarum erythroleucum | 1 | 1 | 0 | 0 | 0 | 1 | 0 | 0 | 0 |
| Hedysarum persicum | 0 | 0 | 1 | 0 | 0 | 0 | 0 | 0 | 0 |
| Hedysarum vanense | 0 | 0 | 0 | 0 | 1 | 1 | 0 | 0 | 0 |
| Heldreichia bupleurifolia | 1 | 1 | 0 | 0 | 0 | 0 | 0 | 0 | 0 |
| Heldreichia rotundifolia | 1 | 1 | 0 | 0 | 0 | 1 | 0 | 0 | 0 |
| Helichrysum athanaton | 0 | 0 | 0 | 1 | 0 | 0 | 0 | 0 | 0 |
| Helichrysum chionophilum | 1 | 0 | 0 | 0 | 0 | 0 | 0 | 0 | 0 |
| Helichrysum davisianum | 0 | 0 | 0 | 1 | 0 | 0 | 0 | 0 | 0 |
| Helichrysum oligocephalum | 0 | 0 | 1 | 1 | 1 | 0 | 0 | 1 | 0 |
| Helichrysum pallasii | 1 | 1 | 0 | 0 | 0 | 1 | 0 | 0 | 0 |
| Helichrysum psychrophilum | 0 | 0 | 1 | 0 | 1 | 1 | 1 | 1 | 0 |
| Helichrysum yurterianum | 0 | 1 | 0 | 0 | 0 | 0 | 0 | 0 | 0 |
| Helictotrichon argaeum | 1 | 1 | 0 | 0 | 0 | 0 | 0 | 0 | 0 |
| Heracleum anisactis | 0 | 0 | 1 | 0 | 1 | 0 | 0 | 0 | 0 |
| Heracleum crenatifolium | 0 | 1 | 0 | 0 | 0 | 1 | 0 | 0 | 0 |
| Heracleum humile | 1 | 1 | 0 | 0 | 0 | 0 | 0 | 0 | 0 |
| Heracleum pastinacifolium | 0 | 1 | 0 | 0 | 1 | 1 | 0 | 0 | 0 |
| Heracleum rawianum | 0 | 0 | 0 | 0 | 1 | 1 | 1 | 0 | 0 |
| Heracleum schelkovnikovii | 0 | 1 | 0 | 0 | 0 | 0 | 0 | 0 | 0 |
| Heracleum sphondylium | 0 | 1 | 0 | 0 | 0 | 0 | 0 | 0 | 0 |
| Herniaria argaea | 1 | 1 | 0 | 0 | 0 | 0 | 0 | 0 | 0 |
| Herniaria olympica | 1 | 0 | 0 | 0 | 0 | 1 | 0 | 0 | 0 |
| Hesperis borbasii | 0 | 0 | 0 | 1 | 0 | 0 | 0 | 0 | 0 |
| Hesperis leucoclada | 0 | 0 | 0 | 1 | 0 | 0 | 0 | 0 | 0 |
| Hesperis luristanica | 0 | 0 | 0 | 1 | 0 | 0 | 0 | 0 | 0 |
| Hesperis nivalis | 0 | 0 | 0 | 1 | 0 | 0 | 0 | 0 | 0 |
| Hyalopoa hracziana | 0 | 1 | 0 | 0 | 0 | 0 | 0 | 0 | 0 |
| Hymenocrater yazdianus | 0 | 0 | 0 | 1 | 0 | 0 | 0 | 0 | 0 |
| Hyoscyamus kotschyanus | 0 | 0 | 0 | 1 | 0 | 0 | 0 | 0 | 0 |
| Hyoscyamus kurdicus | 0 | 0 | 1 | 0 | 1 | 0 | 1 | 0 | 0 |
| Hypericum armenum | 0 | 1 | 1 | 0 | 1 | 1 | 0 | 0 | 0 |
| Hypericum crenulatum | 1 | 0 | 0 | 0 | 0 | 0 | 0 | 0 | 0 |
| Hypericum musadoganii | 1 | 0 | 0 | 0 | 0 | 0 | 0 | 0 | 0 |
| Inula acaulis | 1 | 1 | 1 | 0 | 0 | 1 | 0 | 0 | 0 |
| Inula mariae | 0 | 1 | 0 | 0 | 0 | 0 | 0 | 0 | 0 |
| Iranecio elbrusensis | 0 | 0 | 1 | 0 | 0 | 0 | 0 | 0 | 0 |
| Iranecio oligolepis | 0 | 0 | 1 | 0 | 0 | 0 | 0 | 0 | 0 |
| Iranecio paucilobus | 0 | 0 | 1 | 1 | 0 | 1 | 1 | 0 | 0 |
| Iris barnumiae | 0 | 0 | 1 | 0 | 1 | 1 | 1 | 0 | 0 |
| Isatis brachycarpa | 0 | 0 | 0 | 0 | 0 | 1 | 0 | 0 | 0 |
| Isatis takhtajanii | 1 | 1 | 0 | 1 | 0 | 1 | 0 | 0 | 0 |
| Jasione supina | 1 | 1 | 0 | 0 | 0 | 0 | 0 | 0 | 0 |
| Johrenia alpina | 1 | 0 | 0 | 0 | 0 | 0 | 0 | 0 | 0 |
| Johreniopsis scoparia | 0 | 0 | 1 | 1 | 0 | 0 | 0 | 0 | 0 |
| Johreniopsis seseloides | 0 | 0 | 1 | 1 | 1 | 0 | 0 | 0 | 0 |
| Juncus filiformis | 0 | 1 | 0 | 0 | 0 | 0 | 0 | 0 | 0 |
| Jurinea meda | 0 | 0 | 0 | 1 | 0 | 0 | 0 | 0 | 0 |
| Jurinea viciosoi | 0 | 0 | 0 | 1 | 0 | 0 | 0 | 0 | 0 |
| Jurinella frigida | 0 | 0 | 1 | 0 | 0 | 0 | 0 | 0 | 0 |
| Jurinella microcephala | 0 | 0 | 1 | 0 | 0 | 0 | 0 | 0 | 0 |
| Jurinella moschus | 1 | 1 | 1 | 0 | 1 | 1 | 1 | 0 | 0 |
| Kelussia odoratissima | 0 | 0 | 0 | 1 | 0 | 0 | 0 | 0 | 0 |
| Lactuca denaensis | 0 | 0 | 0 | 1 | 0 | 0 | 0 | 0 | 0 |
| Lactuca hazaranensis | 0 | 0 | 0 | 1 | 0 | 0 | 0 | 0 | 0 |
| Lagochilus kotschyanus | 0 | 0 | 1 | 0 | 0 | 0 | 1 | 0 | 0 |
| Lallemantia canescens | 0 | 0 | 0 | 0 | 1 | 0 | 0 | 0 | 0 |
| Lamium armenum | 0 | 1 | 0 | 0 | 0 | 0 | 0 | 0 | 0 |
| Lamium crinitum | 0 | 1 | 0 | 0 | 0 | 1 | 0 | 0 | 0 |
| Lamium eriocephalum | 1 | 0 | 0 | 0 | 0 | 0 | 0 | 0 | 0 |
| Lamium tomentosum | 0 | 1 | 1 | 0 | 0 | 1 | 1 | 0 | 0 |
| Laserpitium carduchorum | 0 | 0 | 0 | 0 | 0 | 1 | 0 | 0 | 0 |
| Lathyrus bitlisicus | 0 | 0 | 0 | 0 | 0 | 1 | 0 | 0 | 0 |
| Lathyrus brachypterus | 0 | 1 | 0 | 0 | 0 | 1 | 0 | 0 | 0 |
| Lathyrus cyaneus | 0 | 1 | 0 | 0 | 0 | 0 | 0 | 0 | 0 |
| Lathyrus nivalis | 0 | 1 | 0 | 0 | 0 | 1 | 0 | 0 | 0 |
| Leontodon oxylepis | 1 | 0 | 0 | 0 | 0 | 0 | 0 | 0 | 0 |
| Leontodon stenocalathius | 0 | 0 | 1 | 0 | 0 | 0 | 0 | 0 | 0 |
| Leonurus cardiaca | 0 | 0 | 1 | 0 | 0 | 0 | 0 | 0 | 0 |
| Lepechiniella fursei | 0 | 0 | 1 | 0 | 0 | 0 | 0 | 0 | 0 |
| Lepechiniella persica | 0 | 0 | 1 | 0 | 0 | 0 | 0 | 0 | 0 |
| Lepidium pabotii | 0 | 0 | 0 | 1 | 0 | 0 | 0 | 0 | 0 |
| Leucopoa pseudosclerophylla | 0 | 0 | 0 | 1 | 0 | 0 | 0 | 0 | 0 |
| Leutea cupularis | 0 | 0 | 1 | 1 | 0 | 0 | 0 | 0 | 0 |
| Leutea petiolaris | 0 | 0 | 1 | 1 | 1 | 0 | 0 | 0 | 0 |
| Leutea rechingeri | 0 | 0 | 0 | 0 | 0 | 0 | 1 | 0 | 0 |
| Levisticum officinale | 0 | 0 | 0 | 1 | 0 | 0 | 0 | 0 | 0 |
| Ligularia persica | 0 | 0 | 1 | 0 | 0 | 0 | 0 | 0 | 0 |
| Linaria karajensis | 0 | 0 | 1 | 0 | 0 | 0 | 0 | 0 | 0 |
| Linaria remotiflora | 0 | 0 | 0 | 1 | 0 | 0 | 0 | 0 | 0 |
| Linaria schelkownikowii | 0 | 1 | 0 | 0 | 0 | 0 | 0 | 0 | 0 |
| Linaria shahroudensis | 0 | 0 | 1 | 0 | 0 | 0 | 0 | 0 | 0 |
| Linum densiflorum | 0 | 0 | 0 | 0 | 0 | 1 | 0 | 0 | 0 |
| Linum empetrifolium | 1 | 0 | 0 | 0 | 0 | 0 | 0 | 0 | 0 |
| Linum meletonis | 0 | 0 | 0 | 0 | 0 | 1 | 0 | 0 | 0 |
| Linum obtusatum | 1 | 1 | 0 | 0 | 0 | 0 | 0 | 0 | 0 |
| Linum punctatum | 0 | 0 | 0 | 0 | 1 | 1 | 1 | 0 | 0 |
| Linum subbiflorum | 0 | 1 | 0 | 0 | 0 | 0 | 0 | 0 | 0 |
| Linum triflorum | 0 | 0 | 0 | 0 | 0 | 1 | 0 | 0 | 0 |
| Lophanthus turcicus | 0 | 0 | 0 | 0 | 0 | 1 | 0 | 0 | 0 |
| Luzula luzulina | 0 | 1 | 0 | 0 | 0 | 0 | 0 | 0 | 0 |
| Luzula stenophylla | 1 | 1 | 0 | 0 | 0 | 1 | 0 | 0 | 0 |
| Malabaila dasyantha | 0 | 1 | 0 | 0 | 0 | 0 | 0 | 0 | 0 |
| Marrubium astracanicum | 1 | 1 | 1 | 1 | 1 | 1 | 1 | 0 | 1 |
| Marrubium cordatum | 0 | 0 | 0 | 0 | 0 | 1 | 1 | 0 | 0 |
| Marrubium eriocephalum | 0 | 0 | 0 | 0 | 0 | 0 | 1 | 0 | 0 |
| Marrubium heterodon | 1 | 0 | 0 | 0 | 0 | 0 | 0 | 0 | 0 |
| Mattiastrum pygmaeum | 0 | 0 | 1 | 0 | 0 | 0 | 0 | 0 | 0 |
| Mesostemma kotschyana | 0 | 0 | 1 | 1 | 0 | 1 | 0 | 0 | 0 |
| Micrantha multicaulis | 0 | 0 | 0 | 1 | 0 | 0 | 0 | 0 | 0 |
| Minuartia aucheriana | 0 | 0 | 0 | 1 | 0 | 0 | 0 | 0 | 0 |
| Minuartia dianthifolia | 1 | 1 | 0 | 0 | 0 | 1 | 0 | 0 | 0 |
| Minuartia glandulosa | 0 | 1 | 1 | 1 | 1 | 1 | 1 | 0 | 0 |
| Minuartia hamzaoglui | 1 | 0 | 0 | 0 | 0 | 0 | 0 | 0 | 0 |
| Minuartia lineata | 0 | 1 | 1 | 0 | 1 | 1 | 0 | 0 | 0 |
| Minuartia litwinowii | 0 | 0 | 1 | 0 | 0 | 0 | 0 | 0 | 0 |
| Minuartia oreina | 1 | 1 | 1 | 1 | 1 | 1 | 1 | 0 | 0 |
| Minuartia rimarum | 1 | 1 | 0 | 0 | 0 | 0 | 0 | 0 | 0 |
| Minuartia sabalanica | 0 | 0 | 0 | 0 | 1 | 0 | 0 | 0 | 0 |
| Minuartia sublineata | 0 | 0 | 0 | 1 | 1 | 0 | 1 | 0 | 0 |
| Minuartia umbellulifera | 1 | 1 | 0 | 0 | 0 | 1 | 0 | 0 | 0 |
| Muscari anatolicum | 1 | 0 | 0 | 0 | 0 | 0 | 0 | 0 | 0 |
| Muscari bourgaei | 1 | 0 | 0 | 0 | 0 | 0 | 0 | 0 | 0 |
| Muscari coeleste | 1 | 1 | 0 | 0 | 0 | 0 | 0 | 0 | 0 |
| Myopordon aucheri | 0 | 0 | 0 | 1 | 0 | 0 | 0 | 0 | 0 |
| Myopordon damavandica | 0 | 0 | 1 | 0 | 0 | 0 | 0 | 0 | 0 |
| Myopordon hyrcanum | 0 | 0 | 1 | 0 | 0 | 0 | 0 | 0 | 0 |
| Myopordon persicum | 0 | 0 | 0 | 1 | 0 | 0 | 0 | 0 | 0 |
| Myosotis guneri | 0 | 1 | 0 | 0 | 0 | 0 | 0 | 0 | 0 |
| Myosotis olympica | 1 | 1 | 1 | 1 | 1 | 1 | 0 | 0 | 0 |
| Myosotis platyphylla | 0 | 0 | 0 | 0 | 0 | 1 | 0 | 0 | 0 |
| Nepeta alaghezi | 0 | 1 | 0 | 0 | 0 | 0 | 0 | 0 | 0 |
| Nepeta allotria | 0 | 0 | 1 | 0 | 0 | 0 | 0 | 0 | 0 |
| Nepeta archibaldii | 0 | 0 | 0 | 1 | 0 | 0 | 0 | 0 | 0 |
| Nepeta assurgens | 0 | 0 | 0 | 1 | 0 | 0 | 0 | 0 | 0 |
| Nepeta azadkouhensis | 0 | 0 | 1 | 0 | 0 | 0 | 0 | 0 | 0 |
| Nepeta bornmuelleri | 0 | 0 | 0 | 1 | 0 | 0 | 0 | 0 | 0 |
| Nepeta chionophila | 0 | 0 | 0 | 1 | 0 | 0 | 0 | 0 | 0 |
| Nepeta crispa | 0 | 0 | 1 | 1 | 0 | 0 | 0 | 0 | 0 |
| Nepeta dschuparensis | 0 | 0 | 0 | 1 | 0 | 0 | 0 | 0 | 0 |
| Nepeta elymaitica | 0 | 0 | 0 | 1 | 0 | 0 | 1 | 0 | 0 |
| Nepeta glomerulosa | 0 | 0 | 1 | 1 | 0 | 0 | 0 | 0 | 0 |
| Nepeta iranshahrii | 0 | 0 | 0 | 1 | 0 | 0 | 0 | 0 | 0 |
| Nepeta lamiifolia | 0 | 1 | 0 | 0 | 0 | 1 | 0 | 0 | 0 |
| Nepeta lasiocephala | 0 | 0 | 0 | 1 | 0 | 0 | 0 | 0 | 0 |
| Nepeta macrosiphon | 0 | 0 | 0 | 1 | 1 | 1 | 1 | 0 | 0 |
| Nepeta menthoides | 0 | 0 | 1 | 0 | 1 | 0 | 1 | 0 | 0 |
| Nepeta monocephala | 0 | 0 | 0 | 1 | 0 | 0 | 0 | 0 | 0 |
| Nepeta natanzensis | 0 | 0 | 0 | 1 | 0 | 0 | 0 | 0 | 0 |
| Nepeta oxyodonta | 0 | 0 | 0 | 1 | 0 | 0 | 0 | 0 | 0 |
| Nepeta pilinux | 1 | 0 | 0 | 0 | 0 | 0 | 0 | 0 | 0 |
| Nepeta pogonosperma | 0 | 0 | 1 | 0 | 0 | 0 | 0 | 1 | 0 |
| Nepeta racemosa | 0 | 1 | 1 | 0 | 1 | 1 | 0 | 0 | 0 |
| Nepeta rivularis | 0 | 0 | 0 | 1 | 0 | 0 | 0 | 0 | 0 |
| Nepeta sahandica | 0 | 0 | 1 | 0 | 1 | 0 | 0 | 0 | 0 |
| Nepeta sessilifolia | 0 | 0 | 0 | 1 | 0 | 0 | 0 | 0 | 0 |
| Nepeta stenantha | 0 | 1 | 1 | 0 | 0 | 1 | 0 | 0 | 0 |
| Nonea macrantha | 0 | 0 | 0 | 0 | 0 | 1 | 0 | 0 | 0 |
| Nonea persica | 0 | 0 | 1 | 1 | 1 | 1 | 1 | 0 | 0 |
| Nonea pulmonarioides | 0 | 1 | 0 | 0 | 0 | 0 | 0 | 0 | 0 |
| Omphalodes luciliae | 1 | 0 | 0 | 1 | 0 | 1 | 1 | 0 | 0 |
| Onobrychis argaea | 1 | 0 | 0 | 0 | 0 | 0 | 0 | 0 | 0 |
| Onobrychis garinensis | 0 | 0 | 0 | 1 | 0 | 0 | 0 | 0 | 0 |
| Onobrychis marashensis | 1 | 0 | 0 | 0 | 0 | 0 | 0 | 0 | 0 |
| Onobrychis plantago | 0 | 0 | 0 | 1 | 0 | 0 | 0 | 0 | 0 |
| Onobrychis transcaucasica | 0 | 1 | 0 | 0 | 0 | 1 | 0 | 0 | 1 |
| Ononis sessilifolia | 1 | 0 | 0 | 0 | 0 | 0 | 0 | 0 | 0 |
| Onosma ghahremanii | 0 | 0 | 1 | 0 | 0 | 0 | 0 | 0 | 0 |
| Onosma haussknechtii | 0 | 1 | 0 | 0 | 0 | 1 | 0 | 0 | 0 |
| Onosma kilouyensis | 0 | 0 | 1 | 1 | 0 | 0 | 0 | 0 | 0 |
| Onosma liparioides | 0 | 1 | 0 | 0 | 0 | 0 | 0 | 0 | 0 |
| Onosma mirabilis | 0 | 1 | 0 | 0 | 0 | 0 | 0 | 0 | 0 |
| Onosma moussavi | 0 | 0 | 0 | 0 | 1 | 0 | 0 | 0 | 0 |
| Onosma proballanthera | 0 | 0 | 0 | 0 | 0 | 1 | 0 | 0 | 0 |
| Onosma sabalanica | 0 | 0 | 0 | 0 | 1 | 0 | 0 | 0 | 0 |
| Onosma stenosiphon | 0 | 0 | 1 | 1 | 0 | 0 | 0 | 0 | 0 |
| Orobanche gamosepala | 0 | 1 | 0 | 0 | 0 | 0 | 0 | 0 | 0 |
| Oxytropis aellenii | 0 | 0 | 1 | 0 | 0 | 0 | 0 | 0 | 0 |
| Oxytropis armeniaca | 0 | 1 | 0 | 0 | 0 | 0 | 0 | 0 | 0 |
| Oxytropis cinerea | 0 | 0 | 1 | 0 | 0 | 0 | 0 | 0 | 0 |
| Oxytropis guilanica | 0 | 0 | 1 | 0 | 0 | 0 | 0 | 0 | 0 |
| Oxytropis Iranica | 0 | 0 | 1 | 0 | 0 | 0 | 0 | 0 | 0 |
| Oxytropis javaherdehi | 0 | 0 | 1 | 0 | 0 | 0 | 0 | 0 | 0 |
| Oxytropis karjaginii | 0 | 1 | 1 | 1 | 1 | 1 | 0 | 1 | 0 |
| Oxytropis kermanica | 0 | 0 | 1 | 1 | 0 | 0 | 0 | 0 | 0 |
| Oxytropis lazica | 0 | 1 | 0 | 0 | 0 | 0 | 0 | 0 | 1 |
| Oxytropis mahneshanensis | 0 | 0 | 0 | 0 | 0 | 0 | 0 | 1 | 0 |
| Oxytropis masanderanensis | 0 | 0 | 1 | 1 | 0 | 0 | 0 | 0 | 0 |
| Oxytropis persica | 1 | 1 | 1 | 0 | 0 | 1 | 1 | 0 | 0 |
| Oxytropis savellanica | 0 | 1 | 0 | 0 | 1 | 1 | 1 | 0 | 0 |
| Oxytropis shahvarica | 0 | 0 | 1 | 0 | 0 | 0 | 0 | 0 | 0 |
| Oxytropis shirkuhi | 0 | 0 | 0 | 1 | 0 | 0 | 0 | 0 | 0 |
| Oxytropis sivehensis | 0 | 0 | 0 | 0 | 0 | 0 | 1 | 0 | 0 |
| Oxytropis surmandehi | 0 | 0 | 0 | 1 | 0 | 0 | 0 | 0 | 0 |
| Oxytropis sutakensis | 0 | 0 | 1 | 0 | 0 | 0 | 0 | 0 | 0 |
| Oxytropis takhti-soleimanii | 0 | 0 | 1 | 0 | 0 | 0 | 0 | 0 | 0 |
| Oxytropis yazdi | 0 | 0 | 0 | 1 | 0 | 0 | 0 | 0 | 0 |
| Papaver armeniacum | 0 | 1 | 1 | 0 | 1 | 1 | 0 | 0 | 0 |
| Papaver bracteatum | 1 | 1 | 1 | 1 | 1 | 1 | 0 | 0 | 0 |
| Papaver fugax | 1 | 1 | 1 | 1 | 1 | 1 | 1 | 1 | 0 |
| Papaver gabrielianae | 0 | 1 | 0 | 0 | 0 | 0 | 0 | 0 | 0 |
| Papaver orientale | 0 | 1 | 1 | 0 | 1 | 1 | 0 | 0 | 0 |
| Papaver polychaetum | 1 | 0 | 0 | 0 | 0 | 0 | 0 | 0 | 0 |
| Papaver pseudo-orientale | 0 | 1 | 1 | 0 | 1 | 1 | 0 | 1 | 0 |
| Papaver sjunicicum | 0 | 1 | 0 | 0 | 0 | 0 | 0 | 0 | 0 |
| Paracaryum lalezarense | 0 | 0 | 0 | 1 | 0 | 0 | 0 | 0 | 0 |
| Paracaryum polyanthum | 0 | 0 | 1 | 0 | 0 | 0 | 0 | 0 | 0 |
| Paracolpodium tzvelevii | 0 | 1 | 0 | 0 | 0 | 0 | 0 | 0 | 0 |
| Paraquilegia caespitosa | 0 | 0 | 1 | 0 | 0 | 0 | 0 | 0 | 0 |
| Paronychia saxatilis | 0 | 0 | 0 | 0 | 0 | 1 | 0 | 0 | 0 |
| Paronychia turcica | 0 | 0 | 0 | 0 | 0 | 1 | 0 | 0 | 0 |
| Pedicularis cadmea | 1 | 1 | 0 | 0 | 0 | 0 | 0 | 0 | 0 |
| Pedicularis caucasica | 0 | 1 | 1 | 0 | 1 | 1 | 1 | 0 | 0 |
| Pedicularis munzurdaghensis | 0 | 1 | 0 | 0 | 0 | 0 | 0 | 0 | 0 |
| Peltariopsis planisiliqua | 0 | 0 | 0 | 0 | 1 | 0 | 0 | 0 | 0 |
| Pentanema kurdistanicum | 0 | 0 | 0 | 1 | 0 | 0 | 0 | 0 | 0 |
| Petrorhagia sarbaghiae | 0 | 0 | 0 | 1 | 0 | 0 | 0 | 0 | 0 |
| Peucedanum translucens | 0 | 0 | 1 | 0 | 0 | 0 | 0 | 0 | 0 |
| Peucedanum zozimioides | 1 | 0 | 0 | 0 | 0 | 0 | 0 | 0 | 0 |
| Phagnalon persicum | 0 | 0 | 0 | 1 | 0 | 0 | 0 | 0 | 0 |
| Phelipanche zangezuri | 0 | 1 | 0 | 0 | 0 | 0 | 0 | 0 | 0 |
| Phleum iranicum | 0 | 0 | 1 | 0 | 0 | 0 | 0 | 0 | 0 |
| Phlomis anisodonta | 0 | 0 | 1 | 1 | 0 | 0 | 1 | 0 | 0 |
| Phlomis ghilanensis C. Koch | 0 | 1 | 0 | 0 | 0 | 0 | 0 | 0 | 0 |
| Physoptychis gnaphalodes | 0 | 0 | 1 | 1 | 1 | 1 | 1 | 0 | 0 |
| Pimpinella deverroides | 0 | 0 | 0 | 1 | 0 | 0 | 0 | 0 | 0 |
| Piptatherum denaense | 0 | 0 | 0 | 1 | 0 | 0 | 0 | 0 | 0 |
| Piptatherum molinioides | 0 | 0 | 0 | 1 | 0 | 0 | 0 | 0 | 0 |
| Poa bussmannii | 1 | 0 | 0 | 0 | 0 | 0 | 0 | 0 | 0 |
| Poa greuteri | 0 | 1 | 0 | 0 | 0 | 0 | 0 | 0 | 0 |
| Poa longifolia | 0 | 1 | 0 | 0 | 0 | 0 | 1 | 0 | 0 |
| Polygonum dumosum | 0 | 0 | 0 | 1 | 0 | 0 | 0 | 0 | 0 |
| Polygonum luzuloides | 0 | 1 | 0 | 1 | 1 | 1 | 1 | 0 | 1 |
| Polylophium involucratum | 0 | 0 | 1 | 0 | 0 | 0 | 0 | 0 | 0 |
| Potentilla aladaghensis | 1 | 0 | 0 | 0 | 0 | 0 | 0 | 0 | 0 |
| Potentilla anatolica | 0 | 1 | 0 | 0 | 1 | 1 | 0 | 0 | 0 |
| Potentilla argaea | 1 | 1 | 1 | 0 | 1 | 1 | 0 | 0 | 0 |
| Potentilla argyroloma | 0 | 0 | 1 | 1 | 0 | 0 | 0 | 0 | 0 |
| Potentilla aucheriana | 0 | 1 | 1 | 0 | 1 | 1 | 0 | 0 | 0 |
| Potentilla cappadocica | 0 | 1 | 0 | 0 | 0 | 0 | 0 | 0 | 0 |
| Potentilla carduchorum | 0 | 0 | 0 | 0 | 0 | 1 | 0 | 0 | 0 |
| Potentilla cryptophila | 0 | 1 | 1 | 0 | 1 | 0 | 0 | 0 | 0 |
| Potentilla diversidentata | 0 | 0 | 1 | 0 | 0 | 0 | 0 | 0 | 0 |
| Potentilla doddsii | 0 | 1 | 0 | 0 | 0 | 0 | 0 | 0 | 0 |
| Potentilla elvendensis | 0 | 0 | 0 | 1 | 0 | 0 | 0 | 0 | 0 |
| Potentilla flaccida | 0 | 0 | 1 | 1 | 0 | 0 | 0 | 0 | 0 |
| Potentilla geranioides | 0 | 1 | 0 | 0 | 0 | 1 | 0 | 0 | 0 |
| Potentilla lazica | 0 | 1 | 0 | 0 | 0 | 0 | 0 | 0 | 0 |
| Potentilla lignosa | 0 | 0 | 1 | 1 | 0 | 0 | 0 | 0 | 0 |
| Potentilla mallota | 0 | 0 | 1 | 1 | 0 | 0 | 0 | 0 | 0 |
| Potentilla meyeri | 1 | 1 | 1 | 0 | 1 | 1 | 0 | 0 | 0 |
| Potentilla nuda | 0 | 0 | 1 | 1 | 1 | 0 | 0 | 0 | 0 |
| Potentilla nurensis | 0 | 0 | 0 | 1 | 1 | 0 | 0 | 0 | 0 |
| Potentilla pannosa | 0 | 0 | 1 | 1 | 0 | 1 | 0 | 0 | 0 |
| Potentilla polyschista | 0 | 1 | 1 | 0 | 0 | 0 | 0 | 0 | 0 |
| Potentilla porphyrantha | 0 | 1 | 1 | 0 | 1 | 0 | 0 | 0 | 0 |
| Potentilla poteriifolia | 0 | 0 | 1 | 1 | 0 | 0 | 0 | 0 | 0 |
| Potentilla pulvinaris | 1 | 0 | 0 | 0 | 0 | 0 | 0 | 0 | 0 |
| Potentilla sangedehensis | 0 | 0 | 1 | 0 | 0 | 0 | 0 | 0 | 0 |
| Potentilla sawalensis | 0 | 1 | 0 | 0 | 0 | 0 | 0 | 0 | 0 |
| Potentilla subpalmata | 0 | 0 | 0 | 0 | 0 | 1 | 0 | 0 | 0 |
| Prangos platychlaena | 1 | 0 | 0 | 0 | 0 | 0 | 0 | 0 | 0 |
| Prangos tuberculata | 0 | 0 | 0 | 1 | 0 | 0 | 0 | 0 | 0 |
| Prenanthes glareosa | 1 | 0 | 0 | 0 | 0 | 0 | 0 | 0 | 0 |
| Psephellus khalkhalensis | 0 | 0 | 1 | 0 | 0 | 0 | 0 | 0 | 0 |
| Psephellus transcaucasicus | 0 | 1 | 0 | 0 | 0 | 0 | 0 | 0 | 0 |
| Pseudocamelina aphragmodes | 0 | 0 | 0 | 1 | 0 | 0 | 0 | 0 | 0 |
| Pseudocamelina glaucophylla | 0 | 0 | 1 | 1 | 1 | 0 | 0 | 0 | 0 |
| Pseudocamelina kermanica | 0 | 0 | 0 | 1 | 0 | 0 | 0 | 0 | 0 |
| Psychrogeton aellenii | 0 | 0 | 1 | 0 | 0 | 0 | 0 | 0 | 0 |
| Psychrogeton chionophilus | 0 | 0 | 0 | 1 | 0 | 0 | 0 | 0 | 0 |
| Psychrogeton persicus | 0 | 0 | 0 | 1 | 0 | 0 | 0 | 0 | 0 |
| Pulsatilla albana | 0 | 1 | 1 | 0 | 1 | 1 | 0 | 0 | 0 |
| Puschkinia bilgineri | 0 | 0 | 0 | 0 | 0 | 1 | 0 | 0 | 0 |
| Puschkinia kurdica | 0 | 0 | 0 | 0 | 0 | 1 | 0 | 0 | 0 |
| Puschkinia scilloides | 0 | 1 | 1 | 1 | 1 | 1 | 1 | 0 | 0 |
| Ranunculus anatolicus | 0 | 1 | 0 | 0 | 0 | 0 | 0 | 0 | 0 |
| Ranunculus aragazi | 0 | 1 | 0 | 0 | 0 | 0 | 0 | 0 | 0 |
| Ranunculus aucheri | 0 | 0 | 1 | 1 | 0 | 1 | 0 | 0 | 0 |
| Ranunculus bingoeldaghensis | 0 | 1 | 0 | 0 | 0 | 0 | 0 | 0 | 0 |
| Ranunculus brachylobus | 1 | 1 | 1 | 1 | 1 | 1 | 0 | 0 | 0 |
| Ranunculus bulbilliferus | 0 | 0 | 1 | 0 | 0 | 0 | 1 | 0 | 0 |
| Ranunculus crateris | 0 | 0 | 0 | 0 | 0 | 1 | 0 | 0 | 0 |
| Ranunculus crymophilus | 0 | 0 | 1 | 1 | 1 | 1 | 0 | 0 | 0 |
| Ranunculus dalechanensis | 0 | 0 | 0 | 1 | 0 | 0 | 0 | 0 | 0 |
| Ranunculus demissus | 1 | 0 | 0 | 0 | 0 | 0 | 0 | 0 | 0 |
| Ranunculus dissectus | 1 | 1 | 0 | 0 | 0 | 1 | 0 | 0 | 0 |
| Ranunculus diversifolius | 0 | 0 | 0 | 1 | 0 | 1 | 1 | 0 | 0 |
| Ranunculus elymaiticus | 0 | 0 | 0 | 1 | 0 | 0 | 0 | 0 | 0 |
| Ranunculus eriorrhizus | 0 | 0 | 0 | 1 | 0 | 0 | 0 | 0 | 0 |
| Ranunculus fenzlii | 1 | 1 | 0 | 0 | 0 | 1 | 0 | 0 | 0 |
| Ranunculus grandiflorus | 1 | 1 | 0 | 0 | 1 | 0 | 0 | 0 | 0 |
| Ranunculus microflorus | 0 | 0 | 0 | 1 | 0 | 0 | 0 | 0 | 0 |
| Ranunculus papyrocarpus | 0 | 0 | 0 | 1 | 0 | 0 | 0 | 0 | 0 |
| Ranunculus pichleri | 0 | 0 | 0 | 1 | 0 | 0 | 0 | 0 | 0 |
| Ranunculus renzii | 0 | 0 | 0 | 0 | 0 | 1 | 0 | 0 | 0 |
| Ranunculus sojakii | 0 | 0 | 1 | 0 | 0 | 0 | 0 | 0 | 0 |
| Ranunculus straussii | 0 | 0 | 0 | 1 | 0 | 0 | 0 | 0 | 0 |
| Ranunculus tempskyanus | 0 | 1 | 0 | 0 | 0 | 0 | 0 | 0 | 0 |
| Ranunculus termei | 0 | 0 | 0 | 1 | 0 | 0 | 0 | 0 | 0 |
| Ranunculus transcaucasicus | 0 | 1 | 0 | 0 | 1 | 0 | 0 | 0 | 0 |
| Ranunculus trichocarpus | 0 | 0 | 1 | 0 | 1 | 1 | 1 | 0 | 0 |
| Ranunculus vanensis | 0 | 0 | 0 | 0 | 0 | 1 | 0 | 0 | 0 |
| Ranunculus vermirrhizus | 0 | 1 | 0 | 0 | 0 | 0 | 0 | 0 | 0 |
| Ranunculus zenjanensis | 0 | 0 | 0 | 1 | 0 | 0 | 0 | 1 | 0 |
| Rhabdosciadium anatolyi | 0 | 0 | 0 | 0 | 0 | 1 | 0 | 0 | 0 |
| Rhabdosciadium aucheri | 0 | 0 | 0 | 1 | 0 | 0 | 1 | 0 | 0 |
| Rhabdosciadium petiolare | 0 | 0 | 0 | 1 | 0 | 0 | 0 | 0 | 0 |
| Rhamnus cornifolia | 1 | 0 | 0 | 1 | 1 | 1 | 1 | 0 | 0 |
| Rhynchocorys kurdica | 0 | 0 | 0 | 0 | 0 | 1 | 0 | 0 | 0 |
| Ribes anatolicum | 0 | 0 | 0 | 0 | 0 | 1 | 0 | 0 | 0 |
| Ricotia aucheri | 0 | 1 | 0 | 0 | 0 | 0 | 0 | 0 | 0 |
| Ricotia varians | 1 | 0 | 0 | 0 | 0 | 0 | 0 | 0 | 0 |
| Rindera albida | 0 | 0 | 0 | 1 | 0 | 1 | 0 | 0 | 0 |
| Rindera caespitosa | 1 | 1 | 0 | 0 | 0 | 1 | 0 | 0 | 0 |
| Rosularia aizoon | 1 | 1 | 0 | 0 | 0 | 1 | 0 | 0 | 0 |
| Rosularia chrysantha | 1 | 0 | 0 | 0 | 0 | 0 | 0 | 0 | 0 |
| Rosularia davisii | 0 | 0 | 0 | 0 | 0 | 1 | 0 | 0 | 0 |
| Rosularia elymaitica | 0 | 0 | 0 | 1 | 0 | 1 | 0 | 0 | 0 |
| Rosularia rechingeri | 0 | 0 | 0 | 0 | 0 | 1 | 0 | 0 | 0 |
| Rosularia sempervivum | 1 | 1 | 0 | 0 | 0 | 1 | 0 | 0 | 0 |
| Rubia caramanica | 0 | 0 | 0 | 1 | 0 | 0 | 0 | 0 | 0 |
| Rubia pauciflora | 0 | 0 | 0 | 1 | 0 | 0 | 0 | 0 | 0 |
| Rumex acetoselloides | 0 | 1 | 0 | 0 | 0 | 0 | 0 | 0 | 0 |
| Rumex angustifolius | 1 | 1 | 0 | 1 | 0 | 1 | 1 | 0 | 0 |
| Rumex elbursensis | 0 | 0 | 1 | 1 | 1 | 0 | 0 | 0 | 0 |
| Rumex gracilescens | 0 | 1 | 0 | 0 | 0 | 0 | 0 | 0 | 0 |
| Rumex ponticus | 1 | 1 | 0 | 0 | 0 | 1 | 0 | 0 | 0 |
| Salsola canescens | 0 | 1 | 1 | 1 | 0 | 1 | 1 | 0 | 0 |
| Salvia lachnocalyx | 0 | 0 | 0 | 1 | 0 | 0 | 0 | 0 | 0 |
| Salvia pachystachya | 0 | 1 | 0 | 0 | 0 | 1 | 0 | 0 | 1 |
| Salvia sahendica | 0 | 0 | 0 | 0 | 1 | 0 | 0 | 0 | 0 |
| Salvia staminea | 0 | 1 | 1 | 0 | 1 | 1 | 0 | 0 | 1 |
| Saponaria iranica | 0 | 0 | 0 | 1 | 0 | 0 | 0 | 0 | 0 |
| Saponaria pumilio | 1 | 0 | 0 | 0 | 0 | 0 | 0 | 0 | 0 |
| Satureja kallarica | 0 | 0 | 0 | 1 | 0 | 0 | 0 | 0 | 0 |
| Saxifraga iranica | 0 | 0 | 1 | 0 | 0 | 0 | 0 | 0 | 0 |
| Saxifraga koelzii | 0 | 0 | 1 | 0 | 0 | 0 | 0 | 0 | 0 |
| Saxifraga ramsarica | 0 | 0 | 1 | 0 | 0 | 0 | 0 | 0 | 0 |
| Saxifraga wendelboi | 0 | 0 | 1 | 0 | 0 | 0 | 0 | 0 | 0 |
| Scilla alinihatiana | 0 | 1 | 0 | 0 | 0 | 0 | 0 | 0 | 0 |
| Sclerochorton haussknechtii | 0 | 0 | 0 | 1 | 0 | 0 | 0 | 0 | 0 |
| Scorzonera grossheimii | 0 | 0 | 1 | 1 | 1 | 0 | 0 | 0 | 0 |
| Scorzonera intricata | 0 | 0 | 0 | 1 | 0 | 0 | 0 | 0 | 0 |
| Scorzonera karkasensis | 0 | 0 | 0 | 1 | 0 | 0 | 0 | 0 | 0 |
| Scorzonera kirpicznikovii | 0 | 0 | 1 | 0 | 0 | 0 | 0 | 0 | 0 |
| Scorzonera meyeri | 0 | 1 | 1 | 0 | 1 | 0 | 0 | 0 | 0 |
| Scorzonera nivalis | 0 | 0 | 0 | 1 | 0 | 0 | 0 | 0 | 0 |
| Scorzonera psychrophila | 0 | 0 | 0 | 1 | 0 | 0 | 0 | 0 | 0 |
| Scorzonera pygmaea | 1 | 0 | 0 | 0 | 0 | 0 | 0 | 0 | 0 |
| Scorzonera radicosa | 0 | 0 | 1 | 0 | 1 | 0 | 1 | 0 | 0 |
| Scorzonera rigida | 1 | 1 | 0 | 0 | 1 | 1 | 0 | 0 | 0 |
| Scorzonera seidlitzii | 0 | 0 | 1 | 0 | 0 | 0 | 0 | 0 | 0 |
| Scorzonera sericea | 1 | 1 | 0 | 0 | 0 | 0 | 0 | 0 | 0 |
| Scorzonera stenocephala | 0 | 0 | 1 | 1 | 0 | 0 | 0 | 0 | 0 |
| Scorzonera subaphylla | 0 | 0 | 0 | 1 | 0 | 0 | 0 | 0 | 0 |
| Scorzonera xylobasis | 0 | 0 | 1 | 0 | 0 | 0 | 0 | 0 | 0 |
| Scrophularia amplexicaulis | 0 | 1 | 1 | 1 | 1 | 1 | 0 | 0 | 0 |
| Scrophularia atroglandulosa | 0 | 0 | 0 | 0 | 0 | 0 | 1 | 0 | 0 |
| Scrophularia catariifolia | 0 | 0 | 0 | 0 | 0 | 1 | 1 | 0 | 0 |
| Scrophularia chlorantha | 0 | 1 | 0 | 0 | 0 | 1 | 0 | 0 | 0 |
| Scrophularia crassicaulis | 0 | 0 | 1 | 0 | 0 | 0 | 0 | 0 | 0 |
| Scrophularia crassiuscula | 0 | 0 | 0 | 1 | 0 | 0 | 0 | 0 | 0 |
| Scrophularia fatmae | 0 | 1 | 0 | 0 | 0 | 0 | 0 | 0 | 0 |
| Scrophularia flava | 0 | 0 | 0 | 1 | 0 | 0 | 0 | 0 | 0 |
| Scrophularia frigida | 0 | 1 | 1 | 1 | 0 | 0 | 0 | 0 | 0 |
| Scrophularia gorganica | 0 | 0 | 1 | 0 | 0 | 0 | 0 | 0 | 0 |
| Scrophularia kurdica | 0 | 0 | 0 | 0 | 0 | 1 | 0 | 0 | 0 |
| Scrophularia libanotica | 1 | 0 | 0 | 0 | 0 | 0 | 0 | 0 | 0 |
| Scrophularia pumilio | 0 | 0 | 0 | 0 | 0 | 1 | 0 | 0 | 0 |
| Scrophularia subaequiloba | 0 | 1 | 0 | 0 | 0 | 0 | 0 | 0 | 0 |
| Scrophularia subaphylla | 0 | 0 | 1 | 1 | 1 | 0 | 0 | 0 | 0 |
| Scutellaria araxensis | 0 | 0 | 0 | 1 | 0 | 0 | 0 | 0 | 0 |
| Scutellaria glechomoides | 0 | 0 | 1 | 0 | 0 | 0 | 0 | 0 | 0 |
| Scutellaria heterophylla | 1 | 0 | 0 | 0 | 0 | 1 | 0 | 0 | 0 |
| Scutellaria multicaulis | 0 | 0 | 0 | 1 | 0 | 0 | 0 | 0 | 0 |
| Scutellaria patonii | 0 | 0 | 0 | 1 | 0 | 0 | 0 | 0 | 0 |
| Scutellaria pinnatifida | 0 | 0 | 1 | 1 | 1 | 1 | 1 | 0 | 0 |
| Sedum artvinensis | 0 | 1 | 0 | 0 | 0 | 0 | 0 | 0 | 0 |
| Sedum euxinum | 0 | 1 | 0 | 0 | 0 | 0 | 0 | 0 | 0 |
| Sedum hewittii | 0 | 0 | 0 | 0 | 0 | 0 | 0 | 0 | 1 |
| Sedum kotschyanum | 1 | 1 | 0 | 1 | 0 | 1 | 0 | 0 | 0 |
| Sedum nanum | 0 | 1 | 0 | 0 | 0 | 1 | 0 | 0 | 0 |
| Sedum tenellum | 1 | 1 | 1 | 0 | 0 | 1 | 0 | 0 | 0 |
| Semenovia dichotoma | 0 | 0 | 0 | 1 | 0 | 0 | 0 | 0 | 0 |
| Semenovia frigida | 0 | 0 | 0 | 1 | 0 | 0 | 0 | 0 | 0 |
| Semenovia subscaposa | 0 | 0 | 1 | 0 | 0 | 0 | 0 | 0 | 0 |
| Semenovia suffruticosa | 0 | 0 | 0 | 1 | 0 | 0 | 0 | 0 | 0 |
| Semenovia tragioides | 0 | 0 | 1 | 1 | 1 | 0 | 0 | 0 | 0 |
| Sempervivum atropatanum | 0 | 0 | 1 | 0 | 1 | 0 | 0 | 0 | 0 |
| Sempervivum globiferum | 0 | 0 | 0 | 0 | 0 | 0 | 0 | 0 | 1 |
| Sempervivum iranicum | 0 | 0 | 1 | 0 | 0 | 0 | 0 | 0 | 0 |
| Sempervivum pisidicum | 1 | 0 | 0 | 0 | 0 | 0 | 0 | 0 | 0 |
| Sempervivum transcaucasicum | 0 | 1 | 0 | 0 | 0 | 0 | 0 | 0 | 0 |
| Sempervivum tunaekimii | 0 | 1 | 0 | 0 | 0 | 0 | 0 | 0 | 0 |
| Senecio cilicius | 0 | 1 | 0 | 0 | 0 | 1 | 0 | 0 | 0 |
| Senecio davisii | 0 | 0 | 0 | 0 | 0 | 1 | 1 | 0 | 0 |
| Senecio eligulatus | 0 | 0 | 0 | 1 | 0 | 0 | 0 | 0 | 0 |
| Senecio eriospermus | 0 | 1 | 0 | 0 | 0 | 1 | 0 | 0 | 0 |
| Senecio hypochionaeus | 1 | 1 | 0 | 0 | 0 | 0 | 0 | 0 | 0 |
| Senecio iranicus | 0 | 0 | 1 | 0 | 0 | 0 | 0 | 0 | 0 |
| Senecio jurineifolius | 1 | 0 | 0 | 0 | 0 | 0 | 0 | 0 | 0 |
| Senecio kotschyanus | 0 | 0 | 0 | 1 | 0 | 0 | 0 | 0 | 0 |
| Senecio munzurdaglarensis | 0 | 1 | 0 | 0 | 0 | 0 | 0 | 0 | 0 |
| Senecio subnivalis | 0 | 0 | 0 | 1 | 0 | 0 | 0 | 0 | 0 |
| Senecio taraxacifolius | 0 | 1 | 0 | 0 | 1 | 1 | 0 | 0 | 0 |
| Senecio vulcanicus | 0 | 0 | 1 | 0 | 0 | 0 | 0 | 0 | 0 |
| Serratula hakkiarica | 0 | 0 | 0 | 0 | 0 | 1 | 0 | 0 | 0 |
| Serratula haussknechtii | 0 | 1 | 1 | 1 | 1 | 0 | 0 | 0 | 0 |
| Serratula melanocheila | 0 | 0 | 0 | 1 | 0 | 0 | 0 | 0 | 0 |
| Sesleria araratica | 0 | 0 | 0 | 0 | 0 | 1 | 0 | 0 | 0 |
| Sesleria phleoides | 1 | 1 | 1 | 0 | 1 | 1 | 0 | 0 | 0 |
| Sideritis phlomoides | 1 | 0 | 0 | 0 | 0 | 0 | 0 | 0 | 0 |
| Silene araratica | 0 | 0 | 0 | 0 | 1 | 1 | 0 | 0 | 0 |
| Silene argaea | 1 | 0 | 0 | 0 | 0 | 0 | 0 | 0 | 0 |
| Silene azirensis | 0 | 1 | 0 | 0 | 0 | 0 | 0 | 0 | 0 |
| Silene balansae | 1 | 0 | 0 | 0 | 0 | 0 | 0 | 0 | 0 |
| Silene caroli-henrici | 0 | 0 | 0 | 1 | 0 | 0 | 0 | 0 | 0 |
| Silene cartilaginea | 0 | 0 | 0 | 0 | 0 | 1 | 0 | 0 | 0 |
| Silene caryophylloides | 1 | 1 | 0 | 0 | 0 | 0 | 0 | 0 | 0 |
| Silene caucasica | 0 | 0 | 0 | 0 | 0 | 0 | 0 | 0 | 1 |
| Silene cephalantha | 0 | 1 | 0 | 1 | 1 | 0 | 0 | 0 | 0 |
| Silene chustupica | 0 | 1 | 0 | 0 | 0 | 0 | 0 | 0 | 0 |
| Silene daenensis | 0 | 0 | 0 | 1 | 0 | 0 | 0 | 0 | 0 |
| Silene demawendica | 0 | 0 | 1 | 0 | 0 | 0 | 0 | 0 | 0 |
| Silene dianthoides | 1 | 1 | 0 | 0 | 0 | 1 | 0 | 0 | 0 |
| Silene dschuparensis | 0 | 0 | 0 | 1 | 0 | 0 | 0 | 0 | 0 |
| Silene erciyesdaghensis | 1 | 0 | 0 | 0 | 0 | 0 | 0 | 0 | 0 |
| Silene eremicana | 0 | 0 | 0 | 1 | 0 | 1 | 0 | 0 | 0 |
| Silene ghahremaninejadii | 0 | 0 | 0 | 1 | 0 | 0 | 0 | 0 | 0 |
| Silene goniocaula | 0 | 0 | 0 | 1 | 1 | 0 | 1 | 0 | 0 |
| Silene gynodioica | 0 | 0 | 1 | 1 | 0 | 0 | 0 | 0 | 0 |
| Silene hirticalyx | 0 | 0 | 0 | 1 | 0 | 0 | 0 | 0 | 0 |
| Silene konuralpii | 0 | 1 | 0 | 0 | 0 | 0 | 0 | 0 | 0 |
| Silene lasiantha | 0 | 1 | 0 | 0 | 0 | 1 | 0 | 0 | 0 |
| Silene laxa | 1 | 1 | 1 | 1 | 0 | 1 | 0 | 0 | 0 |
| Silene lucida | 0 | 0 | 0 | 0 | 0 | 1 | 0 | 0 | 1 |
| Silene marschallii | 0 | 0 | 1 | 0 | 1 | 0 | 1 | 0 | 0 |
| Silene meyeri | 0 | 1 | 1 | 1 | 1 | 0 | 0 | 1 | 0 |
| Silene miksensis | 0 | 0 | 0 | 0 | 0 | 1 | 0 | 0 | 0 |
| Silene nuncupanda | 1 | 0 | 0 | 0 | 0 | 0 | 0 | 0 | 0 |
| Silene nurensis | 0 | 0 | 0 | 1 | 0 | 0 | 0 | 1 | 0 |
| Silene odontopetala | 1 | 1 | 1 | 0 | 0 | 1 | 1 | 0 | 0 |
| Silene oreades | 1 | 0 | 0 | 0 | 0 | 0 | 0 | 0 | 0 |
| Silene orientoalborzensis | 0 | 0 | 1 | 0 | 0 | 0 | 0 | 0 | 0 |
| Silene oxelmanii | 0 | 0 | 0 | 1 | 0 | 0 | 0 | 0 | 0 |
| Silene persica | 0 | 0 | 0 | 1 | 0 | 0 | 0 | 0 | 0 |
| Silene pseudonurensis | 0 | 0 | 0 | 1 | 0 | 0 | 0 | 0 | 0 |
| Silene pungens | 1 | 1 | 0 | 0 | 1 | 1 | 1 | 0 | 0 |
| Silene rhynchocarpa | 1 | 1 | 0 | 1 | 0 | 1 | 0 | 0 | 0 |
| Silene tragacantha | 0 | 0 | 0 | 1 | 0 | 0 | 0 | 0 | 0 |
| Silene yildirimlii | 0 | 1 | 0 | 0 | 0 | 0 | 0 | 0 | 0 |
| Sorbus tamamschjanae | 0 | 1 | 0 | 0 | 0 | 1 | 0 | 0 | 0 |
| Stachys acerosa | 0 | 0 | 0 | 1 | 0 | 0 | 0 | 0 | 0 |
| Stachys balansae | 0 | 0 | 0 | 0 | 0 | 1 | 0 | 0 | 0 |
| Stachys choruhensis | 0 | 1 | 0 | 0 | 0 | 0 | 0 | 0 | 0 |
| Stachys citrina | 1 | 0 | 0 | 0 | 0 | 0 | 0 | 0 | 0 |
| Stachys lanigera | 0 | 0 | 0 | 1 | 0 | 0 | 1 | 0 | 0 |
| Stachys obtusicrena | 0 | 0 | 0 | 1 | 0 | 0 | 0 | 0 | 0 |
| Stachys pilifera | 0 | 0 | 0 | 1 | 0 | 0 | 0 | 0 | 0 |
| Stachys rizeensis | 0 | 1 | 0 | 0 | 0 | 0 | 0 | 0 | 0 |
| Stachys subnuda | 0 | 1 | 0 | 0 | 0 | 1 | 0 | 0 | 0 |
| Stefanoffia insoluta | 1 | 0 | 0 | 0 | 0 | 0 | 0 | 0 | 0 |
| Stellaria persica | 0 | 0 | 1 | 1 | 1 | 0 | 0 | 0 | 0 |
| Stellaria scaturiginella | 0 | 0 | 1 | 0 | 0 | 0 | 0 | 0 | 0 |
| Stenotaenia elbursensis | 0 | 0 | 1 | 0 | 0 | 0 | 0 | 0 | 0 |
| Stenotaenia haussknechtii | 0 | 0 | 0 | 1 | 0 | 0 | 0 | 0 | 0 |
| Stenotaenia nudicaulis | 0 | 0 | 1 | 0 | 1 | 0 | 0 | 0 | 0 |
| Swertia longifolia | 1 | 1 | 1 | 1 | 0 | 1 | 0 | 0 | 0 |
| Tanacetum bachtiaricum | 0 | 0 | 0 | 1 | 0 | 0 | 0 | 0 | 0 |
| Tanacetum cappadocicum | 0 | 1 | 0 | 0 | 0 | 0 | 0 | 0 | 0 |
| Tanacetum dumosum | 0 | 0 | 0 | 1 | 0 | 0 | 0 | 0 | 0 |
| Tanacetum hololeucum | 0 | 0 | 1 | 0 | 0 | 0 | 0 | 0 | 0 |
| Tanacetum kotschyi | 1 | 1 | 1 | 1 | 1 | 1 | 1 | 0 | 0 |
| Tanacetum mucroniferum | 0 | 1 | 0 | 0 | 0 | 0 | 0 | 0 | 1 |
| Tanacetum nitens | 1 | 1 | 0 | 0 | 0 | 1 | 0 | 0 | 0 |
| Tanacetum nivale | 0 | 0 | 0 | 0 | 0 | 1 | 0 | 0 | 0 |
| Tanacetum persicum | 0 | 0 | 1 | 1 | 0 | 0 | 1 | 1 | 0 |
| Tanacetum polycephalum | 0 | 0 | 1 | 1 | 1 | 1 | 0 | 0 | 0 |
| Tanacetum tenuisectum | 0 | 0 | 1 | 0 | 0 | 0 | 0 | 0 | 0 |
| Tanacetum zahlbruckneri | 0 | 0 | 0 | 0 | 0 | 1 | 0 | 0 | 0 |
| Tanacetum zangezuricum | 0 | 1 | 0 | 0 | 0 | 0 | 0 | 0 | 0 |
| Taraxacum neospurium | 0 | 0 | 1 | 0 | 0 | 0 | 1 | 0 | 0 |
| Taraxacum oliganthum | 1 | 0 | 1 | 0 | 0 | 1 | 0 | 0 | 0 |
| Taraxacum primigenium | 0 | 0 | 0 | 1 | 0 | 0 | 1 | 0 | 0 |
| Taraxacum scolopendrinum | 0 | 0 | 0 | 0 | 0 | 0 | 0 | 0 | 1 |
| Tetrataenium lasiopetalum | 0 | 0 | 0 | 1 | 0 | 1 | 1 | 1 | 0 |
| Teucrium ozturkii | 0 | 1 | 0 | 0 | 0 | 0 | 0 | 0 | 0 |
| Thesium cilicicum | 1 | 0 | 0 | 0 | 0 | 0 | 0 | 0 | 0 |
| Thlaspi crassum | 1 | 0 | 0 | 0 | 0 | 0 | 0 | 0 | 0 |
| Thlaspi kurdicum | 0 | 0 | 0 | 0 | 0 | 1 | 1 | 0 | 0 |
| Thlaspi maassoumii | 0 | 0 | 1 | 0 | 0 | 0 | 0 | 0 | 0 |
| Thlaspi pulvinatum | 0 | 0 | 0 | 0 | 0 | 1 | 0 | 0 | 0 |
| Thlaspi sintenisii | 0 | 1 | 0 | 0 | 0 | 0 | 0 | 0 | 0 |
| Thlaspi stenocarpum | 0 | 0 | 1 | 0 | 0 | 0 | 0 | 0 | 0 |
| Thlaspi tenue | 0 | 0 | 0 | 0 | 1 | 0 | 0 | 1 | 0 |
| Thlaspi valerianoides | 0 | 0 | 0 | 0 | 1 | 1 | 0 | 0 | 0 |
| Thlaspi watsonii | 0 | 0 | 0 | 0 | 0 | 1 | 0 | 0 | 0 |
| Thymus brachychilus | 1 | 1 | 0 | 0 | 0 | 1 | 0 | 0 | 0 |
| Thymus carmanicus | 0 | 0 | 1 | 1 | 0 | 0 | 1 | 0 | 0 |
| Thymus cherlerioides | 1 | 0 | 0 | 0 | 0 | 0 | 0 | 0 | 0 |
| Thymus fallax | 0 | 0 | 1 | 1 | 0 | 0 | 0 | 0 | 0 |
| Thymus fedtschenkoi | 0 | 1 | 0 | 0 | 0 | 1 | 0 | 0 | 1 |
| Thymus praecox | 0 | 1 | 1 | 1 | 1 | 1 | 0 | 0 | 0 |
| Thymus pubescens | 0 | 1 | 1 | 1 | 1 | 1 | 0 | 1 | 1 |
| Trachydium depressum | 0 | 0 | 1 | 1 | 0 | 1 | 1 | 0 | 0 |
| Trachydium eriocarpum | 0 | 0 | 1 | 0 | 0 | 0 | 0 | 0 | 0 |
| Trachydium kotschyi | 0 | 0 | 0 | 1 | 0 | 0 | 0 | 0 | 0 |
| Trachydium pauciradiatum | 0 | 0 | 1 | 0 | 0 | 0 | 0 | 0 | 0 |
| Tragopogon erostris | 0 | 0 | 0 | 1 | 0 | 0 | 0 | 0 | 0 |
| Tragopogon jesdianus | 0 | 0 | 1 | 1 | 0 | 0 | 0 | 0 | 0 |
| Tragopogon kotschyi | 0 | 0 | 1 | 0 | 0 | 0 | 0 | 0 | 0 |
| Tragopogon reticulatus | 0 | 1 | 0 | 0 | 0 | 1 | 0 | 0 | 0 |
| Trifolium kurdistanicum | 0 | 0 | 0 | 1 | 0 | 0 | 0 | 0 | 0 |
| Trifolium longidentatum | 0 | 0 | 0 | 0 | 1 | 1 | 0 | 0 | 0 |
| Trifolium montanum | 0 | 1 | 0 | 0 | 0 | 1 | 0 | 0 | 0 |
| Trifolium radicosum | 0 | 0 | 1 | 0 | 0 | 0 | 1 | 0 | 0 |
| Trifolium sintenisii | 0 | 1 | 0 | 0 | 0 | 0 | 0 | 0 | 0 |
| Tripleurospermum melanolepis | 0 | 1 | 0 | 0 | 0 | 1 | 0 | 0 | 0 |
| Trisetum geghamense | 0 | 1 | 0 | 0 | 0 | 0 | 0 | 0 | 0 |
| Trisetum thospiticum | 0 | 0 | 0 | 0 | 0 | 1 | 0 | 0 | 0 |
| Trisetum turcicum | 1 | 1 | 0 | 0 | 0 | 1 | 0 | 0 | 0 |
| Trollius ranunculoides | 1 | 1 | 0 | 0 | 0 | 1 | 0 | 0 | 0 |
| Tulipa humilis | 1 | 0 | 1 | 1 | 1 | 1 | 0 | 0 | 0 |
| Tulipa koyuncui | 0 | 0 | 0 | 0 | 1 | 0 | 0 | 0 | 0 |
| Valeriana alpestris | 0 | 1 | 0 | 0 | 0 | 1 | 0 | 0 | 0 |
| Valeriana bolkarica | 1 | 0 | 0 | 0 | 0 | 0 | 0 | 0 | 0 |
| Valeriana saxicola | 0 | 1 | 0 | 0 | 0 | 0 | 0 | 0 | 0 |
| Vania campylophylla | 0 | 0 | 0 | 0 | 0 | 1 | 0 | 0 | 0 |
| Vavilovia formosa | 1 | 1 | 1 | 0 | 0 | 1 | 1 | 0 | 0 |
| Verbascum bornmuellerianum | 0 | 0 | 0 | 1 | 0 | 0 | 1 | 0 | 0 |
| Verbascum bourgeauanum | 1 | 0 | 0 | 0 | 0 | 0 | 0 | 0 | 0 |
| Verbascum carmanicum | 0 | 0 | 0 | 1 | 0 | 0 | 0 | 0 | 0 |
| Verbascum faik-karaveliogullarii | 0 | 0 | 0 | 0 | 0 | 1 | 0 | 0 | 0 |
| Verbascum georgicum | 0 | 1 | 0 | 0 | 0 | 0 | 0 | 0 | 0 |
| Verbascum kurdistanicum | 0 | 0 | 0 | 0 | 0 | 1 | 0 | 0 | 0 |
| Verbascum speciosum | 0 | 0 | 0 | 0 | 0 | 1 | 0 | 0 | 0 |
| Verbascum subnivale | 1 | 0 | 0 | 0 | 0 | 0 | 0 | 0 | 0 |
| Verbascum tauri | 1 | 0 | 0 | 0 | 0 | 0 | 0 | 0 | 0 |
| Veronica allahuekberensis | 0 | 1 | 0 | 0 | 0 | 0 | 0 | 0 | 0 |
| Veronica armena | 0 | 1 | 0 | 0 | 0 | 0 | 0 | 0 | 0 |
| Veronica aucheri | 0 | 0 | 1 | 0 | 0 | 0 | 0 | 0 | 0 |
| Veronica baranetzkii | 0 | 1 | 0 | 0 | 0 | 0 | 0 | 0 | 0 |
| Veronica beccabunga | 1 | 1 | 1 | 0 | 1 | 1 | 1 | 0 | 0 |
| Veronica bombycina | 1 | 0 | 0 | 0 | 0 | 1 | 0 | 0 | 0 |
| Veronica caespitosa | 1 | 1 | 0 | 0 | 0 | 0 | 0 | 0 | 0 |
| Veronica daranica | 0 | 0 | 0 | 1 | 0 | 0 | 0 | 0 | 0 |
| Veronica davisii | 0 | 0 | 0 | 0 | 0 | 1 | 0 | 0 | 0 |
| Veronica euphrasiifolia Link | 0 | 1 | 0 | 0 | 0 | 0 | 0 | 0 | 0 |
| Veronica fragilis | 0 | 0 | 0 | 1 | 0 | 0 | 0 | 0 | 0 |
| Veronica fridericae | 0 | 0 | 0 | 0 | 0 | 1 | 0 | 0 | 0 |
| Veronica gentianoides | 1 | 1 | 1 | 0 | 1 | 1 | 1 | 0 | 0 |
| Veronica kopgecidiensis | 0 | 1 | 0 | 0 | 0 | 0 | 0 | 0 | 0 |
| Veronica kotschyana | 1 | 0 | 0 | 0 | 0 | 0 | 0 | 0 | 0 |
| Veronica kurdica | 0 | 0 | 1 | 1 | 0 | 0 | 0 | 0 | 0 |
| Veronica longipedicellata | 0 | 0 | 1 | 0 | 0 | 0 | 0 | 0 | 0 |
| Veronica mirabilis | 0 | 0 | 1 | 0 | 0 | 0 | 0 | 0 | 0 |
| Veronica montbretii | 0 | 1 | 0 | 0 | 0 | 0 | 0 | 0 | 0 |
| Veronica orientalis | 0 | 1 | 1 | 1 | 1 | 1 | 1 | 1 | 0 |
| Veronica paederotae | 0 | 0 | 1 | 0 | 0 | 0 | 0 | 0 | 0 |
| Veronica polium | 0 | 0 | 0 | 0 | 0 | 1 | 0 | 0 | 0 |
| Veronica rechingeri | 0 | 0 | 1 | 0 | 0 | 0 | 0 | 0 | 0 |
| Veronica rubrifolia | 0 | 0 | 1 | 1 | 0 | 0 | 0 | 0 | 0 |
| Veronica surculosa | 1 | 0 | 0 | 0 | 0 | 0 | 0 | 0 | 0 |
| Veronica tauricola | 1 | 0 | 0 | 0 | 0 | 1 | 0 | 0 | 0 |
| Veronica telephiifolia | 0 | 1 | 0 | 0 | 0 | 1 | 0 | 0 | 0 |
| Veronica thymoides | 0 | 0 | 0 | 0 | 0 | 1 | 0 | 0 | 0 |
| Vicia alpestris | 1 | 1 | 0 | 0 | 0 | 1 | 0 | 0 | 0 |
| Vicia canescens | 1 | 1 | 1 | 0 | 1 | 1 | 1 | 0 | 0 |
| Vicia ciceroidea | 0 | 1 | 1 | 1 | 0 | 1 | 1 | 0 | 0 |
| Vicia glareosa | 0 | 1 | 0 | 0 | 0 | 0 | 0 | 0 | 0 |
| Vicia multijuga | 0 | 0 | 1 | 0 | 1 | 0 | 0 | 0 | 0 |
| Viola pachyrrhiza | 0 | 0 | 0 | 1 | 0 | 0 | 0 | 0 | 0 |
| Viola spathulata | 0 | 0 | 1 | 0 | 0 | 0 | 0 | 0 | 0 |
| Xanthogalum purpurascens | 1 | 1 | 1 | 0 | 1 | 1 | 0 | 0 | 0 |
| Zeravschania aucheri | 0 | 0 | 1 | 1 | 1 | 1 | 0 | 0 | 0 |
| Zerdana anchonioides | 0 | 0 | 0 | 1 | 0 | 0 | 0 | 0 | 0 |
| Ziziphora clinopodioides | 1 | 1 | 1 | 0 | 1 | 1 | 0 | 0 | 0 |
| **Total** | **231** | **429** | **355** | **388** | **216** | **392** | **116** | **34** | **48** |

**Figure S1.** ACDs of the Irano-Anatolian region identified using Endemicity Analysis based on non-endemic alpine species (a set must have at least 3 contributing species).


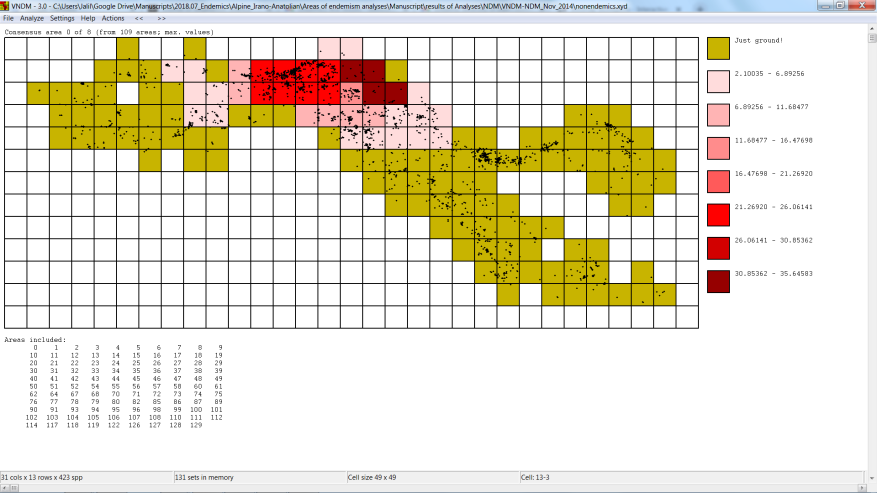

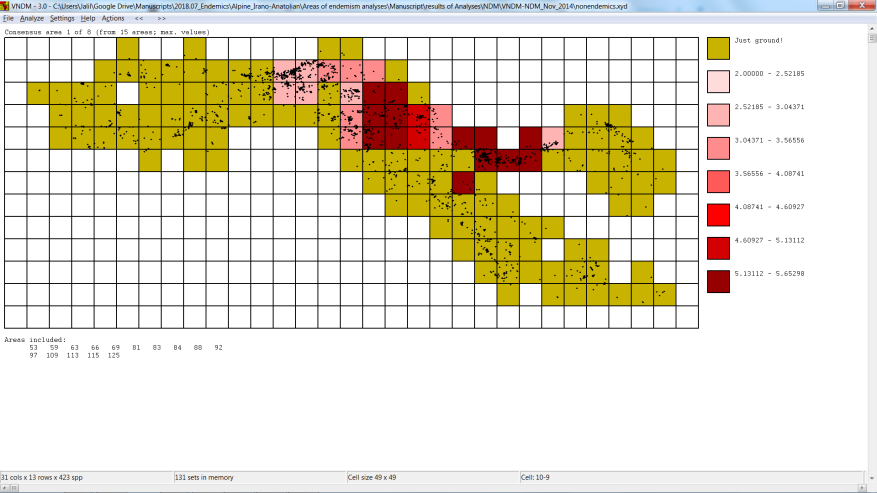

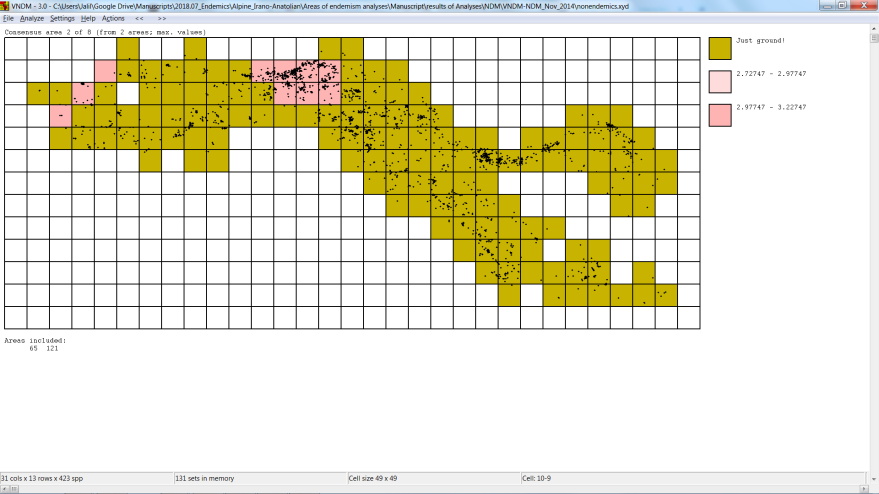

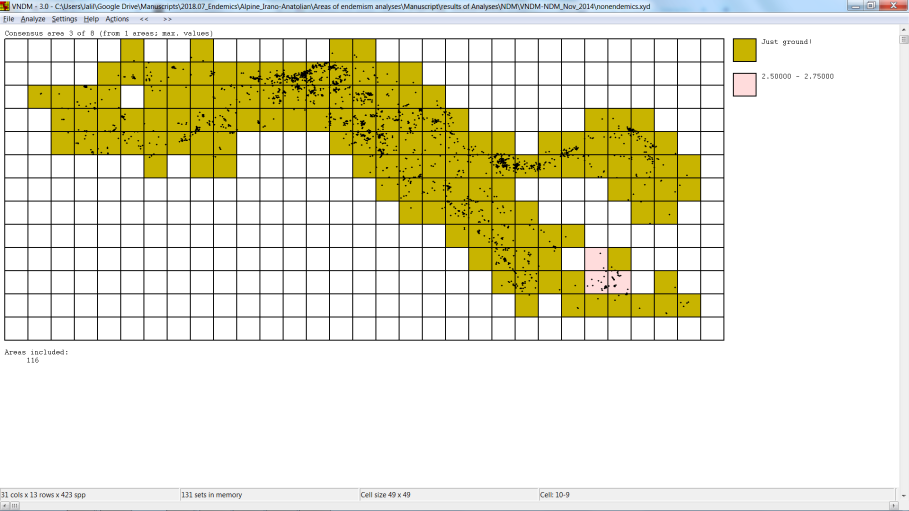

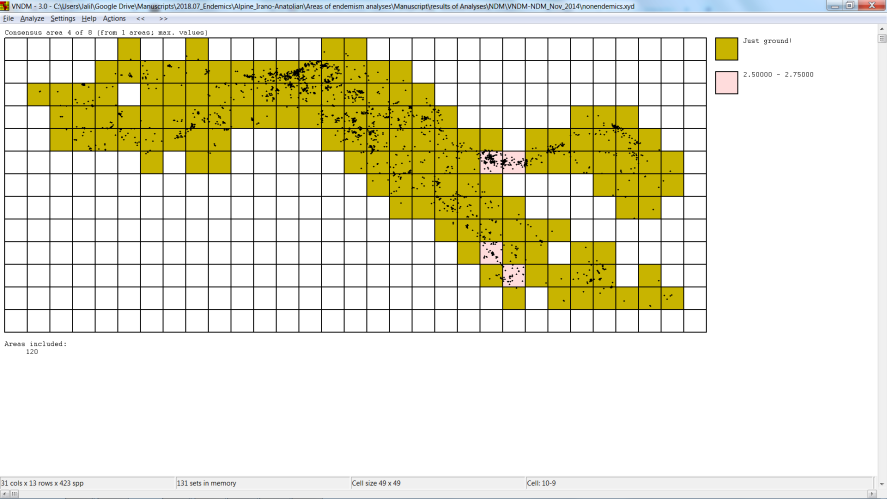

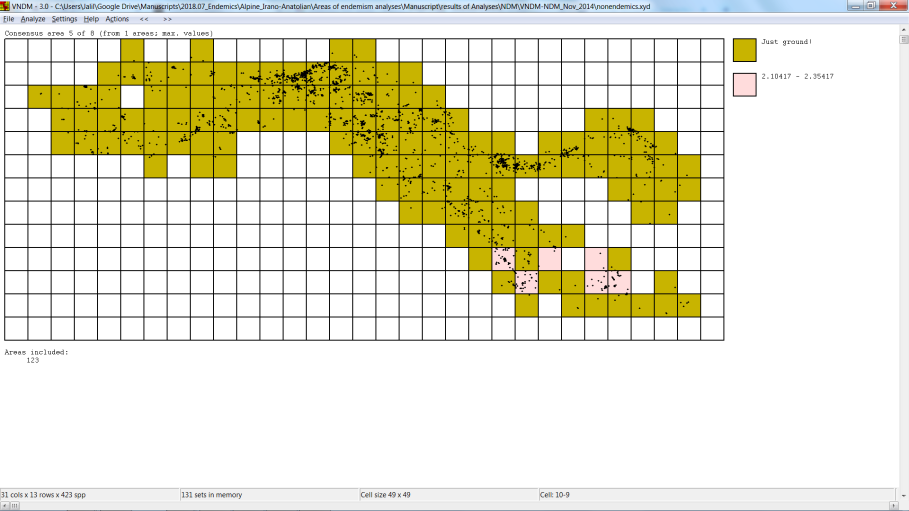

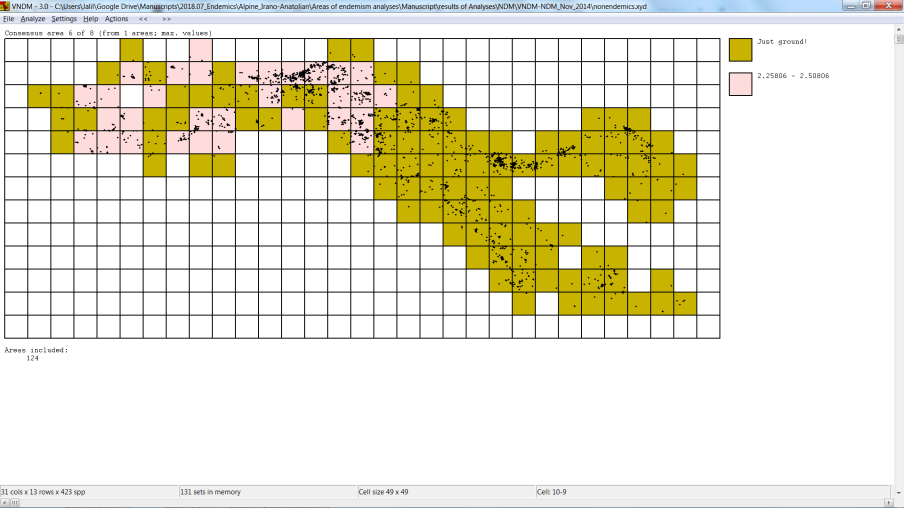

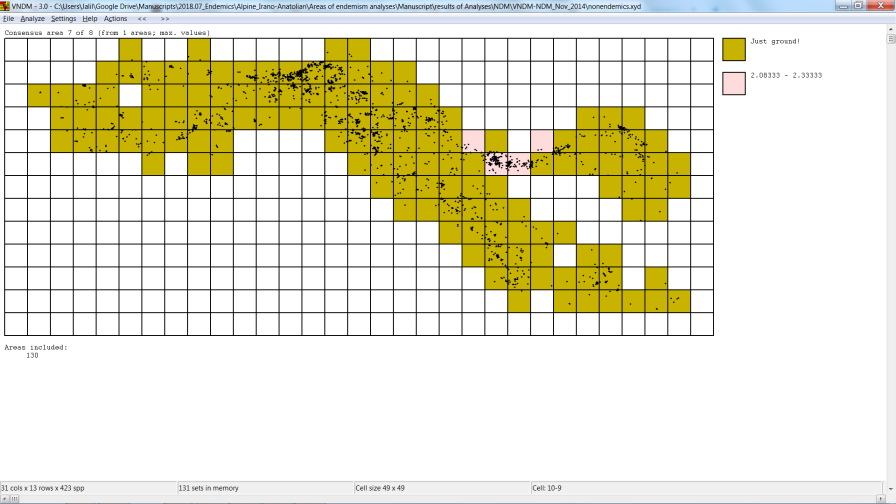


**Figure S2.** Identified units using Network-Clustering approach based on non-endemic alpine species.


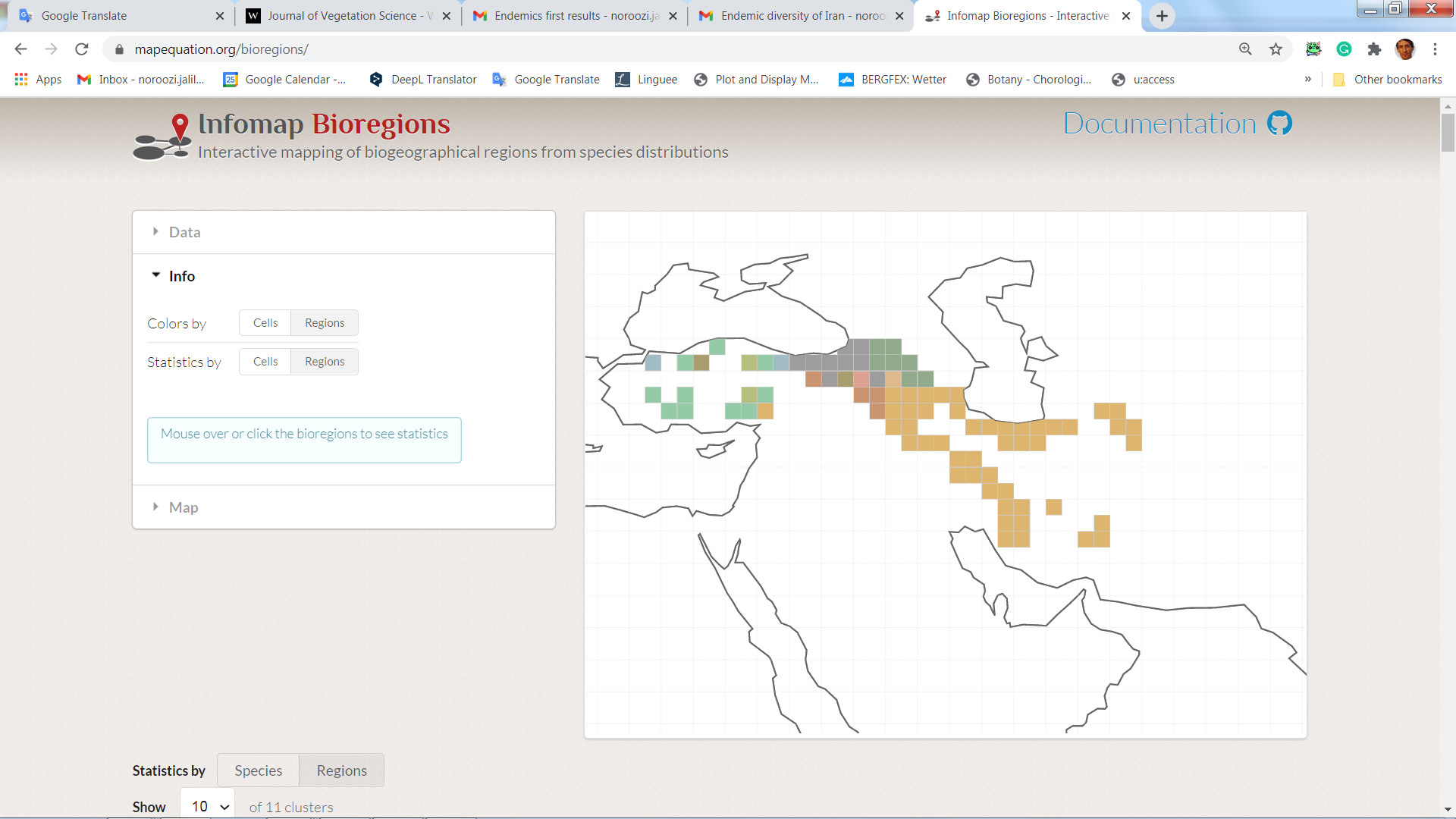

Supplement: Supplementary file 1 — Supplementary file1 (DOCX 1242 KB) [file 35_2021_266_MOESM1_ESM.docx]
